# The First Report of miRNAs from a Thysanopteran Insect, *Thrips palmi* Karny Using High-Throughput Sequencing

**DOI:** 10.1371/journal.pone.0163635

**Published:** 2016-09-29

**Authors:** K. B. Rebijith, R. Asokan, H. Ranjitha Hande, N. K. Krishna Kumar

**Affiliations:** 1 Division of Biotechnology, Indian Institute of Horticultural Research, Bangalore, India; 2 Division of Horticultural Science, Indian Council of Agricultural Research, New Delhi, India; Huazhong University of Science and Technology, CHINA

## Abstract

*Thrips palmi* Karny (Thysanoptera: Thripidae) is the sole vector of *Watermelon bud necrosis tospovirus*, where the crop loss has been estimated to be around USD 50 million annually. Chemical insecticides are of limited use in the management of *T*. *palmi* due to the thigmokinetic behaviour and development of high levels of resistance to insecticides. There is an urgent need to find out an effective futuristic management strategy, where the small RNAs especially microRNAs hold great promise as a key player in the growth and development. miRNAs are a class of short non-coding RNAs involved in regulation of gene expression either by mRNA cleavage or by translational repression. We identified and characterized a total of 77 miRNAs from *T*. *palmi* using high-throughput deep sequencing. Functional classifications of the targets for these miRNAs revealed that majority of them are involved in the regulation of transcription and translation, nucleotide binding and signal transduction. We have also validated few of these miRNAs employing stem-loop RT-PCR, qRT-PCR and Northern blot. The present study not only provides an in-depth understanding of the biological and physiological roles of miRNAs in governing gene expression but may also lead as an invaluable tool for the management of thysanopteran insects in the future.

## Introduction

MicroRNAs (miRNAs) are a family of small (~18–25 nucleotides (nts)), endogenously initiated, non-coding RNAs (ncRNAs) that primarily regulate gene expression in animals, plants and protozoan in a sequence-specific manner. In mammals, approximately 60% of protein-coding gene activities are under the control of miRNAs and they regulate almost every cellular process investigated [1,2]. miRNAs can regulate gene expression either by translation repression or by degradation of mRNA through deadenylation [3]. The second to seventh nucleotides in the 5' end of the miRNA form the “seed” region that provides the most of the pairing specificity [4,5]. miRNA-mediated regulation plays a key role in cellular and developmental processes such as cell division, cell death, disease, hormone secretion and neural development [6–9]. *Lin-4* was the first member of the miRNA family, discovered in *Caenorhabditis elegans*, which regulates the timing of larval development [10]. Subsequently, many miRNAs have been revealed from wide varieties of organisms including insects [11], plants [12], viruses [13] and vertebrates [14].

The majority of miRNAs are ~22 nts in length and the biogenesis of miRNAs is a multiple step process that is widely conserved among eukaryotes. miRNA biogenesis requires RNAase III-like enzymes, Drosha and Pasha (DGCR8, in vertebrates), for generating pre-miRNA (~70 nts) from the primary miRNA (pri-miRNA) transcript [12] which are translocated to the cytoplasm by exportin-5 [15]. Another class of RNAase III enzyme, Dicer (1 & 2 in insects), produces a ~22 bp miRNA:miRNA* duplex from the previously generated pre-miRNA in the cytoplasm [16,17]. Mature miRNAs are then selectively loaded into the RNA-induced silencing complex that contains Argonaute family proteins [5]. Thus, the mature RISC containing the guide strand recognizes the complementary mRNA and cleaves thereby inhibiting the protein translation [18]. Identification of miRNA involves three main approaches, forward genetics, bioinformatics prediction [19,20] and direct cloning and sequencing [21,22,11]. In the recent past, the high-throughput next generation sequencing (NGS) technology, for example, Illumina platform (www.illumina.com) and Roche 454 pyrosequencing platform (www.454.com) have become robust methods of identifying miRNAs from animals and plants [23–29]. As a result, several miRNAs have been reported in the recent past from various orders of insects such as Diptera, Hymenoptera, Coleoptera, Orthoptera, Lepidoptera, Hemiptera and Homoptera [27].

Thrips (Thysanoptera: Thripidae) are one of the major sucking pests on many crops and nearly 6000 species are currently described [30]. Less than 1% of them are considered as pests of agricultural and horticultural crops, and cause crop damage either by direct feeding or as vectors by transmitting plant pathogenic tospoviruses [31]. Globally, 12 species of thrips have been reported as vectors of tospoviruses [32] and among them, *T*. *palmi* is an important polyphagous pest and an extremely successful invasive species that originated in Southeast Asia but has recently spread to large parts of tropical and sub-tropical countries. In India, *Watermelon bud necrosis tospovirus* is succesfully transmitted by *T*. *palmi* adults that acquire the virus during larval stages [33]. The peculiar feature of thrips transmission is that only the nymphs can acquire the virus while the adults can transmit [34]. The rapid parthenogenetic reproduction and the feeding behavior of thrips can cause considerable crop damage. The worldwide annual loss due to tospoviruses is estimated at USD 1 billion and in Asia alone, it is over USD 89 million [34]. Currently, the management of *T*. *palmi* includes mainly chemicals such as imidacloprid and pyrethroids against which *T*. *palmi* has already developed resistance [35]. Given the paucity of genomic information of *T*. *palmi*, it is important to profile the miRNAs for understanding their regulatory role in the biology and the great promise they hold in futuristic pest management. Thus, for the first time, we identified, characterized and validated both conserved and novel miRNAs from *T*. *palmi*. Further analysis identified putative target genes for these miRNAs, which will shed more light on the identification of highly specific miRNAs that can be used shortly for thysanopteran pest management.

## Materials and Methods

### Ethical treatment of animals

No specific permissions were required for these locations/activities, and field studies did not involve endangered or protected species. Ethical approval was not required to work with thrips, the subject species in this study; *T*. *palmi* is an invertebrate and not listed on the endangered species list. Thrips are ubiquitous in their natural ranges.

### Insect Culture

*T*. *palmi* were reared on French bean pods (*Phaseolus vulgaris* cv. Arka Komal) in plastic containers (10X10 cm) at 30 ± 2°C, 80 ± 10% RH and 16:8 L:D photoperiod as described previously [36]. Total RNA was isolated from whole-body homogenates of sample mix, containing a total of 50 mg of different life stages such as eggs, larvae, pupae and adults of *T*. *palmi* using TRIzol reagent (Invitrogen, Carlsbad CA, USA).

### Library preparation and sequence data generation

Samples were processed according to Illumina TruSeq™ Small RNA sample preparation guide. Size fractionated small RNA populations (18–28 nts) were extracted, purified and ligated to 3' and 5' adapters using T4 RNA Ligase (Life Technologies, Ambion, USA). Ligated products were reverse transcribed using SuperScript II (Life Technologies, Invitrogen, USA) followed by PCR amplification with 11 cycles and two size selection gels. High-throughput sequencing of the small RNA libraries was performed on Illumina Hiseq2000.

### Bioinformatics analysis

The obtained sequence tags were subjected to a primary analysis in which low-quality tags, 3' and 5' adapter contaminants were discarded. The sequencing data were investigated against the Rfam (http://rfam.sanger.ac.uk/) and RepBase (http://www.girinst.org/repbase/) as references to annotate the ncRNAs namely, rRNAs, tRNAs, snRNAs, snoRNAs and repeat-associated small RNAs and degraded fragments of expressed genes (exons and introns) in the remaining sequences. All such mapped reads were removed from the dataset before further analysis. Remaining unique sequences were aligned with the miRNA sequences available in the miRBase (v21, http://www.miRBase.org/) to identify the conserved miRNAs. Novel miRNA candidates were identified by employing the miRDeep2 [37] and miRCat (http://srna-workbench.cmp.uea.ac.uk/tools/mircat/) software. *Frankliniella occidentalis* genome (http://www.ncbi.nlm.nih.gov/nuccore/644576459) was used as a reference to extract the potential secondary hairpin structures, employing Vienna RNAfold [38].

### Homology analysis

Homology analysis was carried out with conserved miRNAs of *T*. *palmi* with the miRNAs of other organisms from the miRBase database (Release 21.0) [39]. BLASTn embedded in the miRBase database was used to compare the *T*. *palmi* miRNAs with other species, with an E-value of 0.01 to find out more miRNA homologs. The naming of the miRNAs in this study has been done according to Griffith-Jones, et al., 2006. Since these miRNAs were predicted from *T*. *palmi*, the prefix for all miRNAs was fixed as ‘tpa’. The rest of the naming convention criteria were in accordance with miRBase [40].

### Phylogenetic analysis of microRNA family

All the identified miRNAs were classified into different miRNA precursor families (www.rfam.sanger.ac.uk). Few miRNA families (miR-279, miR-281, miR-1000 and miR-1175) were selected for phylogenetic analysis. RaxML.v.7.0.4 [41] was employed to construct the Maximum Likelihood (ML) tree with 2000 bootstrap replications.

### Prediction of miRNA targets

Target identification is crucial for understanding the biological functions of miRNAs. Unlike the plant counterparts, the imperfect complementarity of animal miRNAs with their target sequences on mRNA makes it more difficult to judge the accuracy of prediction [20]. Targets for identified miRNAs were predicted employing miRanda program [42], against the Expressed Sequence Tags (ESTs) and transcriptome (NCBI Accession: PRJNA203209) database of *F*. *occidentalis*. An alignment score [43] greater than or equal to 130 and miRNA:mRNA binding energy (Minimum Free Energy (MFE, ΔG)) less than -20 kcal/mol were considered as putative target genes. The targets were further annotated against NCBI-RefSeq invertebrate protein database and Gene Ontology (GO) terms were assigned (using Blast-2-GO) based on the annotation. The circos plot was generated using Circos [44] to visualize the interaction between miRNAs and their targets.

### Validation and quantification of *T*. *palmi* miRNAs

#### stem-loop RT-PCR

We were able to validate few of the conserved and novel microRNAs employing Stem-loop RT-PCR primers designed based on the previous reports [22]. Briefly, stem-loop RT primers bind to the 3' portion of miRNA molecules, initiating reverse transcription of the miRNAs. Later, the RT product was amplified using a miRNA specific forward primer and the universal reverse primer.

#### Reverse transcription quantitative PCR (qRT-PCR)

In the present study, we selected differentially expressed and functionally significant 13 miRNAs (nine conserved and four novel) for qRT-PCR. Briefly, Mir-X-miRNA qRT-PCR SYBR Kit (Clontech Laboratories, Inc., USA) was used for the qRT-PCR reactions, which has a single-step, single-tube reaction to produce the first-strand cDNA, which was then specifically and quantitatively amplified using a miRNA-specific primer and SYBR Advantage qPCR chemistry. All the qRT-PCR assays were conducted according to the MIQE guidelines [45]. U6 snRNA was used as an internal control gene for normalization. qRT-PCR was performed on Light Cycler 480 (Roche, USA) using 1:20 diluted cDNAs and SYBR Advantage Premix (Clontech Laboratories, Mountain View, USA), according to the manufacturer’s instructions. Assays were performed in triplicates for three independent biological experiments and the relative gene expression data were analyzed using 2^-ΔΔCT^ method [46]. The values of these three independent experiments were statistically analyzed using one-way ANOVA to calculate the statistical significance.

#### miRNA northern blot

Small RNAs were isolated from pooled *T*. *palmi* (eggs, larvae, pupae and adults) employing mirVANA miRNA isolation kit (Life Technologies, USA). 5 μg of *T*. *palmi* small RNAs were resolved on 15% polyacrylamide gel containing 8M urea. RNA was electro blotted [TransBlot SD Semi-Dry Electrophoretic transfer cell (Bio-Rad, USA)] for 90 minutes at 20V onto HyBond-N+ membrane (GE Healthcare, USA) and immobilized by UV cross-linking (Stratagene, USA). The membrane was hybridized with 5' digoxigenin-labeled locked nucleic acid probes for miRNA detection (100ng/ml, Exiqon) at 37°C overnight. Later the membranes were washed twice in 2x SSC at 37°C for 15 minutes each. Digoxigenin signals were detected with DIG Northern starter kit (Roche) according to the manufacturer’s instructions.

## Results

### Overview of the small RNA Library

**Fig 1:**
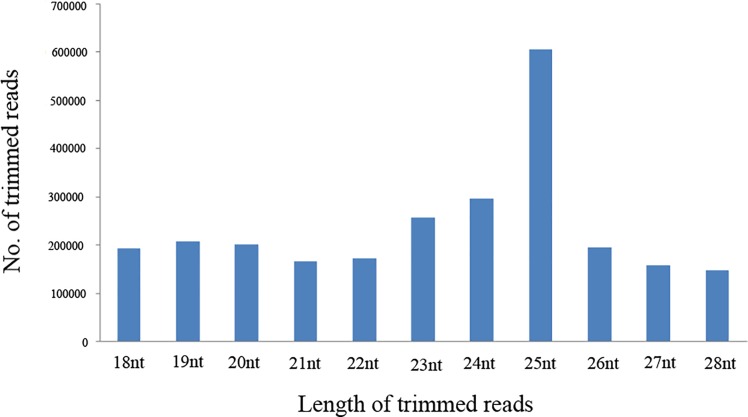
Length distribution of mappable reads obtained from *T*. *palmi* deep-sequencing. Reads with ≥ 18 nt to ≤ 26 nt were considered for miRNA mapping.

We obtained a dataset of about 14 million reads from the pooled *T*. *palmi* small RNA library (egg, larva, pupa and adult) sequenced on Illumina Next Generation Sequencing platform. After various mapping (Table 1), the trimmed high-quality small RNA reads were employed to identify both known and novel miRNAs. Size distribution of the high-quality reads in the library (Fig 1) revealed that the peak was at 25 nts, which was also observed in the pooled library of *Plutella xylostella* (L.) [47]. A small portion (<5%) of our library consisted of read length of around 26 to 28 nts, which could be putative piwi- interacting RNAs (piRNAs) from *T*. *palmi* (S1 Table).

**Table 1:** Summary Statistics of *T*. *palmi* small RNA data analysis.

		Mappable (%)
Raw reads	14,148,849	100
Number of trimmed reads	5,045,585	35.66
Mapped to mRNA	2,212,874	15.63
Repbase	1,458,987	10.31
Rfam mapped	2,817,765	19.87
Total unmappable for miRNA	2,610,489	18.45
miRBase mapped reads	19,434	< 1.0
Average length	23	

### Identification of known miRNA

MiRNAs are known to be conserved among different species within a kingdom. Here, in our study, the mappable sequences were aligned to miRNA sequences from miRBase v.21.0. The analysis resulted in a total of 67 conserved miRNAs representing 54 different miRNA families (Table 2), among which the average similarity between the homologs reached 85% and few of them had a similarity to the extent of 95–100% with 1–2 nts or no difference. Analysis of the 54 miRNA families revealed that 15 were found to be exclusively present in arthropod species (Table 3), while 25 miRNA families were vertebrate specific. Seven miRNA families (miR-10, miR-100, miR-71, miR-9, miR-92, miR-15 and miR-281) were found to be highly conserved in the Animal Kingdom (Table 3) during evolution implicating their importance in regulating the gene transcripts involved in the physiological process. Among the known miRNAs, miR-281 and miR-750 were highly expressed with an expression value of 9560 and 5849 respectively (Table 2).

**Table 2:** Expression value of known miRNAs in *T*. *palmi*. The first column represents miRNA families; the second column represents the number of reads annotated on the particular miRNA family; the third column represents mature miRNA sequences; the fourth column represents the length of mature miRNAs; the fifth column represents miRNAs falling in to related miRNA family.

miRNA family	Expression value (Reads)	Length (nt)	Name of the miRNA	Sequence (5' - 3')	Homologous miRNA
miR-750	5849	23	tpa-miR-750a	CCAGAUCUAACUCUUCCAGCUCA	isc-miR-750
	1916	22	tpa-miR-750b	CCAGAUCUAACUCUUCCAGCUC	ame-miR-750
	70	25	tpa-miR-750c	CCAGAUCUAACUCUUCCAUAUGACG	tca-miR-750-3p
	30	23	tpa-miR-750d	UCAGAUCUAACUCUUCCAGUUCU	sme-miR-750-3p
	4	22	tpa-miR-750e	CAGAUCUAACUCUUCCAGCUCA	lgi-miR-750
miR-92	1687	22	tpa-miR-92	AAUUGCACCCGUCCCGGCCUGA	ame-miR-92b
miR-281	9560	22	tpa-miR-281	AAGAGAGCUAUCCGUCGACAGU	dme-miR-281-2-5p
miR-6240	675	26	tpa-miR-6240	CCAAAGCAUCGCGAAGGCCCACGGCG	mmu-miR-6240
miR-2779	103	20	tpa-miR-2779a	AUAUCCGGCUCGAAGGACCA	bmo-miR-2779
	101	18	tpa-miR-2779b	AUCCGGCUCGAAGGACCA	mse-miR-2779
miR-2796	479	23	tpa-miR-2796	GUAGGCCGGCGGAAACUACUUGC	ame-miR-2796
miR-7550	100	18	tpa-miR-7550	AUCCGGCUCGAAGGACCA	ipu-miR-7550
miR-993	95	20	tpa-miR-993	GAAGCUCGACUCUACAGGUC	ppc-miR-993
miR-5124	85	20	tpa-miR-5124	GGUCCAGUGACUAAGAGCAU	mmu-miR-5124a
miR-9	77	18	tpa-miR-9	UCUUUGGUAUCCUAGCUG	bmo-miR-9c-5p
miR-2478	56	20	tpa-miR-2478	GUAUCCCACUUCUGACACCA	bta-miR-2478
miR-1175	64	22	tpa-miR-1175-5p	AAGUGGAGUAGUGGUCUCAUCG	aae-miR-1175-5p
	6	24	tpa-miR-1175-3p	UGAGAUUCAACUCCUCCAACUUAA	bmo-miR-1175-3p
miR-279	32	25	tpa-miR-279a	UGACUAGAUCCAUACUCGUCUAUAG	tca-miR-279d-3p
	47	22	tpa-miR-279b	UGACUAGAUCCAUACUCGUCUG	bmo-miR-279c-3p
	24	21	tpa-miR-279c	UGACUAGAUCCAUACUCAGCU	ppc-miR-279
	4	23	tpa-miR-279d	UGACUAGAUCCAUACUCGUCUGC	mse-miR-279d
miR-3931	25	23	tpa-miR-3931	UACUUUGAGUCGGUACGAAUCCU	isc-miR-3931
miR-10	21	22	tpa-miR-10	AACCCUGUAGACCCGAAUUUGA	gsa-miR-10b-5p
miR-5119	20	19	tpa-miR-5119	CAUCUCAUCCUGGGGCUGG	mmu-miR-5119
miR-800	18	22	tpa-miR-800	GCCAAACUCGGAAAUUGUCUGC	cel-miR-800-3p
miR-6990	18	21	tpa-miR-6990	CCCAGGGUGAGUCAGGGCUCU	mmu-miR-6990-5p
miR-6489	15	20	tpa-miR-6489-5p	GGCACCGGACUGGCGCCCUU	mja-miR-6489-5p
	6	23	tpa-miR-6489-3p	CGACGGAAAGGUGUCCAAGCUGG	mja-miR-6489-3p
miR-7156	15	23	tpa-miR-7156	UUGUUCUCAAACUGGCUGUCAGA	hsa-miR-7156-5p
miR-5108	14	19	tpa-miR-5108	GUAGAGCACUGGAUGGUUU	mmu-miR-5108
miR-9373	14	21	tpa-miR-9373	CAUCGCUCUUGGCCAGCUCGU	dme-miR-9373-3p
miR-100	13	24	tpa-miR-100	AACCCGUAGAUUCGAAUUUGUGUU	asu-miR-100b-5p
miR-3767	12	21	tpa-miR-3767	UACACAUUAUUUACUACUACU	ame-miR-3767
miR-3049	11	22	tpa-miR-3049	UCCGUCCAACUCCUUUCCGUCU	ame-miR-3049-3p
miR-6382	11	24	tpa-miR-6382	UGGAAUGUAAAGAGAGCACACAAG	mmu-miR-6382
miR-998	10	21	tpa-miR-998	UAGCACCAUGGAAUUCAGCUG	api-miR-998
miR-6493	3	24	tpa-miR-6493-5p	ACGUCCGGCAGGUUUUACCCCU	mja-miR-6493-5p
	9	22	tpa-miR-6493-3p	AGGGGGAAACCGCGCUGAGCGUUA	mja-miR-6493-3p
miR-9198	9	23	tpa-miR-9198	CUUGGCACUGUCAGUGGAUGUGA	efu-miR-9198a
miR-2379	7	22	tpa-miR-2379	AGGCUGCUGGAGAAGAUAUUUU	bta-miR-2379
miR-1000	21	22	tpa-miR-1000a	AUAUUGUCCUGUCACAGCAGUA	api-miR-1000
	14	21	tpa-miR-1000b	AUAUUGUCCUGUCACAGCAGU	bmo-miR-1000
miR-3086	6	22	tpa-miR-3086	CCCAAUGAGCCUACAGUCUAAG	mmu-miR-3086-3p
miR-71	5	25	tpa-miR-71	UGAAAGACCUGUUGGUAGUGAGACG	ppc-miR-71a
miR-2944	5	23	tpa-miR-2944	UAUCACAGCCGUAGUUGCCUUAC	tca-miR-2944b-3p
miR-4724	5	21	tpa-miR-4724	GUACCUUCUGGUUCAGCUAGU	hsa-miR-4724-3p
miR-7475	5	20	tpa-miR-7475	CCGCCGCCGCCGCGCCCUCC	gga-miR-7475-5p
miR-8316	5	19	tpa-miR-8316	AUGGUGUCCAGGUCGUCGC	ppc-miR-8316-3p
miR-412	4	20	tpa-miR-412a	UGGUCGACCAGCUGGAAAGU	rno-miR-412-5p
	7	23	tpa-miR-412b	UGGUCGACCAGCUGGAAAGUAAU	cgr-miR-412-5p
miR-1726	4	22	tpa-miR-1726	AAGCUUGUUGGGUUUGGUUUGU	gga-miR-1726
miR-965	4	22	tpa-miR-965	UAAGCGUAUAGCUUUUCCCCUU	hme-miR-965
miR-6787	4	22	tpa-miR-6787	UGGCGGGGGUAGAGCUGGCUGC	hsa-miR-6787-5p
miR-454	3	22	tpa-miR-454	ACCCUAUCAAUAUUGUCUCUGC	hsa-miR-454-5p
miR-1723	3	23	tpa-miR-1723	UGGGAGCGGAAUGUGCAGCCUCA	gga-miR-1723
miR-786	3	23	tpa-miR-786	UAAUGCCCUUGCUGAGAUUCCAU	crm-miR-786
miR-3344	3	25	tpa-miR-3344	UUGCAAGAAGGACUCAGCCAGCGAG	bmo-miR-3344
miR-4045	3	22	tpa-miR-4045	CCACAAUGAAAGUAGAUGUCCG	cin-miR-4045-5p
miR-3878	3	19	tpa-miR-3878	UGGACGGAGAACUAAUUGU	tca-miR-3878-5p
miR-4459	3	22	tpa-miR-4459	CCAGGAGGCGGAGGAGGUGGAG	hsa-miR-4459
miR-4638	3	23	tpa-miR-4638	CCUGGACACCGCUCAGCCGGCCG	hsa-miR-4638-3p
miR-5625	3	21	tpa-miR-5625	CCCGGAAGUUCUUGAGUAGGA	mmu-miR-5625-5p
miR-7258	3	20	tpa-miR-7258	AAAAGGACUUGACUGCAGCA	mdo-miR-7258-5p
miR-7690	3	23	tpa-miR-7690	AAUCAUCCGGGAGUUGGGAAAGA	cbn-miR-7690
miR-15	3	23	tpa-miR-15	CGUAGCAGCACGUCAUGGUUUGU	ssa-miR-15a-5p
miR-8511	3	18	tpa-miR-8511	UCAGUCUUUUCCUCUUUC	pxy-miR-8511

**Table 3:** Homology analysis of *T*. *palmi* miRNA homologs.

tpa-miR	Insects	Other Arthropods	Other Invertbrates	Vertebrates	Note
tpa-miR-10	√	√	√	√	Highly conserved
tpa-miR-100	√	√	√	√	Highly conserved
tpa-miR-1000	√	—	—	—	Insect specific
tpa-miR-1175	√	—	√	—	Invertebrate specific
tpa-miR-2796	√	—	—	—	Insect specific
tpa-miR-279	√	√	√	—	Invertebrate specific
tpa-miR-71	√	√	√	√	Highly conserved
tpa-miR-750	√	√	√	—	Invertebrate specific
tpa-miR-965	√	√	—	—	Arthropod specific
tpa-miR-993	√	√	√	—	Invertebrate specific
tpa-miR-998	√	—	—	—	Insect specific
tpa-miR-9	√	√	√	√	Highly conserved
tpa-miR-2779	√	—	—	—	Insect specific
tpa-miR-92	√	√	√	√	Highly conserved
tpa-miR-15	—	—	√	√	Highly conserved
tpa-miR-1723	—	—	—	√	Vertebrate specific
tpa-miR-1726	—	—	—	√	Vertebrate specific
tpa-miR-2379	—	—	—	√	Vertebrate specific
tpa-miR-2478	—	—	—	√	Vertebrate specific
tpa-miR-2944	√		—	—	Insect specific
tpa-miR-3049	√	—	—	—	Insect specific
tpa-miR-3086	—	—	—	√	Vertebrate specific
tpa-miR-3344	√	—	—	—	Insect specific
tpa-miR-3767	√	—	—	—	Insect specific
tpa-miR-3878	√	—	—	—	Insect specific
tpa-miR-3931	—	√	—	—	Arthropod specific
tpa-miR-4045	—	—	—	√	Vertebrate specific
tpa-miR-412	—	—	—	√	Vertebrate specific
tpa-miR-4459	—	—	—	√	Vertebrate specific
tpa-miR-454	—	—	—	√	Vertebrate specific
tpa-miR-4638	—	—	—	√	Vertebrate specific
tpa-miR-4724	—	—	—	√	Vertebrate specific
tpa-miR-5108	—	—	—	√	Vertebrate specific
tpa-miR-5119	—	—	—	√	Vertebrate specific
tpa-miR-5124	—	—	—	√	Vertebrate specific
tpa-miR-5625	—	—	—	√	Vertebrate specific
tpa-miR-6240	—	—	—	√	Vertebrate specific
tpa-miR-6382	—	—	—	√	Vertebrate specific
tpa-miR-6489	—	√	—	—	Arthropod specific
tpa-miR-6493	—	√	—	—	Arthropod specific
tpa-miR-6787	—	—	—	√	Vertebrate specific
tpa-miR-6990	—	—	—	√	Vertebrate specific
tpa-miR-7156	—	—	—	√	Vertebrate specific
tpa-miR-7258	—	—	—	√	Vertebrate specific
tpa-miR-7475	—	—	—	√	Vertebrate specific
tpa-miR-7550	—	—	—	√	Vertebrate specific
tpa-miR-7690	—	—	√	—	Invertebrate specific
tpa-miR-786	—	—	√	—	Invertebrate specific
tpa-miR-800	—	—	√	—	Invertebrate specific
tpa-miR-8316	—	—	√	—	Invertebrate specific
tpa-miR-8511	√	—	—	—	Insect specific
tpa-miR-9198	—	—	—	√	Vertebrate specific
tpa-miR-9373	√	—	—	—	Insect specific
tpa-miR-281	√	√	√	√	Highly conserved

### Identification of miRNA-star strands

**Fig 2:**
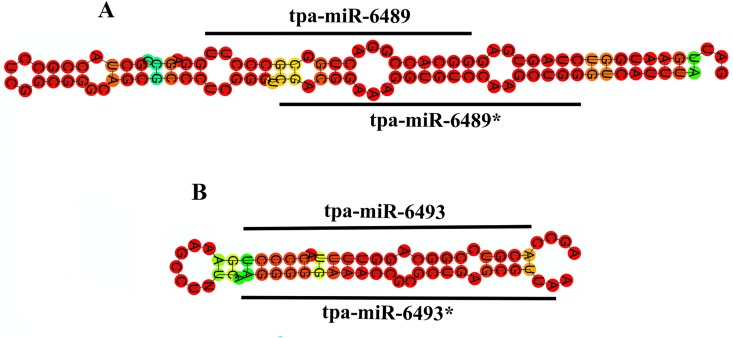
Stem loop structures of two miRNAs and their star strands. **(A)** The secondary structure of tpa-miR-6489 and tpa-miR-6489*. **(B)** The secondary structure of tpa-miR-6493 and tpa-miR-6493*. Both miRNAs and its star reads were marked by black bars. The secondary structure was predicted by employing RNA fold WebServer (www.rna.tbi.univie.ac.at/lgi-bin/RNAfold.cgi).

In most of the cases, once the mature miRNA strand is loaded into RISC, its star strand will be degraded soon after being exported to the cytosol. However, our analysis revealed that two *T*. *palmi* miRNA star (miRNA*) strands namely, miR-6489* and miR-6493* were obtained from our library for their corresponding mature miRNAs (Fig 2). The expression values (Number of reads) of miR-6489* were lower than that of their corresponding miRNAs, whereas, for miR-6493* it was three times higher (Table 2).

### Identification of novel miRNAs

**Fig 3:**
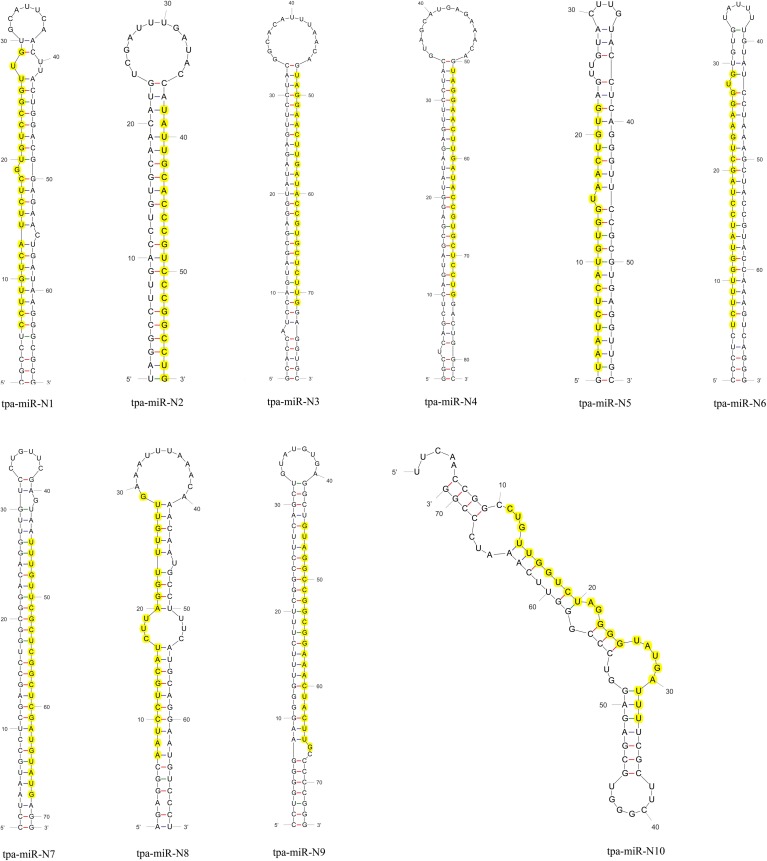
Various hairpin secondary structures of the ten novel pre-miRNAs of *T*. *palmi*. The mature miRNAs are indicated by yellow shades. The secondary structure was predicted using RNA fold WebServer.

We utilized the genomic sequence assembly of *F*. *occidentalis* (Thripidae: Thysanoptera) to identify the novel miRNAs, as no genomic information is available for *T*. *palmi*. Miranalyzer pipeline identified a total of 10 novel miRNAs from *T*. *palmi* for the first time (Table 4), with their predicted precursor secondary structures (Fig 3). The complete details of the mature miRNAs and their corresponding pre-miRNAs have been given in Table 4. The length of the novel miRNAs ranged from 21–24 nucleotides with a preference of Uracil (60%) followed by Cytosine (20%) at the 5' end. Among these ten miRNAs, five were located in the 5' arm while the other five arose from 3' arm (Table 4, Fig 3).

**Table 4:** Details of *T*. *palmi* novel miRNAs obtained from the current study. Information regarding mature and precursor sequences, start and end position, orientation, expression values, MFE value and (A+U) content were given.

miRNAs	Sequence	Locus	Scaffold	start	end	Hairpin Sequence	Start	End	Orientation	Abundance	miRNA length	MFE	Hairpin (A+U)%
tpa-miR-N1	CCUUGUCAUUCUCGUGUCCGGUUG	KL024066	Scaffold373	148612	148635	CGCCUCCUUGUCAUUCUCGUGUCCGGUUGUGCAUUCAACUUACUGGACGGAGAACUGAUAAGGGCGCG	554	577	Negative	10	24	-33.5	45.59
tpa-miR-N2	UAUUGCACCCGUCCCGGCCUG	KL023851	Scaffold158	512267	512247	UAGGCCUUGACCUGUGCAACAUGUCGAUUUGAUACCAUAUUGCACCCGUCCCGGCCUG	1724	1744	Negative	7	21	-20.8	46.55
tpa-miR-N3	UAGGAACUUGAUACCGUGCUCUUG	KL023856	Scaffold163	605332	605309	GCACCAUCCAGUAGCGAGGUAUAGAGUUCCUACGGCACAUUUAACAGUAGGAACUUGAUACCGUGCUCUUGGAGGUGC	2469	2492	Negative	4007	24	-41.22	50
tpa-miR-N4	UAGGAACUUGAUACCGUGCUCCUG	KL023856	Scaffold163	531005	531028	GGCUCAGCUCAGUAGCGAGGUAUAGAGUUCCUACGUAGCAUGAGAAACAGUAGGAACUUGAUACCGUGCUCCUGGACUGGCC	4132	4155	Positive	1026	24	-40.8	47.56
tpa-miR-N5	UAAUCUCAUGUGGUAACUGUG	KL023731	Scaffold38	137500	137520	GUAAUCUCAUGUGGUAACUGUGAGUUGUACUUGUACCUCAGGGUUCCGCGUGAGGUUGC	5735	5755	Positive	21	21	-31.3	50.85
tpa-miR-N6	UCUUUGGUAUCCUAGCUGAAGGUG	KL023728	Scaffold35	1566837	1566860	CCCUCUCUUUGGUAUCCUAGCUGAAGGUGUGUGUAUUUUGUAUCCUAAAGCUACCGUACCAAAGUCAGGG	13396	13419	Positive	13	24	-26.8	54.29
tpa-miR-N7	UUUGUUCGCUCGGCUCGAUGUAUG	KL023727	Scaffold34	1931509	1931532	CCUAAUGCCUCGAGCCUGGCGGACAGGUUGUCCUGUUCGAGUAAUUUGUUCGCUCGGCUCGAUGUAUGAGG	1925	1948	Positive	380	24	-37.9	45.07
tpa-miR-N8	AAUCCUGCAUCUUAGGUUUGUUG	KL023694	Scaffold1	2310295	2310273	AGAGGCAAUCCUGCAUCUUAGGUUUGUUGAAAUUUAAACAAACAAUGCCUUUCAUGCAGGAAUGUCCCU	5827	5849	Negative	9	23	-25	60.87
tpa-miR-N9	GUAGGCCGGCGGAAACUACUUG	KL023695	Scaffold2	1399443	1399464	CCUGGGGAAGGGGUUUCUUUCGGCCUUCAGCUGUAUGUGAGGCUGUAGGCCGGCGGAAACUACUUGCCCCCGGG	2544	2565	Positive	9	22	-44.6	37.84
tpa-miR-N10	CUGUUGGUCUAGGGGUAUGAUUU	KL023751	Scaffold58	1266	1245	UUCAACCGGCCUGUUGGUCUAGGGGUAUGAUUUUCGCUUCGGGUGCGAGAGGUCCCGGGUUCAAAUCCCGG	1197	1281	Positive	37	23	-21.5	42.3

### Abundance of novel miRNAs

The novel miRNAs identified from *T*. *palmi* varied in their expression values in the library. Among the novel miRNAs, tpa-miR-N3 (4007 copies), tpa-miR-N4 (1026 copies) and tpa-miR-N7 (380 copies) had the highest abundance compared to the remaining novel miRNAs (Table 4). Whereas, few other novel miRNAs namely, tpa-miR-N1, tpa-miR-N2, tpa-miR-N6, tpa-miR-N8 and tpa-miR-N9 were found to be very minimal (≤ 15 copies). The length and Minimum Free Energy (MFE) for these novel pre-miRNAs ranged from 58–82 nts and -20.8 to -44.6 kcal/mol respectively. The (A+U) % of the novel pre-miRNAs was in the range of 37.84% to 60.87% (Table 4).

### Sequence and phylogenetic analysis

**Fig 4:**
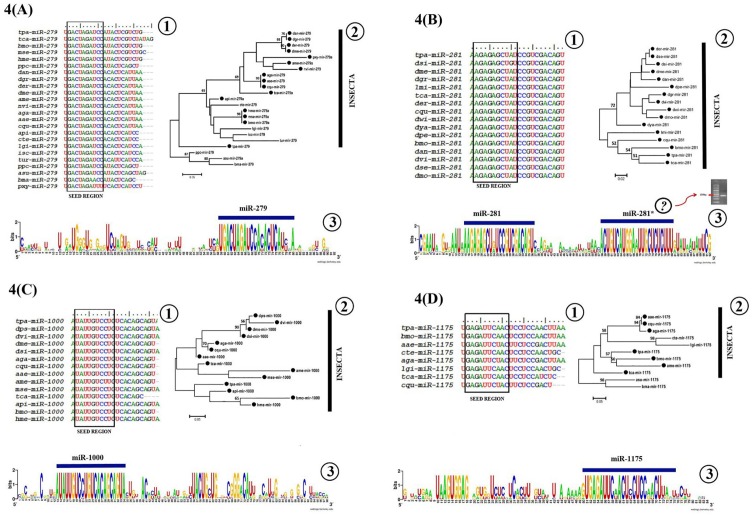
**(A) to (D). Homology, phylogeny and weblogo analysis of *T*. *palmi* miRNAs**. **4(A) to (D) 1.** Homology in the seed region of the *T*. *palmi* miRNA with respect to its counterpart from other insect species. Sequence conservation of the *T*. *palmi* mature miRNAs including the seed region over a wide range of insects. The first three letters of each miRNAs indicating the name of the species. **4(A) to (D) 2.** Phylogenetic trees (ML tree, RaxML.v.7.0.4) of four families of precursor miRNA sequences from various members of the animal kingdom. **4(A) to (D) 3**. *T*. *palmi* pre-miRNAs weblogo. The pre-miRNA sequence logos for the *T*. *palmi*, in which mature miRNA is indicated by blue bars. Each logo consists of stacks of symbols, one for each nucleotide position in the sequence. The height indicates the sequence conservation at that nucleotide position and the height of symbols within the stack indicates the relative frequency of each nucleotide at that position. Fig 4(B) 3 indicated the possible presence of miR-281* which was not evident from the NGS raw reads. However, the presence of miR-281* has been validated by stem-loop RT-PCR.

Sequence and phylogenetic analyses revealed that some of the known miRNAs were expressed in a wide range of insect species and are highly conserved (Table 3, Fig 4A1–4D1 and Fig 4A3–4D3). Mature miRNAs are highly conserved among various species within the Kingdom and are considered to be the evolutionarily conserved regulators of the gene expression [48]. The phylogenetic trees for miR-1000, miR-1175, miR-281 and miR-279 revealed that *T*. *palmi* miRNAs grouped with the closely related species of insects (Fig 4A2–4D2). However, few miRNAs (miR-1000, miR-2796, miR-965, miR-998, miR-2779, etc.) are highly specific to few species (Table 3). Fig 4A–4D revealed that *T*. *palmi* miRNAs are well conserved, particularly in the seed region compared to the homologous miRNAs from other species.

### Target Prediction

Targets were predicted for known and novel miRNAs of *T*. *palmi* employing miRanda on a scale of 0–7 to indicate the stringency of miRNA-target pairing with the smaller numbers representing higher stringency. ESTs and transcriptome of *F*. *occidentalis* were used as a reference for target searches with a cut-off score 140.

#### Targets for known miRNAs

**Fig 5:**
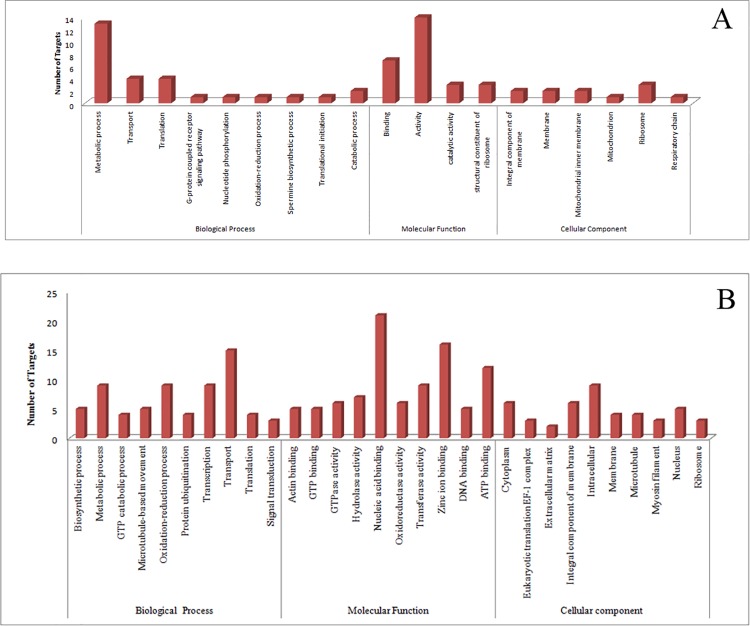
**Gene Ontology (GO) classification of the putative target genes for the *T*. *palmi* miRNAs against ESTs (A) and transcriptome (B) sequences of *F*. *occidentalis*.** GO terms were assigned to each target gene based on the annotation and were summarized into three main GO categories viz. (i) biological process (BP) (ii) molecular function (MF) and (iii) cellular component (CC). Only top ten subcategories are presented in the case of GO for transcriptome sequences.

All 67 known miRNAs were searched for targets against ESTs and transcriptome sequences of *F*. *occidentalis*. Out of the known 67 miRNAs, 20 and 40 known miRNAs were found to have targets in ESTs and transcriptome respectively (S2 and S3 Tables). The enrichment analysis (Blast-2-GO) was performed employing gene ontology (GO) terms for genes targeted by miRNAs (Fig 5A and 5B). For those targets in the ESTs, three motifs were over-represented in GO-BP (biological process) category like ‘metabolic process’, ‘transport’ and ‘translation’. The GO-MF (molecular function) category was over-represented by the motif ‘activity’ and ‘binding’ (Fig 5A). On the other hand, GO-terms enrichment analysis of miRNA targets in the transcriptome yielded motifs for ‘transport’, ‘metabolic process’ and ‘oxidation-reduction process’ in GO-BP category; while, GO-MF category was over-represented with motifs for ‘nucleic acid binding’, ‘zinc-ion binding’ and ‘ATP binding’ (Fig 5B). Complete details of the Blast-2-GO analysis have been provided in S4 and S5 Tables.

#### Targets for novel miRNA

Ten novel miRNAs were searched for their targets in the *F*. *occidentalis* transcriptome. A total of 33 miRNA-target pairs were obtained (S6 Table) and further Blast-2-GO analysis yielded ‘regulation of transcription’ and ‘binding’ as GO-BP and GO-MF category respectively (S7 Table). Complete details of the miRNA targets and Blast-2-GO analysis have been provided in S7 Table.

**Fig 6:**
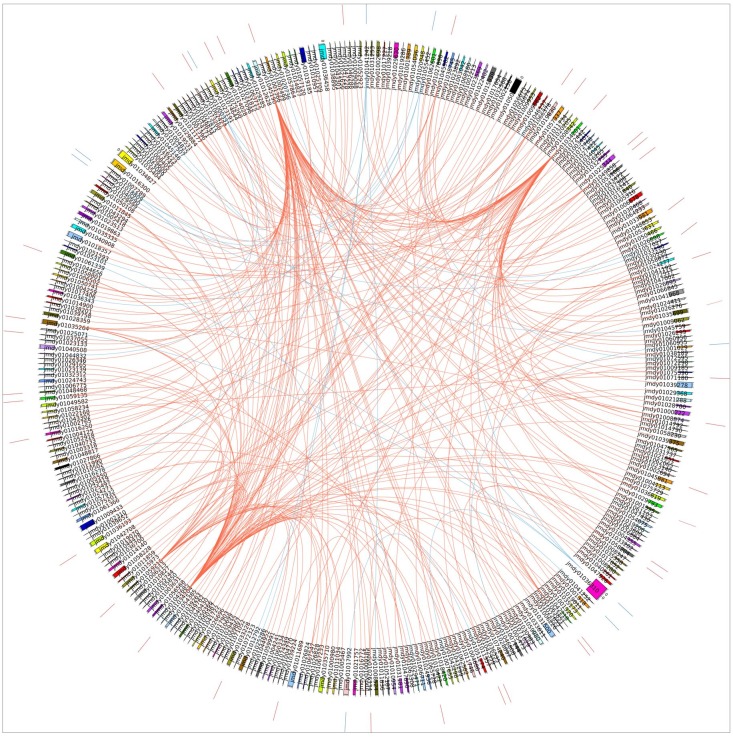
The synteny analyses using Circos (Krzywinski et al. 2009). Map of the Western Flower Thrips, *F*. *occidentalis* scaffolds linking *T*. *palmi* miRNAs and their targets prepared using Circos. The outer circle represents the highlights of 10 novel miRNA in blue and 40 known miRNA represented in red colour. The inner lines in red colour represent known miRNAs and their targets (839 targets) and blue lines represent 11 novel miRNAs and their targets (33 targets) across 300 scaffolds of *F*. *occidentalis* genome.

The synteny analysis of the *T*. *palmi* miRNAs and their targets were performed by employing circos^44^. In brief, the Blast analysis was performed using *T*. *palmi* miRNA sequences (known and novel) against *F*. *occidentalis* scaffolds (Largest 300). The positions of miRNAs were identified and their targets represented in the Circos plot (Fig 6).

### Validation of *T*. *palmi* microRNAs

**Fig 7:**
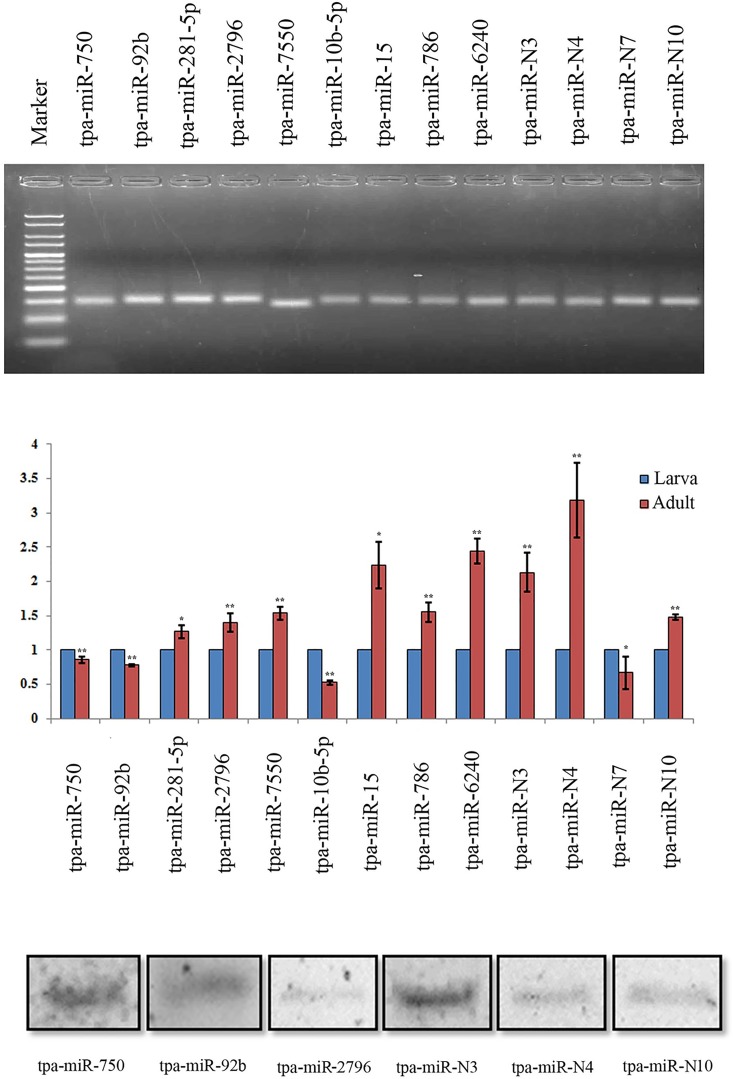
Validation of selected conserved and novel miRNAs from *T*. *palmi*. (**A**) Stem-loop RT-PCR analyses of nine conserved and four novel miRNAs from *T*. *palmi*. The products were resolved on 3% agarose gel in 1X TBE stained with ethidium bromide. HyperLadder™ 25bp (Bioline, USA) employed as a marker. (**B**) Stem-loop RT-qPCR analysis of spatiotemporally expressed *T*. *palmi* miRNAs in larva and adults. ‘*’ and ‘**’ means a statistically significant difference at level p < 0.05 and p < 0.001 respectively for these miRNAs in the larva and adult *T*. *palmi*. The error bars indicate standard deviation for three biological replications. (**C**) Small RNA Northern blot validation. Both conserved (tpa-miR-750, tpa-miR-92b, tpa-miR-2796) and novel (tpa-miR-N3, tpa-miR-N4 and tpa-miR-N10) miRNAs were validated by small RNA northern analysis employing small RNA isolated from pooled *T*. *palmi*.

The present study revealed the novel miRNAs from *T*. *palmi* (Tables 2 and 4). However, further validation of these miRNAs was performed by (i) stem-loop end-point reverse transcriptase PCR (RT-PCR) (ii) real-time quantitative reverse transcriptase PCR (qRT-PCR) and (iii) small RNA Northern blots. Using stem-loop end-point RT-PCR, we have validated 9 conserved (tpa-miR-750, tpa-miR-92b, tpa-miR-281-5p, tpa-miR-2796, tpa-miR-10b-5p, tpa-miR-786, tpa-miR-6240, tpa-miR-7550 and tpa-mir-15) and 4 novel miRNAs (tpa-miR-N3, tpa-miR-N4, tpa-miR-N7, tpa-miR-N10) from *T*. *palmi* using the primer sets as described (Table 5). All of these miRNAs were amplified with an approximate product size of 75 bp (Fig 7A).

**Table 5:** List of Universal Reverse primer, Stem-loop RT primers and forward primers employed in small RNA validation.

Sl. No.	Oligo Name	Oligo Sequence (5' to 3')
1	Universal Reverse	ATCCAGTGCAGGGTCCGAGG
2	RT/tpa-miR-750	GTCGTATCCAGTGCAGGGTCCGAGGTATTCGCACTGGATACGACGAGCTG
3	F/tpa-miR-750	GCGGCGGCCAGATCTAACTCTTC
4	RT/tpa-miR-92b	GTCGTATCCAGTGCAGGGTCCGAGGTATTCGCACTGGATACGACTCAGGC
5	F/tpa-miR-92b	GCGGCGGAATTGCACCCGTCCC
6	RT/tpa-miR-281-5p	GTCGTATCCAGTGCAGGGTCCGAGGTATTCGCACTGGATACGACACTGTC
7	F/tpa-miR-281-5p	GCGGCGGAAGAGAGCTATCCGTC
8	RT/tpa-miR-2796	GTCGTATCCAGTGCAGGGTCCGAGGTATTCGCACTGGATACGACGCAAGT
9	F/tpa-miR-2796	GCGGCGGGTAGGCCGGCGGAAAC
10	RT/tpa-miR-7550	GTCGTATCCAGTGCAGGGTCCGAGGTATTCGCACTGGATACGACTGGTCC
11	F/tpa-miR-7550	GCGGCGGATCCGGCTCGAAGGAC
12	RT/tpa-miR-10b-5p	GTCGTATCCAGTGCAGGGTCCGAGGTATTCGCACTGGATACGACTCAAAT
13	F/tpa-miR-10b-5p	GCGGCGGAACCCTGTAGACCCG
14	RT/tpa-miR-15	GTCGTATCCAGTGCAGGGTCCGAGGTATTCGCACTGGATACGACACAAAC
15	F/tpa-miR-15	GCGGCGGCGTAGCAGCACGTCATG
16	RT/tpa-miR-8511	GTCGTATCCAGTGCAGGGTCCGAGGTATTCGCACTGGATACGACGAAAGA
17	F/tpa-miR-8511	GCGGCGGTCAGTCTTTTCCTC
18	RT/tpa-miR-6240	GTCGTATCCAGTGCAGGGTCCGAGGTATTCGCACTGGATACGACCGCCGT
19	F/tpa-miR-6240	GCGGCGGCCAAAGCATCGCGAAG
20	RT/tpa-miR-N3	GTCGTATCCAGTGCAGGGTCCGAGGTATTCGCACTGGATACGACCAAGAG
21	F/tpa-miR-N3	GCGGCGGTAGGAACTTGATACCG
22	RT/tpa-miR-N4	GTCGTATCCAGTGCAGGGTCCGAGGTATTCGCACTGGATACGACCAGGAG
23	F/tpa-miR-N4	GCGGCGGTAGGAACTTGATACCG
24	RT/tpa-miR-N7	GTCGTATCCAGTGCAGGGTCCGAGGTATTCGCACTGGATACGACCATACA
25	F/tpa-miR-N7	GCGGCGGTTTGTTCGCTCGGCTC
26	RT/tpa-miR-N10	GTCGTATCCAGTGCAGGGTCCGAGGTATTCGCACTGGATACGACAAATCA
27	F/tpa-miR-N10	GCGGCGGCTGTTGGTCTAGGGG
28	RT/tpa-miR-281*	GTCGTATCCAGTGCAGGGTCCGAGGTATTCGCACTGGATACGACAAAGAG
29	F/tpa-miR-281*	GCGGCGGACUGUCAUGGAAUUGC

Our study also quantified the expression level of the above-mentioned 13 miRNAs from *T*. *palmi* larvae and adults using qRT-PCR (Table 6, Fig 7B). Results suggested that the miRNA expression was higher in larval stages compared to adults in four microRNAs namely *tpa*-miR-750, tpa-miR-92b, tpa-miR-10b-5p and tpa-miR-N7 (Fig 7B). Finally, we also validated 3 conserved (tpa-miR-750, tpa-miR-92b and tpa-miR-2796) and three novel miRNAs (tpa-miR-N3, tpa-miR-N4 and tpa-miR-N10) employing a more sensitive small RNA Northern blot technique (Fig 7C).

**Table 6:** MicroRNA specific primers employed in the RT-qPCR.

miRNA	Sequence (5' - 3')
tpa-miR-750 Forward	CCAGATCTAACTCTTCCAGCTC
tpa-miR-92b Forward	AATTGCACCCGTCCCGGCCTGA
tpa-miR-281-5p Forward	AAGAGAGCTATCCGTCGACAGT
tpa-miR-2796 Forward	GTAGGCCGGCGGAAACTACTTGC
tpa-miR-7550 Forward	ATCCGGCTCGAAGGACCA
tpa-miR-10b-5p Forward	AACCCTGTAGACCCGAATTTGA
tpa-miR-15 Forward	CGTAGCAGCACGTCATGGTTTGT
tpa-miR-8511 Forward	TCAGTCTTTTCCTCTTTC
tpa-miR-6240 Forward	CCAAAGCATCGCGAAGGCCCACGGCG
tpa-miR-N3 Forward	TAGGAACTTGATACCGTGCTCTTG
tpa-miR-N4 Forward	TAGGAACTTGATACCGTGCTCCTG
tpa-miR-N7 Forward	TTTGTTCGCTCGGCTCGATGTATG
tpa-miR-N10 Forward	CTGTTGGTCTAGGGGTATGATTT

## Discussion

### Illumina deep sequencing approach for identification of microRNAs

With the advent of the next generation sequencing technologies, miRNAs have been discovered at an accelerated pace. Presently miRNAs are known from more than 25 insect species, which includes 12 *Drosophila* species [49]. Among them, the most recent ones are from *Plutella xylostella* [47], *Spodoptera frugiperda* [50], etc. Several miRNAs have been reported from various orders of insects such as Diptera, Hymenoptera, Coleoptera, Orthoptera, Lepidoptera, Hemiptera and Homoptera [27], and for the first time, we report the small RNAs from a thysanopteran insect, *T*. *palmi*. In this regard, small RNA library was prepared from the pooled samples of different developmental stages of *T*. *palmi* and then Illumina (sequencing-by-synthesis) sequencing technology was used to identify miRNAs from the library. The Illumina sequencing approach is one of the high throughput technologies by which miRNAs of any organisms can be identified [51–60,24]. Size distributions of the high-quality reads varied from 18–28 nts in the library. The peak was at 25 nt which was at par with the previous studies [47,60,61]. According to Bartel (2004), the average miRNA length is 22 nt in animals and study conducted showed that the average length is 23 nt.

The unique read distributes of 26–28 nts with a relative lower abundance are common for many small RNA libraries [62–64] indicating the presence of piRNAs. Piwi RNAs (piRNAs) are the class of small RNAs mediating chromatin modifications [65] which are derived mainly from retro-transposons and other repetitive elements with high sequence diversity [62,65,66]. Thus, the results indicated that *T*. *palmi* genome encodes not only miRNAs but also other small RNAs such as piRNAs (S1 Table) in a lower proportion that might be involved in the trans-generational epigenetic inheritance [67].

### Homology-based predictions of miRNAs

The identification of small RNAs (especially miRNAs) based on genomic information has been reported previously in several insects [60,62]. In this regard, we report the identification and characterization of miRNAs from *T*. *palmi* based on Illumina small RNA sequencing. We employed *F*. *occidentalis* genome sequences as a reference for *T*. *palmi* since the complete genome for *T*. *palmi* is still not available. However, a large proportion (93.12%) of the *T*. *palmi* sRNA sequences could be mapped on to *F*. *occidentalis* genome. This higher percentage of mapping was possible because *T*. *palmi* & *F*. *occidentalis* (reference genome) belong to the same family, Thripidae. Mapping onto a whole genome sequence also helped in elucidating the sample proportion of small ncRNAs such as tRNA, rRNA, snoRNA or snRNA [68]. All of these sequences were annotated by aligning the reads with Rfam database, which indicated the efficiency of deep sequencing in identifying small RNAs. Our results indicated that there is a rich small RNA world evident in *T*. *palmi*.

Our study revealed 67 conserved and ten novel miRNAs from *T*. *palmi* for the first time. The (A+U) content of the pre-miRNAs should be in the range of 30–70% [69], as those with higher (A+U) content bind more strongly to proteins [20,70]. In this regard, the (A+U) content of the novel pre-miRNAs ranged from 37.84% (tpa-miR-N9) to 60.87% (tpa-miR-N8). In our dataset, the most abundant miRNAs were tpa-miR-281 (from the conserved miRNAs) and tpa-miR-N3 (from the novel miRNAs) with a total of 9560 and 4007 number of reads respectively.

miRNAs are known to be conserved among different species within a kingdom and are evolutionarily conserved regulators of gene expression [20,71]. Our homology and phylogenetic analysis revealed that insect miRNAs are known to be well-conserved, despite considerable diversity in the genome (Fig 4A–4D). In most of the cases, detection of miRNA*s is difficult with the available methods as these molecules are liable to degrade soon after being exported to the cytosol [27]. However, in our study during the process of identifying conserved miRNAs, two miRNAs for instance, miR-6489* and miR-6493* that matched to the same precursor sequences with their mismatched complementary mature miRNAs were also detected. The weblogo sequences analysis revealed the likely presence of miR-281* which was not identified in the raw reads of NGS. The presence of miR-281* was identified by BLASTN option in miRBase and further confirmed by stem-loop RT PCR (Table 5, Fig 4B3). The absence of miR-281* could be due to the faster degradation as compared to miR-281.

### Possible roles of *T*. *palmi* miRNAs

Although thousands of small RNAs have been discovered in the recent past, [39,40,47,50,72] the primary challenge is to fully identify the spatiotemporally expressed microRNAs and to determine their individual functions. The majority of the microRNAs have been identified through either computational prediction or cloning and sequencing [73]. In this study, we employed Illumina next generation sequencing approach to identify miRNAs from *T*. *palmi*. Currently, there are several mature and precursor microRNAs deposited in the miRBase [39]. In this connection, we identified a total of 77 miRNAs from *T*. *palmi* using high throughput sequencing. The current study is the first report of miRNA profiling from a *Thrips* species employing deep sequencing approach. This approach is far superior to the other approaches of miRNA identification, as it can discover novel microRNAs [74].

The analysis of the expression value (read numbers) revealed that the highest expression was for miR-281 (9560 reads). Recent studies have proved that microRNA-281 regulate the expression of *ecdysone receptor* (*EcR*) isoform B, in the silkworm, *Bombyx mori* [75]. Thus, miR-281 may be involved in development and metamorphosis of *T*. *palmi* by regulating the genes involved in the ecdysone cascade. The second highest expression was for miR-750 with an expression value of 7869. RNAi studies proved that the putative JH receptor *ultraspiracle* (*USP*) [76,77] is a likely target of miR-750. Thus, it indicates that miR-750 may be involved in hormone signaling, immunity and stress response by regulating the *vitellogenin* (*Vg*) gene in *T*. *palmi* [78].

Another interesting microRNA obtained in the current study was miR-92 with an expression value of 1687. Previous studies had shown that miR-92 regulates *Mef2*, the key transcription factor for muscle development and differentiation in *Drosophila* [79]. An insect-specific microRNA, miR-2796 was identified in *T*. *palmi* with an expression value of 479. miR-2796 was found to be the most abundant microRNA in honey bee brain [80]. Additionally, miR-2796 bound to the coding sequence (CDs) of *PLC-epsilon* gene in *Apis* and *Tribolium*, affecting the mRNA stability by splicing rather than the normal canonical translational repression [81]. However, interestingly both miR-2796 and *PLC-epsilon* gene were missing from the genus *Drosophila*, even though it was found in other dipterans [80].

Our analyses revealed miR-993 were identified only in invertebrates (Table 3). miR-993 belongs to the miR-100/10 family, and both miR-993 and miR-10 are derived from the ancient miR-100 through duplication and arm-switching [60,82]. miR100/10 family members could regulate the expression of relevant *Hox*-genes, thus may play a crucial role in insect development [83]. Rest of the insect-specific miRNAs identified in *T*. *palmi* may play an important role in insect-specific features, such as metamorphosis, parthenogenesis and biogenesis of pheromones [84]. Whereas, the other invertebrate and vertebrate-specific miRNAs (Table 3) identified from *T*. *palmi* required special attention, as their nonexistence in other species of insects could be due to the absence of genomic information for most of those insects [60]. These specific microRNAs may be involved in some special biological processes that distinguish thysanopteran insects from others.

### Developmental roles of *T*. *palmi* miRNAs

The expression profile of miRNA varies among different developmental stages [85,86]. In the present study, the developmental expression profiles (larval and adult stage) of microRNAs namely, miR-750, miR-92b, miR-281-5p, miR-2796, miR-7550, miR-10b-5p, miR-15, miR-786, miR-6240, tpa-miR-N3, tpa-miR-N4, tpa-miR-N7 and tpa-miR-N10 were investigated by qRT-PCR (Fig 7B). The higher expression of miR-750, miR-92b, miR-10b-5p and tpa-miR-N7 in *T*. *palmi* larvae reflected their possible involvement in insect-specific features such as metamorphosis, whereas, the high levels of miR-281-5p, miR-2796, miR-7550, miR-15, miR-786, miR-6240, tpa-miR-N3, tpa-miR-N4 and tpa-miR-N10 in the adult stage indicated their role in the adult development, parthenogenesis and sexual reproduction.

Target prediction is crucial to understand the biological role of a particular miRNA. Unlike their plant counterparts, the imperfect complementarity of animal miRNAs to their target mRNA sequences makes it more difficult to judge the accuracy of prediction. MicroRNAs can bring about mRNA cleavage or translational repression of target mRNAs by binding to 3' UTRs, 5' UTRs and even to coding regions [87]. However, animal miRNAs primarily target the 3' UTRs; and therefore, we limited our target search to (i) expressed sequence tags (ESTs) and (ii) transcriptomic sequences of *F*. *occidentalis*. The predicted targets were annotated against GO database and the targeted genes included transcription factors, signal transduction, hormone pathways, molting and even metabolism. Therefore, all these conserved and novel miRNAs identified from *T*. *palmi* could play a vital role in diverse biological processes, thus undoubtedly participating in the regulation of thrips growth and development.

In summary, results from this study add to our growing pool of miRNA database and is the first report on such analysis in a thysanopteran insect, *T*. *palmi*. Deep sequencing of small RNAs has facilitated the identification of miRNAs from *T*. *palmi*. Sixty-seven conserved and ten novel miRNAs that were identified with high confidence and sufficient evidence are the contributions from our study. Most of the *T*. *palmi* miRNAs were homologous to insects as compared to the vertebrates. Sequence and phylogenetic analyses revealed that most of the *T*. *palmi* miRNAs are highly conserved in various species, making miRNAs, a hallmark of evolutionarily conserved regulators of gene expression. To harmonize the data, and to provide more useful biological insights, we also carried out *in silico* analysis for identifying potential targets for these miRNAs. Unlike the plant counterparts, the imperfect complementarity of metazoan miRNAs to the target has been found to be sufficient to promote the RNA silencing, as in the case of *Drosophila* and *Bactrocera* [88]. Our results indicated that the list of putative mRNA targets was very extensive (S2, S3 and S6 Tables), even with stringent parameters applied to miRanda. Our results suggested that most of the putative target genes for *T*. *palmi* miRNAs were associated with several KEGG pathways like metabolic process, transport, translation, signal pathways and oxidative phosphorylation. However, further wet lab experiments are still required for the validation of these targets in understanding the biology of this insect. Expression levels of few miRNAs were also validated by both qRT-PCR and Northern analysis.

Several miRNAs were identified and characterized from animals and plants and among them, very few were further explored for various applications by disrupting specific pathways targeted by these miRNAs. This can be achieved by employing the artificial microRNAs (amiRs) [89]. Recent studies successfully demonstrated the use of amiRNAs for targeting the reporter and the endogenous genes in animals and plants. Identification and expression of a few essential insect-specific gene(s) in plants, can target and degrade an invading insect’s genes, consequently confer insect resistance [90]. Results from our study indicated few miRNAs have been predicted to be involved in the adult development process, which can be further utilized in gene functional studies through RNAi-based approach or in developing miRNA mimics both for feeding and *in planta* expression [29,88,91] as novel pest management strategies based on gene silencing and insect transgenesis.

## Supporting Information

S1 Table Small RNAs (Piwi RNAs) with nucleotide lengths larger than 25 nucleotides obtained from our sequencing data. (DOCX)

S2 Table Potential targets for the identified known miRNAs with EST orthologs of *F*. *occidentalis*. (DOC)

S3 Table Potential targets for the identified known miRNAs with the transcriptomic sequences of *F*. *occidentalis*. (DOC)

S4 Table Complete Functional categories of gene ontology classification of the putative target genes for the *T*. *palmi* miRNAs against ESTs of *F*. *occidentalis*. (DOC)

S5 Table Complete Functional categories of gene ontology classification of the putative target genes for the known miRNAs against transcriptome sequences of *F*. *occidentalis*. (DOC)

S6 Table Potential targets for the identified novel miRNAs with Transcriptome sequences of *F*. *occidentalis*. (DOC)

S7 Table Complete Functional categories of gene ontology classification of the putative target genes for the novel miRNAs against transcriptome sequences of *F*. *occidentalis*. (DOC)
