# Supplementary material for: The First Report of miRNAs from a Thysanopteran Insect, Thrips palmi Karny Using High-Throughput Sequencing
Source: PLoS One. 2016 Sep 29;11(9):e0163635. doi: 10.1371/journal.pone.0163635 (PMC5042526; doi:10.1371/journal.pone.0163635)
Supplement: S1 Table — (DOCX) [file pone.0163635.s001.docx]

| **smallRNA ID** | **Sequence (5' -> 3'** | **Length (nt)** | **Hit in the piRNABank** | **E-value** |
| --- | --- | --- | --- | --- |
| tpa_piR1 | CCAAAGCAUCGCGAAGGCCCACGGCG | 26 | dr_piR_0052831 | 0.0047 |
| tpa_piR2 | TTTCCGTAGTGTAGTGGTTATCACGTTC | 28 | rno_piR_005901 | 1.70E-05 |
| tpa_piR3 | CCTGTGGTCTAGTGGTTAGGATTCGGCG | 28 | ona_piR_166322 | 0.00049 |
| tpa_piR4 | CCCTCGGTTCTGGCGTCAAGCGGGCCG | 27 | No Hit | NA |
| tpa_piR5 | GGGTTCGATTCCCGGTCAGGGAACCA | 26 | dr_piR_0017650 | 0.1 |
| tpa_piR6 | TGAAAGACAACTCTTAGCGGTGGATC | 26 | No Hit | NA |
| tpa_piR7 | ATTGGTGGTTCAGTGGTAGAATTCTCGC | 28 | hsa_piR_001312 | 1.80E-05 |
| tpa_piR8 | ATTGTGGTTCAGTGGTAGAATTCTCGCC | 28 | hsa_piR_018570 | 0.00065 |

**Supplementary Table S1.** **Small RNAs (Piwi RNAs) with nucleotide lengths larger than 25 nucleotides obtained from our sequencing data**
