# Supplementary material for: The First Report of miRNAs from a Thysanopteran Insect, Thrips palmi Karny Using High-Throughput Sequencing
Source: PLoS One. 2016 Sep 29;11(9):e0163635. doi: 10.1371/journal.pone.0163635 (PMC5042526; doi:10.1371/journal.pone.0163635)
Supplement: S2 Table — (DOC) [file pone.0163635.s002.doc]

| **Supplementary Table S2. Potential targets for the identified known miRNAs with EST orthologs of *F. occidentalis.*** | | | | | | | | | | | | |
| --- | --- | --- | --- | --- | --- | --- | --- | --- | --- | --- | --- | --- |
| **miRNA** | **Target Id** | **Hit Acc.** | **Protein Name** | **Alignment score** | **Energy** | **miRNA_start** | **miRNA_end** | **Target_start** | **Target_end** | **Alignment length** | **miRNA_aligned_fragment** | **Target_aligned_fragment** |
| isc-miR-750 | gi|297511326|gb|GT303584.1|GT303584 | XP_001601396 | ubx domain-containing protein 4-like isoform 1 | 149 | -31.51 | 2 | 22 | 414 | 442 | 27 | Query: 3' acUCGACCUUCUC--A-----AUCUAGACc 5' | Ref: 5' ggAG-TGGAGGAGCATACCCGTAGATCTGg 3' |
| ame-miR-750 | gi|297511326|gb|GT303584.1|GT303584 | XP_001601396 | ubx domain-containing protein 4-like isoform 1 | 148 | -30.97 | 2 | 19 | 414 | 442 | 24 | Query: 3' cucgACCUUCUC--A-----AUCUAGACc 5' | Ref: 5' ggagTGGAGGAGCATACCCGTAGATCTGg 3' |
| cte-miR-750 | gi|297511326|gb|GT303584.1|GT303584 | XP_001601396 | ubx domain-containing protein 4-like isoform 1 | 149 | -31.51 | 2 | 22 | 414 | 442 | 27 | Query: 3' acUCGACCUUCUC--A-----AUCUAGACc 5' | Ref: 5' ggAG-TGGAGGAGCATACCCGTAGATCTGg 3' |
| ame-miR-92b | gi|297513463|gb|GT299234.1|GT299234 | #N/A | #N/A | 167 | -31.84 | 2 | 21 | 285 | 307 | 20 | Query: 3' agUCCGGCCCUGCC-CACGUUAa 5' | Ref: 5' ggAGGGTGGGGGGGTGTGCAATt 3' |
| ame-miR-92b | gi|297508143|gb|GT311016.1|GT311016 | #N/A | #N/A | 168 | -31.2 | 2 | 19 | 1053 | 1073 | 17 | Query: 3' agucCGGCCCUGCCCACGUUAa 5' | Ref: 5' gacaGCTGGG-TGGGTGCAATa 3' |
| mmu-miR-6240 | gi|297510447|gb|GT308789.1|GT308789 | XP_002430216 | eukaryotic translation initiation factor 3 | 169 | -30.24 | 2 | 25 | 554 | 579 | 24 | Query: 3' gcGGC-ACCCGGAAGCGCUACGAAACc 5' | Ref: 5' aaCCGATTGCCCTTC-AGGTGCTTTGg 3' |
| mmu-miR-6240 | gi|297508254|gb|GT307760.1|GT307760 | #N/A | #N/A | 169 | -35.37 | 2 | 25 | 18 | 48 | 28 | Query: 3' gcGGCACCCG-GA-AGCGC---UACGAAACc 5' | Ref: 5' ggCCCTGGGCTCTGTTGTGTACATGCTTTGg 3' |
| mmu-miR-6240 | gi|297506038|gb|GT309485.1|GT309485 | XP_001948759 | protein downstream neighbor of son homolog | 179 | -37.13 | 2 | 25 | 401 | 427 | 24 | Query: 3' gcGGCACCCG-GAAGCGCUACGAAACc 5' | Ref: 5' tcCAGTCCGCACTTCGTGGTGCTTTGg 3' |
| mmu-miR-6240 | gi|297504585|gb|GT303009.1|GT303009 | #N/A | #N/A | 159 | -30.34 | 2 | 25 | 69 | 95 | 24 | Query: 3' gcGGCACCCG-GAAGCGCUACGAAACc 5' | Ref: 5' gcCCGTATGTAGGTCATGGTGCTTTGg 3' |
| tca-miR-750-3p | gi|297511326|gb|GT303584.1|GT303584 | XP_001601396 | ubx domain-containing protein 4-like isoform 1 | 149 | -30.53 | 2 | 20 | 411 | 442 | 25 | Query: 3' gcaguaUACCUUCUC--A-----AUCUAGACc 5' | Ref: 5' ggcggaGTGGAGGAGCATACCCGTAGATCTGg 3' |
| mmu-miR-6990-5p | gi|297511413|gb|GT311562.1|GT311562 | XP_003242244 | mannose-1-phosphate guanyltransferase alpha-a-like isoform 2 | 170 | -31.92 | 2 | 20 | 656 | 677 | 19 | Query: 3' ucUCGGGACU-GAGUGGGACCc 5' | Ref: 5' acAGCTCCGAGCTTACCCTGGg 3' |
| mmu-miR-6990-5p | gi|297503792|gb|GT302478.1|GT302478 | EFR20539 | spermine synthase | 169 | -32.56 | 2 | 18 | 660 | 680 | 16 | Query: 3' ucucGGGACUGAGUGGGACCc 5' | Ref: 5' ttttCCTTGTCTTACCCTGGg 3' |
| mja-miR-6489-5p | gi|297502033|gb|GT311286.1|GT311286 | #N/A | #N/A | 161 | -31.67 | 2 | 19 | 1 | 21 | 18 | Query: 3' uuCCCGCGGUCA-GGCCACGg 5' | Ref: 5' ggGGACTCTAGTGCCGGTGCc 3' |
| ame-miR-3049-3p | gi|297502893|gb|GT299023.1|GT299023 | #N/A | #N/A | 169 | -31.04 | 2 | 18 | 54 | 75 | 16 | Query: 3' ucugcCUUUCCUCAACCUGCCu 5' | Ref: 5' agtgaGCGAGGGGTTGGACGGa 3' |
| mja-miR-6493-3p | gi|297512918|gb|GT311924.1|GT311924 | #N/A | #N/A | 156 | -30.51 | 2 | 23 | 293 | 320 | 25 | Query: 3' auUGCG--AGUCGCGCC--AAAGGGGGa 5' | Ref: 5' aaACGCAAACGGGGGGGTTTTTCCCCCc 3' |
| mja-miR-6493-3p | gi|297512455|gb|GT306408.1|GT306408 | #N/A | #N/A | 169 | -30.03 | 2 | 22 | 660 | 683 | 20 | Query: 3' auuGCGAGUCGCGCCAAAGGGGGa 5' | Ref: 5' ttcTGCTAAGTGTGAATTCCCCCt 3' |
| mja-miR-6493-3p | gi|297511074|gb|GT303418.1|GT303418 | #N/A | #N/A | 159 | -30.55 | 2 | 17 | 574 | 598 | 16 | Query: 3' auugcgagUCGCGCC-AAAGGGGGa 5' | Ref: 5' ggaccaaaAGCGTGGCATTCCCCCt 3' |
| mja-miR-6493-3p | gi|297510776|gb|GT311165.1|GT311165 | #N/A | #N/A | 159 | -32.05 | 2 | 22 | 25 | 52 | 24 | Query: 3' auuGCGAGUCGCGC-CA---AAGGGGGa 5' | Ref: 5' cctCGAGCAGCGTGTGTCTCTTCCCCCg 3' |
| mja-miR-6493-3p | gi|297509064|gb|GT302976.1|GT302976 | EOR03886 | b-cell receptor-associated 31-like protein | 170 | -30.11 | 2 | 23 | 363 | 384 | 21 | Query: 3' auUGCGAGUCGCGCCAAAGGGGGa 5' | Ref: 5' ttATCCTCA-C-CGGTTTCCCCCc 3' |
| mja-miR-6493-3p | gi|297506013|gb|GT309460.1|GT309460 | #N/A | #N/A | 168 | -30.75 | 2 | 20 | 332 | 355 | 19 | Query: 3' auugcGAGUCGCGCCA-AAGGGGGa 5' | Ref: 5' cacttTTCAGCG-GGTGTTCCCCCc 3' |
| mja-miR-6493-3p | gi|297501756|gb|GT303185.1|GT303185 | #N/A | #N/A | 173 | -35.46 | 2 | 23 | 442 | 466 | 22 | Query: 3' auUGCGAGUCGCG-CCAAAGGGGGa 5' | Ref: 5' caATGACTAGTGCTGGTTTCCCCCc 3' |
| efu-miR-9198a | gi|297510657|gb|GT303130.1|GT303130 | ACT32993 | beta-ketoacyl- thiolase | 172 | -32.76 | 2 | 22 | 247 | 270 | 21 | Query: 3' agUGUAGGUGACUG-UCACGGUUc 5' | Ref: 5' acACATACATTGGCTTGTGCCAAg 3' |
| efu-miR-9198a | gi|297503348|gb|GT311517.1|GT311517 | ABV60291 | trypsin-1- partial | 165 | -30.14 | 2 | 22 | 572 | 594 | 20 | Query: 3' agUGUAGGUGACUGUCACGGUUc 5' | Ref: 5' tgACACAAGATGACGGTGCCAAg 3' |
| mja-miR-6489-3p | gi|297503422|gb|GT303650.1|GT303650 | #N/A | #N/A | 177 | -32.15 | 2 | 22 | 127 | 149 | 20 | Query: 3' ggUCGAACCUGUGGAAAGGCAGc 5' | Ref: 5' tcGGCTGGGCTACTTTTCCGTCc 3' |
| tca-miR-2944b-3p | gi|297508223|gb|GT307729.1|GT307729 | ENN80902 | cad isoform 2 | 169 | -30.34 | 2 | 22 | 461 | 483 | 20 | Query: 3' caUUCCGUUGAUGCCGACACUAu 5' | Ref: 5' taGAGGATTCTGTGGCTGTGATg 3' |
| tca-miR-2944b-3p | gi|297503890|gb|GT302532.1|GT302532 | #N/A | #N/A | 185 | -34.17 | 2 | 22 | 597 | 619 | 20 | Query: 3' caUUCCGUUGAUGCCGACACUAu 5' | Ref: 5' aaGAGGCTGCTATGGCTGTGATa 3' |
| gga-miR-7475-5p | gi|297514485|gb|GT300154.1|GT300154 | #N/A | #N/A | 157 | -32.55 | 2 | 14 | 164 | 183 | 12 | Query: 3' ccucccgCGCCGCCGCCGCc 5' | Ref: 5' aagtttgGTGGTGGCGGCGg 3' |
| gga-miR-7475-5p | gi|297513377|gb|GT299058.1|GT299058 | #N/A | #N/A | 157 | -33.04 | 2 | 14 | 169 | 188 | 12 | Query: 3' ccucccgCGCCGCCGCCGCc 5' | Ref: 5' ggctatgGAGGCGGCGGCGg 3' |
| gga-miR-7475-5p | gi|297513007|gb|GT305096.1|GT305096 | #N/A | #N/A | 159 | -33.53 | 2 | 16 | 644 | 663 | 14 | Query: 3' ccuccCGCGCCGCCGCCGCc 5' | Ref: 5' tattcGTTTGGCGGCGGCGg 3' |
| gga-miR-7475-5p | gi|297511947|gb|GT299620.1|GT299620 | EGI61501 | 40s ribosomal protein | 149 | -31.76 | 2 | 15 | 2 | 22 | 14 | Query: 3' ccucccGCGC-CGCCGCCGCc 5' | Ref: 5' gggaacCACGTGTGGCGGCGg 3' |
| gga-miR-7475-5p | gi|297510934|gb|GT303321.1|GT303321 | #N/A | #N/A | 153 | -30.8 | 2 | 18 | 806 | 825 | 16 | Query: 3' ccuCCCGCGCCGCCGCCGCc 5' | Ref: 5' tctGCGTTTGGGGGCGGCGg 3' |
| gga-miR-7475-5p | gi|297509760|gb|GT298778.1|GT298778 | #N/A | #N/A | 159 | -30.42 | 2 | 19 | 140 | 159 | 18 | Query: 3' ccUCCCG-CGCCGCCGCCGCc 5' | Ref: 5' caAGCGCAGC-GCGGCGGCGt 3' |
| gga-miR-7475-5p | gi|297509408|gb|GT298438.1|GT298438 | #N/A | #N/A | 154 | -31.5 | 2 | 19 | 35 | 52 | 17 | Query: 3' ccUCCCGCGCCGCCGCCGCc 5' | Ref: 5' cgAGGGC-C-GAGGCGGCGt 3' |
| gga-miR-7475-5p | gi|297509305|gb|GT298338.1|GT298338 | #N/A | #N/A | 149 | -30.84 | 2 | 19 | 11 | 33 | 20 | Query: 3' ccUCCCGCGC---CGCCGCCGCc 5' | Ref: 5' agGGGATGCGATAGGGGCGGCGa 3' |
| gga-miR-7475-5p | gi|297509145|gb|GT298184.1|GT298184 | #N/A | #N/A | 151 | -33.25 | 2 | 16 | 37 | 56 | 14 | Query: 3' ccuccCGCGCCGCCGCCGCc 5' | Ref: 5' cttctGGGGGGGGGCGGCGg 3' |
| gga-miR-7475-5p | gi|297508675|gb|GT302497.1|GT302497 | NP_001087875 | mgc82014 protein | 172 | -34.45 | 2 | 17 | 190 | 209 | 15 | Query: 3' ccucCCGCGCCGCCGCCGCc 5' | Ref: 5' ccctGGCGCGCCGGCGGCGa 3' |
| gga-miR-7475-5p | gi|297508166|gb|GT311039.1|GT311039 | #N/A | #N/A | 151 | -31.38 | 2 | 14 | 1069 | 1087 | 12 | Query: 3' ccucccgCGCCGCCGCCGCc 5' | Ref: 5' atctttgGC-GCGGCGGCGc 3' |
| gga-miR-7475-5p | gi|297505708|gb|GT301331.1|GT301331 | XP_003400988 | proline synthase co-transcribed bacterial homolog | 158 | -34.25 | 2 | 17 | 620 | 638 | 15 | Query: 3' ccucCCGCGCCGCCGCCGCc 5' | Ref: 5' attcGGT-TGGCGGCGGCGg 3' |
| gga-miR-7475-5p | gi|297505658|gb|GT301236.1|GT301236 | #N/A | #N/A | 152 | -33.49 | 2 | 19 | 130 | 148 | 17 | Query: 3' ccUCCCGCGCCGCCGCCGCc 5' | Ref: 5' ccATGG-GGGGAGGCGGCGg 3' |
| gga-miR-7475-5p | gi|297505076|gb|GT309965.1|GT309965 | XP_975509 | delta-aminolevulinic acid dehydratase | 150 | -30.45 | 2 | 11 | 726 | 745 | 9 | Query: 3' ccucccgcgcCGCCGCCGCc 5' | Ref: 5' agcacaatacGCGGCGGCGg 3' |
| gga-miR-7475-5p | gi|297503450|gb|GT305043.1|GT305043 | XP_972141 | nucleoporin 85 | 164 | -39.01 | 2 | 17 | 1 | 16 | 15 | Query: 3' ccucCCGCGCCGCCGCCGCc 5' | Ref: 5' ----GGGGGGGCGGCGGCGg 3' |
| gga-miR-7475-5p | gi|297503032|gb|GT304621.1|GT304621 | #N/A | #N/A | 156 | -31.44 | 2 | 13 | 606 | 625 | 11 | Query: 3' ccucccgcGCCGCCGCCGCc 5' | Ref: 5' ctttccttTGGCGGCGGCGg 3' |
| gga-miR-7475-5p | gi|297501863|gb|GT311208.1|GT311208 | #N/A | #N/A | 165 | -34.86 | 2 | 19 | 41 | 61 | 18 | Query: 3' ccUCCCGCGCCG-CCGCCGCc 5' | Ref: 5' aaAGGCCTCGGCAGGCGGCGg 3' |
| gga-miR-7475-5p | gi|297501827|gb|GT301482.1|GT301482 | EHJ66162 | regulator of ribosome biosynthesis | 159 | -35.17 | 2 | 16 | 722 | 741 | 14 | Query: 3' ccuccCGCGCCGCCGCCGCc 5' | Ref: 5' gttttGGGTGGTGGCGGCGg 3' |
| ppc-miR-8316-3p | gi|297503895|gb|GT302537.1|GT302537 | XP_966810 | cg14661 cg14661-pa | 177 | -30.84 | 2 | 18 | 386 | 404 | 16 | Query: 3' cgCUGCUGGACCUGUGGUa 5' | Ref: 5' aaGCCGACCTGGACACCAa 3' |
| cgr-miR-412-5p | gi|297512888|gb|GT304932.1|GT304932 | XP_005183484 | atp synthase mitochondrial f1 complex assembly factor 2-like | 164 | -31.12 | 2 | 22 | 644 | 667 | 21 | Query: 3' uaAUGAAAGG-UCGACCAGCUGGu 5' | Ref: 5' aaTTCTGACCTTGCTGGTCGACCg 3' |
| hsa-miR-6787-5p | gi|297514625|gb|GT300247.1|GT300247 | XP_755363 | 40s ribosomal protein | 149 | -30.76 | 2 | 21 | 409 | 431 | 21 | Query: 3' cgUCGGUCGAGAU--GGGGGCGGu 5' | Ref: 5' agAG-CAGGTGAACGCCCCCGCCg 3' |
| hsa-miR-6787-5p | gi|297514021|gb|GT309159.1|GT309159 | EFR23061 | 39s ribosomal protein mitochondrial | 163 | -32.61 | 2 | 20 | 345 | 366 | 18 | Query: 3' cguCGGUCGAGAUGGGGGCGGu 5' | Ref: 5' ggcGTCATTTCATCCCCCGCCa 3' |
| hsa-miR-6787-5p | gi|297513287|gb|GT298882.1|GT298882 | XP_002434705 | tyrosine tryptophan | 153 | -32.64 | 2 | 20 | 1 | 26 | 22 | Query: 3' cguCGGU--CGA--GAUGGGGGCGGu 5' | Ref: 5' cgtGCCGCTGCTCGCCGCCCCCGCCt 3' |
| hsa-miR-6787-5p | gi|297512859|gb|GT304903.1|GT304903 | #N/A | #N/A | 167 | -32.27 | 2 | 17 | 496 | 518 | 16 | Query: 3' cgucggUCGA-GAUGGGGGCGGu 5' | Ref: 5' ctatagAGCTGCTGCCCCCGCCc 3' |
| hsa-miR-6787-5p | gi|297511927|gb|GT299554.1|GT299554 | #N/A | #N/A | 161 | -30.96 | 2 | 16 | 311 | 331 | 14 | Query: 3' cgucgguCGAGAUGGGGGCGGu 5' | Ref: 5' tgtctttGCT-TACCCCCGCCg 3' |
| hsa-miR-6787-5p | gi|297511500|gb|GT304443.1|GT304443 | #N/A | #N/A | 158 | -33.45 | 2 | 19 | 3 | 24 | 17 | Query: 3' cgucGGUCGAGAUGGGGGCGGu 5' | Ref: 5' gggaCCAGACTGGCCCCCGCCa 3' |
| hsa-miR-6787-5p | gi|297511291|gb|GT311488.1|GT311488 | XP_001662499 | lipase 1 precursor | 163 | -32.62 | 2 | 21 | 296 | 319 | 21 | Query: 3' cgUCGGUCGAG--AUGGGGGCGGu 5' | Ref: 5' tgGGCATGCTCTATATCCCCGCCa 3' |
| hsa-miR-6787-5p | gi|297511228|gb|GT303529.1|GT303529 | #N/A | #N/A | 170 | -34 | 2 | 21 | 6 | 26 | 19 | Query: 3' cgUCGGUCGAGAUGGGGGCGGu 5' | Ref: 5' ttATTCGGCT-TACCCCCGCCg 3' |
| hsa-miR-6787-5p | gi|297510178|gb|GT298488.1|GT298488 | #N/A | #N/A | 166 | -30.78 | 2 | 19 | 368 | 389 | 17 | Query: 3' cgucGGUCGAGAUGGGGGCGGu 5' | Ref: 5' ggccCCATTTTAACCCCCGCCa 3' |
| hsa-miR-6787-5p | gi|297510093|gb|GT300559.1|GT300559 | XP_001859028 | 39s ribosomal protein mitochondrial | 161 | -32.97 | 2 | 20 | 332 | 352 | 18 | Query: 3' cguCGGUCGAGAUGGGGGCGGu 5' | Ref: 5' ggcGTCAATTC-ATCCCCGCCg 3' |
| hsa-miR-6787-5p | gi|297510043|gb|GT308426.1|GT308426 | #N/A | #N/A | 160 | -31.77 | 2 | 18 | 106 | 129 | 18 | Query: 3' cgucgGUCGAGAU--GGGGGCGGu 5' | Ref: 5' gtctaCGGTTCTGTCCCCCCGCCt 3' |
| hsa-miR-6787-5p | gi|297509881|gb|GT308262.1|GT308262 | #N/A | #N/A | 159 | -31.46 | 2 | 21 | 433 | 456 | 21 | Query: 3' cgUCGGUCGA--GAUGGGGGCGGu 5' | Ref: 5' acACCTGGCTGATTCCCCCCGCCa 3' |
| hsa-miR-6787-5p | gi|297509433|gb|GT298463.1|GT298463 | #N/A | #N/A | 148 | -34.63 | 2 | 19 | 641 | 669 | 24 | Query: 3' cgucGGU-CGAGAU------GGGGGCGGu 5' | Ref: 5' atcaCCGCGCTCTAAACAACCCCCCGCCg 3' |
| hsa-miR-6787-5p | gi|297508542|gb|GT299907.1|GT299907 | CAQ03601 | cytochrome oxidase subunit ii | 150 | -31.37 | 2 | 15 | 11 | 32 | 13 | Query: 3' cgucggucGAGAUGGGGGCGGu 5' | Ref: 5' tggcggttTTCTCGCCCCGCCg 3' |
| hsa-miR-6787-5p | gi|297508267|gb|GT307819.1|GT307819 | #N/A | #N/A | 149 | -30.05 | 2 | 20 | 136 | 160 | 21 | Query: 3' cguCGGU--CG-AGAUGGGGGCGGu 5' | Ref: 5' gctGCCAAGGCGGCAAACCCCGCCa 3' |
| hsa-miR-6787-5p | gi|297507677|gb|GT310551.1|GT310551 | #N/A | #N/A | 159 | -31.46 | 2 | 21 | 63 | 86 | 21 | Query: 3' cgUCGGUCGA--GAUGGGGGCGGu 5' | Ref: 5' acACCTGGCTGATTCCCCCCGCCa 3' |
| hsa-miR-6787-5p | gi|297507305|gb|GT301988.1|GT301988 | #N/A | #N/A | 154 | -30.05 | 2 | 17 | 168 | 188 | 15 | Query: 3' cgucggUCGAGAUGGGGGCGGu 5' | Ref: 5' ttcaggAGTTCGA-CCCCGCCg 3' |
| hsa-miR-6787-5p | gi|297507013|gb|GT307884.1|GT307884 | XP_002104734 | GD21106 | 154 | -31.05 | 2 | 21 | 55 | 75 | 19 | Query: 3' cgUCGGUCGAGAUGGGGGCGGu 5' | Ref: 5' tgAGCCAG-GAACCCCCCGCCt 3' |
| hsa-miR-6787-5p | gi|297506043|gb|GT309490.1|GT309490 | XP_002003345 | dynamin 1-like protein | 163 | -32.14 | 2 | 21 | 10 | 32 | 20 | Query: 3' cgUCGGUCGAGA-UGGGGGCGGu 5' | Ref: 5' agGGCCCTTTCTCGCCCCCGCCc 3' |
| hsa-miR-6787-5p | gi|297504918|gb|GT309805.1|GT309805 | #N/A | #N/A | 155 | -31.12 | 2 | 20 | 2 | 23 | 18 | Query: 3' cguCGGUCGAGAUGGGGGCGGu 5' | Ref: 5' gtcGTGAGCGAGGCCCCCGCCg 3' |
| hsa-miR-6787-5p | gi|297504846|gb|GT306639.1|GT306639 | #N/A | #N/A | 164 | -34.32 | 2 | 20 | 607 | 628 | 19 | Query: 3' cguCGGUCGAGA-UGGGGGCGGu 5' | Ref: 5' cccGCCA-CTCTCCCCCCCGCCa 3' |
| hsa-miR-6787-5p | gi|297504072|gb|GT306085.1|GT306085 | #N/A | #N/A | 160 | -30.12 | 2 | 17 | 31 | 52 | 15 | Query: 3' cgucggUCGAGAUGGGGGCGGu 5' | Ref: 5' tccagaGGGTCCACCCCCGCCc 3' |
| hsa-miR-6787-5p | gi|297503500|gb|GT311618.1|GT311618 | #N/A | #N/A | 158 | -30.72 | 2 | 15 | 582 | 603 | 13 | Query: 3' cgucggucGAGAUGGGGGCGGu 5' | Ref: 5' cagttttaCTTTCCCCCCGCCa 3' |
| hsa-miR-6787-5p | gi|297503322|gb|GT311534.1|GT311534 | #N/A | #N/A | 166 | -33.73 | 2 | 15 | 337 | 358 | 13 | Query: 3' cgucggucGAGAUGGGGGCGGu 5' | Ref: 5' cacctcttCTCTATCCCCGCCg 3' |
| hsa-miR-6787-5p | gi|297503307|gb|GT311519.1|GT311519 | ESO97880 | methylcrotonoyl- carboxylase subunit mitochondrial-like | 150 | -31.29 | 2 | 11 | 182 | 203 | 9 | Query: 3' cgucggucgagaUGGGGGCGGu 5' | Ref: 5' gttatgtccagaACCCCCGCCa 3' |
| hsa-miR-6787-5p | gi|297503218|gb|GT303535.1|GT303535 | NP_647946 | CG10672 | 157 | -31.01 | 2 | 21 | 542 | 561 | 19 | Query: 3' cgUCGGUCGAGAUGGGGGCGGu 5' | Ref: 5' taGGTCAGCATT--CCCCGCCg 3' |
| hsa-miR-6787-5p | gi|297503140|gb|GT311446.1|GT311446 | ESO97880 | methylcrotonoyl- carboxylase subunit mitochondrial-like | 150 | -31.29 | 2 | 11 | 416 | 437 | 9 | Query: 3' cgucggucgagaUGGGGGCGGu 5' | Ref: 5' gttatgtccagaACCCCCGCCa 3' |
| hsa-miR-6787-5p | gi|297502848|gb|GT298934.1|GT298934 | #N/A | #N/A | 158 | -30.97 | 2 | 21 | 630 | 650 | 19 | Query: 3' cgUCGGUCGAGAUGGGGGCGGu 5' | Ref: 5' nnGGCGGGC-CCGCCCCCGCCa 3' |
| hsa-miR-6787-5p | gi|297502781|gb|GT298823.1|GT298823 | XP_312420 | AGAP002519-PA | 161 | -31.83 | 2 | 20 | 11 | 34 | 20 | Query: 3' cguCGGU-CGAG-AUGGGGGCGGu 5' | Ref: 5' tgcGTCGTGCTCATCCCCCCGCCc 3' |
| hsa-miR-6787-5p | gi|297501652|gb|GT308904.1|GT308904 | EFN81256 | dna-directed rna polymerase ii subunit rpb7-like | 160 | -30.42 | 2 | 19 | 441 | 461 | 17 | Query: 3' cgucGGUCGAGAUGGGGGCGGu 5' | Ref: 5' cacaCCA-TTCCATCCCCGCCg 3' |
| mmu-miR-412-5p | gi|297512888|gb|GT304932.1|GT304932 | XP_005183484 | atp synthase mitochondrial f1 complex assembly factor 2-like | 164 | -31.12 | 2 | 22 | 644 | 667 | 21 | Query: 3' uaAUGAAAGG-UCGACCAGCUGGu 5' | Ref: 5' aaTTCTGACCTTGCTGGTCGACCg 3' |
| hsa-miR-454-5p | gi|297514499|gb|GT304399.1|GT304399 | #N/A | #N/A | 167 | -30.35 | 2 | 21 | 224 | 248 | 22 | Query: 3' cgUCUCUGU---UAUAACUAUCCCa 5' | Ref: 5' acAGAGACAGGGATCGTGATAGGGa 3' |
| hsa-miR-454-5p | gi|297513901|gb|GT299758.1|GT299758 | #N/A | #N/A | 167 | -30.35 | 2 | 21 | 224 | 248 | 22 | Query: 3' cgUCUCUGU---UAUAACUAUCCCa 5' | Ref: 5' acAGAGACAGGGATCGTGATAGGGa 3' |
| gga-miR-1723 | gi|297505989|gb|GT301792.1|GT301792 | XP_973821 | PREDICTED: similar to AGAP003199-PA | 157 | -30.32 | 2 | 20 | 502 | 523 | 18 | Query: 3' acucCGACGUGUAAGGCGAGGGu 5' | Ref: 5' tggtGCTGC-CGACTCGCTCCCt 3' |
| gga-miR-1723 | gi|297502787|gb|GT298829.1|GT298829 | XP_003704427 | zinc finger protein 256-like | 182 | -40.79 | 2 | 21 | 426 | 447 | 19 | Query: 3' acuCCGACGUGUAAGGCGAGGGu 5' | Ref: 5' attGGCTGCACG-TCCGCTCCCa 3' |
| bmo-miR-3344 | gi|297512811|gb|GT311819.1|GT311819 | #N/A | #N/A | 171 | -30.39 | 2 | 22 | 66 | 93 | 23 | Query: 3' gagcGACC--GACUCAGG-AAGAACGUu 5' | Ref: 5' acttCTGGGCTTTAGTCCTTTCTTGCAa 3' |
| hsa-miR-4459 | gi|297514916|gb|GT308368.1|GT308368 | EFZ13829 | kad2_drops ame: full=adenylate kinase ame: full=atp-amp transphosphorylase ame: full=atp:amp phosphotransferase ame: full=adenylate kinase cytosolic and mitochondrial ame: full=adenylate monophosphate kinase | 156 | -30.29 | 2 | 18 | 189 | 211 | 17 | Query: 3' gagguGGAGGAGGC-GGAGGACc 5' | Ref: 5' gtcgtTCTCCTAGGCCCTCCTGg 3' |
| hsa-miR-4459 | gi|297514275|gb|GT309319.1|GT309319 | #N/A | #N/A | 179 | -38.59 | 2 | 20 | 389 | 410 | 18 | Query: 3' gagGUGGAGGAGGCGGAGGACc 5' | Ref: 5' gcaCACCACCTCCACCTCCTGg 3' |
| hsa-miR-4459 | gi|297513834|gb|GT303969.1|GT303969 | EHJ65023 | 2-oxoisovalerate dehydrogenase subunit mitochondrial-like | 154 | -30.07 | 2 | 20 | 765 | 783 | 18 | Query: 3' gagGUGGAGGAGGCGGAGGACc 5' | Ref: 5' aaaTACC-CCTC--CCTCCTGg 3' |
| hsa-miR-4459 | gi|297513120|gb|GT305301.1|GT305301 | #N/A | #N/A | 180 | -41.48 | 2 | 21 | 187 | 208 | 19 | Query: 3' gaGGUGGAGGAGGCGGAGGACc 5' | Ref: 5' ttCCATGTCCTCCTCCTCCTGg 3' |
| hsa-miR-4459 | gi|297512905|gb|GT311911.1|GT311911 | XP_001638799 | predicted protein | 167 | -36.39 | 2 | 20 | 264 | 285 | 18 | Query: 3' gagGUGGAGGAGGCGGAGGACc 5' | Ref: 5' ataCCCTTTCTGTGCCTCCTGg 3' |
| hsa-miR-4459 | gi|297512514|gb|GT307468.1|GT307468 | #N/A | #N/A | 167 | -31.97 | 2 | 21 | 53 | 76 | 21 | Query: 3' gaGGUGGAGGAG--GCGGAGGACc 5' | Ref: 5' gtCCACTTTCTTGGGGCCTCCTGa 3' |
| hsa-miR-4459 | gi|297511698|gb|GT311751.1|GT311751 | #N/A | #N/A | 170 | -38.36 | 2 | 21 | 52 | 72 | 19 | Query: 3' gaGGUGGAGGAGGCGGAGGACc 5' | Ref: 5' gaCCGCCACCTTC-CCTCCTGg 3' |
| hsa-miR-4459 | gi|297511477|gb|GT303692.1|GT303692 | #N/A | #N/A | 165 | -30.07 | 2 | 19 | 25 | 47 | 18 | Query: 3' gaggUGGAGGAGG-CGGAGGACc 5' | Ref: 5' ggagACCGACTCCGGCCTCCTGc 3' |
| hsa-miR-4459 | gi|297511029|gb|GT311322.1|GT311322 | #N/A | #N/A | 161 | -31.78 | 2 | 21 | 634 | 653 | 19 | Query: 3' gaGGUGGAGGAGGCGGAGGACc 5' | Ref: 5' ttCCCCCCCCTC--CCTCCTGg 3' |
| hsa-miR-4459 | gi|297510415|gb|GT298587.1|GT298587 | XP_002597516 | eukaryotic translation initiation factor 4 | 153 | -30.09 | 2 | 19 | 677 | 701 | 20 | Query: 3' gaggUGGAGGAGG---CGGAGGACc 5' | Ref: 5' cagaATCTCCGACATGGCCTCCTGg 3' |
| hsa-miR-4459 | gi|297510320|gb|GT308705.1|GT308705 | XP_397320 | ap-3 complex subunit sigma-2-like | 168 | -37.29 | 2 | 21 | 645 | 666 | 19 | Query: 3' gaGGUGGAGGAGGCGGAGGACc 5' | Ref: 5' ccCTGCCTAAGCCGCCTCCTGg 3' |
| hsa-miR-4459 | gi|297509784|gb|GT298802.1|GT298802 | #N/A | #N/A | 165 | -31.26 | 2 | 18 | 101 | 122 | 16 | Query: 3' gagguGGAGGAGGCGGAGGACc 5' | Ref: 5' tgaatCCTTCCCCCCCTCCTGg 3' |
| hsa-miR-4459 | gi|297509591|gb|GT298615.1|GT298615 | #N/A | #N/A | 167 | -36.39 | 2 | 20 | 254 | 275 | 18 | Query: 3' gagGUGGAGGAGGCGGAGGACc 5' | Ref: 5' ataCCCTTTCTGTGCCTCCTGg 3' |
| hsa-miR-4459 | gi|297509131|gb|GT298170.1|GT298170 | #N/A | #N/A | 164 | -32.29 | 2 | 20 | 333 | 352 | 18 | Query: 3' gagGUGGAGGAGGCGGAGGACc 5' | Ref: 5' ataCAACTCCT--GCCTCCTGg 3' |
| hsa-miR-4459 | gi|297508858|gb|GT302680.1|GT302680 | #N/A | #N/A | 164 | -33.14 | 2 | 18 | 212 | 234 | 17 | Query: 3' gagguGGAGGAGGC-GGAGGACc 5' | Ref: 5' gtcgtTCTCCTACGCCCTCCTGg 3' |
| hsa-miR-4459 | gi|297507703|gb|GT310577.1|GT310577 | #N/A | #N/A | 155 | -39.65 | 2 | 21 | 52 | 79 | 25 | Query: 3' gaGGUGGAGGAGG------CGGAGGACc 5' | Ref: 5' ctCCGCGTCTTCCAGATGAGCCTCCTGg 3' |
| hsa-miR-4459 | gi|297507213|gb|GT301804.1|GT301804 | #N/A | #N/A | 161 | -31.15 | 2 | 18 | 32 | 53 | 16 | Query: 3' gagguGGAGGAGGCGGAGGACc 5' | Ref: 5' cagtcCATCCTCGTCCTCCTGg 3' |
| hsa-miR-4459 | gi|297507175|gb|GT301720.1|GT301720 | #N/A | #N/A | 170 | -33.11 | 2 | 21 | 229 | 249 | 19 | Query: 3' gaGGUGGAGGAGGCGGAGGACc 5' | Ref: 5' caTCGCTTCTTCC-CCTCCTGc 3' |
| hsa-miR-4459 | gi|297506646|gb|GT305766.1|GT305766 | #N/A | #N/A | 155 | -30.85 | 2 | 21 | 194 | 219 | 23 | Query: 3' gaGGUGGAGGA----GGCGGAGGACc 5' | Ref: 5' ccCTCCCTTTTGAACTCGCCTCCTGa 3' |
| hsa-miR-4459 | gi|297506248|gb|GT302051.1|GT302051 | XP_003705928 | anaphase-promoting complex subunit cdc26-like | 159 | -30.01 | 2 | 21 | 93 | 116 | 21 | Query: 3' gaGGUGGAG--GAGGCGGAGGACc 5' | Ref: 5' gtCTACTTTTATTCTTCCTCCTGg 3' |
| hsa-miR-4459 | gi|297505336|gb|GT310243.1|GT310243 | NP_001165801 | succinate dehydrogenase subunit flavoprotein | 170 | -32.7 | 2 | 21 | 283 | 303 | 19 | Query: 3' gaGGUGGAGGAGGCGGAGGACc 5' | Ref: 5' agCCACCCTCTCT-CCTCCTGa 3' |
| hsa-miR-4459 | gi|297504458|gb|GT302926.1|GT302926 | #N/A | #N/A | 159 | -33.44 | 2 | 21 | 497 | 515 | 19 | Query: 3' gaGGUGGAGGAGGCGGAGGACc 5' | Ref: 5' ggCCATC-CCTC--CCTCCTGg 3' |
| hsa-miR-4459 | gi|297504421|gb|GT305937.1|GT305937 | #N/A | #N/A | 160 | -31.44 | 2 | 21 | 298 | 317 | 19 | Query: 3' gaGGUGGAGGAGGCGGAGGACc 5' | Ref: 5' ttTCCCCT-CTCC-CCTCCTGt 3' |
| hsa-miR-4459 | gi|297504051|gb|GT302651.1|GT302651 | EKV20488 | succinyl- synthetase beta | 161 | -30.37 | 2 | 21 | 89 | 110 | 20 | Query: 3' gaGGUGGAGGAGGC-GGAGGACc 5' | Ref: 5' ttCAACCT-CGCCGTCCTCCTGa 3' |
| hsa-miR-4459 | gi|297503678|gb|GT311702.1|GT311702 | XP_003072433 | 60s ribosomal protein l9 | 159 | -30.16 | 2 | 18 | 251 | 271 | 16 | Query: 3' gagguGGAGGAGGCGGAGGACc 5' | Ref: 5' aaaacCTTTCTTC-CCTCCTGg 3' |
| hsa-miR-4459 | gi|297502043|gb|GT311296.1|GT311296 | EFN66779 | esf1-like protein | 155 | -31.48 | 2 | 16 | 592 | 613 | 14 | Query: 3' gagguggAGGAGGCGGAGGACc 5' | Ref: 5' agcttgaTCTATTGCCTCCTGg 3' |
| hsa-miR-4459 | gi|297502040|gb|GT311293.1|GT311293 | #N/A | #N/A | 184 | -38.77 | 2 | 21 | 243 | 264 | 19 | Query: 3' gaGGUGGAGGAGGCGGAGGACc 5' | Ref: 5' atCCTCCTCCTCCTCCTCCTGt 3' |
| hsa-miR-4459 | gi|297501979|gb|GT301638.1|GT301638 | XP_003801312 | adenylate kinase mitochondrial | 156 | -30.29 | 2 | 18 | 189 | 211 | 17 | Query: 3' gagguGGAGGAGGC-GGAGGACc 5' | Ref: 5' gtcgtTCTCCTAGGCCCTCCTGg 3' |
| hsa-miR-4459 | gi|297501938|gb|GT303280.1|GT303280 | #N/A | #N/A | 180 | -34.83 | 2 | 21 | 465 | 486 | 19 | Query: 3' gaGGUGGAGGAGGCGGAGGACc 5' | Ref: 5' caTTACTTCTTCTGCCTCCTGc 3' |
| hsa-miR-4638-3p | gi|297513471|gb|GT299242.1|GT299242 | XP_001600011 | PREDICTED: prohibitin-2 | 177 | -30.57 | 2 | 22 | 258 | 280 | 20 | Query: 3' gcCGGCCGACUCGCCACAGGUCc 5' | Ref: 5' caGTAGATTGAGTGGTGTCCAGa 3' |
| hsa-miR-4638-3p | gi|297510659|gb|GT303132.1|GT303132 | EIM19765 | survival of motor neuron-related-splicing factor 30 | 167 | -31.36 | 2 | 20 | 646 | 668 | 18 | Query: 3' gccgGCCGACUCGCCACAGGUCc 5' | Ref: 5' cgtaTGGTAGAGTCGTGTCCAGg 3' |
| hsa-miR-4638-3p | gi|297504556|gb|GT306028.1|GT306028 | #N/A | #N/A | 164 | -34.07 | 2 | 22 | 105 | 128 | 21 | Query: 3' gcCGGCCGACUCGC-CACAGGUCc 5' | Ref: 5' cgGCCGGGGGACCGTCTGTCCAGt 3' |
| mja-miR-6493-5p | gi|297511384|gb|GT304370.1|GT304370 | #N/A | #N/A | 159 | -31.38 | 2 | 21 | 454 | 478 | 22 | Query: 3' ucCCCAUUUUGG---ACGGCCUGCa 5' | Ref: 5' cgGGGAAGGACCCTTTTCCGGACGt 3' |
| mja-miR-6493-5p | gi|297507563|gb|GT310438.1|GT310438 | #N/A | #N/A | 174 | -34.54 | 2 | 20 | 267 | 289 | 19 | Query: 3' uccCCAU-UUUGGACGGCCUGCa 5' | Ref: 5' gcaGGTGCACACCTGCCGGACGt 3' |
| mja-miR-6493-5p | gi|297506512|gb|GT305497.1|GT305497 | #N/A | #N/A | 163 | -30.18 | 2 | 21 | 444 | 467 | 21 | Query: 3' ucCCCAUUUUGG--ACGGCCUGCa 5' | Ref: 5' tcGGGGAGGACCCTTTCCGGACGg 3' |
| mja-miR-6493-5p | gi|297503334|gb|GT311546.1|GT311546 | XP_005047381 | isoform b | 162 | -30.69 | 2 | 21 | 423 | 443 | 19 | Query: 3' ucCCCAUUUUGGACGGCCUGCa 5' | Ref: 5' gaGGGTACCTCC-GCCGGACGc 3' |
| ssa-miR-15a-5p | gi|297508049|gb|GT310922.1|GT310922 | #N/A | #N/A | 164 | -31.52 | 2 | 19 | 210 | 234 | 19 | Query: 3' uguuuGGUACUG-C-ACGACGAUGc 5' | Ref: 5' ccgtcTCATGGCAGATGCTGCTACg 3' |
|  |  |  |  |  |  |  |  |  |  |  |  |  |
| **The first report of miRNAome from a thysanopteran insect, Thrips palmi Karny using high-throughput sequencing.**  **Authors : K. B. Rebijith, R. Asokan, H. Ranjitha Hande and N. K. Krishna Kumar** | | | | | | | | | | | | |
