# Supplementary material for: The First Report of miRNAs from a Thysanopteran Insect, Thrips palmi Karny Using High-Throughput Sequencing
Source: PLoS One. 2016 Sep 29;11(9):e0163635. doi: 10.1371/journal.pone.0163635 (PMC5042526; doi:10.1371/journal.pone.0163635)
Supplement: S3 Table — (DOC) [file pone.0163635.s003.doc]

| **Supplementary Table S3. Potential targets for the identified known miRNAs with the transcriptomic sequences of *F. occidentalis.*** | | | | | | | | | | | | |
| --- | --- | --- | --- | --- | --- | --- | --- | --- | --- | --- | --- | --- |
| **miRNA** | **Target Id** | **Hit Acc.** | **Protein Name** | **Alignment score** | **Energy** | **miRNA_start** | **miRNA_end** | **Target_start** | **Target_end** | **Alignment length** | **miRNA_aligned_fragment** | **Target_aligned_fragment** |
| hsa-miR-4459 | gi|619320369|gb|GAXD01000380.1| | XP_975653 | o-glycosyl hydrolase family 30 protein | 162 | -37.62 | 2 | 21 | 1361 | 1385 | 22 | Query: 3' gaGGUGGA--GGAGGC-GGAGGACc 5' | Ref: 5' ctCCATCTGGTCACCGCCCTCCTGg 3' |
| gga-miR-7475-5p | gi|619292414|gb|GAXD01027724.1| | XP_975371 | PREDICTED: similar to GA21917-PA | 156 | -30.4 | 2 | 18 | 188 | 208 | 17 | Query: 3' ccuCCCGC-GCCGCCGCCGCc 5' | Ref: 5' gccGGGCGTTCCCGGCGGCGt 3' |
| mja-miR-6493-3p | gi|619298543|gb|GAXD01022078.1| | XP_974917 | PREDICTED: similar to tensin | 160 | -33.43 | 2 | 20 | 210 | 233 | 19 | Query: 3' auugcGAGUCGCGCC-AAAGGGGGa 5' | Ref: 5' tttgaCTCGGC-CGGCCTTCCCCCt 3' |
| hsa-miR-6787-5p | gi|619308326|gb|GAXD01012296.1| | XP_974031 | solute carrier family 41 member 2 | 160 | -31.41 | 2 | 21 | 1318 | 1339 | 19 | Query: 3' cgUCGGUCGAGAUGGGGGCGGu 5' | Ref: 5' acAGACTCGTCTCCCCCCGCCa 3' |
| gga-miR-7475-5p | gi|619320182|gb|GAXD01000567.1| | XP_973999 | cdna sequence bc060632 | 148 | -31.06 | 2 | 19 | 1863 | 1886 | 21 | Query: 3' ccUCCCGCG---CCG-CCGCCGCc 5' | Ref: 5' ggAAGGCGTCCAGGTAGGCGGCGa 3' |
| gga-miR-7475-5p | gi|619318013|gb|GAXD01002736.1| | XP_973587 | deoxyribonuclease i | 154 | -38.41 | 2 | 19 | 962 | 981 | 17 | Query: 3' ccUCCCGCGCCGCCGCCGCc 5' | Ref: 5' gcGGAGGGAGGAGGCGGCGg 3' |
| gga-miR-7475-5p | gi|619318012|gb|GAXD01002737.1| | XP_973587 | deoxyribonuclease i | 154 | -38.41 | 2 | 19 | 1264 | 1283 | 17 | Query: 3' ccUCCCGCGCCGCCGCCGCc 5' | Ref: 5' gcGGAGGGAGGAGGCGGCGg 3' |
| hsa-miR-4459 | gi|619298228|gb|GAXD01022393.1| | XP_973382 | brg-1 associated factor | 154 | -30.58 | 2 | 21 | 181 | 201 | 19 | Query: 3' gaGGUGGAGGAGGCGGAGGACc 5' | Ref: 5' taCCGAC-ACACTGCCTCCTGg 3' |
| gga-miR-7475-5p | gi|619319534|gb|GAXD01001215.1| | XP_973200 | PREDICTED: similar to Uncharacterized protein KIAA1370 homolog | 149 | -31.47 | 2 | 19 | 127 | 149 | 20 | Query: 3' ccUCCCGCGCCG---CCGCCGCc 5' | Ref: 5' gaAGGACGGGTCCCAGGCGGCGg 3' |
| gga-miR-7475-5p | gi|619300140|gb|GAXD01020482.1| | XP_973200 | PREDICTED: similar to Uncharacterized protein KIAA1370 homolog | 149 | -31.47 | 2 | 19 | 127 | 149 | 20 | Query: 3' ccUCCCGCGCCG---CCGCCGCc 5' | Ref: 5' gaAGGACGGGTCCCAGGCGGCGg 3' |
| gga-miR-7475-5p | gi|619300139|gb|GAXD01020483.1| | XP_973200 | PREDICTED: similar to Uncharacterized protein KIAA1370 homolog | 149 | -31.47 | 2 | 19 | 127 | 149 | 20 | Query: 3' ccUCCCGCGCCG---CCGCCGCc 5' | Ref: 5' gaAGGACGGGTCCCAGGCGGCGg 3' |
| hsa-miR-4459 | gi|619312554|gb|GAXD01008195.1| | XP_972501 | dead-box rna-dependent helicase p68 | 174 | -31.88 | 2 | 19 | 1818 | 1839 | 17 | Query: 3' gaggUGGAGGAGGCGGAGGACc 5' | Ref: 5' cgtgACCTGCTGCGCCTCCTGc 3' |
| ppc-miR-71a | gi|619294769|gb|GAXD01025369.1| | XP_972394 | PREDICTED: similar to dynactin | 178 | -30.47 | 2 | 19 | 270 | 294 | 17 | Query: 3' gcagaguGAUGGUUGUCCAGAAAGu 5' | Ref: 5' aggtaatTTGCTAACAGGTCTTTCa 3' |
| hsa-miR-4459 | gi|619320354|gb|GAXD01000395.1| | XP_972068 | basement membrane-specific heparan sulfate proteoglycan core | 169 | -32.31 | 2 | 18 | 1777 | 1798 | 16 | Query: 3' gagguGGAGGAGGCGGAGGACc 5' | Ref: 5' ataatCTTCCTCTTCCTCCTGg 3' |
| hsa-miR-4459 | gi|619317965|gb|GAXD01002784.1| | XP_972068 | low quality protein: basement membrane-specific heparan sulfate proteoglycan core | 169 | -32.31 | 2 | 18 | 1657 | 1678 | 16 | Query: 3' gagguGGAGGAGGCGGAGGACc 5' | Ref: 5' ataatCTTCCTCTTCCTCCTGg 3' |
| hsa-miR-6787-5p | gi|619298732|gb|GAXD01021889.1| | XP_971500 | midline fasciclin | 150 | -31.78 | 2 | 11 | 238 | 259 | 9 | Query: 3' cgucggucgagaUGGGGGCGGu 5' | Ref: 5' ccccagaaagagACCCCCGCCa 3' |
| gga-miR-7475-5p | gi|619296120|gb|GAXD01024018.1| | XP_970124 | citrate synthase | 165 | -33.09 | 2 | 19 | 2949 | 2969 | 18 | Query: 3' ccUCCCG-CGCCGCCGCCGCc 5' | Ref: 5' taAGGGCGGTGATGGCGGCGc 3' |
| hsa-miR-4459 | gi|619304915|gb|GAXD01015707.1| | XP_968889 | large subunit gtpase 1 homolog | 155 | -30.32 | 2 | 21 | 271 | 293 | 20 | Query: 3' gaGGUGGAGG-AGGCGGAGGACc 5' | Ref: 5' ctCTACTGCTGTGCACCTCCTGg 3' |
| hsa-miR-4459 | gi|619306139|gb|GAXD01014483.1| | XP_968639 | clathrin coat assembly protein ap- | 167 | -32.03 | 2 | 21 | 953 | 976 | 21 | Query: 3' gaGGUGGAGGAGG--CGGAGGACc 5' | Ref: 5' aaCCATCTCTTTCATTCCTCCTGa 3' |
| hsa-miR-4459 | gi|619306138|gb|GAXD01014484.1| | XP_968639 | clathrin coat assembly protein ap- | 167 | -32.03 | 2 | 21 | 953 | 976 | 21 | Query: 3' gaGGUGGAGGAGG--CGGAGGACc 5' | Ref: 5' aaCCATCTCTTTCATTCCTCCTGa 3' |
| gga-miR-7475-5p | gi|619316177|gb|GAXD01004572.1| | XP_968538 | heterogeneous nuclear ribonucleoprotein k-like | 167 | -41.65 | 2 | 17 | 94 | 114 | 16 | Query: 3' ccucCCG-CGCCGCCGCCGCc 5' | Ref: 5' cggtGGCGGCGGTGGCGGCGg 3' |
| hsa-miR-4459 | gi|619297564|gb|GAXD01023057.1| | XP_968493 | wd repeat domain 70 | 169 | -30.66 | 2 | 18 | 564 | 585 | 16 | Query: 3' gagguGGAGGAGGCGGAGGACc 5' | Ref: 5' cttttCCTTTTTTGCCTCCTGa 3' |
| hsa-miR-4459 | gi|619297562|gb|GAXD01023059.1| | XP_968493 | wd repeat domain 70 | 169 | -30.66 | 2 | 18 | 564 | 585 | 16 | Query: 3' gagguGGAGGAGGCGGAGGACc 5' | Ref: 5' cttttCCTTTTTTGCCTCCTGa 3' |
| hsa-miR-4459 | gi|619316518|gb|GAXD01004231.1| | XP_968050 | udp-n-acetylglucosamine--dolichyl-phosphate n-acetylglucosaminephosphotransferase-like | 171 | -30.32 | 2 | 20 | 2398 | 2419 | 18 | Query: 3' gagGUGGAGGAGGCGGAGGACc 5' | Ref: 5' agaCAAATCTTCTGCCTCCTGt 3' |
| hsa-miR-4459 | gi|619319236|gb|GAXD01001513.1| | XP_967163 | cold shock domain-containing protein e1-like | 174 | -35.85 | 2 | 19 | 380 | 401 | 17 | Query: 3' gaggUGGAGGAGGCGGAGGACc 5' | Ref: 5' gcagACCACCTCCACCTCCTGg 3' |
| hsa-miR-4459 | gi|619316661|gb|GAXD01004088.1| | XP_967163 | cold shock domain-containing protein e1-like | 174 | -35.85 | 2 | 19 | 380 | 401 | 17 | Query: 3' gaggUGGAGGAGGCGGAGGACc 5' | Ref: 5' gcagACCACCTCCACCTCCTGg 3' |
| rno-miR-412-5p | gi|619299167|gb|GAXD01021454.1| | XP_966556 | wd40 protein | 176 | -31.42 | 2 | 17 | 522 | 541 | 15 | Query: 3' ugaaAGGUCGACCAGCUGGu 5' | Ref: 5' tgcaTCTAGCTGGTCGACCt 3' |
| cgr-miR-412-5p | gi|619299167|gb|GAXD01021454.1| | XP_966556 | wd40 protein | 176 | -31.55 | 2 | 17 | 519 | 541 | 15 | Query: 3' uaaugaaAGGUCGACCAGCUGGu 5' | Ref: 5' taatgcaTCTAGCTGGTCGACCt 3' |
| mmu-miR-412-5p | gi|619299167|gb|GAXD01021454.1| | XP_966556 | wd40 protein | 176 | -31.55 | 2 | 17 | 519 | 541 | 15 | Query: 3' uaaugaaAGGUCGACCAGCUGGu 5' | Ref: 5' taatgcaTCTAGCTGGTCGACCt 3' |
| hsa-miR-4459 | gi|619307707|gb|GAXD01012915.1| | XP_955598 | histone h3 | 161 | -31.36 | 2 | 18 | 132 | 153 | 16 | Query: 3' gagguGGAGGAGGCGGAGGACc 5' | Ref: 5' gaagtTTTCAACCGCCTCCTGg 3' |
| hsa-miR-4459 | gi|619308342|gb|GAXD01012280.1| | XP_510851 | nodal modulator partial | 158 | -33.93 | 2 | 21 | 291 | 311 | 19 | Query: 3' gaGGUGGAGGAGGCGGAGGACc 5' | Ref: 5' ctTTACCAGC-CTGCCTCCTGg 3' |
| gga-miR-7475-5p | gi|619318201|gb|GAXD01002548.1| | XP_394539 | PREDICTED: hypothetical protein LOC411065 isoform 1 | 166 | -33.65 | 2 | 19 | 1356 | 1375 | 17 | Query: 3' ccUCCCGCGCCGCCGCCGCc 5' | Ref: 5' cgAGGCCGCCGAGGCGGCGg 3' |
| gga-miR-7475-5p | gi|619300045|gb|GAXD01020577.1| | XP_394539 | PREDICTED: hypothetical protein LOC411065 isoform 1 | 166 | -33.65 | 2 | 19 | 977 | 996 | 17 | Query: 3' ccUCCCGCGCCGCCGCCGCc 5' | Ref: 5' cgAGGCCGCCGAGGCGGCGg 3' |
| hsa-miR-4638-3p | gi|619313404|gb|GAXD01007345.1| | XP_394336 | PREDICTED: battenin | 157 | -31.36 | 2 | 21 | 376 | 398 | 20 | Query: 3' gccGGCCG-ACUCGCCACAGGUCc 5' | Ref: 5' agaTCGGCGTGTTC-GTGTCCAGg 3' |
| efu-miR-9198a | gi|619319202|gb|GAXD01001547.1| | XP_391843 | 3-ketoacyl- mitochondrial | 164 | -31.73 | 2 | 22 | 1194 | 1217 | 21 | Query: 3' agUGUAGGUGACUG-UCACGGUUc 5' | Ref: 5' gcACAAACATTGGCTTGTGCCAAg 3' |
| mmu-miR-6382 | gi|619305048|gb|GAXD01015574.1| | XP_321925 | alpha- -mannosyltransferase alg2-like | 172 | -30.01 | 2 | 23 | 218 | 244 | 24 | Query: 3' gaACA-CACGAGAGA--AAUGUAAGGu 5' | Ref: 5' taTGTAGTGCTCTCTAACAACATTCCa 3' |
| gga-miR-7475-5p | gi|619295278|gb|GAXD01024860.1| | XP_319584 | aquaporin -like | 150 | -30.09 | 2 | 17 | 819 | 843 | 20 | Query: 3' ccucCCGCG----CC-GCCGCCGCc 5' | Ref: 5' tcgcGGCGCCCCTGGTCGGCGGCGc 3' |
| hsa-miR-6787-5p | gi|619320076|gb|GAXD01000673.1| | XP_317885 | guanyl-nucleotide exchange factor | 155 | -32.33 | 2 | 21 | 305 | 328 | 21 | Query: 3' cgUCGGUCGAG--AUGGGGGCGGu 5' | Ref: 5' ggACCCGGCCCCGGGCCCCCGCCg 3' |
| hsa-miR-6787-5p | gi|619300250|gb|GAXD01020372.1| | XP_317493 | dihydrolipoamide acetyltransferase component of pyruvate dehydrogenase | 156 | -30.68 | 2 | 18 | 571 | 593 | 17 | Query: 3' cgucgGUCGA-GAUGGGGGCGGu 5' | Ref: 5' tgctgCACCTGCAGCCCCCGCCg 3' |
| hsa-miR-6787-5p | gi|619300248|gb|GAXD01020374.1| | XP_317493 | dihydrolipoamide acetyltransferase component of pyruvate dehydrogenase | 156 | -30.68 | 2 | 18 | 571 | 593 | 17 | Query: 3' cgucgGUCGA-GAUGGGGGCGGu 5' | Ref: 5' tgctgCACCTGCAGCCCCCGCCg 3' |
| gga-miR-7475-5p | gi|619295064|gb|GAXD01025074.1| | XP_311188 | AGAP000663-PA | 160 | -31.41 | 2 | 18 | 399 | 419 | 17 | Query: 3' ccuCCCGCGC-CGCCGCCGCc 5' | Ref: 5' cttGGGCGGGAGGGGCGGCGc 3' |
| hsa-miR-4459 | gi|619317095|gb|GAXD01003654.1| | XP_006157234 | small nuclear ribonucleoprotein sm d3 isoform x2 | 165 | -37.39 | 2 | 21 | 230 | 251 | 20 | Query: 3' gaGGUGGAGGAGGC-GGAGGACc 5' | Ref: 5' agCCACCATC-CCGTCCTCCTGg 3' |
| gga-miR-7475-5p | gi|619299465|gb|GAXD01021156.1| | XP_005186310 | monocarboxylate transporter 14-like isoform x2 | 147 | -31.03 | 2 | 19 | 242 | 259 | 17 | Query: 3' ccUCCCGCGCCGCCGCCGCc 5' | Ref: 5' ggAGGGTCT--TGGCGGCGc 3' |
| gga-miR-7475-5p | gi|619319294|gb|GAXD01001455.1| | XP_005183229 | synaptic vesicle glycoprotein 2b-like | 150 | -35.87 | 2 | 17 | 1388 | 1411 | 19 | Query: 3' ccucCCGCG-CCG---CCGCCGCc 5' | Ref: 5' ggtaGGCGTAGGCCAGGGCGGCGg 3' |
| hsa-miR-6787-5p | gi|619297567|gb|GAXD01023054.1| | XP_005182256 | peptidylglycine alpha-hydroxylating monooxygenase-like | 154 | -31.58 | 2 | 21 | 466 | 486 | 19 | Query: 3' cgUCGGUCGAGAUGGGGGCGGu 5' | Ref: 5' ccAGCGGCCGCTG-CCCCGCCt 3' |
| hsa-miR-4459 | gi|619303214|gb|GAXD01017408.1| | XP_005181790 | zinc finger rna-binding protein 2-like isoform x1 | 159 | -34.67 | 2 | 21 | 1255 | 1279 | 22 | Query: 3' gaGGUGGAGGAGGC---GGAGGACc 5' | Ref: 5' atCCCCGTCCTTTGGGACCTCCTGg 3' |
| hsa-miR-6787-5p | gi|619295698|gb|GAXD01024440.1| | XP_004934452 | nad-dependent protein deacetylase sirtuin-4 isoform x3 | 167 | -32.92 | 2 | 20 | 776 | 797 | 18 | Query: 3' cguCGGUCGAGAUGGGGGCGGu 5' | Ref: 5' gccGCGGGTTCGATCCCCGCCa 3' |
| hsa-miR-4459 | gi|619318122|gb|GAXD01002627.1| | XP_004931987 | phospholipase a-2-activating | 172 | -32.02 | 2 | 21 | 274 | 295 | 19 | Query: 3' gaGGUGGAGGAGGCGGAGGACc 5' | Ref: 5' tcCTACCTCCACGTCCTCCTGt 3' |
| mja-miR-6493-3p | gi|619320106|gb|GAXD01000643.1| | XP_004931363 | mannose-1-phosphate guanyltransferase beta-like | 156 | -31.05 | 2 | 23 | 99 | 124 | 23 | Query: 3' auUGCGAGU-CG-CGCCAAAGGGGGa 5' | Ref: 5' aaATGCCGATGTGGTTGTTTCCCCCc 3' |
| gga-miR-1723 | gi|619320357|gb|GAXD01000392.1| | XP_004931128 | v-type proton atpase 116 kda subunit a isoform 1-like isoform x1 | 165 | -36.2 | 2 | 22 | 247 | 267 | 20 | Query: 3' acUCCGACGUGUAAGGCGAGGGu 5' | Ref: 5' ggGGGC-GCTC-TTCCGCTCCCg 3' |
| gga-miR-1723 | gi|619308079|gb|GAXD01012543.1| | XP_004931128 | v-type proton atpase 116 kda subunit a isoform 1-like isoform x1 | 165 | -36.2 | 2 | 22 | 247 | 267 | 20 | Query: 3' acUCCGACGUGUAAGGCGAGGGu 5' | Ref: 5' ggGGGC-GCTC-TTCCGCTCCCg 3' |
| ppc-miR-71a | gi|619297950|gb|GAXD01022671.1| | XP_004928213 | diphthine--ammonia ligase-like isoform x1 | 181 | -31.83 | 2 | 23 | 151 | 176 | 22 | Query: 3' gcaGAGUGAU-GGUUGUCCAGAAAGu 5' | Ref: 5' tgaCTCACCAGACAATAGGTCTTTCa 3' |
| hsa-miR-4638-3p | gi|619297263|gb|GAXD01023358.1| | XP_004924182 | e3 ubiquitin-protein ligase znrf2-like | 168 | -32.19 | 2 | 19 | 1372 | 1393 | 17 | Query: 3' gccggCCGACUCGCCACAGGUCc 5' | Ref: 5' tcctaGGCTGAG-GCTGTCCAGg 3' |
| gga-miR-7475-5p | gi|619297356|gb|GAXD01023265.1| | XP_004922059 | oxidase peroxidase | 159 | -30.05 | 2 | 16 | 753 | 772 | 14 | Query: 3' ccuccCGCGCCGCCGCCGCc 5' | Ref: 5' acgctGCACCGCGGCGGCGg 3' |
| gga-miR-7475-5p | gi|619297353|gb|GAXD01023268.1| | XP_004922059 | PREDICTED: uncharacterized protein LOC101744777 | 159 | -30.05 | 2 | 16 | 753 | 772 | 14 | Query: 3' ccuccCGCGCCGCCGCCGCc 5' | Ref: 5' acgctGCACCGCGGCGGCGg 3' |
| hsa-miR-6787-5p | gi|619297353|gb|GAXD01023268.1| | XP_004922059 | PREDICTED: uncharacterized protein LOC101744777 | 157 | -31.9 | 2 | 20 | 1306 | 1330 | 21 | Query: 3' cguCGGUCG-AGAUG--GGGGCGGu 5' | Ref: 5' tgcGCCCGCATCGACATCCCCGCCa 3' |
| gga-miR-7475-5p | gi|619318421|gb|GAXD01002328.1| | XP_004921895 | PREDICTED: uncharacterized protein LOC101741041 | 169 | -33.1 | 2 | 18 | 332 | 351 | 16 | Query: 3' ccuCCCGCGCCGCCGCCGCc 5' | Ref: 5' gatGCGCGCGGGGGCGGCGc 3' |
| ppc-miR-8316-3p | gi|619299182|gb|GAXD01021439.1| | XP_004749677 | ectonucleoside triphosphate diphosphohydrolase 1 isoform x2 | 172 | -31.12 | 2 | 18 | 1317 | 1336 | 17 | Query: 3' cgCUGCUGGAC-CUGUGGUa 5' | Ref: 5' gaGGCGACCTGCGACACCAc 3' |
| ppc-miR-8316-3p | gi|619299181|gb|GAXD01021440.1| | XP_004749677 | ectonucleoside triphosphate diphosphohydrolase 1 isoform x2 | 172 | -31.12 | 2 | 18 | 1301 | 1320 | 17 | Query: 3' cgCUGCUGGAC-CUGUGGUa 5' | Ref: 5' gaGGCGACCTGCGACACCAc 3' |
| gga-miR-7475-5p | gi|619298393|gb|GAXD01022228.1| | XP_004535629 | aminopeptidase n-like | 159 | -33.04 | 2 | 17 | 788 | 809 | 17 | Query: 3' ccucCCGC--GCCGCCGCCGCc 5' | Ref: 5' gggcGGCGAACGACGGCGGCGa 3' |
| gga-miR-7475-5p | gi|619298393|gb|GAXD01022228.1| | XP_004535629 | aminopeptidase n-like | 150 | -34.9 | 2 | 17 | 1544 | 1567 | 19 | Query: 3' ccucCCGCGCC-G---CCGCCGCc 5' | Ref: 5' tgtcGGCGTGGACGAGGGCGGCGg 3' |
| gga-miR-7475-5p | gi|619295556|gb|GAXD01024582.1| | XP_004533591 | PREDICTED: gelsolin-like | 153 | -32.39 | 2 | 19 | 880 | 900 | 18 | Query: 3' ccUCCCGCGCC-GCCGCCGCc 5' | Ref: 5' ccACGGAGAGGATGGCGGCGg 3' |
| gga-miR-1723 | gi|619313345|gb|GAXD01007404.1| | XP_004529416 | low-density lipoprotein | 151 | -30.68 | 2 | 22 | 1653 | 1672 | 20 | Query: 3' acUCCGACGUGUAAGGCGAGGGu 5' | Ref: 5' cgAGG-TGC-CGGT-CGCTCCCg 3' |
| mja-miR-6493-3p | gi|619299959|gb|GAXD01020663.1| | XP_004154316 | PREDICTED: uncharacterized protein LOC101218508, partial | 162 | -31.91 | 2 | 23 | 424 | 449 | 24 | Query: 3' auUGCG--AGUC-GCGCCAAAGGGGGa 5' | Ref: 5' gaGCGCTATCAGACG-GGCTTCCCCCg 3' |
| hsa-miR-4459 | gi|619295370|gb|GAXD01024768.1| | XP_003887545 | hypothetical protein EHEL_070370 | 156 | -31.67 | 2 | 18 | 160 | 182 | 17 | Query: 3' gagguGGAGGAGGC-GGAGGACc 5' | Ref: 5' gctttCTTGTTCTGTCCTCCTGg 3' |
| gga-miR-7475-5p | gi|619307639|gb|GAXD01012983.1| | XP_003708329 | dentin matrix protein 4-like | 158 | -33.93 | 2 | 19 | 2548 | 2567 | 17 | Query: 3' ccUCCCGCGCCGCCGCCGCc 5' | Ref: 5' tgAGGTCGAGCAGGCGGCGg 3' |
| hsa-miR-4459 | gi|619318742|gb|GAXD01002007.1| | XP_003708013 | microtubule-associated serine threonine-protein kinase 2 | 180 | -38.92 | 2 | 21 | 824 | 845 | 19 | Query: 3' gaGGUGGAGGAGGCGGAGGACc 5' | Ref: 5' ctCCTCCTTCCCCGCCTCCTGt 3' |
| hsa-miR-4459 | gi|619318695|gb|GAXD01002054.1| | XP_003707957 | dynein heavy cytoplasmic-like isoform 1 | 150 | -31.84 | 2 | 21 | 4604 | 4631 | 25 | Query: 3' gaGGUGG--AGGAGGC----GGAGGACc 5' | Ref: 5' aaCCACTTGTTCTTTGTGGTCCTCCTGg 3' |
| hsa-miR-4638-3p | gi|619294909|gb|GAXD01025229.1| | XP_003706536 | aplp_locmi ame: full=apolipophorins contains: ame: full=apolipophorin-2 ame: full=apolipophorin ii ame: full=apolp-2 contains: ame: full=apolipophorin-1 ame: full=apolipophorin i ame: full=apolp-1 flags: precursor | 164 | -36.32 | 2 | 18 | 8662 | 8686 | 18 | Query: 3' gccggcCGACUC--GCCACAGGUCc 5' | Ref: 5' aggataGGTGAGGTCGGTGTCCAGg 3' |
| hsa-miR-4638-3p | gi|619304279|gb|GAXD01016343.1| | XP_003705946 | ras-related protein rap-2c-like | 159 | -34.7 | 2 | 21 | 2427 | 2450 | 20 | Query: 3' gccGGCC-GACUCGCCACAGGUCc 5' | Ref: 5' tgtTCGGTCCCGGCTGTGTCCAGg 3' |
| mmu-miR-6240 | gi|619309121|gb|GAXD01011501.1| | XP_003705883 | PREDICTED: uncharacterized protein KIAA0564-like | 167 | -30.34 | 2 | 21 | 17 | 43 | 20 | Query: 3' gcggcaCCCGGAAGC-GCUACGAAACc 5' | Ref: 5' gatccaGGGCCTCAGACAATGCTTTGg 3' |
| gga-miR-7475-5p | gi|619319663|gb|GAXD01001086.1| | XP_003704678 | icarapin- partial | 164 | -35.62 | 2 | 17 | 386 | 405 | 15 | Query: 3' ccucCCGCGCCGCCGCCGCc 5' | Ref: 5' gtcaGGCGCATCGGCGGCGg 3' |
| gga-miR-7475-5p | gi|619319663|gb|GAXD01001086.1| | XP_003704678 | icarapin- partial | 163 | -33.28 | 2 | 17 | 295 | 315 | 16 | Query: 3' ccucCCGCGC-CGCCGCCGCc 5' | Ref: 5' aggcGGCTCGCGCGGCGGCGt 3' |
| gga-miR-7475-5p | gi|619319663|gb|GAXD01001086.1| | XP_003704678 | icarapin- partial | 155 | -36.01 | 2 | 17 | 397 | 417 | 16 | Query: 3' ccucCCG-CGCCGCCGCCGCc 5' | Ref: 5' cggcGGCGGCTTCGGCGGCGg 3' |
| gga-miR-7475-5p | gi|619319662|gb|GAXD01001087.1| | XP_003704678 | icarapin- partial | 164 | -35.62 | 2 | 17 | 386 | 405 | 15 | Query: 3' ccucCCGCGCCGCCGCCGCc 5' | Ref: 5' gtcaGGCGCATCGGCGGCGg 3' |
| gga-miR-7475-5p | gi|619319662|gb|GAXD01001087.1| | XP_003704678 | icarapin- partial | 163 | -33.28 | 2 | 17 | 295 | 315 | 16 | Query: 3' ccucCCGCGC-CGCCGCCGCc 5' | Ref: 5' aggcGGCTCGCGCGGCGGCGt 3' |
| gga-miR-7475-5p | gi|619319662|gb|GAXD01001087.1| | XP_003704678 | icarapin- partial | 155 | -36.01 | 2 | 17 | 397 | 417 | 16 | Query: 3' ccucCCG-CGCCGCCGCCGCc 5' | Ref: 5' cggcGGCGGCTTCGGCGGCGg 3' |
| gga-miR-7475-5p | gi|619299711|gb|GAXD01020910.1| | XP_003703997 | e3 ubiquitin-protein ligase ubr3-like | 168 | -41.2 | 2 | 19 | 364 | 386 | 20 | Query: 3' ccUCCCG--CG-CCGCCGCCGCc 5' | Ref: 5' gaAGGGCAAGCGGGCGGCGGCGg 3' |
| hsa-miR-4459 | gi|619299768|gb|GAXD01020853.1| | XP_003703256 | heat shock protein beta-1-like isoform 2 | 159 | -32.46 | 2 | 21 | 305 | 327 | 20 | Query: 3' gaGGUGGAGGAGGC-GGAGGACc 5' | Ref: 5' tcTTGTCGCCGCCGTCCTCCTGg 3' |
| mmu-miR-6990-5p | gi|619315985|gb|GAXD01004764.1| | XP_003702640 | adp-ribosylation factor-binding protein gga1-like | 173 | -30.63 | 2 | 20 | 87 | 106 | 18 | Query: 3' ucUCGGGACUGAGUGGGACCc 5' | Ref: 5' caAGCCC-GACTAACCCTGGt 3' |
| hsa-miR-4638-3p | gi|619300478|gb|GAXD01020144.1| | XP_003701602 | glutathione-s-transferase gst | 170 | -39.46 | 2 | 22 | 290 | 317 | 25 | Query: 3' gcCGGCC--GA-CUCG--CCACAGGUCc 5' | Ref: 5' ctGCCGGCCCTCGAGCATGGTGTCCAGg 3' |
| hsa-miR-4638-3p | gi|619300476|gb|GAXD01020146.1| | XP_003701602 | glutathione s-transferase e1 | 170 | -39.46 | 2 | 22 | 285 | 312 | 25 | Query: 3' gcCGGCC--GA-CUCG--CCACAGGUCc 5' | Ref: 5' ctGCCGGCCCTCGAGCATGGTGTCCAGg 3' |
| mmu-miR-5625-5p | gi|619314677|gb|GAXD01006072.1| | XP_003701552 | oxysterol-binding protein 9 | 167 | -30.3 | 2 | 18 | 432 | 451 | 16 | Query: 3' aggaUGAGUUCUUGAAGGCCc 5' | Ref: 5' ggaaAC-CAAGAGCTTCCGGg 3' |
| mmu-miR-5119 | gi|619300506|gb|GAXD01020116.1| | XP_003701391 | serine threonine-protein kinase wnk1 | 167 | -30.34 | 2 | 16 | 1696 | 1714 | 14 | Query: 3' ggucGGGGUCCUACUCUAc 5' | Ref: 5' ctctTCCCGGGATGAGATg 3' |
| ame-miR-3049-3p | gi|619318244|gb|GAXD01002505.1| | XP_003700418 | choline ethanolamine kinase-like | 167 | -30.72 | 2 | 19 | 2552 | 2573 | 18 | Query: 3' ucugCCUU-UCCUCAACCUGCCu 5' | Ref: 5' aagaGGAAGAGGA-TTGGACGGa 3' |
| hsa-miR-4638-3p | gi|619320196|gb|GAXD01000553.1| | XP_003700417 | PREDICTED: uncharacterized protein LOC100875144 | 152 | -30.95 | 2 | 18 | 544 | 567 | 17 | Query: 3' gccggcCGACUCGC-CACAGGUCc 5' | Ref: 5' tggtgaGGTGGGTGACTGTCCAGg 3' |
| hsa-miR-4638-3p | gi|619300287|gb|GAXD01020335.1| | XP_003700417 | PREDICTED: uncharacterized protein LOC100875144 | 152 | -30.95 | 2 | 18 | 544 | 567 | 17 | Query: 3' gccggcCGACUCGC-CACAGGUCc 5' | Ref: 5' tggtgaGGTGGGTGACTGTCCAGg 3' |
| hsa-miR-6787-5p | gi|619300026|gb|GAXD01020596.1| | XP_003699508 | 40s ribosomal protein s8-like | 157 | -31.66 | 2 | 21 | 481 | 504 | 22 | Query: 3' cgUCGGUCGAGAU---GGGGGCGGu 5' | Ref: 5' agAG-CAGTTCCAGACCCCCCGCCt 3' |
| mja-miR-6493-3p | gi|619319069|gb|GAXD01001680.1| | XP_003698962 | upstream-binding protein 1-like | 157 | -30.07 | 2 | 23 | 2512 | 2536 | 22 | Query: 3' auUG-CGAGUCGCGCCAAAGGGGGa 5' | Ref: 5' agACAGCTCGCAAGGGCTTCCCCCt 3' |
| mja-miR-6493-3p | gi|619305528|gb|GAXD01015094.1| | XP_003698455 | PREDICTED: UNC93-like protein-like | 164 | -30.65 | 2 | 23 | 3281 | 3306 | 23 | Query: 3' auUGCGAG-UCGCGCC-AAAGGGGGa 5' | Ref: 5' ggATGTTCTACTGGGGATTTCCCCCt 3' |
| mmu-miR-6990-5p | gi|619300094|gb|GAXD01020528.1| | XP_003696009 | PREDICTED: uncharacterized protein LOC100866111 | 166 | -32.63 | 2 | 20 | 458 | 480 | 20 | Query: 3' ucUCGGGAC--UGAGUGGGACCc 5' | Ref: 5' agAGCCCTGTCACAAACCCTGGt 3' |
| efu-miR-9198a | gi|619309062|gb|GAXD01011560.1| | XP_003695195 | PREDICTED: uncharacterized protein LOC100868132 | 173 | -33.09 | 2 | 22 | 198 | 220 | 20 | Query: 3' agUGUAGGUGACUGUCACGGUUc 5' | Ref: 5' gcACACACACTTTCAGTGCCAAg 3' |
| hsa-miR-6787-5p | gi|619296170|gb|GAXD01023968.1| | XP_003695139 | cklf-like marvel transmembrane domain-containing protein 4-like | 158 | -32.76 | 2 | 21 | 63 | 83 | 19 | Query: 3' cgUCGGUCGAGAUGGGGGCGGu 5' | Ref: 5' acAGCGAGTAC-GTCCCCGCCg 3' |
| gga-miR-7475-5p | gi|619309022|gb|GAXD01011600.1| | XP_003694796 | lysosomal acid phosphatase-like | 176 | -43.4 | 2 | 18 | 21 | 41 | 17 | Query: 3' ccuCCCGCGCC-GCCGCCGCc 5' | Ref: 5' ggcGGGCGCGGACGGCGGCGa 3' |
| gga-miR-7475-5p | gi|619309022|gb|GAXD01011600.1| | XP_003694796 | lysosomal acid phosphatase-like | 156 | -33.86 | 2 | 17 | 590 | 609 | 15 | Query: 3' ccucCCGCGCCGCCGCCGCc 5' | Ref: 5' tcctGGAGGTGCGGCGGCGc 3' |
| mja-miR-6493-5p | gi|619298052|gb|GAXD01022569.1| | XP_003694644 | e3 ubiquitin-protein ligase rnf8-a-like | 155 | -30.63 | 2 | 20 | 1874 | 1895 | 18 | Query: 3' uccCCAUUUUGGACGGCCUGCa 5' | Ref: 5' agtGGGGGTCCCAGCCGGACGc 3' |
| gga-miR-7475-5p | gi|619319646|gb|GAXD01001103.1| | XP_003694474 | PREDICTED: peroxidase-like | 159 | -30.05 | 2 | 16 | 753 | 772 | 14 | Query: 3' ccuccCGCGCCGCCGCCGCc 5' | Ref: 5' acgctGCACCGCGGCGGCGg 3' |
| gga-miR-7475-5p | gi|619319646|gb|GAXD01001103.1| | XP_003694474 | PREDICTED: peroxidase-like | 151 | -30.2 | 2 | 16 | 2749 | 2768 | 14 | Query: 3' ccuccCGCGCCGCCGCCGCc 5' | Ref: 5' accctGCGAGCAGGCGGCGg 3' |
| hsa-miR-6787-5p | gi|619319646|gb|GAXD01001103.1| | XP_003694474 | PREDICTED: peroxidase-like | 157 | -31.9 | 2 | 20 | 1306 | 1330 | 21 | Query: 3' cguCGGUCG-AGAUG--GGGGCGGu 5' | Ref: 5' tgcGCCCGCATCGACATCCCCGCCa 3' |
| hsa-miR-4459 | gi|619297593|gb|GAXD01023028.1| | XP_003692768 | e3 ubiquitin-protein ligase suppressor of | 158 | -30.96 | 2 | 19 | 470 | 491 | 17 | Query: 3' gaggUGGAGGAGGCGGAGGACc 5' | Ref: 5' atgaACTACCCCTTCCTCCTGg 3' |
| gga-miR-7475-5p | gi|619320331|gb|GAXD01000418.1| | XP_003689751 | transmembrane protease serine 9-like | 149 | -32.37 | 2 | 19 | 1531 | 1551 | 18 | Query: 3' ccUCCCG-CGCCGCCGCCGCc 5' | Ref: 5' ccAGGATGGTGAGGGCGGCGg 3' |
| mmu-miR-6240 | gi|619319624|gb|GAXD01001125.1| | XP_003641199 | probable atp-dependent rna helicase ddx60 isoform x1 | 177 | -30.16 | 2 | 25 | 3498 | 3521 | 23 | Query: 3' gcGGCACCCGGAAGCGCUACGAAACc 5' | Ref: 5' tcCCATGGGTCTT--AGATGCTTTGa 3' |
| ame-miR-3049-3p | gi|619317199|gb|GAXD01003550.1| | XP_003493563 | homeobox protein pknox2-like | 160 | -30.09 | 2 | 21 | 344 | 365 | 19 | Query: 3' ucUGCCUUUCCUCAACCUGCCu 5' | Ref: 5' ggACGACCCGGAGGTGGACGGa 3' |
| hsa-miR-4459 | gi|619318484|gb|GAXD01002265.1| | XP_003489882 | isoforms a c f g h-like isoform 2 | 170 | -33.79 | 2 | 19 | 2 | 23 | 17 | Query: 3' gaggUGGAGGAGGCGGAGGACc 5' | Ref: 5' cttgGCCTGCGCCGCCTCCTGc 3' |
| hsa-miR-4459 | gi|619295561|gb|GAXD01024577.1| | XP_003489013 | diacylglycerol kinase 1-like | 172 | -34.75 | 2 | 21 | 113 | 134 | 19 | Query: 3' gaGGUGGAGGAGGCGGAGGACc 5' | Ref: 5' gtCTGCTACCTCAGCCTCCTGg 3' |
| gga-miR-7475-5p | gi|619297695|gb|GAXD01022926.1| | XP_003488654 | low quality protein: cubilin-like | 153 | -30.85 | 2 | 15 | 405 | 425 | 14 | Query: 3' ccucccGCGCC-GCCGCCGCc 5' | Ref: 5' gttccaCTCGGACGGCGGCGg 3' |
| hsa-miR-6787-5p | gi|619320164|gb|GAXD01000585.1| | XP_003488426 | polyadenylate-binding protein 1-like isoform 1 | 155 | -33.49 | 2 | 21 | 1249 | 1274 | 23 | Query: 3' cgUCGGUCGAGAU----GGGGGCGGu 5' | Ref: 5' ccAGTCGGCCCAACAGGCCCCCGCCc 3' |
| hsa-miR-6787-5p | gi|619318381|gb|GAXD01002368.1| | XP_003488426 | polyadenylate-binding protein 1-like isoform 1 | 155 | -33.49 | 2 | 21 | 1714 | 1739 | 23 | Query: 3' cgUCGGUCGAGAU----GGGGGCGGu 5' | Ref: 5' ccAGTCGGCCCAACAGGCCCCCGCCc 3' |
| mmu-miR-6990-5p | gi|619297186|gb|GAXD01023435.1| | XP_003487942 | PREDICTED: hypothetical protein LOC100745406 | 163 | -30.08 | 2 | 20 | 287 | 307 | 18 | Query: 3' ucUCGGGACUGAGUGGGACCc 5' | Ref: 5' ggGGCTGGGCCTCACCCTGGt 3' |
| hsa-miR-6787-5p | gi|619298839|gb|GAXD01021782.1| | XP_003486835 | carboxy-terminal domain rna polymerase ii polypeptide a small phosphatase 1-like | 172 | -33.6 | 2 | 21 | 1587 | 1608 | 19 | Query: 3' cgUCGGUCGAGAUGGGGGCGGu 5' | Ref: 5' taAGCCGGCACAAGCCCCGCCa 3' |
| hsa-miR-4459 | gi|619294403|gb|GAXD01025735.1| | XP_003484849 | mannosyl-oligosaccharide glucosidase-like isoform 1 | 170 | -41.72 | 2 | 21 | 994 | 1018 | 22 | Query: 3' gaGGUGGAGGA--GGC-GGAGGACc 5' | Ref: 5' atTCGCCTCCTCGCCGTCCTCCTGg 3' |
| gga-miR-7475-5p | gi|619309052|gb|GAXD01011570.1| | XP_003436059 | inositol-trisphosphate 3-kinase b-like isoform 2 | 155 | -30.29 | 2 | 19 | 1435 | 1454 | 18 | Query: 3' ccUCCCGCGCCG-CCGCCGCc 5' | Ref: 5' tcGGGGC-TGGTGGGCGGCGa 3' |
| bmo-miR-3344 | gi|619309052|gb|GAXD01011570.1| | XP_003436059 | inositol-trisphosphate 3-kinase b-like isoform 2 | 165 | -30.06 | 2 | 22 | 2183 | 2207 | 20 | Query: 3' gagcGACCGACUCAGGAAGAACGUu 5' | Ref: 5' tgaaCTCGCTGAGGAGGTCTTGCAa 3' |
| mja-miR-6493-3p | gi|619297390|gb|GAXD01023231.1| | XP_003427147 | choline-phosphate cytidylyltransferase b-like isoform 2 | 179 | -35.24 | 2 | 22 | 1206 | 1228 | 20 | Query: 3' auuGCGAGUCGCGCCAAAGGGGGa 5' | Ref: 5' tgcTGCTCTGCGC-GTTTCCCCCg 3' |
| gga-miR-7475-5p | gi|619295142|gb|GAXD01024996.1| | XP_003427127 | muscleblind cg33197-pa | 156 | -32.71 | 2 | 14 | 660 | 680 | 13 | Query: 3' ccucccgCGCC-GCCGCCGCc 5' | Ref: 5' cctacaaGCGGCCGGCGGCGg 3' |
| hsa-miR-6787-5p | gi|619306826|gb|GAXD01013796.1| | XP_003427112 | 5-aminolevulinate erythroid- mitochondrial-like isoform 2 | 159 | -32.56 | 2 | 21 | 61 | 83 | 20 | Query: 3' cgUCGGUCGAGAUG-GGGGCGGu 5' | Ref: 5' ccGGCCGTCCCTTCACCCCGCCa 3' |
| hsa-miR-4638-3p | gi|619314176|gb|GAXD01006573.1| | XP_003425573 | prominin-like isoform 3 | 158 | -30.14 | 2 | 17 | 316 | 337 | 15 | Query: 3' gccggccGACUCGCCACAGGUCc 5' | Ref: 5' gtcctccCTGAG-AGTGTCCAGg 3' |
| hsa-miR-4638-3p | gi|619300391|gb|GAXD01020231.1| | XP_003401965 | amp deaminase 2-like isoform 1 | 167 | -34.89 | 2 | 21 | 2875 | 2898 | 20 | Query: 3' gccGGC-CGACUCGCCACAGGUCc 5' | Ref: 5' tccTCGTGCTTGACGGTGTCCAGg 3' |
| bmo-miR-3344 | gi|619298502|gb|GAXD01022119.1| | XP_003399863 | receptor-type tyrosine-protein phosphatase r-like | 174 | -31.43 | 2 | 21 | 5947 | 5970 | 19 | Query: 3' gagcgACCGACUCAGGAAGAACGUu 5' | Ref: 5' agttgTGGCTGAGT-TGTCTTGCAa 3' |
| hsa-miR-4638-3p | gi|619300293|gb|GAXD01020329.1| | XP_003399734 | PREDICTED: hypothetical protein LOC100647311 | 152 | -30.95 | 2 | 18 | 544 | 567 | 17 | Query: 3' gccggcCGACUCGC-CACAGGUCc 5' | Ref: 5' tggtgaGGTGGGTGACTGTCCAGg 3' |
| hsa-miR-4638-3p | gi|619300289|gb|GAXD01020333.1| | XP_003399734 | PREDICTED: hypothetical protein LOC100647311 | 152 | -30.95 | 2 | 18 | 544 | 567 | 17 | Query: 3' gccggcCGACUCGC-CACAGGUCc 5' | Ref: 5' tggtgaGGTGGGTGACTGTCCAGg 3' |
| mja-miR-6493-3p | gi|619318910|gb|GAXD01001839.1| | XP_003399532 | protein transport protein sec24c-like | 158 | -30.05 | 2 | 23 | 5485 | 5511 | 25 | Query: 3' auUGCGAGUC---GCG-CCAAAGGGGGa 5' | Ref: 5' aaAC-CTTGGACCTGCAGGTTTCCCCCc 3' |
| hsa-miR-4459 | gi|619296141|gb|GAXD01023997.1| | XP_003399087 | e3 ubiquitin-protein ligase hyd-like | 155 | -30.14 | 2 | 20 | 1042 | 1063 | 18 | Query: 3' gagGUGGAGGAGGCGGAGGACc 5' | Ref: 5' ccaCAGGTCCTAATCCTCCTGg 3' |
| hsa-miR-4459 | gi|619319533|gb|GAXD01001216.1| | XP_003399071 | regulator of nonsense transcripts 1-like isoform 1 | 151 | -32.18 | 2 | 21 | 1545 | 1570 | 23 | Query: 3' gaGGUGGAGGAGG----CGGAGGACc 5' | Ref: 5' ctCTCCCTTATTCAAGGGCCTCCTGg 3' |
| efu-miR-9198a | gi|619317629|gb|GAXD01003120.1| | XP_003398944 | golgin subfamily a member 4 | 165 | -30.71 | 2 | 22 | 611 | 633 | 22 | Query: 3' agUGUAG--GUGACUGUCACGGUUc 5' | Ref: 5' gcATATCCTCACTG--AGTGCCAAg 3' |
| efu-miR-9198a | gi|619299333|gb|GAXD01021288.1| | XP_003398944 | golgin subfamily a member 4 | 165 | -30.71 | 2 | 22 | 698 | 720 | 22 | Query: 3' agUGUAG--GUGACUGUCACGGUUc 5' | Ref: 5' gcATATCCTCACTG--AGTGCCAAg 3' |
| mja-miR-6493-3p | gi|619299858|gb|GAXD01020764.1| | XP_003398290 | pumilio homolog 1-like | 167 | -30.14 | 2 | 21 | 5155 | 5179 | 20 | Query: 3' auugCGAGUCG-CGCCAAAGGGGGa 5' | Ref: 5' cataGCTCTGCGGCCATTTCCCCCt 3' |
| mmu-miR-6990-5p | gi|619319238|gb|GAXD01001511.1| | XP_003397476 | myosin heavy muscle-like isoform 1 | 167 | -30.09 | 2 | 18 | 364 | 383 | 16 | Query: 3' ucucGGGACUGAGUGGGACCc 5' | Ref: 5' tcttCCTTGAC-CACCCTGGc 3' |
| gga-miR-7475-5p | gi|619301231|gb|GAXD01019391.1| | XP_003396248 | PREDICTED: hypothetical protein LOC100645196 | 160 | -32.59 | 2 | 18 | 204 | 224 | 17 | Query: 3' ccuCCCG-CGCCGCCGCCGCc 5' | Ref: 5' gtcGGACAGCGACGGCGGCGg 3' |
| hsa-miR-6787-5p | gi|619308919|gb|GAXD01011703.1| | XP_003377509 | reverse transcriptase family protein | 144 | -31.39 | 2 | 20 | 125 | 144 | 18 | Query: 3' cguCGGUCGAGAUGGGGGCGGu 5' | Ref: 5' gctGCCAG--AGGACCCCGCCa 3' |
| hsa-miR-6787-5p | gi|619292226|gb|GAXD01027912.1| | XP_003249835 | kinesin 3a | 154 | -31.36 | 2 | 21 | 100 | 124 | 22 | Query: 3' cgUCGGU--CG-AGAUGGGGGCGGu 5' | Ref: 5' acAGCAAGGGCATCATCCCCCGCCt 3' |
| efu-miR-9198a | gi|619320456|gb|GAXD01000293.1| | XP_003246504 | PREDICTED: hypothetical protein LOC100164801 isoform 2 | 163 | -30.26 | 2 | 22 | 161 | 182 | 20 | Query: 3' agUGUAGGUGACUGUCACGGUUc 5' | Ref: 5' gcAGGTGCCCTGAC-GTGCCAAg 3' |
| hsa-miR-6787-5p | gi|619320456|gb|GAXD01000293.1| | XP_003246504 | PREDICTED: hypothetical protein LOC100164801 isoform 2 | 156 | -31.84 | 2 | 19 | 396 | 416 | 17 | Query: 3' cgucGGUCGAGAUGGGGGCGGu 5' | Ref: 5' tccaCCGCATCT-CCCCCGCCg 3' |
| hsa-miR-4459 | gi|619306310|gb|GAXD01014312.1| | XP_003245566 | PREDICTED: spondin-2-like | 162 | -34.26 | 2 | 20 | 307 | 330 | 20 | Query: 3' gagGUGG--AGGAGGCGGAGGACc 5' | Ref: 5' ttgCACCCGTTGACCGCCTCCTGg 3' |
| hsa-miR-4459 | gi|619309782|gb|GAXD01010840.1| | XP_003245479 | PREDICTED: hypothetical protein LOC100570013 | 168 | -30.23 | 2 | 21 | 490 | 511 | 19 | Query: 3' gaGGUGGAGGAGGCGGAGGACc 5' | Ref: 5' gtCCACCAACACCACCTCCTGc 3' |
| hsa-miR-6787-5p | gi|619294081|gb|GAXD01026057.1| | XP_003244050 | pancreatic lipase-related protein 1-like isoform 2 | 163 | -32.38 | 2 | 20 | 206 | 227 | 18 | Query: 3' cguCGGUCGAGAUGGGGGCGGu 5' | Ref: 5' gccGTCGGGCCCACCCCCGCCa 3' |
| hsa-miR-6787-5p | gi|619294080|gb|GAXD01026058.1| | XP_003244050 | pancreatic lipase-related protein 1-like isoform 2 | 163 | -32.38 | 2 | 20 | 206 | 227 | 18 | Query: 3' cguCGGUCGAGAUGGGGGCGGu 5' | Ref: 5' gccGTCGGGCCCACCCCCGCCa 3' |
| hsa-miR-6787-5p | gi|619294078|gb|GAXD01026060.1| | XP_003244050 | pancreatic lipase-related protein 1-like isoform 2 | 163 | -32.38 | 2 | 20 | 206 | 227 | 18 | Query: 3' cguCGGUCGAGAUGGGGGCGGu 5' | Ref: 5' gccGTCGGGCCCACCCCCGCCa 3' |
| gga-miR-7475-5p | gi|619312461|gb|GAXD01008288.1| | XP_002741225 | ribosomal protein s9-like | 169 | -42.22 | 2 | 19 | 370 | 390 | 18 | Query: 3' ccUCCC-GCGCCGCCGCCGCc 5' | Ref: 5' tcAGGGAGGTGGCGGCGGCGg 3' |
| hsa-miR-6787-5p | gi|619294431|gb|GAXD01025707.1| | XP_002737197 | hop homeobox-like | 167 | -34.74 | 2 | 21 | 186 | 208 | 20 | Query: 3' cgUCGGUCGAGAU-GGGGGCGGu 5' | Ref: 5' gaAGTCGGCGCAAGCCCCCGCCg 3' |
| gga-miR-7475-5p | gi|619295775|gb|GAXD01024363.1| | XP_002613715 | hypothetical protein BRAFLDRAFT_104160 | 143 | -31.16 | 2 | 14 | 89 | 107 | 12 | Query: 3' ccucccgCGCCGCCGCCGCc 5' | Ref: 5' actgcagGAGG-GGCGGCGg 3' |
| gga-miR-7475-5p | gi|619307745|gb|GAXD01012877.1| | XP_002587832 | hypothetical protein BRAFLDRAFT_143543 | 158 | -32.61 | 2 | 17 | 295 | 317 | 18 | Query: 3' ccucCCGCGC--CG-CCGCCGCc 5' | Ref: 5' gcgcGGCGCGGAGCGGGCGGCGc 3' |
| efu-miR-9198a | gi|619298518|gb|GAXD01022103.1| | XP_002432920 | elongation factor 1- | 173 | -30.48 | 2 | 18 | 353 | 375 | 16 | Query: 3' aguguaGGUGACUGUCACGGUUc 5' | Ref: 5' aagttgCCACTGATCGTGCCAAg 3' |
| gga-miR-7475-5p | gi|619295105|gb|GAXD01025033.1| | XP_002432901 | conserved hypothetical protein | 174 | -41.7 | 2 | 19 | 126 | 145 | 17 | Query: 3' ccUCCCGCGCCGCCGCCGCc 5' | Ref: 5' ccGGGGCCTGGCGGCGGCGg 3' |
| hsa-miR-6787-5p | gi|619312815|gb|GAXD01007934.1| | XP_002432036 | fatty acid | 160 | -30.23 | 2 | 21 | 428 | 449 | 19 | Query: 3' cgUCGGUCGAGAUGGGGGCGGu 5' | Ref: 5' gaGGCTGGCGTCAACCCCGCCg 3' |
| hsa-miR-6787-5p | gi|619291737|gb|GAXD01028401.1| | XP_002431484 | tyrosine-protein phosphatase 10d | 179 | -34.85 | 2 | 20 | 247 | 268 | 18 | Query: 3' cguCGGUCGAGAUGGGGGCGGu 5' | Ref: 5' gatGCCAATTTTACCCCCGCCt 3' |
| mmu-miR-6990-5p | gi|619299999|gb|GAXD01020623.1| | XP_002431217 | signal recognition particle 72 kda | 158 | -30.07 | 2 | 20 | 899 | 920 | 19 | Query: 3' ucUCGGGACUGA-GUGGGACCc 5' | Ref: 5' gcACCCGTAGCTGCACCCTGGg 3' |
| ssa-miR-15a-5p | gi|619309756|gb|GAXD01010866.1| | XP_002429512 | cg1347 cg1347-pa | 174 | -31.86 | 2 | 22 | 451 | 471 | 20 | Query: 3' ugUUUGGUACUGCACGACGAUGc 5' | Ref: 5' acAAGCCGTGAC--GCTGCTACc 3' |
| gga-miR-7475-5p | gi|619314650|gb|GAXD01006099.1| | XP_002429421 | actin-binding lim protein 1-like | 160 | -42.43 | 2 | 19 | 29 | 52 | 21 | Query: 3' ccUCCCGCGC-CG---CCGCCGCc 5' | Ref: 5' cgGGGGCGCGAGCCTGGGCGGCGg 3' |
| gga-miR-7475-5p | gi|619294689|gb|GAXD01025449.1| | XP_002428965 | aminopeptidase n | 146 | -34.4 | 2 | 17 | 98 | 122 | 20 | Query: 3' ccucCCG-CGCC----GCCGCCGCc 5' | Ref: 5' atgtGGCAGCGGTACCTGGCGGCGg 3' |
| hsa-miR-4459 | gi|619300205|gb|GAXD01020417.1| | XP_002428414 | herpud family member 2 | 163 | -31.77 | 2 | 20 | 1421 | 1442 | 18 | Query: 3' gagGUGGAGGAGGCGGAGGACc 5' | Ref: 5' ctgTACCCACTGTGCCTCCTGc 3' |
| hsa-miR-4459 | gi|619318887|gb|GAXD01001862.1| | XP_002428406 | conserved hypothetical protein | 172 | -31.69 | 2 | 21 | 684 | 705 | 19 | Query: 3' gaGGUGGAGGAGGCGGAGGACc 5' | Ref: 5' aaCCACCACTCCCTCCTCCTGa 3' |
| gga-miR-7475-5p | gi|619297012|gb|GAXD01023609.1| | XP_002428231 | repulsive guidance molecule a-like isoform 2 | 156 | -32.08 | 2 | 18 | 91 | 111 | 17 | Query: 3' ccuCCCGCGCC-GCCGCCGCc 5' | Ref: 5' ggtGGAGGTGGACGGCGGCGa 3' |
| mja-miR-6493-5p | gi|619307738|gb|GAXD01012884.1| | XP_002428182 | hypothetical protein Phum_PHUM368740 | 167 | -33.45 | 2 | 21 | 228 | 251 | 21 | Query: 3' ucCCCAUUUU--GGACGGCCUGCa 5' | Ref: 5' aaGGGTGGAGCTCCGGCCGGACGa 3' |
| hsa-miR-4459 | gi|619318602|gb|GAXD01002147.1| | XP_002427383 | Peroxiredoxin-6, putative | 151 | -30.08 | 2 | 19 | 254 | 275 | 18 | Query: 3' gaggUGGAGGAGGC-GGAGGACc 5' | Ref: 5' cgggACCT-GGCCGTCCTCCTGg 3' |
| gga-miR-1723 | gi|619300311|gb|GAXD01020311.1| | XP_002426207 | transformer-2 sex-determining | 159 | -31.14 | 2 | 22 | 541 | 562 | 20 | Query: 3' acUCCGACGUGUAAGGCGAGGGu 5' | Ref: 5' cgAGGTCCCGC-TCCCGCTCCCg 3' |
| gga-miR-1723 | gi|619300310|gb|GAXD01020312.1| | XP_002426207 | transformer-2 sex-determining | 159 | -31.14 | 2 | 22 | 541 | 562 | 20 | Query: 3' acUCCGACGUGUAAGGCGAGGGu 5' | Ref: 5' cgAGGTCCCGC-TCCCGCTCCCg 3' |
| hsa-miR-6787-5p | gi|619319820|gb|GAXD01000929.1| | XP_002426198 | oxysterol binding protein | 161 | -31.25 | 2 | 20 | 1226 | 1246 | 18 | Query: 3' cguCGGUCGAGAUGGGGGCGGu 5' | Ref: 5' agtGGCA-CTGTATCCCCGCCa 3' |
| hsa-miR-6787-5p | gi|619314730|gb|GAXD01006019.1| | XP_002426198 | oxysterol binding protein | 161 | -31.25 | 2 | 20 | 1226 | 1246 | 18 | Query: 3' cguCGGUCGAGAUGGGGGCGGu 5' | Ref: 5' agtGGCA-CTGTATCCCCGCCa 3' |
| hsa-miR-4459 | gi|619318417|gb|GAXD01002332.1| | XP_002426163 | conserved hypothetical protein | 169 | -37.67 | 2 | 21 | 944 | 965 | 20 | Query: 3' gaGGU-GGAGGAGGCGGAGGACc 5' | Ref: 5' tgCCATCCTCCTTT-CCTCCTGg 3' |
| mmu-miR-6240 | gi|619306335|gb|GAXD01014287.1| | XP_002426121 | transcription factor kayak-like | 166 | -30.95 | 2 | 24 | 352 | 379 | 24 | Query: 3' gcgGCACCCGGAAGCGCU--ACGAAACc 5' | Ref: 5' tgaCGTCGGTCTGGGTGGTTTGCTTTGg 3' |
| hsa-miR-6787-5p | gi|619299268|gb|GAXD01021353.1| | XP_002426032 | kh domain-containing protein | 162 | -31.72 | 2 | 21 | 3091 | 3114 | 21 | Query: 3' cgUC-GGUCGAGAU-GGGGGCGGu 5' | Ref: 5' agAGTCCCTCTCTGCCCCCCGCCt 3' |
| hsa-miR-6787-5p | gi|619299267|gb|GAXD01021354.1| | XP_002426032 | kh domain-containing protein | 162 | -31.72 | 2 | 21 | 3011 | 3034 | 21 | Query: 3' cgUC-GGUCGAGAU-GGGGGCGGu 5' | Ref: 5' agAGTCCCTCTCTGCCCCCCGCCt 3' |
| hsa-miR-6787-5p | gi|619299266|gb|GAXD01021355.1| | XP_002426032 | kh domain-containing protein | 162 | -31.72 | 2 | 21 | 3011 | 3034 | 21 | Query: 3' cgUC-GGUCGAGAU-GGGGGCGGu 5' | Ref: 5' agAGTCCCTCTCTGCCCCCCGCCt 3' |
| hsa-miR-6787-5p | gi|619299265|gb|GAXD01021356.1| | XP_002426032 | kh domain-containing protein | 162 | -31.72 | 2 | 21 | 3011 | 3034 | 21 | Query: 3' cgUC-GGUCGAGAU-GGGGGCGGu 5' | Ref: 5' agAGTCCCTCTCTGCCCCCCGCCt 3' |
| hsa-miR-4459 | gi|619299267|gb|GAXD01021354.1| | XP_002426032 | kh domain-containing protein | 162 | -33.14 | 2 | 20 | 3407 | 3430 | 20 | Query: 3' gagGUGGAGGAG--GCGGAGGACc 5' | Ref: 5' tggTACTTCCACAATGCCTCCTGg 3' |
| hsa-miR-4459 | gi|619299266|gb|GAXD01021355.1| | XP_002426032 | kh domain-containing protein | 162 | -33.14 | 2 | 20 | 3407 | 3430 | 20 | Query: 3' gagGUGGAGGAG--GCGGAGGACc 5' | Ref: 5' tggTACTTCCACAATGCCTCCTGg 3' |
| hsa-miR-4459 | gi|619299265|gb|GAXD01021356.1| | XP_002426032 | kh domain-containing protein | 162 | -33.14 | 2 | 20 | 3407 | 3430 | 20 | Query: 3' gagGUGGAGGAG--GCGGAGGACc 5' | Ref: 5' tggTACTTCCACAATGCCTCCTGg 3' |
| gga-miR-7475-5p | gi|619300331|gb|GAXD01020291.1| | XP_002425888 | serine threonine-protein phosphatase 2a 56 kda regulatory subunit delta | 160 | -37 | 2 | 18 | 4573 | 4593 | 17 | Query: 3' ccuCCCG-CGCCGCCGCCGCc 5' | Ref: 5' gatGGGCACCGGGGGCGGCGg 3' |
| gga-miR-7475-5p | gi|619300330|gb|GAXD01020292.1| | XP_002425888 | serine threonine-protein phosphatase 2a 56 kda regulatory subunit delta | 160 | -37 | 2 | 18 | 4548 | 4568 | 17 | Query: 3' ccuCCCG-CGCCGCCGCCGCc 5' | Ref: 5' gatGGGCACCGGGGGCGGCGg 3' |
| hsa-miR-4459 | gi|619312198|gb|GAXD01008551.1| | XP_002425652 | large proline-rich protein bat2 | 170 | -33.01 | 2 | 21 | 542 | 562 | 19 | Query: 3' gaGGUGGAGGAGGCGGAGGACc 5' | Ref: 5' gcCTGCC-CCTCCTCCTCCTGt 3' |
| gga-miR-7475-5p | gi|619318144|gb|GAXD01002605.1| | XP_002425584 | carbohydrate sulfotransferase 5 | 161 | -35.44 | 2 | 19 | 131 | 151 | 18 | Query: 3' ccUCCCG-CGCCGCCGCCGCc 5' | Ref: 5' gtGGGGCGGCGCGGGCGGCGc 3' |
| gga-miR-7475-5p | gi|619312043|gb|GAXD01008706.1| | XP_002425584 | carbohydrate sulfotransferase 5 | 161 | -35.44 | 2 | 19 | 131 | 151 | 18 | Query: 3' ccUCCCG-CGCCGCCGCCGCc 5' | Ref: 5' gtGGGGCGGCGCGGGCGGCGc 3' |
| efu-miR-9198a | gi|619297991|gb|GAXD01022630.1| | XP_002425460 | 60s ribosomal protein | 163 | -32.47 | 2 | 22 | 536 | 564 | 26 | Query: 3' agUGUAGG-----UGACUGU-CACGGUUc 5' | Ref: 5' gtGCATCCAACAGACTGGCATGTGCCAAg 3' |
| gga-miR-7475-5p | gi|619299249|gb|GAXD01021372.1| | XP_002425087 | cg10990 cg10990-pb | 155 | -31.73 | 2 | 19 | 379 | 396 | 17 | Query: 3' ccUCCCGCGCCGCCGCCGCc 5' | Ref: 5' ggGGGGCCCG--GGCGGCGt 3' |
| gga-miR-7475-5p | gi|619299248|gb|GAXD01021373.1| | XP_002425087 | cg10990 cg10990-pb | 155 | -31.73 | 2 | 19 | 379 | 396 | 17 | Query: 3' ccUCCCGCGCCGCCGCCGCc 5' | Ref: 5' ggGGGGCCCG--GGCGGCGt 3' |
| hsa-miR-4459 | gi|619301202|gb|GAXD01019420.1| | XP_002424891 | slit-robo rho gtpase-activating | 160 | -30.94 | 2 | 21 | 395 | 416 | 19 | Query: 3' gaGGUGGAGGAGGCGGAGGACc 5' | Ref: 5' ccCCTCCAACCCGGCCTCCTGa 3' |
| hsa-miR-6787-5p | gi|619319227|gb|GAXD01001522.1| | XP_002424807 | hect e3 ubiquitin | 158 | -37 | 2 | 21 | 9 | 33 | 22 | Query: 3' cgUCGGU-CGAGAU--GGGGGCGGu 5' | Ref: 5' gcGGCCATCCTCCAGTCCCCCGCCg 3' |
| hsa-miR-6787-5p | gi|619313838|gb|GAXD01006911.1| | XP_002424807 | hect e3 ubiquitin | 158 | -37 | 2 | 21 | 9 | 33 | 22 | Query: 3' cgUCGGU-CGAGAU--GGGGGCGGu 5' | Ref: 5' gcGGCCATCCTCCAGTCCCCCGCCg 3' |
| gga-miR-1723 | gi|619305660|gb|GAXD01014962.1| | XP_002424541 | endothelin-converting enzyme | 147 | -30.13 | 2 | 22 | 535 | 556 | 20 | Query: 3' acUCCGACGUGUAAGGCGAGGGu 5' | Ref: 5' ccAGGGGGGGC-TGACGCTCCCg 3' |
| hsa-miR-4459 | gi|619289680|gb|GAXD01030458.1| | XP_002424120 | conserved hypothetical protein | 171 | -31.65 | 2 | 20 | 138 | 159 | 18 | Query: 3' gagGUGGAGGAGGCGGAGGACc 5' | Ref: 5' cagCTCCTCCACCTCCTCCTGc 3' |
| hsa-miR-4459 | gi|619294508|gb|GAXD01025630.1| | XP_002423659 | conserved hypothetical protein | 157 | -31.67 | 2 | 14 | 164 | 185 | 12 | Query: 3' gagguggagGAGGCGGAGGACc 5' | Ref: 5' gctgtgcggCGCCGCCTCCTGg 3' |
| hsa-miR-6787-5p | gi|619307022|gb|GAXD01013600.1| | XP_002423408 | torso-like protein | 154 | -32.06 | 2 | 21 | 241 | 261 | 19 | Query: 3' cgUCGGUCGAGAUGGGGGCGGu 5' | Ref: 5' gcGGTC-GCATGGCCCCCGCCc 3' |
| gga-miR-7475-5p | gi|619297953|gb|GAXD01022668.1| | XP_002402316 | alpha- -mannosyl-glycoprotein beta- -n- | 148 | -30.43 | 2 | 17 | 75 | 94 | 15 | Query: 3' ccucCCGCGCCGCCGCCGCc 5' | Ref: 5' ccgaGGAGAACCGGCGGCGg 3' |
| mja-miR-6489-5p | gi|619293840|gb|GAXD01026298.1| | XP_002015931 | GL10763 | 162 | -30.58 | 2 | 19 | 191 | 210 | 17 | Query: 3' uuCCCGCGGUCAGGCCACGg 5' | Ref: 5' gaGGCCCCCGGCCCGGTGCc 3' |
| hsa-miR-4638-3p | gi|619319072|gb|GAXD01001677.1| | XP_001966149 | GF19520 | 164 | -30.09 | 2 | 18 | 595 | 618 | 17 | Query: 3' gccggcCGACUCGC-CACAGGUCc 5' | Ref: 5' actagtGCTCAGTGTGTGTCCAGg 3' |
| hsa-miR-4459 | gi|619295715|gb|GAXD01024423.1| | XP_001965383 | phospholipase c at isoform g | 174 | -30.01 | 2 | 19 | 677 | 698 | 17 | Query: 3' gaggUGGAGGAGGCGGAGGACc 5' | Ref: 5' gatgAACTCCTCCCCCTCCTGc 3' |
| hsa-miR-6787-5p | gi|619315752|gb|GAXD01004997.1| | XP_001962583 | GF14366 | 161 | -32.97 | 2 | 20 | 45 | 65 | 18 | Query: 3' cguCGGUCGAGAUGGGGGCGGu 5' | Ref: 5' ggcGTCAATTC-ATCCCCGCCg 3' |
| hsa-miR-6787-5p | gi|619320733|gb|GAXD01000016.1| | XP_001959844 | aquaporin -like | 167 | -33.68 | 2 | 20 | 1132 | 1153 | 18 | Query: 3' cguCGGUCGAGAUGGGGGCGGu 5' | Ref: 5' ggcGCCAGCCTCAACCCCGCCc 3' |
| hsa-miR-4459 | gi|619293205|gb|GAXD01026933.1| | XP_001950798 | PREDICTED: hypothetical protein LOC100165401 | 162 | -32.74 | 2 | 21 | 65 | 85 | 19 | Query: 3' gaGGUGGAGGAGGCGGAGGACc 5' | Ref: 5' ggTCGCCGCC-CAGCCTCCTGg 3' |
| hsa-miR-4459 | gi|619305062|gb|GAXD01015560.1| | XP_001950530 | laminin subunit alpha-1-like | 149 | -31.85 | 2 | 20 | 1 | 22 | 21 | Query: 3' gagGUGGA--GGAG-GCGGAGGACc 5' | Ref: 5' ---TACCTCGCCGCTTACCTCCTGg 3' |
| hsa-miR-6787-5p | gi|619307759|gb|GAXD01012863.1| | XP_001949462 | proto-oncogene tyrosine-protein kinase | 162 | -30.95 | 2 | 21 | 24 | 47 | 21 | Query: 3' cgUCGGUCGA-GAUG-GGGGCGGu 5' | Ref: 5' tcACCCAGTTGCTGTACCCCGCCc 3' |
| gga-miR-7475-5p | gi|619298464|gb|GAXD01022157.1| | XP_001945039 | PREDICTED: hypothetical protein LOC100163234 | 170 | -39.33 | 2 | 19 | 1809 | 1828 | 17 | Query: 3' ccUCCCGCGCCGCCGCCGCc 5' | Ref: 5' ggGGAGCGCGGGGGCGGCGt 3' |
| hsa-miR-4459 | gi|619295504|gb|GAXD01024634.1| | XP_001944998 | protein spire-like | 170 | -36.49 | 2 | 21 | 1 | 21 | 19 | Query: 3' gaGGUGGAGGAGGCGGAGGACc 5' | Ref: 5' cgCCG-CTCTTCCTCCTCCTGg 3' |
| ame-miR-3049-3p | gi|619298186|gb|GAXD01022435.1| | XP_001944877 | PREDICTED: afadin-like | 155 | -31.42 | 2 | 21 | 1366 | 1390 | 22 | Query: 3' ucUGCCU---UUCCUCAACCUGCCu 5' | Ref: 5' ggATGGACTGGAGGGCATGGACGGc 3' |
| hsa-miR-4638-3p | gi|619299095|gb|GAXD01021526.1| | XP_001944504 | facilitated trehalose transporter tret1-like | 160 | -30.59 | 2 | 22 | 3646 | 3669 | 21 | Query: 3' gcCG-GCCGACUCGCCACAGGUCc 5' | Ref: 5' tcGCGCGCCTTGACGATGTCCAGg 3' |
| gga-miR-7475-5p | gi|619299293|gb|GAXD01021328.1| | XP_001944498 | 6-phosphofructo-2-kinase fructose- - | 163 | -30.14 | 2 | 16 | 722 | 741 | 14 | Query: 3' ccuccCGCGCCGCCGCCGCc 5' | Ref: 5' gaccaGCATGGCGGCGGCGc 3' |
| hsa-miR-4459 | gi|619313324|gb|GAXD01007425.1| | XP_001942650 | thioredoxin reductase mitochondrial-like | 166 | -32.28 | 2 | 21 | 1030 | 1050 | 19 | Query: 3' gaGGUGGAGGAGGCGGAGGACc 5' | Ref: 5' taTCATCTCC-ATGCCTCCTGa 3' |
| hsa-miR-6787-5p | gi|619295106|gb|GAXD01025032.1| | XP_001942514 | atp-binding cassette sub-family g member 1-like | 161 | -30.71 | 2 | 20 | 347 | 370 | 20 | Query: 3' cguCG-GUCGA-GAUGGGGGCGGu 5' | Ref: 5' ctcGCACATCTCCTGTCCCCGCCa 3' |
| gga-miR-7475-5p | gi|619305131|gb|GAXD01015491.1| | XP_001866179 | juvenile hormone-inducible protein | 160 | -33.22 | 2 | 19 | 239 | 260 | 19 | Query: 3' ccUCCCGCG-CCG-CCGCCGCc 5' | Ref: 5' cgAGGTCGTGGGCGGGCGGCGa 3' |
| mmu-miR-6240 | gi|619316919|gb|GAXD01003830.1| | XP_001847295 | zinc finger protein | 153 | -30.32 | 2 | 25 | 235 | 256 | 23 | Query: 3' gcGGCACCCGGAAGCGCUACGAAACc 5' | Ref: 5' atCCG-GGTCC--AGCG-TGCTTTGg 3' |
| gga-miR-7475-5p | gi|619320561|gb|GAXD01000188.1| | XP_001844610 | kakapo | 164 | -33.3 | 2 | 19 | 51 | 69 | 17 | Query: 3' ccUCCCGCGCCGCCGCCGCc 5' | Ref: 5' cgAGGCCGT-GCGGCGGCGc 3' |
| mmu-miR-6990-5p | gi|619315605|gb|GAXD01005144.1| | XP_001814083 | myosin heavy chain cg17927-pf isoform 6 | 167 | -30.09 | 2 | 18 | 364 | 383 | 16 | Query: 3' ucucGGGACUGAGUGGGACCc 5' | Ref: 5' tcttCCTTGAC-CACCCTGGc 3' |
| gga-miR-7475-5p | gi|619320316|gb|GAXD01000433.1| | XP_001813711 | acetyl-coenzyme a synthetase | 155 | -34.76 | 2 | 17 | 1175 | 1196 | 17 | Query: 3' ccucCCGCGCCG--CCGCCGCc 5' | Ref: 5' ttgaGGTCCGGCAGGGCGGCGg 3' |
| hsa-miR-4459 | gi|619317637|gb|GAXD01003112.1| | XP_001811380 | multisynthetase auxiliary p38 | 155 | -30.62 | 2 | 18 | 477 | 500 | 18 | Query: 3' gagguGGAGG-A-GGCGGAGGACc 5' | Ref: 5' aaaacCCTCCATACTCCCTCCTGg 3' |
| gga-miR-7475-5p | gi|619317973|gb|GAXD01002776.1| | XP_001664041 | phosphatidylinositol-binding clathrin assembly protein | 161 | -31.47 | 2 | 16 | 2038 | 2056 | 14 | Query: 3' ccuccCGCGCCGCCGCCGCc 5' | Ref: 5' gcacaGC-CGGCGGCGGCGc 3' |
| gga-miR-7475-5p | gi|619317972|gb|GAXD01002777.1| | XP_001664041 | phosphatidylinositol-binding clathrin assembly protein | 161 | -31.47 | 2 | 16 | 2104 | 2122 | 14 | Query: 3' ccuccCGCGCCGCCGCCGCc 5' | Ref: 5' gcacaGC-CGGCGGCGGCGc 3' |
| gga-miR-7475-5p | gi|619300545|gb|GAXD01020077.1| | XP_001664041 | phosphatidylinositol-binding clathrin assembly protein | 161 | -31.47 | 2 | 16 | 2056 | 2074 | 14 | Query: 3' ccuccCGCGCCGCCGCCGCc 5' | Ref: 5' gcacaGC-CGGCGGCGGCGc 3' |
| gga-miR-7475-5p | gi|619298791|gb|GAXD01021830.1| | XP_001663122 | arylalkylamine n-acetyltransferase 1 | 171 | -35.03 | 2 | 16 | 671 | 690 | 14 | Query: 3' ccuccCGCGCCGCCGCCGCc 5' | Ref: 5' gcgtcGTGCGGCGGCGGCGt 3' |
| hsa-miR-4459 | gi|619299765|gb|GAXD01020856.1| | XP_001656812 | threonine dehydrogenase | 154 | -35.71 | 2 | 21 | 452 | 476 | 22 | Query: 3' gaGGUGGA--GGAGGC-GGAGGACc 5' | Ref: 5' ctTCACTTGAGCCCCGACCTCCTGg 3' |
| isc-miR-3931 | gi|619319041|gb|GAXD01001708.1| | XP_001655295 | NFAT, putative | 184 | -31.12 | 2 | 22 | 1370 | 1393 | 21 | Query: 3' ucCUAA-GCAUGGCUGAGUUUCAu 5' | Ref: 5' caGGTTGCCTACCGACTCAAAGTg 3' |
| gga-miR-7475-5p | gi|619297545|gb|GAXD01023076.1| | XP_001653296 | hypothetical protein AaeL_AAEL008569 | 161 | -32.54 | 2 | 18 | 2355 | 2374 | 16 | Query: 3' ccuCCCGCGCCGCCGCCGCc 5' | Ref: 5' gttGGAGGCGGGGGCGGCGc 3' |
| hsa-miR-4459 | gi|619297053|gb|GAXD01023568.1| | XP_001649036 | nuclear pore complex protein nup98 | 146 | -31.53 | 2 | 21 | 900 | 924 | 22 | Query: 3' gaGGUGGA--GGAG-GCGGAGGACc 5' | Ref: 5' aaCCACCTGAGAACAGGCCTCCTGg 3' |
| gga-miR-7475-5p | gi|619295539|gb|GAXD01024599.1| | XP_001607551 | pleckstrin homology domain-containing family g member 5-like | 156 | -32.27 | 2 | 18 | 664 | 684 | 17 | Query: 3' ccuCCCGCGCCG-CCGCCGCc 5' | Ref: 5' ggcGGCCGTCGCGGGCGGCGg 3' |
| hsa-miR-6787-5p | gi|619297967|gb|GAXD01022654.1| | XP_001607390 | serine threonine-protein kinase ulk2-like isoform 1 | 151 | -30.12 | 2 | 12 | 297 | 318 | 10 | Query: 3' cgucggucgagAUGGGGGCGGu 5' | Ref: 5' acatggtgagaTGCCCCCGCCa 3' |
| hsa-miR-4459 | gi|619314631|gb|GAXD01006118.1| | XP_001607270 | 60s ribosomal protein l11 | 166 | -30.5 | 2 | 21 | 3839 | 3859 | 19 | Query: 3' gaGGUGGAGGAGGCGGAGGACc 5' | Ref: 5' caCCA-CGCTTCCTCCTCCTGt 3' |
| hsa-miR-4459 | gi|619320325|gb|GAXD01000424.1| | XP_001606528 | lipase 1-like | 171 | -36.5 | 2 | 21 | 809 | 831 | 20 | Query: 3' gaGGUGGAG-GAGGCGGAGGACc 5' | Ref: 5' atCCGCTGCATTCCGCCTCCTGc 3' |
| hsa-miR-4638-3p | gi|619320325|gb|GAXD01000424.1| | XP_001606528 | lipase 1-like | 165 | -31.93 | 2 | 14 | 235 | 257 | 12 | Query: 3' gccggccgacUCGCCACAGGUCc 5' | Ref: 5' gccttaaagcAGCGGTGTCCAGg 3' |
| hsa-miR-4459 | gi|619318687|gb|GAXD01002062.1| | XP_001606497 | zinc transporter | 172 | -36.45 | 2 | 21 | 18 | 39 | 19 | Query: 3' gaGGUGGAGGAGGCGGAGGACc 5' | Ref: 5' gcCCGAACCCTCCGCCTCCTGc 3' |
| bta-miR-2379 | gi|619309626|gb|GAXD01010996.1| | XP_001605615 | adipocyte plasma membrane-associated | 180 | -32.36 | 2 | 21 | 1514 | 1535 | 19 | Query: 3' uuUUAUAGAAGAGGUCGUCGGa 5' | Ref: 5' cgAGCAGCTTCTCCAGCAGCCt 3' |
| gga-miR-7475-5p | gi|619315825|gb|GAXD01004924.1| | XP_001605059 | fatty acid synthase-like isoform 1 | 155 | -33.5 | 2 | 17 | 956 | 976 | 16 | Query: 3' ccucCCGCGCCG-CCGCCGCc 5' | Ref: 5' cggaGTTGTGGCAGGCGGCGa 3' |
| gga-miR-7475-5p | gi|619300556|gb|GAXD01020066.1| | XP_001605059 | fatty acid synthase-like isoform 1 | 159 | -30.53 | 2 | 19 | 241 | 258 | 17 | Query: 3' ccUCCCGCGCCGCCGCCGCc 5' | Ref: 5' cgAGGGAGCG--GGCGGCGa 3' |
| gga-miR-7475-5p | gi|619300556|gb|GAXD01020066.1| | XP_001605059 | fatty acid synthase-like isoform 1 | 159 | -30.41 | 2 | 17 | 1063 | 1083 | 16 | Query: 3' ccucCCGC-GCCGCCGCCGCc 5' | Ref: 5' tggcGGCGATGTCGGCGGCGa 3' |
| ppc-miR-8316-3p | gi|619300556|gb|GAXD01020066.1| | XP_001605059 | fatty acid synthase-like isoform 1 | 160 | -30.08 | 2 | 18 | 54 | 75 | 19 | Query: 3' cgCUGCUGGAC---CUGUGGUa 5' | Ref: 5' atGGCGGCCTGGCCGACACCAc 3' |
| hsa-miR-4638-3p | gi|619310929|gb|GAXD01009820.1| | XP_001604810 | cytochrome p450 cyp12a2 | 159 | -34.93 | 2 | 22 | 340 | 361 | 20 | Query: 3' gcCGGCCGACUCGCCACAGGUCc 5' | Ref: 5' ggGCCGG-AGGTTGTTGTCCAGg 3' |
| tca-miR-750-3p | gi|619298765|gb|GAXD01021856.1| | XP_001602416 | translational activator gcn1-like | 158 | -31.47 | 2 | 19 | 439 | 460 | 17 | Query: 3' gcaguauACCUUCUCAAUCUAGACc 5' | Ref: 5' aacgtctTGGAAGA---AGATCTGg 3' |
| tca-miR-750-3p | gi|619298763|gb|GAXD01021858.1| | XP_001602416 | translational activator gcn1-like | 158 | -31.47 | 2 | 19 | 439 | 460 | 17 | Query: 3' gcaguauACCUUCUCAAUCUAGACc 5' | Ref: 5' aacgtctTGGAAGA---AGATCTGg 3' |
| bmo-miR-3344 | gi|619318704|gb|GAXD01002045.1| | XP_001601669 | dolichyl-diphosphooligosaccharide--protein glycosyltransferase subunit 2-like | 182 | -33.35 | 2 | 24 | 166 | 191 | 23 | Query: 3' gaGCGACCGA-CUCAGGAAGAACGUu 5' | Ref: 5' tgCACTGGCTCGAGTCAGTCTTGCAt 3' |
| hsa-miR-4459 | gi|619299164|gb|GAXD01021457.1| | XP_001600320 | serine-threonine kinase receptor-associated | 172 | -37.14 | 2 | 21 | 656 | 677 | 19 | Query: 3' gaGGUGGAGGAGGCGGAGGACc 5' | Ref: 5' ctCCACTTTTTGCACCTCCTGg 3' |
| hsa-miR-6787-5p | gi|619298506|gb|GAXD01022115.1| | XP_001600235 | 3-phosphoinositide-dependent protein kinase 1-like | 166 | -37.83 | 2 | 21 | 38 | 58 | 19 | Query: 3' cgUCGGUCGAGAUGGGGGCGGu 5' | Ref: 5' acGGGCAGC-CCACCCCCGCCa 3' |
| hsa-miR-4638-3p | gi|619302899|gb|GAXD01017723.1| | WP_019861481 | family transcriptional regulator | 159 | -40.57 | 2 | 22 | 245 | 275 | 28 | Query: 3' gcCGGCCGAC-------UCGCC-ACAGGUCc 5' | Ref: 5' atGCCGGTTGAAATTCCAGCGGATGTCCAGg 3' |
| hsa-miR-6787-5p | gi|619320203|gb|GAXD01000546.1| | WP_017813111 | endoglucanase | 153 | -30.5 | 2 | 18 | 1883 | 1902 | 16 | Query: 3' cgucgGUCGAGAUGGGGGCGGu 5' | Ref: 5' cgaaaCAGC-C-ATCCCCGCCa 3' |
| hsa-miR-6787-5p | gi|619320202|gb|GAXD01000547.1| | WP_017813111 | endoglucanase | 153 | -30.5 | 2 | 18 | 2323 | 2342 | 16 | Query: 3' cgucgGUCGAGAUGGGGGCGGu 5' | Ref: 5' cgaaaCAGC-C-ATCCCCGCCa 3' |
| gga-miR-1723 | gi|619295605|gb|GAXD01024533.1| | WP_009258689 | swarming motility protein ybia | 172 | -34.44 | 2 | 22 | 164 | 187 | 21 | Query: 3' acUCCGACGUG-UAAGGCGAGGGu 5' | Ref: 5' tgAGGGTGCTCAACTCCGCTCCCc 3' |
| mmu-miR-5108 | gi|619301795|gb|GAXD01018827.1| | WP_002430991 | conserved hypothetical protein | 180 | -33.6 | 2 | 17 | 21 | 39 | 15 | Query: 3' uuuGGUAGGUCACGAGAUg 5' | Ref: 5' catCCATCCAGTGCTCTAc 3' |
| mmu-miR-6990-5p | gi|619298258|gb|GAXD01022363.1| | P22648 | fas2_scham ame: full=fasciclin-2 ame: full=fasciclin ii short=fas ii flags: precursor | 151 | -33.21 | 2 | 18 | 306 | 331 | 21 | Query: 3' ucucGGGAC-UGAG----UGGGACCc 5' | Ref: 5' aggtTCCTGAACTCGATGACCCTGGg 3' |
| ame-miR-3049-3p | gi|619317267|gb|GAXD01003482.1| | NP_001166084 | serine protease 49 precursor | 159 | -30.4 | 2 | 21 | 19 | 42 | 21 | Query: 3' ucUGCCUUUCCUC--AACCUGCCu 5' | Ref: 5' ggACGGGATGGCGCCCTGGACGGg 3' |
| mja-miR-6493-3p | gi|619312708|gb|GAXD01008041.1| | NP_001166081 | serine protease 52 precursor | 153 | -30.24 | 2 | 23 | 146 | 172 | 24 | Query: 3' auUGCGAGUCGC---GCCAAAGGGGGa 5' | Ref: 5' ggACGCCAAGCGCAACCAGTTCCCCCa 3' |
| gga-miR-7475-5p | gi|619309180|gb|GAXD01011442.1| | NP_001165966 | esterase fe4 | 151 | -32.14 | 2 | 17 | 472 | 492 | 16 | Query: 3' ccucCCGC-GCCGCCGCCGCc 5' | Ref: 5' tcgtGGCGTTCACGGCGGCGg 3' |
| hsa-miR-4459 | gi|619309180|gb|GAXD01011442.1| | NP_001165966 | esterase fe4 | 159 | -31.21 | 2 | 20 | 614 | 635 | 18 | Query: 3' gagGUGGAGGAGGCGGAGGACc 5' | Ref: 5' cggCGCGTCAACGGCCTCCTGg 3' |
| gga-miR-7475-5p | gi|619303891|gb|GAXD01016731.1| | NP_001153721 | atp-dependent rna helicase belle | 163 | -37.35 | 2 | 17 | 221 | 241 | 16 | Query: 3' ccucCCG-CGCCGCCGCCGCc 5' | Ref: 5' acttGGCGGCGGGGGCGGCGg 3' |
| mmu-miR-6990-5p | gi|619319054|gb|GAXD01001695.1| | NP_001116500 | matrix metalloproteinase 1 isoform 2 | 159 | -32.85 | 2 | 20 | 670 | 690 | 18 | Query: 3' ucUCGGGACUGAGUGGGACCc 5' | Ref: 5' cgGGCCCGGCGGCACCCTGGc 3' |
| gga-miR-7475-5p | gi|619304058|gb|GAXD01016564.1| | NP_001085720 | glycerol kinase 5 | 149 | -34.14 | 2 | 19 | 77 | 99 | 20 | Query: 3' ccUCCCGCGCCG---CCGCCGCc 5' | Ref: 5' cgAGGATGCGATCAAGGCGGCGg 3' |
| gga-miR-7475-5p | gi|619299404|gb|GAXD01021217.1| | NP_001011592 | tetraspanin 6 | 156 | -32.76 | 2 | 17 | 1087 | 1106 | 15 | Query: 3' ccucCCGCGCCGCCGCCGCc 5' | Ref: 5' ggccGACATGGTGGCGGCGg 3' |
| gga-miR-7475-5p | gi|619299404|gb|GAXD01021217.1| | NP_001011592 | tetraspanin 6 | 153 | -36.12 | 2 | 19 | 1153 | 1173 | 18 | Query: 3' ccUCCCGCGC-CGCCGCCGCc 5' | Ref: 5' tgGGGTCGGGCACGGCGGCGg 3' |
| hsa-miR-4459 | gi|619312953|gb|GAXD01007796.1| | ESO85346 | gamma-glutamyltranspeptidase 3-like | 167 | -34.1 | 2 | 21 | 168 | 190 | 20 | Query: 3' gaGGUGGAGGAGG-CGGAGGACc 5' | Ref: 5' ttCCAAAACCTCCTGCCTCCTGt 3' |
| hsa-miR-4459 | gi|619312468|gb|GAXD01008281.1| | ESO82003 | exportin-6 isoform 2 | 166 | -35.03 | 2 | 21 | 77 | 101 | 22 | Query: 3' gaGGUG-GAG--GAGGCGGAGGACc 5' | Ref: 5' aaCCACACTCAGCTTCCCCTCCTGg 3' |
| efu-miR-9198a | gi|619308010|gb|GAXD01012612.1| | ESO02556 | wd and tetratricopeptide repeats protein 1-like | 173 | -30.4 | 2 | 22 | 9 | 31 | 20 | Query: 3' agUGUAGGUGACUGUCACGGUUc 5' | Ref: 5' atACGTCCGACAACAGTGCCAAa 3' |
| hsa-miR-6787-5p | gi|619312265|gb|GAXD01008484.1| | ERM96395 | 4-coumarate- ligase-like protein | 151 | -30.29 | 2 | 18 | 191 | 211 | 16 | Query: 3' cgucgGUCGAGAUGGGGGCGGu 5' | Ref: 5' gcgtaCCGATC-GCCCCCGCCg 3' |
| hsa-miR-4459 | gi|619308573|gb|GAXD01012049.1| | ERL94398 | hypothetical protein D910_11677 | 169 | -30.85 | 2 | 21 | 104 | 123 | 19 | Query: 3' gaGGUGGAGGAGGCGGAGGACc 5' | Ref: 5' agCCATCTTCTC--CCTCCTGt 3' |
| hsa-miR-4459 | gi|619297563|gb|GAXD01023058.1| | ERL93320 | gad1_drome ame: full=gastrulation defective protein 1 homolog | 169 | -30.66 | 2 | 18 | 482 | 503 | 16 | Query: 3' gagguGGAGGAGGCGGAGGACc 5' | Ref: 5' cttttCCTTTTTTGCCTCCTGa 3' |
| gga-miR-7475-5p | gi|619304731|gb|GAXD01015891.1| | ERL92772 | sugar transporter 18 | 152 | -34.82 | 2 | 19 | 111 | 134 | 21 | Query: 3' ccUCCCGC-GCCG---CCGCCGCc 5' | Ref: 5' gtAGGGTGAAGGCGAAGGCGGCGg 3' |
| hsa-miR-4459 | gi|619294924|gb|GAXD01025214.1| | ERL92281 | bullous pemphigoid antigen isoforms 6 9 10 | 163 | -37.44 | 2 | 21 | 83 | 105 | 20 | Query: 3' gaGGUGG-AGGAGGCGGAGGACc 5' | Ref: 5' cgCCGCCAAGCTCGGCCTCCTGg 3' |
| hsa-miR-6787-5p | gi|619294678|gb|GAXD01025460.1| | ERL91856 | midline fasciclin | 162 | -33.98 | 2 | 21 | 838 | 858 | 19 | Query: 3' cgUCGGUCGAGAUGGGGGCGGu 5' | Ref: 5' tcAGCGAGCTGGA-CCCCGCCa 3' |
| hsa-miR-4459 | gi|619300032|gb|GAXD01020590.1| | ERL84562 | phospholipid scramblase 1 | 165 | -34.07 | 2 | 20 | 722 | 742 | 18 | Query: 3' gagGUGGAGGAGGCGGAGGACc 5' | Ref: 5' ggaTGTCTCCTCT-CCTCCTGg 3' |
| mmu-miR-5625-5p | gi|619298782|gb|GAXD01021839.1| | ENN80871 | peptidyl-prolyl cis-trans isomerase | 173 | -33.11 | 2 | 20 | 442 | 461 | 18 | Query: 3' agGAUGAGUUCUUGAAGGCCc 5' | Ref: 5' gaCTGCT-GAGAACTTCCGGg 3' |
| hsa-miR-4459 | gi|619295167|gb|GAXD01024971.1| | ENN76864 | isoform b | 158 | -31.45 | 2 | 20 | 1264 | 1286 | 19 | Query: 3' gagGUGGAGGAGG-CGGAGGACc 5' | Ref: 5' ggaCGCCCTCGCTGGCCTCCTGg 3' |
| hsa-miR-4459 | gi|619317377|gb|GAXD01003372.1| | ENN73803 | hypothetical protein YQE_09580, partial | 168 | -34.64 | 2 | 21 | 421 | 442 | 19 | Query: 3' gaGGUGGAGGAGGCGGAGGACc 5' | Ref: 5' atCCACGTCCCTTACCTCCTGg 3' |
| gga-miR-7475-5p | gi|619318948|gb|GAXD01001801.1| | ENN70525 | hypothetical protein YQE_12701, partial | 143 | -31.16 | 2 | 14 | 89 | 107 | 12 | Query: 3' ccucccgCGCCGCCGCCGCc 5' | Ref: 5' actgcagGAGG-GGCGGCGg 3' |
| gga-miR-7475-5p | gi|619293956|gb|GAXD01026182.1| | ELA31896 | 4-coumarate- ligase | 159 | -30.9 | 2 | 18 | 56 | 74 | 16 | Query: 3' ccuCCCGCGCCGCCGCCGCc 5' | Ref: 5' agtGGCTGC-GCGGCGGCGt 3' |
| gga-miR-1723 | gi|619318538|gb|GAXD01002211.1| | EKC39039 | adp-ribosylation factor gtpase-activating protein 2 | 163 | -30.92 | 2 | 22 | 2095 | 2116 | 20 | Query: 3' acUCCGACGUGUAAGGCGAGGGu 5' | Ref: 5' agAGGTT-TGCCTTGCGCTCCCa 3' |
| hsa-miR-4459 | gi|619315368|gb|GAXD01005381.1| | EHJ78426 | inorganic phosphate cotransporter-like | 166 | -35.25 | 2 | 15 | 185 | 206 | 13 | Query: 3' gagguggaGGAGGCGGAGGACc 5' | Ref: 5' cttccgtaCCTCTGCCTCCTGc 3' |
| hsa-miR-6787-5p | gi|619300806|gb|GAXD01019816.1| | EHJ77437 | bullous pemphigoid antigen isoforms 6 9 10 | 150 | -32.68 | 2 | 21 | 50 | 74 | 22 | Query: 3' cgUCGGU-CGAGAU--GGGGGCGGu 5' | Ref: 5' tcGGCCATGGTCAAGGACCCCGCCa 3' |
| hsa-miR-6787-5p | gi|619298644|gb|GAXD01021977.1| | EHJ71927 | facilitated trehalose transporter tret1-like | 160 | -30.76 | 2 | 19 | 350 | 370 | 17 | Query: 3' cgucGGUCGAGAUGGGGGCGGu 5' | Ref: 5' ggctCCAGTGC-GCCCCCGCCc 3' |
| mmu-miR-5625-5p | gi|619312728|gb|GAXD01008021.1| | EHJ71830 | low quality protein: integrator complex subunit 3-like | 177 | -33.44 | 2 | 20 | 226 | 245 | 18 | Query: 3' agGAUGAGUUCUUGAAGGCCc 5' | Ref: 5' tgCTACT-AAGAGCTTCCGGg 3' |
| hsa-miR-6787-5p | gi|619297402|gb|GAXD01023219.1| | EHJ66728 | protein associated with topo ii-related 1 | 160 | -34.35 | 2 | 19 | 872 | 892 | 17 | Query: 3' cgucGGUCGAGAUGGGGGCGGu 5' | Ref: 5' gggcCCAG-AATACCCCCGCCa 3' |
| gga-miR-7475-5p | gi|619300227|gb|GAXD01020395.1| | EHJ66162 | regulator of ribosome biosynthesis | 157 | -32.55 | 2 | 14 | 840 | 859 | 12 | Query: 3' ccucccgCGCCGCCGCCGCc 5' | Ref: 5' aagtttgGTGGTGGCGGCGg 3' |
| hsa-miR-4459 | gi|619309631|gb|GAXD01010991.1| | EHJ64389 | nadp-dependent oxidoreductase | 175 | -41.13 | 2 | 21 | 72 | 94 | 20 | Query: 3' gaGGUGGA-GGAGGCGGAGGACc 5' | Ref: 5' caCCGCCTACTTCGGCCTCCTGg 3' |
| ame-miR-3049-3p | gi|619304944|gb|GAXD01015678.1| | EGW00840 | xin actin-binding repeat-containing protein 1 | 169 | -36.57 | 2 | 21 | 416 | 438 | 21 | Query: 3' ucUGCCU--UUCCUCAACCUGCCu 5' | Ref: 5' ggACGGATGAAGGGG-TGGACGGa 3' |
| gga-miR-7475-5p | gi|619320416|gb|GAXD01000333.1| | EGI68611 | rna-binding protein cabeza | 174 | -43.71 | 2 | 19 | 400 | 419 | 17 | Query: 3' ccUCCCGCGCCGCCGCCGCc 5' | Ref: 5' cgAGGCGGCGGCGGCGGCGg 3' |
| hsa-miR-6787-5p | gi|619289128|gb|GAXD01031010.1| | EGI67150 | choline-phosphate cytidylyltransferase b | 167 | -31.46 | 2 | 20 | 227 | 248 | 18 | Query: 3' cguCGGUCGAGAUGGGGGCGGu 5' | Ref: 5' aatGCAGGCCTTATCCCCGCCg 3' |
| hsa-miR-4638-3p | gi|619320037|gb|GAXD01000712.1| | EGI66502 | trinucleotide repeat-containing gene 6a protein | 160 | -30.69 | 2 | 19 | 622 | 643 | 17 | Query: 3' gccggCCGACUCGCCACAGGUCc 5' | Ref: 5' gctgaGGCTGCG-GCTGTCCAGg 3' |
| hsa-miR-4638-3p | gi|619299001|gb|GAXD01021620.1| | EGI66502 | trinucleotide repeat-containing gene 6a protein | 160 | -30.69 | 2 | 19 | 658 | 679 | 17 | Query: 3' gccggCCGACUCGCCACAGGUCc 5' | Ref: 5' gctgaGGCTGCG-GCTGTCCAGg 3' |
| mja-miR-6493-3p | gi|619300103|gb|GAXD01020519.1| | EGI65783 | litaf-like protein | 155 | -35.26 | 2 | 23 | 171 | 201 | 29 | Query: 3' auUGCGAGUCGCGCCA--------AAGGGGGa 5' | Ref: 5' ttATGCTCAG-GCGGTTGGAGGCGTTCCCCCa 3' |
| ame-miR-3049-3p | gi|619315339|gb|GAXD01005410.1| | EGI64728 | e3 ubiquitin-protein ligase ubr5 | 166 | -30.43 | 2 | 20 | 153 | 177 | 21 | Query: 3' ucuGCCUUUCC---UCAACCUGCCu 5' | Ref: 5' aacTGGAATGGGGCAGTTGGACGGa 3' |
| hsa-miR-4459 | gi|619320668|gb|GAXD01000081.1| | EGI63671 | zinc finger rna-binding protein | 159 | -34.67 | 2 | 21 | 1255 | 1279 | 22 | Query: 3' gaGGUGGAGGAGGC---GGAGGACc 5' | Ref: 5' atCCCCGTCCTTTGGGACCTCCTGg 3' |
| hsa-miR-4459 | gi|619320667|gb|GAXD01000082.1| | EGI63671 | zinc finger rna-binding protein | 159 | -34.67 | 2 | 21 | 1333 | 1357 | 22 | Query: 3' gaGGUGGAGGAGGC---GGAGGACc 5' | Ref: 5' atCCCCGTCCTTTGGGACCTCCTGg 3' |
| bmo-miR-3344 | gi|619297228|gb|GAXD01023393.1| | EGI62379 | Syntaxin-12 | 164 | -30.75 | 2 | 22 | 252 | 277 | 21 | Query: 3' gagcGACCGA-CUCAGGAAGAACGUu 5' | Ref: 5' tggaCTGGCTGGAGGGGGTCTTGCAg 3' |
| gga-miR-7475-5p | gi|619314572|gb|GAXD01006177.1| | EGI60892 | low quality protein: cubilin-like | 161 | -35.4 | 2 | 18 | 225 | 244 | 16 | Query: 3' ccuCCCGCGCCGCCGCCGCc 5' | Ref: 5' gccGAGGGCTGCGGCGGCGa 3' |
| gga-miR-7475-5p | gi|619295582|gb|GAXD01024556.1| | EGI60892 | Cubilin | 152 | -33.23 | 2 | 18 | 1223 | 1243 | 17 | Query: 3' ccuCCCGCGC-CGCCGCCGCc 5' | Ref: 5' cgtGGTGGAGAGCGGCGGCGg 3' |
| gga-miR-7475-5p | gi|619294561|gb|GAXD01025577.1| | EGI60892 | Cubilin | 165 | -32.01 | 2 | 19 | 326 | 346 | 18 | Query: 3' ccUCC-CGCGCCGCCGCCGCc 5' | Ref: 5' caAGGAGAGCTGCGGCGGCGt 3' |
| hsa-miR-6787-5p | gi|619298357|gb|GAXD01022264.1| | EGI58340 | purine nucleoside phosphorylase | 154 | -30.69 | 2 | 21 | 731 | 751 | 19 | Query: 3' cgUCGGUCGAGAUGGGGGCGGu 5' | Ref: 5' tcGGACCGCGCT-TCCCCGCCa 3' |
| gga-miR-7475-5p | gi|619295095|gb|GAXD01025043.1| | EGI58305 | sugar transporter erd6-like 4 | 156 | -30.67 | 2 | 17 | 556 | 575 | 15 | Query: 3' ccucCCGCGCCGCCGCCGCc 5' | Ref: 5' tcctGACGGAGCGGCGGCGg 3' |
| gga-miR-7475-5p | gi|619299707|gb|GAXD01020914.1| | EGF76564 | rna-binding protein 34-like | 165 | -39.94 | 2 | 18 | 179 | 198 | 16 | Query: 3' ccuCCCGCGCCGCCGCCGCc 5' | Ref: 5' ggtGGTGGTGGCGGCGGCGg 3' |
| crm-miR-786 | gi|619300527|gb|GAXD01020095.1| | EFZ23162 | e3 ubiquitin-protein ligase huwe1 | 184 | -30.12 | 2 | 22 | 5821 | 5844 | 21 | Query: 3' uaCCUUAG-AGUCGUUCCCGUAAu 5' | Ref: 5' tgGGAATTATCCGCAAGGGCATTa 3' |
| hsa-miR-6787-5p | gi|619298609|gb|GAXD01022012.1| | EFZ22996 | kinesin light chain | 153 | -32.15 | 2 | 20 | 231 | 255 | 21 | Query: 3' cguCGGUCGA-G--AUGGGGGCGGu 5' | Ref: 5' aatGCTGGCTACGAGATCCCCGCCa 3' |
| hsa-miR-4638-3p | gi|619302572|gb|GAXD01018050.1| | EFZ22635 | hypothetical protein SINV_00315 | 162 | -30.06 | 2 | 20 | 2655 | 2679 | 20 | Query: 3' gccgGCCGACUC--GCCACAGGUCc 5' | Ref: 5' gttgCTGCTGAGTCTGTTGTCCAGg 3' |
| hsa-miR-6787-5p | gi|619309067|gb|GAXD01011555.1| | EFZ18650 | neogenin 1-like protein | 166 | -34.49 | 2 | 19 | 18 | 39 | 17 | Query: 3' cgucGGUCGAGAUGGGGGCGGu 5' | Ref: 5' atcaTCGACTCTGTCCCCGCCg 3' |
| gga-miR-7475-5p | gi|619320099|gb|GAXD01000650.1| | EFZ17028 | endonuclease and reverse transcriptase-like protein | 158 | -30.08 | 2 | 19 | 19 | 38 | 17 | Query: 3' ccUCCCGCGCCGCCGCCGCc 5' | Ref: 5' ggAGGCTCTGGAGGCGGCGc 3' |
| mja-miR-6489-5p | gi|619307939|gb|GAXD01012683.1| | EFZ15506 | hypothetical protein SINV_02754 | 156 | -30.23 | 2 | 17 | 33 | 52 | 15 | Query: 3' uuccCGCGGUCAGGCCACGg 5' | Ref: 5' tccaGGGGAAGTCCGGTGCa 3' |
| hsa-miR-6787-5p | gi|619313173|gb|GAXD01007576.1| | EFZ09608 | zinc finger protein fyve domain containing | 156 | -30.51 | 2 | 21 | 1373 | 1391 | 19 | Query: 3' cgUCGGUCGAGAUGGGGGCGGu 5' | Ref: 5' agAGTCAG---AACCCCCGCCc 3' |
| gga-miR-7475-5p | gi|619299128|gb|GAXD01021493.1| | EFZ09250 | bullous pemphigoid antigen isoforms 6 9 10 | 173 | -35.01 | 2 | 19 | 709 | 729 | 18 | Query: 3' ccUCCCGCGCCG-CCGCCGCc 5' | Ref: 5' ccAGAGCGCGGCGGGCGGCGt 3' |
| hsa-miR-4459 | gi|619310476|gb|GAXD01010235.1| | EFX85598 | histone-arginine methyltransferase carmer-like isoform 1 | 154 | -31.86 | 2 | 21 | 469 | 489 | 19 | Query: 3' gaGGUGGAGGAGGCGGAGGACc 5' | Ref: 5' gcCTGCC-CAGCCACCTCCTGg 3' |
| gga-miR-7475-5p | gi|619319645|gb|GAXD01001104.1| | EFX84548 | hypothetical protein DAPPUDRAFT_314899 | 159 | -30.05 | 2 | 16 | 753 | 772 | 14 | Query: 3' ccuccCGCGCCGCCGCCGCc 5' | Ref: 5' acgctGCACCGCGGCGGCGg 3' |
| gga-miR-7475-5p | gi|619319645|gb|GAXD01001104.1| | EFX84548 | hypothetical protein DAPPUDRAFT_314899 | 151 | -30.2 | 2 | 16 | 2664 | 2683 | 14 | Query: 3' ccuccCGCGCCGCCGCCGCc 5' | Ref: 5' accctGCGAGCAGGCGGCGg 3' |
| hsa-miR-6787-5p | gi|619319645|gb|GAXD01001104.1| | EFX84548 | hypothetical protein DAPPUDRAFT_314899 | 157 | -31.9 | 2 | 20 | 1306 | 1330 | 21 | Query: 3' cguCGGUCG-AGAUG--GGGGCGGu 5' | Ref: 5' tgcGCCCGCATCGACATCCCCGCCa 3' |
| gga-miR-7475-5p | gi|619297354|gb|GAXD01023267.1| | EFX80390 | hypothetical protein DAPPUDRAFT_318553 | 159 | -30.05 | 2 | 16 | 753 | 772 | 14 | Query: 3' ccuccCGCGCCGCCGCCGCc 5' | Ref: 5' acgctGCACCGCGGCGGCGg 3' |
| hsa-miR-6787-5p | gi|619297354|gb|GAXD01023267.1| | EFX80390 | hypothetical protein DAPPUDRAFT_318553 | 157 | -31.9 | 2 | 20 | 1306 | 1330 | 21 | Query: 3' cguCGGUCG-AGAUG--GGGGCGGu 5' | Ref: 5' tgcGCCCGCATCGACATCCCCGCCa 3' |
| hsa-miR-4459 | gi|619306798|gb|GAXD01013824.1| | EFX75168 | hypothetical protein DAPPUDRAFT_323673 | 154 | -38.45 | 2 | 20 | 23 | 49 | 23 | Query: 3' gagGUGGAG-----GAGGCGGAGGACc 5' | Ref: 5' gtaCATCTCCGGAGCGTCGCCTCCTGg 3' |
| hsa-miR-6787-5p | gi|619296174|gb|GAXD01023964.1| | EFX62063 | venom allergen 3-like isoform 1 | 160 | -33.11 | 2 | 21 | 722 | 740 | 19 | Query: 3' cgUCGGUCGAGAUGGGGGCGGu 5' | Ref: 5' gaGGCCGGC---ACCCCCGCCt 3' |
| hsa-miR-6787-5p | gi|619306357|gb|GAXD01014265.1| | EFR28262 | filamin-c-like isoform x4 | 170 | -33.15 | 2 | 21 | 162 | 182 | 19 | Query: 3' cgUCGGUCGAGAUGGGGGCGGu 5' | Ref: 5' cgAGCTGGCCCT-CCCCCGCCt 3' |
| gga-miR-7475-5p | gi|619308408|gb|GAXD01012214.1| | EFR26368 | carbonic anhydrase 1 | 153 | -32.34 | 2 | 19 | 652 | 672 | 18 | Query: 3' ccUCCCGCG-CCGCCGCCGCc 5' | Ref: 5' acGGCGAGCAGGAGGCGGCGg 3' |
| hsa-miR-4459 | gi|619308408|gb|GAXD01012214.1| | EFR26368 | carbonic anhydrase 1 | 158 | -30.64 | 2 | 19 | 1507 | 1528 | 17 | Query: 3' gaggUGGAGGAGGCGGAGGACc 5' | Ref: 5' ggggACCGCCCGAGCCTCCTGg 3' |
| hsa-miR-6787-5p | gi|619306829|gb|GAXD01013793.1| | EFR24366 | 5-aminolevulinic acid synthase | 159 | -32.56 | 2 | 21 | 61 | 83 | 20 | Query: 3' cgUCGGUCGAGAUG-GGGGCGGu 5' | Ref: 5' ccGGCCGTCCCTTCACCCCGCCa 3' |
| hsa-miR-6787-5p | gi|619306825|gb|GAXD01013797.1| | EFR24366 | 5-aminolevulinic acid synthase | 159 | -32.56 | 2 | 21 | 61 | 83 | 20 | Query: 3' cgUCGGUCGAGAUG-GGGGCGGu 5' | Ref: 5' ccGGCCGTCCCTTCACCCCGCCa 3' |
| gga-miR-7475-5p | gi|619299712|gb|GAXD01020909.1| | EFN89745 | e3 ubiquitin-protein ligase ubr3 | 168 | -41.2 | 2 | 19 | 364 | 386 | 20 | Query: 3' ccUCCCG--CG-CCGCCGCCGCc 5' | Ref: 5' gaAGGGCAAGCGGGCGGCGGCGg 3' |
| hsa-miR-4459 | gi|619318390|gb|GAXD01002359.1| | EFN87810 | lim domain only protein 7 | 163 | -30.1 | 2 | 16 | 1393 | 1414 | 14 | Query: 3' gagguggAGGAGGCGGAGGACc 5' | Ref: 5' acgtctgTGCTCTGCCTCCTGt 3' |
| hsa-miR-4459 | gi|619299971|gb|GAXD01020651.1| | EFN87810 | lim domain only protein 7 | 163 | -30.1 | 2 | 16 | 1393 | 1414 | 14 | Query: 3' gagguggAGGAGGCGGAGGACc 5' | Ref: 5' acgtctgTGCTCTGCCTCCTGt 3' |
| hsa-miR-4638-3p | gi|619297619|gb|GAXD01023002.1| | EFN86660 | ataxin-2-like protein | 160 | -30.37 | 2 | 22 | 182 | 206 | 22 | Query: 3' gcCGGC--CGACUCGCCACAGGUCc 5' | Ref: 5' gtGCTGAAGCTGGGATTTGTCCAGg 3' |
| gga-miR-7475-5p | gi|619311405|gb|GAXD01009344.1| | EFN85802 | serine threonine-protein kinase genghis khan | 150 | -33.27 | 2 | 11 | 29 | 48 | 9 | Query: 3' ccucccgcgcCGCCGCCGCc 5' | Ref: 5' tgttcttggtGCGGCGGCGg 3' |
| mja-miR-6489-5p | gi|619317124|gb|GAXD01003625.1| | EFN83086 | gtpase-activating rap ran-gap domain-like protein 3 | 156 | -30.48 | 2 | 19 | 47 | 70 | 21 | Query: 3' uuCCCGCGG-UCA---GGCCACGg 5' | Ref: 5' cgGGTCGCCTAGTACCCCGGTGCt 3' |
| hsa-miR-6787-5p | gi|619319541|gb|GAXD01001208.1| | EFN82417 | solute carrier family 41 member 1-like | 160 | -31.41 | 2 | 21 | 3368 | 3389 | 19 | Query: 3' cgUCGGUCGAGAUGGGGGCGGu 5' | Ref: 5' acAGACTCGTCTCCCCCCGCCa 3' |
| hsa-miR-6787-5p | gi|619308325|gb|GAXD01012297.1| | EFN82417 | solute carrier family 41 member 1-like | 160 | -31.41 | 2 | 21 | 719 | 740 | 19 | Query: 3' cgUCGGUCGAGAUGGGGGCGGu 5' | Ref: 5' acAGACTCGTCTCCCCCCGCCa 3' |
| gga-miR-7475-5p | gi|619313313|gb|GAXD01007436.1| | EFN82103 | nuclear receptor-binding protein homolog | 157 | -30.07 | 2 | 19 | 1915 | 1935 | 18 | Query: 3' ccUCCCGCG-CCGCCGCCGCc 5' | Ref: 5' caATGTCGCTGCCGGCGGCGg 3' |
| hsa-miR-4459 | gi|619312148|gb|GAXD01008601.1| | EFN81966 | protein c1orf9-like protein | 180 | -35.89 | 2 | 21 | 648 | 669 | 19 | Query: 3' gaGGUGGAGGAGGCGGAGGACc 5' | Ref: 5' ttTTGCCTCCTCCTCCTCCTGc 3' |
| hsa-miR-4459 | gi|619311910|gb|GAXD01008839.1| | EFN80503 | Afadin | 169 | -30.92 | 2 | 18 | 2635 | 2656 | 16 | Query: 3' gagguGGAGGAGGCGGAGGACc 5' | Ref: 5' cgttcCCGCCTCAGCCTCCTGa 3' |
| hsa-miR-6787-5p | gi|619297568|gb|GAXD01023053.1| | EFN78426 | peptidylglycine alpha-hydroxylating monooxygenase | 154 | -31.08 | 2 | 21 | 466 | 486 | 19 | Query: 3' cgUCGGUCGAGAUGGGGGCGGu 5' | Ref: 5' ccAGCGGCCGCTG-CCCCGCCc 3' |
| gga-miR-7475-5p | gi|619297000|gb|GAXD01023621.1| | EFN76250 | Prestin | 154 | -30.03 | 2 | 16 | 356 | 376 | 15 | Query: 3' ccuccCGCG-CCGCCGCCGCc 5' | Ref: 5' ccgccGTGCAGGAGGCGGCGg 3' |
| hsa-miR-4638-3p | gi|619318184|gb|GAXD01002565.1| | EFN74805 | cytochrome p450 4c1 | 154 | -36.01 | 2 | 22 | 12 | 35 | 22 | Query: 3' gcCGGCCGACUCG--CCACAGGUCc 5' | Ref: 5' tgGGCGGC-GAGCATCATGTCCAGg 3' |
| hsa-miR-4459 | gi|619299574|gb|GAXD01021047.1| | EFN72044 | 14-3-3 protein zeta | 160 | -32.39 | 2 | 21 | 2783 | 2804 | 19 | Query: 3' gaGGUGGAGGAGGCGGAGGACc 5' | Ref: 5' cgCTATGTCAAACGCCTCCTGg 3' |
| gga-miR-7475-5p | gi|619310141|gb|GAXD01010507.1| | EFN71944 | eukaryotic translation initiation factor 4 gamma 3 | 154 | -32.38 | 2 | 17 | 743 | 764 | 17 | Query: 3' ccucCCGCG-CC-GCCGCCGCc 5' | Ref: 5' atccGCCGCAGGCCGGCGGCGg 3' |
| hsa-miR-6787-5p | gi|619318158|gb|GAXD01002591.1| | EFN71504 | pancreatic lipase-related protein 2 | 161 | -31.48 | 2 | 19 | 436 | 458 | 18 | Query: 3' cgucGGUCGAG-AUGGGGGCGGu 5' | Ref: 5' gtttCCGGCGCACACCCCCGCCt 3' |
| hsa-miR-6787-5p | gi|619297931|gb|GAXD01022690.1| | EFN71504 | pancreatic lipase-related protein 2 | 161 | -31.48 | 2 | 19 | 372 | 394 | 18 | Query: 3' cgucGGUCGAG-AUGGGGGCGGu 5' | Ref: 5' gtttCCGGCGCACACCCCCGCCt 3' |
| hsa-miR-6787-5p | gi|619297928|gb|GAXD01022693.1| | EFN71504 | pancreatic lipase-related protein 2 | 161 | -31.48 | 2 | 19 | 372 | 394 | 18 | Query: 3' cgucGGUCGAG-AUGGGGGCGGu 5' | Ref: 5' gtttCCGGCGCACACCCCCGCCt 3' |
| hsa-miR-4638-3p | gi|619300288|gb|GAXD01020334.1| | EFN70286 | mitogen-activated protein kinase-binding protein 1 | 152 | -30.95 | 2 | 18 | 544 | 567 | 17 | Query: 3' gccggcCGACUCGC-CACAGGUCc 5' | Ref: 5' tggtgaGGTGGGTGACTGTCCAGg 3' |
| gga-miR-7475-5p | gi|619311890|gb|GAXD01008859.1| | EFN67110 | Talin-1 | 153 | -32 | 2 | 19 | 17 | 39 | 20 | Query: 3' ccUCCCGCGC---CGCCGCCGCc 5' | Ref: 5' tgAGGGCTTGATATCGGCGGCGc 3' |
| gga-miR-7475-5p | gi|619307976|gb|GAXD01012646.1| | EFN66210 | Cubilin | 159 | -33.41 | 2 | 18 | 233 | 251 | 16 | Query: 3' ccuCCCGCGCCGCCGCCGCc 5' | Ref: 5' tgcGGACGT-GCGGCGGCGg 3' |
| mmu-miR-5119 | gi|619319572|gb|GAXD01001177.1| | EFN65329 | serine threonine-protein kinase wnk1 | 167 | -30.34 | 2 | 16 | 1613 | 1631 | 14 | Query: 3' ggucGGGGUCCUACUCUAc 5' | Ref: 5' ctctTCCCGGGATGAGATg 3' |
| hsa-miR-6787-5p | gi|619315297|gb|GAXD01005452.1| | EFN65152 | cordon-bleu 1 | 158 | -31.57 | 2 | 20 | 140 | 162 | 19 | Query: 3' cguCGGUCGAGAUG-GGGGCGGu 5' | Ref: 5' tccGCCAGCAGAGCACCCCGCCg 3' |
| mja-miR-6489-5p | gi|619320078|gb|GAXD01000671.1| | EFN64991 | zinc-type alcohol dehydrogenase-like protein | 147 | -33.59 | 2 | 19 | 1780 | 1800 | 19 | Query: 3' uuCCCG--CGGUCAGGCCACGg 5' | Ref: 5' gaGGGCGAGGCCG-CCGGTGCc 3' |
| mja-miR-6489-5p | gi|619318520|gb|GAXD01002229.1| | EFN64991 | zinc-type alcohol dehydrogenase-like protein | 147 | -33.59 | 2 | 19 | 2376 | 2396 | 19 | Query: 3' uuCCCG--CGGUCAGGCCACGg 5' | Ref: 5' gaGGGCGAGGCCG-CCGGTGCc 3' |
| mja-miR-6489-5p | gi|619296185|gb|GAXD01023953.1| | EFN64991 | zinc-type alcohol dehydrogenase-like protein | 147 | -33.59 | 2 | 19 | 2457 | 2477 | 19 | Query: 3' uuCCCG--CGGUCAGGCCACGg 5' | Ref: 5' gaGGGCGAGGCCG-CCGGTGCc 3' |
| hsa-miR-4459 | gi|619297945|gb|GAXD01022676.1| | EFN60525 | pre-mrna 3 -end-processing factor fip1 | 164 | -31.85 | 2 | 20 | 167 | 186 | 18 | Query: 3' gagGUGGAGGAGGCGGAGGACc 5' | Ref: 5' atgCAC--CCTCCCCCTCCTGg 3' |
| gga-miR-7475-5p | gi|619319604|gb|GAXD01001145.1| | EFA13301 | hypothetical protein TcasGA2_TC014831 | 153 | -32.02 | 2 | 16 | 629 | 651 | 17 | Query: 3' ccuccCGCG--CC-GCCGCCGCc 5' | Ref: 5' tacatGCGCCAGGCCGGCGGCGg 3' |
| gga-miR-7475-5p | gi|619317920|gb|GAXD01002829.1| | EFA11365 | fatty acid synthase | 158 | -37.61 | 2 | 19 | 1919 | 1938 | 17 | Query: 3' ccUCCCGCGCCGCCGCCGCc 5' | Ref: 5' ggAGTCGGCGGGGGCGGCGg 3' |
| hsa-miR-6787-5p | gi|619317956|gb|GAXD01002793.1| | EFA07319 | ornithine decarboxylase-like | 151 | -31.51 | 2 | 21 | 3330 | 3354 | 22 | Query: 3' cgUCGGUCGAGA---UGGGGGCGGu 5' | Ref: 5' acAGTGAGTTTTCAGGGCCCCGCCg 3' |
| hsa-miR-6787-5p | gi|619308624|gb|GAXD01011998.1| | EFA03400 | hypothetical protein TcasGA2_TC013386 | 160 | -35.93 | 2 | 19 | 33 | 56 | 19 | Query: 3' cgucGGUCG-AGAUG-GGGGCGGu 5' | Ref: 5' gcgcCCAGCGGCTGCGCCCCGCCc 3' |
| hsa-miR-6787-5p | gi|619306827|gb|GAXD01013795.1| | EFA03355 | 5-aminolevulinic acid synthase | 159 | -32.56 | 2 | 21 | 61 | 83 | 20 | Query: 3' cgUCGGUCGAGAUG-GGGGCGGu 5' | Ref: 5' ccGGCCGTCCCTTCACCCCGCCa 3' |
| hsa-miR-4638-3p | gi|619300401|gb|GAXD01020221.1| | EFA02178 | rab3-gef cg5627-pb | 156 | -32.54 | 2 | 22 | 116 | 139 | 21 | Query: 3' gcCGGCCGAC-UCGCCACAGGUCc 5' | Ref: 5' gtGGTGGTGGCGGTCGTGTCCAGg 3' |
| mja-miR-6493-3p | gi|619311774|gb|GAXD01008975.1| | EFA00499 | sticks and stones cg33141-pa | 169 | -30.14 | 2 | 23 | 97 | 122 | 23 | Query: 3' auUGCGAGUCGCGC--CAAAGGGGGa 5' | Ref: 5' tgAAGCTTAGTGTGCTCTTTCCCCCa 3' |
| gga-miR-7475-5p | gi|619298287|gb|GAXD01022334.1| | EEZ97174 | rna-binding protein 1 | 169 | -41.96 | 2 | 18 | 464 | 483 | 16 | Query: 3' ccuCCCGCGCCGCCGCCGCc 5' | Ref: 5' ggtGGTGGCGGCGGCGGCGg 3' |
| gga-miR-7475-5p | gi|619294151|gb|GAXD01025987.1| | CDJ21009 | hypothetical protein EgrG_000335800 | 149 | -33.72 | 2 | 16 | 1327 | 1350 | 18 | Query: 3' ccuccCGC--GCCG--CCGCCGCc 5' | Ref: 5' gggccGCGACCGGCTGGGCGGCGg 3' |
| gga-miR-7475-5p | gi|619294150|gb|GAXD01025988.1| | CDJ21009 | hypothetical protein EgrG_000335800 | 149 | -33.72 | 2 | 16 | 1314 | 1337 | 18 | Query: 3' ccuccCGC--GCCG--CCGCCGCc 5' | Ref: 5' gggccGCGACCGGCTGGGCGGCGg 3' |
| gga-miR-7475-5p | gi|619291653|gb|GAXD01028485.1| | CCF23214 | forkhead box subgroup partial | 160 | -36.86 | 2 | 17 | 92 | 111 | 15 | Query: 3' ccucCCGCGCCGCCGCCGCc 5' | Ref: 5' tggaGGTGGCGCGGCGGCGc 3' |
| tca-miR-279d-3p | gi|619302625|gb|GAXD01017997.1| | CAJ17317 | 60s ribosomal protein l24 | 153 | -30.58 | 2 | 23 | 321 | 354 | 30 | Query: 3' gauAUCUGCUCAUACCU---------AGAUCAGu 5' | Ref: 5' atcTTGACGGGTGTGGAAATCACACTTCTAGTCt 3' |
| hsa-miR-4638-3p | gi|619305564|gb|GAXD01015058.1| | CAB55603 | moderately methionine rich storage protein | 168 | -37.49 | 2 | 22 | 159 | 182 | 21 | Query: 3' gcCGGCCGACUCGCC-ACAGGUCc 5' | Ref: 5' aaGCCGAGAGGGCGGTTGTCCAGg 3' |
| gga-miR-7475-5p | gi|619304238|gb|GAXD01016384.1| | BAO00946 | crustacean cardioactive peptide | 157 | -37.08 | 2 | 19 | 165 | 186 | 19 | Query: 3' ccUCCCGCGCC--GCCGCCGCc 5' | Ref: 5' ccGGGACGAGGGCCGGCGGCGg 3' |
| hsa-miR-6787-5p | gi|619299335|gb|GAXD01021286.1| | BAN21261 | moesin ezrin radixin homolog 1 | 155 | -30.02 | 2 | 20 | 3774 | 3793 | 18 | Query: 3' cguCGGUCGAGAUGGGGGCGGu 5' | Ref: 5' gccGCCCGC-CTG-CCCCGCCg 3' |
| mmu-miR-5119 | gi|619295286|gb|GAXD01024852.1| | BAN21021 | pre-mrna-processing factor 19-like | 165 | -30.15 | 2 | 14 | 578 | 596 | 12 | Query: 3' ggucggGGUCCUACUCUAc 5' | Ref: 5' tgtagaCCAGGATGAGATg 3' |
| gga-miR-7475-5p | gi|619315943|gb|GAXD01004806.1| | BAN20945 | splicing factor hnrnp- | 167 | -41.86 | 2 | 17 | 259 | 279 | 16 | Query: 3' ccucCCG-CGCCGCCGCCGCc 5' | Ref: 5' tggtGGCGGCGGTGGCGGCGg 3' |
| hsa-miR-6787-5p | gi|619300101|gb|GAXD01020521.1| | BAN20936 | peroxiredoxin | 155 | -31.48 | 2 | 18 | 886 | 909 | 18 | Query: 3' cgucgGUCGAG-A-UGGGGGCGGu 5' | Ref: 5' cactgCAGCTTGTGGTCCCCGCCg 3' |
| mja-miR-6493-3p | gi|619297389|gb|GAXD01023232.1| | BAN20834 | choline-phosphate cytidylyltransferase b | 179 | -35.24 | 2 | 22 | 1173 | 1195 | 20 | Query: 3' auuGCGAGUCGCGCCAAAGGGGGa 5' | Ref: 5' tgcTGCTCTGCGC-GTTTCCCCCg 3' |
| hsa-miR-4638-3p | gi|619309239|gb|GAXD01011383.1| | BAN20639 | prohibitin 2 | 177 | -30.57 | 2 | 22 | 260 | 282 | 20 | Query: 3' gcCGGCCGACUCGCCACAGGUCc 5' | Ref: 5' caGTAGATTGAGTGGTGTCCAGa 3' |
| hsa-miR-6787-5p | gi|619320029|gb|GAXD01000720.1| | BAN20603 | protein tyrosine phosphatase prl | 157 | -30.59 | 2 | 14 | 2092 | 2113 | 12 | Query: 3' cgucggucgAGAUGGGGGCGGu 5' | Ref: 5' cgcagttcaTCTCCCCCCGCCc 3' |
| hsa-miR-6787-5p | gi|619316327|gb|GAXD01004422.1| | BAN20603 | protein tyrosine phosphatase prl | 157 | -30.59 | 2 | 14 | 1957 | 1978 | 12 | Query: 3' cgucggucgAGAUGGGGGCGGu 5' | Ref: 5' cgcagttcaTCTCCCCCCGCCc 3' |
| bmo-miR-3344 | gi|619316715|gb|GAXD01004034.1| | BAN20569 | conserved hypothetical protein | 165 | -31.37 | 2 | 24 | 2992 | 3019 | 25 | Query: 3' gaGCGACC--GACUCAGGA-AGAACGUu 5' | Ref: 5' gcTGCTGGGACTGAGGGGTCTCTTGCAa 3' |
| mja-miR-6493-3p | gi|619320705|gb|GAXD01000044.1| | BAJ78669 | rna polymerase ii largest subunit | 157 | -30.79 | 2 | 23 | 1571 | 1593 | 22 | Query: 3' auUGCGAGUCG-CGCCAAAGGGGGa 5' | Ref: 5' gaAC-CTCAGCTGC-ACTTCCCCCt 3' |
| hsa-miR-4459 | gi|619320352|gb|GAXD01000397.1| | BAI83426 | sugar transporter 12 | 155 | -30.25 | 2 | 16 | 1992 | 2013 | 14 | Query: 3' gagguggAGGAGGCGGAGGACc 5' | Ref: 5' gagcgagTCGCTCGCCTCCTGg 3' |
| mja-miR-6489-5p | gi|619319722|gb|GAXD01001027.1| | BAI83422 | sugar transporter 8 | 165 | -33.11 | 2 | 18 | 125 | 144 | 16 | Query: 3' uucCCGCGGUCAGGCCACGg 5' | Ref: 5' ggcGGCGTCATCCCGGTGCc 3' |
| gga-miR-7475-5p | gi|619301712|gb|GAXD01018910.1| | BAI83420 | sugar transporter 6 | 160 | -31.17 | 2 | 19 | 2156 | 2174 | 17 | Query: 3' ccUCCCGCGCCGCCGCCGCc 5' | Ref: 5' atGGGGC-CGATGGCGGCGc 3' |
| hsa-miR-4638-3p | gi|619294910|gb|GAXD01025228.1| | BAG75121 | lipophorin precursor | 164 | -36.32 | 2 | 18 | 8696 | 8720 | 18 | Query: 3' gccggcCGACUC--GCCACAGGUCc 5' | Ref: 5' aggataGGTGAGGTCGGTGTCCAGg 3' |
| mmu-miR-5119 | gi|619316444|gb|GAXD01004305.1| | BAG46932 | cell wall-associated hydrolase | 185 | -41.81 | 2 | 18 | 355 | 373 | 16 | Query: 3' ggUCGGGGUCCUACUCUAc 5' | Ref: 5' acAGCCCCAGGATGAGATg 3' |
| mmu-miR-6990-5p | gi|619315603|gb|GAXD01005146.1| | BAG30740 | muscle myosin heavy chain | 167 | -30.09 | 2 | 18 | 364 | 383 | 16 | Query: 3' ucucGGGACUGAGUGGGACCc 5' | Ref: 5' tcttCCTTGAC-CACCCTGGc 3' |
| hsa-miR-6787-5p | gi|619295983|gb|GAXD01024155.1| | BAE80729 | luciferin 4-monooxygenase-like | 158 | -31.01 | 2 | 17 | 339 | 359 | 15 | Query: 3' cgucggUCGAGAUGGGGGCGGu 5' | Ref: 5' tcgcggACCTCTA-CCCCGCCg 3' |
| hsa-miR-6787-5p | gi|619295983|gb|GAXD01024155.1| | BAE80729 | luciferin 4-monooxygenase-like | 155 | -30.3 | 2 | 17 | 624 | 646 | 16 | Query: 3' cgucggUCGAGAUG-GGGGCGGu 5' | Ref: 5' ccgcggACCTCTTCACCCCGCCg 3' |
| gga-miR-1723 | gi|619298086|gb|GAXD01022535.1| | BAE46610 | reverse transcriptase | 156 | -33.98 | 2 | 22 | 656 | 677 | 21 | Query: 3' acUCCGACGUGUAAG-GCGAGGGu 5' | Ref: 5' ggAGG-AGCGC-TTCGCGCTCCCg 3' |
| gga-miR-1723 | gi|619298085|gb|GAXD01022536.1| | BAE46610 | reverse transcriptase | 156 | -33.98 | 2 | 22 | 656 | 677 | 21 | Query: 3' acUCCGACGUGUAAG-GCGAGGGu 5' | Ref: 5' ggAGG-AGCGC-TTCGCGCTCCCg 3' |
| mmu-miR-6240 | gi|619294914|gb|GAXD01025224.1| | BAA10929 | cytochrome p450 like_tbp | 178 | -30.95 | 2 | 25 | 70 | 97 | 25 | Query: 3' gcGGC-ACCC-GGAAGCGCUACGAAACc 5' | Ref: 5' atCCGCAGGGACCATCGCAATGCTTTGt 3' |
| gga-miR-7475-5p | gi|619299000|gb|GAXD01021621.1| | B4KR05 | tret1_dromo ame: full=facilitated trehalose transporter tret1 | 160 | -35.8 | 2 | 18 | 78 | 100 | 19 | Query: 3' ccuCCCG---CGCCGCCGCCGCc 5' | Ref: 5' gctGGGCCTGGCGCCGGCGGCGg 3' |
| hsa-miR-4459 | gi|619318688|gb|GAXD01002061.1| | AGN56418 | udp n-acetylglucosamine pyrophosphorylases 1 | 167 | -30.9 | 2 | 21 | 2837 | 2859 | 20 | Query: 3' gaGGUGGAGGAGG-CGGAGGACc 5' | Ref: 5' ggCCAGCTGCTCCACCCTCCTGa 3' |
| gga-miR-1723 | gi|619320703|gb|GAXD01000046.1| | AGM61336 | a chain pfv target capture complex at a resolution | 172 | -34.44 | 2 | 22 | 164 | 187 | 21 | Query: 3' acUCCGACGUG-UAAGGCGAGGGu 5' | Ref: 5' tgAGGGTGCTCAACTCCGCTCCCc 3' |
| mmu-miR-5124a | gi|619299324|gb|GAXD01021297.1| | AGM53719 | rna polymerase | 177 | -30.22 | 2 | 18 | 997 | 1016 | 16 | Query: 3' uacGAGAAUCAGUGACCUGg 5' | Ref: 5' tatTTTTTAGTCACTGGACc 3' |
| gga-miR-7475-5p | gi|619298790|gb|GAXD01021831.1| | AGM38193 | arylalkylamine n-acetyltransferase 2 | 171 | -35.03 | 2 | 16 | 671 | 690 | 14 | Query: 3' ccuccCGCGCCGCCGCCGCc 5' | Ref: 5' gcgtcGTGCGGCGGCGGCGt 3' |
| gga-miR-7475-5p | gi|619305850|gb|GAXD01014772.1| | AGM32567 | glycosyl-phosphatidyl-inositol-anchored protein | 178 | -45.33 | 2 | 19 | 1888 | 1907 | 17 | Query: 3' ccUCCCGCGCCGCCGCCGCc 5' | Ref: 5' cgAGGACGCGGTGGCGGCGg 3' |
| gga-miR-7475-5p | gi|619305849|gb|GAXD01014773.1| | AGM32567 | glycosyl-phosphatidyl-inositol-anchored protein | 178 | -45.33 | 2 | 19 | 1786 | 1805 | 17 | Query: 3' ccUCCCGCGCCGCCGCCGCc 5' | Ref: 5' cgAGGACGCGGTGGCGGCGg 3' |
| hsa-miR-6787-5p | gi|619317989|gb|GAXD01002760.1| | AGM32429 | scp-like extracellular domain containing protein 1 | 160 | -33.11 | 2 | 21 | 627 | 645 | 19 | Query: 3' cgUCGGUCGAGAUGGGGGCGGu 5' | Ref: 5' gaGGCCGGC---ACCCCCGCCt 3' |
| gga-miR-7475-5p | gi|619312460|gb|GAXD01008289.1| | AGM32374 | s9e ribosomal protein | 169 | -42.22 | 2 | 19 | 603 | 623 | 18 | Query: 3' ccUCCC-GCGCCGCCGCCGCc 5' | Ref: 5' tcAGGGAGGTGGCGGCGGCGg 3' |
| mmu-miR-6990-5p | gi|619317940|gb|GAXD01002809.1| | AGI37646 | heat shock cognate 70-2 protein | 160 | -30.78 | 2 | 20 | 1322 | 1340 | 18 | Query: 3' ucUCGGGACUGAGUGGGACCc 5' | Ref: 5' tgAACCCT--CTTACCCTGGg 3' |
| mmu-miR-6990-5p | gi|619296927|gb|GAXD01023694.1| | AGI37646 | heat shock cognate 70-2 protein | 160 | -30.78 | 2 | 20 | 1396 | 1414 | 18 | Query: 3' ucUCGGGACUGAGUGGGACCc 5' | Ref: 5' tgAACCCT--CTTACCCTGGg 3' |
| mmu-miR-6990-5p | gi|619299076|gb|GAXD01021545.1| | AGH30327 | agonaute 2 | 154 | -33.97 | 2 | 20 | 630 | 652 | 20 | Query: 3' ucUCGGGACUG--AGUGGGACCc 5' | Ref: 5' agGGCCTAGGCAGCCACCCTGGg 3' |
| mmu-miR-6990-5p | gi|619299075|gb|GAXD01021546.1| | AGH30327 | agonaute 2 | 154 | -33.97 | 2 | 20 | 630 | 652 | 20 | Query: 3' ucUCGGGACUG--AGUGGGACCc 5' | Ref: 5' agGGCCTAGGCAGCCACCCTGGg 3' |
| mmu-miR-6990-5p | gi|619299073|gb|GAXD01021548.1| | AGH30327 | agonaute 2 | 154 | -33.97 | 2 | 20 | 630 | 652 | 20 | Query: 3' ucUCGGGACUG--AGUGGGACCc 5' | Ref: 5' agGGCCTAGGCAGCCACCCTGGg 3' |
| hsa-miR-6787-5p | gi|619320557|gb|GAXD01000192.1| | AGG35950 | carbonic anhydrase 2-like partial | 157 | -30.72 | 2 | 19 | 1109 | 1131 | 18 | Query: 3' cgucGGUCGAGA-UGGGGGCGGu 5' | Ref: 5' gctcCTCGCGCTCGCCCCCGCCt 3' |
| hsa-miR-4459 | gi|619303816|gb|GAXD01016806.1| | AGE94872 | rna-binding protein of the pumilio family) | 166 | -35.11 | 2 | 15 | 95 | 116 | 13 | Query: 3' gagguggaGGAGGCGGAGGACc 5' | Ref: 5' ctaagaagTCTCCGCCTCCTGg 3' |
| hsa-miR-4459 | gi|619311877|gb|GAXD01008872.1| | AGC78890 | hypothetical protein (mitochondrion) | 155 | -31.55 | 2 | 21 | 1191 | 1215 | 22 | Query: 3' gaGGUGGAGG---AGGCGGAGGACc 5' | Ref: 5' ggCTGCTTCCAAGCCCACCTCCTGg 3' |
| gga-miR-7475-5p | gi|619320738|gb|GAXD01000011.1| | AFW97644 | vitellogenin | 165 | -33.38 | 2 | 19 | 190 | 210 | 18 | Query: 3' ccUCCCGCG-CCGCCGCCGCc 5' | Ref: 5' agAGGGCCCAGGAGGCGGCGa 3' |
| gga-miR-7475-5p | gi|619320738|gb|GAXD01000011.1| | AFW97644 | vitellogenin | 161 | -34.57 | 2 | 14 | 725 | 744 | 12 | Query: 3' ccucccgCGCCGCCGCCGCc 5' | Ref: 5' gtcgaagGTGGCGGCGGCGg 3' |
| gga-miR-7475-5p | gi|619320738|gb|GAXD01000011.1| | AFW97644 | vitellogenin | 161 | -30.89 | 2 | 16 | 1554 | 1572 | 14 | Query: 3' ccuccCGCGCCGCCGCCGCc 5' | Ref: 5' ttgatGC-CGGCGGCGGCGc 3' |
| gga-miR-7475-5p | gi|619320738|gb|GAXD01000011.1| | AFW97644 | vitellogenin | 151 | -31.2 | 2 | 19 | 2500 | 2517 | 17 | Query: 3' ccUCCCGCGCCGCCGCCGCc 5' | Ref: 5' ggAGGGAGT--TGGCGGCGc 3' |
| gga-miR-7475-5p | gi|619320738|gb|GAXD01000011.1| | AFW97644 | vitellogenin | 149 | -32.54 | 2 | 19 | 1176 | 1196 | 18 | Query: 3' ccUC-CCGCGCCGCCGCCGCc 5' | Ref: 5' tgAGAGATGGGCTGGCGGCGg 3' |
| hsa-miR-6787-5p | gi|619320740|gb|GAXD01000009.1| | AFW97644 | vitellogenin | 167 | -33.47 | 2 | 21 | 925 | 948 | 21 | Query: 3' cgUCGGUCGAGAU--GGGGGCGGu 5' | Ref: 5' ccAGTGAGCTGTACGCCCCCGCCt 3' |
| hsa-miR-6787-5p | gi|619320736|gb|GAXD01000013.1| | AFW97644 | vitellogenin | 158 | -36 | 2 | 21 | 3435 | 3460 | 23 | Query: 3' cgUCGGUC-GAGAUG---GGGGCGGu 5' | Ref: 5' gaGGCCGGCCGCTACCAGCCCCGCCa 3' |
| hsa-miR-4459 | gi|619320739|gb|GAXD01000010.1| | AFW97644 | vitellogenin | 163 | -34.38 | 2 | 16 | 3204 | 3225 | 14 | Query: 3' gagguggAGGAGGCGGAGGACc 5' | Ref: 5' tcagcagTTCGCCGCCTCCTGg 3' |
| hsa-miR-4459 | gi|619320736|gb|GAXD01000013.1| | AFW97644 | vitellogenin | 163 | -34.38 | 2 | 16 | 3311 | 3332 | 14 | Query: 3' gagguggAGGAGGCGGAGGACc 5' | Ref: 5' tcagcagTTCGCCGCCTCCTGg 3' |
| hsa-miR-4638-3p | gi|619313951|gb|GAXD01006798.1| | AFN83771 | protein disulfide isomerase | 147 | -31.04 | 2 | 22 | 484 | 511 | 25 | Query: 3' gcCGGCCGACUC---GC--CACAGGUCc 5' | Ref: 5' tgGTGGACTGAGCATCGACCTGTCCAGg 3' |
| gga-miR-7475-5p | gi|619308265|gb|GAXD01012357.1| | AEE63510 | glycine n-methyltransferase | 151 | -32.97 | 2 | 17 | 96 | 116 | 16 | Query: 3' ccucCCGCGC-CGCCGCCGCc 5' | Ref: 5' acgcGCTGGGAGCGGCGGCGg 3' |
| hsa-miR-4459 | gi|619295785|gb|GAXD01024353.1| | AEE62409 | acyl- -binding protein | 156 | -30.24 | 2 | 19 | 72 | 96 | 20 | Query: 3' gaggUGGAGGAG--GC-GGAGGACc 5' | Ref: 5' gtagGCCTTCTTGGCGTCCTCCTGg 3' |
| hsa-miR-6787-5p | gi|619293993|gb|GAXD01026145.1| | ACT53736 | juvenile hormone esterase-like protein est1 | 163 | -34.08 | 2 | 21 | 153 | 175 | 20 | Query: 3' cgUCGGUCG-AGAUGGGGGCGGu 5' | Ref: 5' ggGGCCAGCGCCTCACCCCGCCg 3' |
| mmu-miR-6990-5p | gi|619315604|gb|GAXD01005145.1| | ACQ91270 | myosin heavy chain | 167 | -30.09 | 2 | 18 | 364 | 383 | 16 | Query: 3' ucucGGGACUGAGUGGGACCc 5' | Ref: 5' tcttCCTTGAC-CACCCTGGc 3' |
| gga-miR-7475-5p | gi|619295318|gb|GAXD01024820.1| | ACE75395 | protein 5nuc-like | 157 | -33.89 | 2 | 18 | 29 | 48 | 16 | Query: 3' ccuCCCGCGCCGCCGCCGCc 5' | Ref: 5' gacGAGGGCGATGGCGGCGt 3' |
| gga-miR-7475-5p | gi|619311959|gb|GAXD01008790.1| | ABZ04021 | serine protease 13 | 159 | -34.97 | 2 | 17 | 693 | 713 | 16 | Query: 3' ccucCCGCGCCG-CCGCCGCc 5' | Ref: 5' ggtcGGCGCAGTGGGCGGCGg 3' |
| hsa-miR-4459 | gi|619307186|gb|GAXD01013436.1| | ABY66392 | tubulin beta-1 chain | 159 | -37.12 | 2 | 21 | 333 | 357 | 22 | Query: 3' gaGGUGGAGGAG---GCGGAGGACc 5' | Ref: 5' gtCCTCGTCCGCGGTCGCCTCCTGg 3' |
| gga-miR-7475-5p | gi|619300385|gb|GAXD01020237.1| | ABX57814 | delta class glutathione s-transferase | 161 | -31.11 | 2 | 19 | 103 | 123 | 18 | Query: 3' ccUCCCGCGC-CGCCGCCGCc 5' | Ref: 5' ccACGGCGCGCTTGGCGGCGt 3' |
| hsa-miR-4459 | gi|619301448|gb|GAXD01019174.1| | ABN12015 | inositol -triphosphate kinase 1-like protein | 158 | -30.8 | 2 | 21 | 851 | 875 | 22 | Query: 3' gaGGU-GGA--GGAGGCGGAGGACc 5' | Ref: 5' cgTCATCCTGCCCACCACCTCCTGg 3' |
| hsa-miR-4459 | gi|619307189|gb|GAXD01013433.1| | ABH03477 | beta-1 partial | 159 | -37.12 | 2 | 21 | 333 | 357 | 22 | Query: 3' gaGGUGGAGGAG---GCGGAGGACc 5' | Ref: 5' gtCCTCGTCCGCGGTCGCCTCCTGg 3' |
| hsa-miR-4459 | gi|619307188|gb|GAXD01013434.1| | ABH03477 | beta-1 partial | 159 | -37.12 | 2 | 21 | 333 | 357 | 22 | Query: 3' gaGGUGGAGGAG---GCGGAGGACc 5' | Ref: 5' gtCCTCGTCCGCGGTCGCCTCCTGg 3' |
| gga-miR-7475-5p | gi|619301479|gb|GAXD01019143.1| | AAZ94273 | cytochrome p450 | 166 | -33.5 | 2 | 16 | 1870 | 1890 | 15 | Query: 3' ccuccCGCGCCG-CCGCCGCc 5' | Ref: 5' gtcccGCGCGGCGGGCGGCGa 3' |
| hsa-miR-4459 | gi|619298016|gb|GAXD01022605.1| | AAQ55293 | retinoid x receptor | 157 | -30.34 | 2 | 20 | 1102 | 1122 | 18 | Query: 3' gagGUGGAGGAGGCGGAGGACc 5' | Ref: 5' gtgCGCTGTCTCT-CCTCCTGg 3' |
| hsa-miR-4459 | gi|619298015|gb|GAXD01022606.1| | AAQ55293 | retinoid x receptor | 157 | -30.34 | 2 | 20 | 1060 | 1080 | 18 | Query: 3' gagGUGGAGGAGGCGGAGGACc 5' | Ref: 5' gtgCGCTGTCTCT-CCTCCTGg 3' |
| efu-miR-9198a | gi|619319303|gb|GAXD01001446.1| | AAL78751 | translation elongation factor-1 gamma | 173 | -30.48 | 2 | 18 | 353 | 375 | 16 | Query: 3' aguguaGGUGACUGUCACGGUUc 5' | Ref: 5' aagttgCCACTGATCGTGCCAAg 3' |
| efu-miR-9198a | gi|619298520|gb|GAXD01022101.1| | AAL78751 | translation elongation factor-1 gamma | 173 | -30.48 | 2 | 18 | 353 | 375 | 16 | Query: 3' aguguaGGUGACUGUCACGGUUc 5' | Ref: 5' aagttgCCACTGATCGTGCCAAg 3' |
| mmu-miR-6990-5p | gi|619318373|gb|GAXD01002376.1| | AAF09840 | AE001886_6hypothetical protein DR_0254 | 169 | -38.41 | 2 | 20 | 133 | 152 | 18 | Query: 3' ucUCGGGACUGAGUGGGACCc 5' | Ref: 5' agGGACC-GACTCACCCTGGg 3' |
| mja-miR-6493-3p | gi|619318373|gb|GAXD01002376.1| | AAF09840 | AE001886_6hypothetical protein DR_0254 | 165 | -32.61 | 2 | 22 | 105 | 126 | 20 | Query: 3' auuGCGAGUCGCGCCAAAGGGGGa 5' | Ref: 5' catCGCCTA-CG-GGTTTCCCCCt 3' |
| gga-miR-7475-5p | gi|619317838|gb|GAXD01002911.1| | AAD16096 | r2 protein | 152 | -35.09 | 2 | 19 | 2407 | 2430 | 21 | Query: 3' ccUCCCGCGCC--G--CCGCCGCc 5' | Ref: 5' ggAGGGCCTGGACCTGGGCGGCGa 3' |
| hsa-miR-4459 | gi|619314326|gb|GAXD01006423.1| | AAC25395 | ubiquitin hydrolase | 177 | -33.7 | 2 | 18 | 4 | 25 | 16 | Query: 3' gagguGGAGGAGGCGGAGGACc 5' | Ref: 5' tcagtCCTCCTCCCCCTCCTGa 3' |
| mmu-miR-6990-5p | gi|619320158|gb|GAXD01000591.1| | AAB48959 | Gag | 158 | -30.97 | 2 | 20 | 1557 | 1579 | 20 | Query: 3' ucUCGGGACUGA--GUGGGACCc 5' | Ref: 5' ggAGGCCTTAGTGGCACCCTGGg 3' |
| hsa-miR-6787-5p | gi|619320158|gb|GAXD01000591.1| | AAB48959 | Gag | 160 | -32.89 | 2 | 19 | 1143 | 1163 | 17 | Query: 3' cgucGGUCGAGAUGGGGGCGGu 5' | Ref: 5' cgctCCTCCTCT-CCCCCGCCg 3' |
| dme-miR-281-2-5p | gi|619296980|gb|GAXD01023641.1| | #N/A | #N/A | 179 | -31.78 | 2 | 21 | 254 | 276 | 20 | Query: 3' ugAC-AGCUGCCUAUCGAGAGAa 5' | Ref: 5' ttTGTTCGATGGAGAGCTCTCTt 3' |
| dgr-miR-281-2-5p | gi|619296980|gb|GAXD01023641.1| | #N/A | #N/A | 179 | -31.78 | 2 | 21 | 254 | 276 | 20 | Query: 3' ugAC-AGCUGCCUAUCGAGAGAa 5' | Ref: 5' ttTGTTCGATGGAGAGCTCTCTt 3' |
| lmi-miR-281-5p | gi|619296980|gb|GAXD01023641.1| | #N/A | #N/A | 179 | -31.78 | 2 | 21 | 254 | 276 | 20 | Query: 3' ugAC-AGCUGCCUAUCGAGAGAa 5' | Ref: 5' ttTGTTCGATGGAGAGCTCTCTt 3' |
| tca-miR-281-5p | gi|619296980|gb|GAXD01023641.1| | #N/A | #N/A | 179 | -31.78 | 2 | 21 | 254 | 276 | 20 | Query: 3' ugAC-AGCUGCCUAUCGAGAGAa 5' | Ref: 5' ttTGTTCGATGGAGAGCTCTCTt 3' |
| der-miR-281-2-5p | gi|619296980|gb|GAXD01023641.1| | #N/A | #N/A | 179 | -31.78 | 2 | 21 | 254 | 276 | 20 | Query: 3' ugAC-AGCUGCCUAUCGAGAGAa 5' | Ref: 5' ttTGTTCGATGGAGAGCTCTCTt 3' |
| cqu-miR-281-5p | gi|619296980|gb|GAXD01023641.1| | #N/A | #N/A | 179 | -31.78 | 2 | 21 | 254 | 276 | 20 | Query: 3' ugAC-AGCUGCCUAUCGAGAGAa 5' | Ref: 5' ttTGTTCGATGGAGAGCTCTCTt 3' |
| dwi-miR-281-2-5p | gi|619296980|gb|GAXD01023641.1| | #N/A | #N/A | 179 | -31.78 | 2 | 21 | 254 | 276 | 20 | Query: 3' ugAC-AGCUGCCUAUCGAGAGAa 5' | Ref: 5' ttTGTTCGATGGAGAGCTCTCTt 3' |
| dya-miR-281-2-5p | gi|619296980|gb|GAXD01023641.1| | #N/A | #N/A | 179 | -31.78 | 2 | 21 | 254 | 276 | 20 | Query: 3' ugAC-AGCUGCCUAUCGAGAGAa 5' | Ref: 5' ttTGTTCGATGGAGAGCTCTCTt 3' |
| dpe-miR-281-2-5p | gi|619296980|gb|GAXD01023641.1| | #N/A | #N/A | 179 | -31.78 | 2 | 21 | 254 | 276 | 20 | Query: 3' ugAC-AGCUGCCUAUCGAGAGAa 5' | Ref: 5' ttTGTTCGATGGAGAGCTCTCTt 3' |
| bmo-miR-281-5p | gi|619296980|gb|GAXD01023641.1| | #N/A | #N/A | 179 | -31.78 | 2 | 21 | 254 | 276 | 20 | Query: 3' ugAC-AGCUGCCUAUCGAGAGAa 5' | Ref: 5' ttTGTTCGATGGAGAGCTCTCTt 3' |
| mmu-miR-6240 | gi|619318265|gb|GAXD01002484.1| | #N/A | #N/A | 162 | -32.77 | 2 | 20 | 303 | 329 | 19 | Query: 3' gcggcacCCGGAAGC-GCUACGAAACc 5' | Ref: 5' acgaagaGGCCCCCGTTGGTGCTTTGg 3' |
| mmu-miR-6240 | gi|619315837|gb|GAXD01004912.1| | #N/A | #N/A | 159 | -30.34 | 2 | 25 | 215 | 241 | 24 | Query: 3' gcGGCACCCG-GAAGCGCUACGAAACc 5' | Ref: 5' gcCCGTATGTAGGTCATGGTGCTTTGg 3' |
| mmu-miR-6240 | gi|619307730|gb|GAXD01012892.1| | #N/A | #N/A | 157 | -30.66 | 2 | 25 | 142 | 167 | 24 | Query: 3' gcGGC-ACCCGGAAGCGCUACGAAACc 5' | Ref: 5' taCCGTTTCGCC-GGGAGATGCTTTGg 3' |
| mmu-miR-6240 | gi|619306971|gb|GAXD01013651.1| | #N/A | #N/A | 161 | -30.04 | 2 | 25 | 258 | 287 | 27 | Query: 3' gcGGC-ACCCG-GAAGCGCU--ACGAAACc 5' | Ref: 5' tgCCGTTGGGCGGTTCTCCGTTTGCTTTGc 3' |
| mmu-miR-6240 | gi|619304695|gb|GAXD01015927.1| | #N/A | #N/A | 163 | -38.96 | 2 | 25 | 3 | 31 | 26 | Query: 3' gcGGCACCCGGAAGCGCU---ACGAAACc 5' | Ref: 5' cgCCGTGGGCTGGGGAGGCTCTGCTTTGg 3' |
| mmu-miR-6240 | gi|619298397|gb|GAXD01022224.1| | #N/A | #N/A | 164 | -32.26 | 2 | 23 | 661 | 691 | 26 | Query: 3' gcggCAC-CCGGAAGCGC----UACGAAACc 5' | Ref: 5' aatgGTGTGGCTTTGGTGTTCTATGCTTTGg 3' |
| mmu-miR-6240 | gi|619292749|gb|GAXD01027389.1| | #N/A | #N/A | 165 | -30.37 | 2 | 23 | 94 | 121 | 23 | Query: 3' gcggCACCCGGAAGCGC--UACGAAACc 5' | Ref: 5' gcggGTGGGCTCCCGTGTCCTGCTTTGt 3' |
| mmu-miR-6240 | gi|619290356|gb|GAXD01029782.1| | #N/A | #N/A | 168 | -31.07 | 2 | 24 | 107 | 132 | 23 | Query: 3' gcgGCA-CCCGGAAGCGCUACGAAACc 5' | Ref: 5' atgTGTCGTGTCTTC-TGGTGCTTTGt 3' |
| dan-miR-281-2-5p | gi|619296980|gb|GAXD01023641.1| | #N/A | #N/A | 179 | -31.78 | 2 | 21 | 254 | 276 | 20 | Query: 3' ugAC-AGCUGCCUAUCGAGAGAa 5' | Ref: 5' ttTGTTCGATGGAGAGCTCTCTt 3' |
| dvi-miR-281-2-5p | gi|619296980|gb|GAXD01023641.1| | #N/A | #N/A | 179 | -31.78 | 2 | 21 | 254 | 276 | 20 | Query: 3' ugAC-AGCUGCCUAUCGAGAGAa 5' | Ref: 5' ttTGTTCGATGGAGAGCTCTCTt 3' |
| dse-miR-281-2-5p | gi|619296980|gb|GAXD01023641.1| | #N/A | #N/A | 179 | -31.78 | 2 | 21 | 254 | 276 | 20 | Query: 3' ugAC-AGCUGCCUAUCGAGAGAa 5' | Ref: 5' ttTGTTCGATGGAGAGCTCTCTt 3' |
| dmo-miR-281-2-5p | gi|619296980|gb|GAXD01023641.1| | #N/A | #N/A | 179 | -31.78 | 2 | 21 | 254 | 276 | 20 | Query: 3' ugAC-AGCUGCCUAUCGAGAGAa 5' | Ref: 5' ttTGTTCGATGGAGAGCTCTCTt 3' |
| ame-miR-2796 | gi|619319618|gb|GAXD01001131.1| | #N/A | #N/A | 150 | -30.16 | 2 | 22 | 428 | 456 | 26 | Query: 3' cgUUCAU-CAAA---GGCG--GCCGGAUg 5' | Ref: 5' gcAAGGACGTGTGGACCGCTACGGCCTAc 3' |
| ame-miR-2796 | gi|619309063|gb|GAXD01011559.1| | #N/A | #N/A | 158 | -30.77 | 2 | 21 | 113 | 134 | 19 | Query: 3' cguUCAUCAAAGGCGGCCGGAUg 5' | Ref: 5' gacGGGAGGTT-AGCCGGCCTAc 3' |
| ame-miR-2796 | gi|619299699|gb|GAXD01020922.1| | #N/A | #N/A | 160 | -35.03 | 2 | 13 | 31 | 53 | 11 | Query: 3' cguucaucaaaGGCGGCCGGAUg 5' | Ref: 5' gcacgggagccCCGCCGGCCTAc 3' |
| ame-miR-2796 | gi|619299306|gb|GAXD01021315.1| | #N/A | #N/A | 150 | -30.16 | 2 | 22 | 146 | 174 | 26 | Query: 3' cgUUCAU-CAAA---GGCG--GCCGGAUg 5' | Ref: 5' gcAAGGATGTGTGGACCGCTACGGCCTAc 3' |
| ame-miR-2796 | gi|619293556|gb|GAXD01026582.1| | #N/A | #N/A | 148 | -31.81 | 2 | 21 | 200 | 222 | 19 | Query: 3' cguUCAUCAAAGGCGGCCGGAUg 5' | Ref: 5' ggcGGGAGTGATGCTCGGCCTAc 3' |
| ame-miR-2796 | gi|619293555|gb|GAXD01026583.1| | #N/A | #N/A | 148 | -31.81 | 2 | 21 | 182 | 204 | 19 | Query: 3' cguUCAUCAAAGGCGGCCGGAUg 5' | Ref: 5' ggcGGGAGTGATGCTCGGCCTAc 3' |
| mse-miR-2796 | gi|619319618|gb|GAXD01001131.1| | #N/A | #N/A | 150 | -30.16 | 2 | 22 | 428 | 456 | 26 | Query: 3' cgUUCAU-CAAA---GGCG--GCCGGAUg 5' | Ref: 5' gcAAGGACGTGTGGACCGCTACGGCCTAc 3' |
| mse-miR-2796 | gi|619309063|gb|GAXD01011559.1| | #N/A | #N/A | 158 | -30.77 | 2 | 21 | 113 | 134 | 19 | Query: 3' cguUCAUCAAAGGCGGCCGGAUg 5' | Ref: 5' gacGGGAGGTT-AGCCGGCCTAc 3' |
| mse-miR-2796 | gi|619299699|gb|GAXD01020922.1| | #N/A | #N/A | 160 | -35.03 | 2 | 13 | 31 | 53 | 11 | Query: 3' cguucaucaaaGGCGGCCGGAUg 5' | Ref: 5' gcacgggagccCCGCCGGCCTAc 3' |
| mse-miR-2796 | gi|619299306|gb|GAXD01021315.1| | #N/A | #N/A | 150 | -30.16 | 2 | 22 | 146 | 174 | 26 | Query: 3' cgUUCAU-CAAA---GGCG--GCCGGAUg 5' | Ref: 5' gcAAGGATGTGTGGACCGCTACGGCCTAc 3' |
| mse-miR-2796 | gi|619293556|gb|GAXD01026582.1| | #N/A | #N/A | 148 | -31.81 | 2 | 21 | 200 | 222 | 19 | Query: 3' cguUCAUCAAAGGCGGCCGGAUg 5' | Ref: 5' ggcGGGAGTGATGCTCGGCCTAc 3' |
| mse-miR-2796 | gi|619293555|gb|GAXD01026583.1| | #N/A | #N/A | 148 | -31.81 | 2 | 21 | 182 | 204 | 19 | Query: 3' cguUCAUCAAAGGCGGCCGGAUg 5' | Ref: 5' ggcGGGAGTGATGCTCGGCCTAc 3' |
| bmo-miR-2796-3p | gi|619319618|gb|GAXD01001131.1| | #N/A | #N/A | 150 | -30.16 | 2 | 22 | 428 | 456 | 26 | Query: 3' cgUUCAU-CAAA---GGCG--GCCGGAUg 5' | Ref: 5' gcAAGGACGTGTGGACCGCTACGGCCTAc 3' |
| bmo-miR-2796-3p | gi|619309063|gb|GAXD01011559.1| | #N/A | #N/A | 158 | -30.77 | 2 | 21 | 113 | 134 | 19 | Query: 3' cguUCAUCAAAGGCGGCCGGAUg 5' | Ref: 5' gacGGGAGGTT-AGCCGGCCTAc 3' |
| bmo-miR-2796-3p | gi|619299699|gb|GAXD01020922.1| | #N/A | #N/A | 160 | -35.03 | 2 | 13 | 31 | 53 | 11 | Query: 3' cguucaucaaaGGCGGCCGGAUg 5' | Ref: 5' gcacgggagccCCGCCGGCCTAc 3' |
| bmo-miR-2796-3p | gi|619299306|gb|GAXD01021315.1| | #N/A | #N/A | 150 | -30.16 | 2 | 22 | 146 | 174 | 26 | Query: 3' cgUUCAU-CAAA---GGCG--GCCGGAUg 5' | Ref: 5' gcAAGGATGTGTGGACCGCTACGGCCTAc 3' |
| bmo-miR-2796-3p | gi|619293556|gb|GAXD01026582.1| | #N/A | #N/A | 148 | -31.81 | 2 | 21 | 200 | 222 | 19 | Query: 3' cguUCAUCAAAGGCGGCCGGAUg 5' | Ref: 5' ggcGGGAGTGATGCTCGGCCTAc 3' |
| bmo-miR-2796-3p | gi|619293555|gb|GAXD01026583.1| | #N/A | #N/A | 148 | -31.81 | 2 | 21 | 182 | 204 | 19 | Query: 3' cguUCAUCAAAGGCGGCCGGAUg 5' | Ref: 5' ggcGGGAGTGATGCTCGGCCTAc 3' |
| tca-miR-2796-3p | gi|619319618|gb|GAXD01001131.1| | #N/A | #N/A | 150 | -30.16 | 2 | 22 | 428 | 456 | 26 | Query: 3' cgUUCAU-CAAA---GGCG--GCCGGAUg 5' | Ref: 5' gcAAGGACGTGTGGACCGCTACGGCCTAc 3' |
| tca-miR-2796-3p | gi|619309063|gb|GAXD01011559.1| | #N/A | #N/A | 158 | -30.77 | 2 | 21 | 113 | 134 | 19 | Query: 3' cguUCAUCAAAGGCGGCCGGAUg 5' | Ref: 5' gacGGGAGGTT-AGCCGGCCTAc 3' |
| tca-miR-2796-3p | gi|619299699|gb|GAXD01020922.1| | #N/A | #N/A | 160 | -35.03 | 2 | 13 | 31 | 53 | 11 | Query: 3' cguucaucaaaGGCGGCCGGAUg 5' | Ref: 5' gcacgggagccCCGCCGGCCTAc 3' |
| tca-miR-2796-3p | gi|619299306|gb|GAXD01021315.1| | #N/A | #N/A | 150 | -30.16 | 2 | 22 | 146 | 174 | 26 | Query: 3' cgUUCAU-CAAA---GGCG--GCCGGAUg 5' | Ref: 5' gcAAGGATGTGTGGACCGCTACGGCCTAc 3' |
| tca-miR-2796-3p | gi|619293556|gb|GAXD01026582.1| | #N/A | #N/A | 148 | -31.81 | 2 | 21 | 200 | 222 | 19 | Query: 3' cguUCAUCAAAGGCGGCCGGAUg 5' | Ref: 5' ggcGGGAGTGATGCTCGGCCTAc 3' |
| tca-miR-2796-3p | gi|619293555|gb|GAXD01026583.1| | #N/A | #N/A | 148 | -31.81 | 2 | 21 | 182 | 204 | 19 | Query: 3' cguUCAUCAAAGGCGGCCGGAUg 5' | Ref: 5' ggcGGGAGTGATGCTCGGCCTAc 3' |
| hme-miR-2796 | gi|619319618|gb|GAXD01001131.1| | #N/A | #N/A | 150 | -30.16 | 2 | 22 | 428 | 456 | 26 | Query: 3' cgUUCAU-CAAA---GGCG--GCCGGAUg 5' | Ref: 5' gcAAGGACGTGTGGACCGCTACGGCCTAc 3' |
| hme-miR-2796 | gi|619309063|gb|GAXD01011559.1| | #N/A | #N/A | 158 | -30.77 | 2 | 21 | 113 | 134 | 19 | Query: 3' cguUCAUCAAAGGCGGCCGGAUg 5' | Ref: 5' gacGGGAGGTT-AGCCGGCCTAc 3' |
| hme-miR-2796 | gi|619299699|gb|GAXD01020922.1| | #N/A | #N/A | 160 | -35.03 | 2 | 13 | 31 | 53 | 11 | Query: 3' cguucaucaaaGGCGGCCGGAUg 5' | Ref: 5' gcacgggagccCCGCCGGCCTAc 3' |
| hme-miR-2796 | gi|619299306|gb|GAXD01021315.1| | #N/A | #N/A | 150 | -30.16 | 2 | 22 | 146 | 174 | 26 | Query: 3' cgUUCAU-CAAA---GGCG--GCCGGAUg 5' | Ref: 5' gcAAGGATGTGTGGACCGCTACGGCCTAc 3' |
| hme-miR-2796 | gi|619293556|gb|GAXD01026582.1| | #N/A | #N/A | 148 | -31.81 | 2 | 21 | 200 | 222 | 19 | Query: 3' cguUCAUCAAAGGCGGCCGGAUg 5' | Ref: 5' ggcGGGAGTGATGCTCGGCCTAc 3' |
| hme-miR-2796 | gi|619293555|gb|GAXD01026583.1| | #N/A | #N/A | 148 | -31.81 | 2 | 21 | 182 | 204 | 19 | Query: 3' cguUCAUCAAAGGCGGCCGGAUg 5' | Ref: 5' ggcGGGAGTGATGCTCGGCCTAc 3' |
| bta-miR-2478 | gi|619295072|gb|GAXD01025066.1| | #N/A | #N/A | 166 | -30.07 | 2 | 19 | 81 | 100 | 17 | Query: 3' acCACAGUCUUCACCCUAUg 5' | Ref: 5' ggGGGTGAGGGGTGGGATAc 3' |
| aae-miR-1175-5p | gi|619295371|gb|GAXD01024767.1| | #N/A | #N/A | 163 | -31.13 | 2 | 21 | 165 | 191 | 24 | Query: 3' gcUACUCUG-----GUGAUGAGGUGAa 5' | Ref: 5' gcGTGAGACTGGCTTACTGCTCCACTc 3' |
| tca-miR-279d-3p | gi|619314075|gb|GAXD01006674.1| | #N/A | #N/A | 186 | -30.45 | 2 | 23 | 112 | 136 | 21 | Query: 3' gauAUCUGCUCAUACCUAGAUCAGu 5' | Ref: 5' cccTAGACGAGTGAGAGTCTAGTCa 3' |
| sme-miR-750-3p | gi|619297187|gb|GAXD01023434.1| | #N/A | #N/A | 185 | -31.25 | 2 | 22 | 221 | 243 | 20 | Query: 3' ucUUGACCUUCUCAAUCUAGACu 5' | Ref: 5' agAAGTGGAGGAGGTAGATCTGc 3' |
| cqu-miR-1175-5p | gi|619295371|gb|GAXD01024767.1| | #N/A | #N/A | 163 | -31.13 | 2 | 21 | 165 | 191 | 24 | Query: 3' gcUACUCUG-----GUGAUGAGGUGAa 5' | Ref: 5' gcGTGAGACTGGCTTACTGCTCCACTc 3' |
| bmo-miR-279c-3p | gi|619314075|gb|GAXD01006674.1| | #N/A | #N/A | 176 | -30.5 | 2 | 21 | 115 | 136 | 19 | Query: 3' guCUGCUCAUACCUAGAUCAGu 5' | Ref: 5' taGACGAGTGAGAGTCTAGTCa 3' |
| mse-miR-279d | gi|619314075|gb|GAXD01006674.1| | #N/A | #N/A | 181 | -30.89 | 2 | 22 | 114 | 136 | 20 | Query: 3' cgUCUGCUCAUACCUAGAUCAGu 5' | Ref: 5' ctAGACGAGTGAGAGTCTAGTCa 3' |
| hme-miR-279d | gi|619314075|gb|GAXD01006674.1| | #N/A | #N/A | 176 | -30.5 | 2 | 21 | 115 | 136 | 19 | Query: 3' guCUGCUCAUACCUAGAUCAGu 5' | Ref: 5' taGACGAGTGAGAGTCTAGTCa 3' |
| mmu-miR-6990-5p | gi|619295993|gb|GAXD01024145.1| | #N/A | #N/A | 179 | -36.5 | 2 | 20 | 340 | 360 | 18 | Query: 3' ucUCGGGACUGAGUGGGACCc 5' | Ref: 5' agGGTCCTAACTCACCCTGGt 3' |
| mmu-miR-6990-5p | gi|619293116|gb|GAXD01027022.1| | #N/A | #N/A | 162 | -31.74 | 2 | 20 | 115 | 139 | 22 | Query: 3' ucUCGGGACUGA----GUGGGACCc 5' | Ref: 5' tcAGCTCGGACTTGAGCACCCTGGg 3' |
| mja-miR-6489-5p | gi|619319285|gb|GAXD01001464.1| | #N/A | #N/A | 164 | -30.91 | 2 | 18 | 90 | 110 | 17 | Query: 3' uucCCGCGGUCA-GGCCACGg 5' | Ref: 5' gccGGCGCCGATGCCGGTGCg 3' |
| mja-miR-6489-5p | gi|619318705|gb|GAXD01002044.1| | #N/A | #N/A | 156 | -30.84 | 2 | 18 | 2596 | 2616 | 17 | Query: 3' uucCCGCGGUC-AGGCCACGg 5' | Ref: 5' ggaGCTGCCAGAGCCGGTGCt 3' |
| mja-miR-6489-5p | gi|619313328|gb|GAXD01007421.1| | #N/A | #N/A | 152 | -30.76 | 2 | 19 | 676 | 697 | 19 | Query: 3' uuCCCG-CGG-UCAGGCCACGg 5' | Ref: 5' atGGGCAGCTAAAACCGGTGCc 3' |
| mja-miR-6489-5p | gi|619307037|gb|GAXD01013585.1| | #N/A | #N/A | 160 | -31.86 | 2 | 19 | 516 | 537 | 19 | Query: 3' uuCCCG-CGG-UCAGGCCACGg 5' | Ref: 5' atGGGCAGCTAAGGCCGGTGCc 3' |
| mja-miR-6489-5p | gi|619306459|gb|GAXD01014163.1| | #N/A | #N/A | 158 | -30.23 | 2 | 19 | 132 | 151 | 17 | Query: 3' uuCCCGCGGUCAGGCCACGg 5' | Ref: 5' ggGCGTGTCCGGCCGGTGCg 3' |
| mja-miR-6489-5p | gi|619304599|gb|GAXD01016023.1| | #N/A | #N/A | 164 | -30.99 | 2 | 17 | 219 | 238 | 15 | Query: 3' uuccCGCGGUCAGGCCACGg 5' | Ref: 5' gttcGCGCTGGCCCGGTGCc 3' |
| mja-miR-6489-5p | gi|619302098|gb|GAXD01018524.1| | #N/A | #N/A | 156 | -31.04 | 2 | 19 | 206 | 228 | 20 | Query: 3' uuCCCGCGGU--CA-GGCCACGg 5' | Ref: 5' gaGGTCGTCACCGTCCCGGTGCc 3' |
| mja-miR-6489-5p | gi|619300480|gb|GAXD01020142.1| | #N/A | #N/A | 174 | -36.12 | 2 | 19 | 488 | 507 | 17 | Query: 3' uuCCCGCGGUCAGGCCACGg 5' | Ref: 5' gtGGGCGTCGGGCCGGTGCt 3' |
| mja-miR-6489-5p | gi|619294511|gb|GAXD01025627.1| | #N/A | #N/A | 173 | -34.48 | 2 | 18 | 1050 | 1069 | 16 | Query: 3' uucCCGCGGUCAGGCCACGg 5' | Ref: 5' tgaGGCGCCGGCCCGGTGCg 3' |
| mja-miR-6489-5p | gi|619293175|gb|GAXD01026963.1| | #N/A | #N/A | 166 | -32.25 | 2 | 19 | 75 | 94 | 17 | Query: 3' uuCCCGCGGUCAGGCCACGg 5' | Ref: 5' caGGGTGCAGGGCCGGTGCa 3' |
| hsa-miR-7156-5p | gi|619311099|gb|GAXD01009650.1| | #N/A | #N/A | 180 | -30.15 | 2 | 22 | 305 | 328 | 21 | Query: 3' agACUGUCGG-UCAAACUCUUGUu 5' | Ref: 5' acTGGCAACTGAGTTTGAGAACAa 3' |
| dme-miR-9373-3p | gi|619307444|gb|GAXD01013178.1| | #N/A | #N/A | 175 | -30.76 | 2 | 20 | 567 | 587 | 18 | Query: 3' ugCUCGACCGGUUCUCGCUAc 5' | Ref: 5' ttGATCTGACCAGGAGCGATg 3' |
| dme-miR-9373-3p | gi|619302182|gb|GAXD01018440.1| | #N/A | #N/A | 158 | -30.01 | 2 | 20 | 155 | 176 | 19 | Query: 3' ugCUCGA-CCGGUUCUCGCUAc 5' | Ref: 5' atGGGCTCGGAGTAGAGCGATg 3' |
| dme-miR-9373-3p | gi|619298442|gb|GAXD01022179.1| | #N/A | #N/A | 162 | -30.42 | 2 | 20 | 202 | 225 | 21 | Query: 3' ugCUCGACCG---GUUCUCGCUAc 5' | Ref: 5' agGGGTTGGCGGTGAAGAGCGATg 3' |
| ame-miR-3049-3p | gi|619318551|gb|GAXD01002198.1| | #N/A | #N/A | 162 | -34.35 | 2 | 21 | 41 | 65 | 22 | Query: 3' ucUGCCUUUCC-UCA--ACCUGCCu 5' | Ref: 5' agGCGGAAGGGTACTCCTGGACGGg 3' |
| ame-miR-3049-3p | gi|619299086|gb|GAXD01021535.1| | #N/A | #N/A | 162 | -34.35 | 2 | 21 | 41 | 65 | 22 | Query: 3' ucUGCCUUUCC-UCA--ACCUGCCu 5' | Ref: 5' agGCGGAAGGGTACTCCTGGACGGg 3' |
| ame-miR-3049-3p | gi|619299084|gb|GAXD01021537.1| | #N/A | #N/A | 162 | -34.35 | 2 | 21 | 41 | 65 | 22 | Query: 3' ucUGCCUUUCC-UCA--ACCUGCCu 5' | Ref: 5' agGCGGAAGGGTACTCCTGGACGGg 3' |
| ame-miR-3049-3p | gi|619298324|gb|GAXD01022297.1| | #N/A | #N/A | 163 | -31.73 | 2 | 21 | 253 | 275 | 20 | Query: 3' ucUGCCUUUCC-UCAACCUGCCu 5' | Ref: 5' agACGGACGGGCCGATGGACGGa 3' |
| ame-miR-3049-3p | gi|619297541|gb|GAXD01023080.1| | #N/A | #N/A | 162 | -31.88 | 2 | 21 | 519 | 543 | 22 | Query: 3' ucUGCCUUUCCUC-A--ACCUGCCu 5' | Ref: 5' agACGGGGAAGAGATGGTGGACGGc 3' |
| ame-miR-3049-3p | gi|619297447|gb|GAXD01023174.1| | #N/A | #N/A | 162 | -32.29 | 2 | 21 | 14 | 34 | 19 | Query: 3' ucUGCCUUUCCUCAACCUGCCu 5' | Ref: 5' cgACGGCTCGGAG-TGGACGGg 3' |
| ame-miR-3049-3p | gi|619293332|gb|GAXD01026806.1| | #N/A | #N/A | 167 | -33.99 | 2 | 21 | 102 | 124 | 20 | Query: 3' ucUGCCUUUCCU-CAACCUGCCu 5' | Ref: 5' caAGGGAGGGGAGGGTGGACGGa 3' |
| ame-miR-3049-3p | gi|619292069|gb|GAXD01028069.1| | #N/A | #N/A | 169 | -30.29 | 2 | 18 | 3 | 24 | 16 | Query: 3' ucugcCUUUCCUCAACCUGCCu 5' | Ref: 5' aaagaGAGAGGGGGTGGACGGg 3' |
| ame-miR-3049-3p | gi|619289381|gb|GAXD01030757.1| | #N/A | #N/A | 167 | -33.99 | 2 | 21 | 102 | 124 | 20 | Query: 3' ucUGCCUUUCCU-CAACCUGCCu 5' | Ref: 5' caAGGGAGGGGAGGGTGGACGGa 3' |
| mja-miR-6493-3p | gi|619318364|gb|GAXD01002385.1| | #N/A | #N/A | 164 | -30.29 | 2 | 23 | 2077 | 2097 | 21 | Query: 3' auUGCGAGUCGCGCCAAAGGGGGa 5' | Ref: 5' ttAC-CTCAGT-C-GTTTCCCCCt 3' |
| mja-miR-6493-3p | gi|619317896|gb|GAXD01002853.1| | #N/A | #N/A | 177 | -33.11 | 2 | 23 | 1194 | 1218 | 22 | Query: 3' auUGCG-AGUCGCGCCAAAGGGGGa 5' | Ref: 5' ttAGGCTTCGGCCTGGTTTCCCCCa 3' |
| mja-miR-6493-3p | gi|619308669|gb|GAXD01011953.1| | #N/A | #N/A | 163 | -32.99 | 2 | 18 | 91 | 116 | 18 | Query: 3' auugcgaGUCGCG-CC-AAAGGGGGa 5' | Ref: 5' gacatgaCAGTGCTGGATTTCCCCCt 3' |
| mja-miR-6493-3p | gi|619304355|gb|GAXD01016267.1| | #N/A | #N/A | 164 | -33.08 | 2 | 23 | 115 | 141 | 24 | Query: 3' auUGCGAGU-CGCG--CCAAAGGGGGa 5' | Ref: 5' tcACGTTCGCGCGCTTTCTTTCCCCCg 3' |
| mja-miR-6493-3p | gi|619301469|gb|GAXD01019153.1| | #N/A | #N/A | 164 | -31.47 | 2 | 23 | 69 | 89 | 21 | Query: 3' auUGCGAGUCGCGCCAAAGGGGGa 5' | Ref: 5' agAC-CTC-GTG-GGTTTCCCCCa 3' |
| mja-miR-6493-3p | gi|619299055|gb|GAXD01021566.1| | #N/A | #N/A | 158 | -30.47 | 2 | 22 | 1340 | 1363 | 21 | Query: 3' auuGCGAGUCGCGCC-AAAGGGGGa 5' | Ref: 5' ggtCGCTGTGC-CGGAATTCCCCCt 3' |
| mja-miR-6493-3p | gi|619295049|gb|GAXD01025089.1| | #N/A | #N/A | 153 | -33.15 | 2 | 23 | 183 | 210 | 25 | Query: 3' auUGCGAGUCGCGCCA----AAGGGGGa 5' | Ref: 5' gaGCGCTGCTAGCGGTCACCTTCCCCCt 3' |
| mja-miR-6493-3p | gi|619292932|gb|GAXD01027206.1| | #N/A | #N/A | 165 | -30.21 | 2 | 22 | 39 | 62 | 20 | Query: 3' auuGCGAGUCGCGCCAAAGGGGGa 5' | Ref: 5' gctCTCTCGGTGGTGGTTCCCCCg 3' |
| mja-miR-6493-3p | gi|619291181|gb|GAXD01028957.1| | #N/A | #N/A | 161 | -32.13 | 2 | 23 | 137 | 162 | 23 | Query: 3' auUGCGAGUCGCGCC--AAAGGGGGa 5' | Ref: 5' cgAGGCTCCTTGCGGGACTTCCCCCc 3' |
| efu-miR-9198a | gi|619300701|gb|GAXD01019921.1| | #N/A | #N/A | 156 | -30.1 | 2 | 22 | 2566 | 2591 | 23 | Query: 3' agUGUAGGUGACUG---UCACGGUUc 5' | Ref: 5' gcGCCTGCGCTGGCCAGGGTGCCAAg 3' |
| efu-miR-9198a | gi|619300700|gb|GAXD01019922.1| | #N/A | #N/A | 156 | -30.1 | 2 | 22 | 2496 | 2521 | 23 | Query: 3' agUGUAGGUGACUG---UCACGGUUc 5' | Ref: 5' gcGCCTGCGCTGGCCAGGGTGCCAAg 3' |
| efu-miR-9198a | gi|619297400|gb|GAXD01023221.1| | #N/A | #N/A | 172 | -30.01 | 2 | 17 | 467 | 489 | 15 | Query: 3' aguguagGUGACUGUCACGGUUc 5' | Ref: 5' tttgtaaTACTGATAGTGCCAAg 3' |
| efu-miR-9198a | gi|619295243|gb|GAXD01024895.1| | #N/A | #N/A | 181 | -35.62 | 2 | 18 | 289 | 311 | 16 | Query: 3' aguguaGGUGACUGUCACGGUUc 5' | Ref: 5' cctccgCCATTGACAGTGCCAAg 3' |
| bta-miR-2379 | gi|619315286|gb|GAXD01005463.1| | #N/A | #N/A | 192 | -31.76 | 2 | 21 | 594 | 615 | 19 | Query: 3' uuUUAUAGAAGAGGUCGUCGGa 5' | Ref: 5' ggAATATTTTCTCTAGCAGCCc 3' |
| mmu-miR-3086-3p | gi|619295767|gb|GAXD01024371.1| | #N/A | #N/A | 166 | -32.4 | 2 | 15 | 1216 | 1237 | 13 | Query: 3' gaaucugaCAUCCGAGUAACCc 5' | Ref: 5' ctgcttggGTGGGCTCATTGGg 3' |
| mja-miR-6489-3p | gi|619319912|gb|GAXD01000837.1| | #N/A | #N/A | 165 | -31.61 | 2 | 22 | 636 | 656 | 20 | Query: 3' ggUCGAACCUGUGGAAAGGCAGc 5' | Ref: 5' ctGGC-TGAAC-CCTTTCCGTCg 3' |
| mja-miR-6489-3p | gi|619314338|gb|GAXD01006411.1| | #N/A | #N/A | 188 | -30.92 | 2 | 21 | 380 | 402 | 19 | Query: 3' gguCGAACCUGUGGAAAGGCAGc 5' | Ref: 5' agtGCTTGGGCACATTTCCGTCc 3' |
| mja-miR-6489-3p | gi|619299926|gb|GAXD01020696.1| | #N/A | #N/A | 155 | -30.08 | 2 | 16 | 650 | 672 | 14 | Query: 3' ggucgaacCUGUGGAAAGGCAGc 5' | Ref: 5' acgccggcGGCAGGTTTCCGTCg 3' |
| ppc-miR-71a | gi|619318251|gb|GAXD01002498.1| | #N/A | #N/A | 179 | -30.99 | 2 | 24 | 402 | 426 | 22 | Query: 3' gcAGAGUGAUGGUUGUCCAGAAAGu 5' | Ref: 5' tcTCTATTTACCGGCCGGTCTTTCa 3' |
| tca-miR-2944b-3p | gi|619310335|gb|GAXD01010348.1| | #N/A | #N/A | 172 | -32.33 | 2 | 22 | 432 | 457 | 23 | Query: 3' caUUCCGUU---GAUGCCGACACUAu 5' | Ref: 5' tgAGGGCAGTGTTTATGGCTGTGATg 3' |
| gga-miR-7475-5p | gi|619320680|gb|GAXD01000069.1| | #N/A | #N/A | 145 | -33.42 | 2 | 19 | 88 | 108 | 18 | Query: 3' ccUCCCGCGCC-GCCGCCGCc 5' | Ref: 5' ggGGAGAGGGGAAGGCGGCGg 3' |
| gga-miR-7475-5p | gi|619320659|gb|GAXD01000090.1| | #N/A | #N/A | 167 | -36.18 | 2 | 17 | 1977 | 1998 | 17 | Query: 3' ccucCCGCGCCG--CCGCCGCc 5' | Ref: 5' gcgtGGCGCGGCGTGGCGGCGc 3' |
| gga-miR-7475-5p | gi|619320409|gb|GAXD01000340.1| | #N/A | #N/A | 155 | -34.84 | 2 | 18 | 1306 | 1327 | 18 | Query: 3' ccuCCCGCG-CCG-CCGCCGCc 5' | Ref: 5' ggcGTGCGTGGGCGGGCGGCGg 3' |
| gga-miR-7475-5p | gi|619320154|gb|GAXD01000595.1| | #N/A | #N/A | 164 | -37.19 | 2 | 18 | 1735 | 1755 | 17 | Query: 3' ccuCCCG-CGCCGCCGCCGCc 5' | Ref: 5' tctGGGCGGTGGAGGCGGCGg 3' |
| gga-miR-7475-5p | gi|619320154|gb|GAXD01000595.1| | #N/A | #N/A | 159 | -30.36 | 2 | 17 | 1231 | 1251 | 16 | Query: 3' ccucCCGC-GCCGCCGCCGCc 5' | Ref: 5' cggtGGTGACGTCGGCGGCGc 3' |
| gga-miR-7475-5p | gi|619320130|gb|GAXD01000619.1| | #N/A | #N/A | 159 | -30.36 | 2 | 17 | 1231 | 1251 | 16 | Query: 3' ccucCCGC-GCCGCCGCCGCc 5' | Ref: 5' cggtGGTGACGTCGGCGGCGc 3' |
| gga-miR-7475-5p | gi|619319667|gb|GAXD01001082.1| | #N/A | #N/A | 165 | -34.03 | 2 | 19 | 531 | 551 | 18 | Query: 3' ccUCCCGC-GCCGCCGCCGCc 5' | Ref: 5' acACGGAGCCGGCGGCGGCGa 3' |
| gga-miR-7475-5p | gi|619319667|gb|GAXD01001082.1| | #N/A | #N/A | 155 | -31.79 | 2 | 17 | 798 | 818 | 16 | Query: 3' ccucCCG-CGCCGCCGCCGCc 5' | Ref: 5' tcttGGCGGCGAGGGCGGCGg 3' |
| gga-miR-7475-5p | gi|619319667|gb|GAXD01001082.1| | #N/A | #N/A | 152 | -30.58 | 2 | 18 | 1575 | 1595 | 17 | Query: 3' ccuCCCG-CGCCGCCGCCGCc 5' | Ref: 5' cctGGGCAACCTCGGCGGCGg 3' |
| gga-miR-7475-5p | gi|619319666|gb|GAXD01001083.1| | #N/A | #N/A | 168 | -41.46 | 2 | 19 | 1415 | 1436 | 19 | Query: 3' ccUC-CCGC-GCCGCCGCCGCc 5' | Ref: 5' gcAGCGGCGTCGGTGGCGGCGg 3' |
| gga-miR-7475-5p | gi|619319666|gb|GAXD01001083.1| | #N/A | #N/A | 165 | -34.03 | 2 | 19 | 531 | 551 | 18 | Query: 3' ccUCCCGC-GCCGCCGCCGCc 5' | Ref: 5' acACGGAGCCGGCGGCGGCGa 3' |
| gga-miR-7475-5p | gi|619319666|gb|GAXD01001083.1| | #N/A | #N/A | 165 | -39.35 | 2 | 18 | 550 | 569 | 16 | Query: 3' ccuCCCGCGCCGCCGCCGCc 5' | Ref: 5' gatGGGGATGGCGGCGGCGg 3' |
| gga-miR-7475-5p | gi|619319666|gb|GAXD01001083.1| | #N/A | #N/A | 155 | -31.79 | 2 | 17 | 798 | 818 | 16 | Query: 3' ccucCCG-CGCCGCCGCCGCc 5' | Ref: 5' tcttGGCGGCGAGGGCGGCGg 3' |
| gga-miR-7475-5p | gi|619319666|gb|GAXD01001083.1| | #N/A | #N/A | 153 | -33.3 | 2 | 19 | 1383 | 1403 | 18 | Query: 3' ccUCCCGC-GCCGCCGCCGCc 5' | Ref: 5' tgATGGCGTTCTCGGCGGCGg 3' |
| gga-miR-7475-5p | gi|619319666|gb|GAXD01001083.1| | #N/A | #N/A | 152 | -30.43 | 2 | 19 | 1656 | 1679 | 21 | Query: 3' ccUCCCGCG---CC-GCCGCCGCc 5' | Ref: 5' tgATGGCGTTGAGGCCGGCGGCGa 3' |
| gga-miR-7475-5p | gi|619319666|gb|GAXD01001083.1| | #N/A | #N/A | 151 | -31.12 | 2 | 12 | 1633 | 1652 | 10 | Query: 3' ccucccgcgCCGCCGCCGCc 5' | Ref: 5' gctaccgagGGTGGCGGCGg 3' |
| gga-miR-7475-5p | gi|619319601|gb|GAXD01001148.1| | #N/A | #N/A | 169 | -36.1 | 2 | 19 | 445 | 465 | 18 | Query: 3' ccUCCCG-CGCCGCCGCCGCc 5' | Ref: 5' cgATGGCGGTGGCGGCGGCGc 3' |
| gga-miR-7475-5p | gi|619319601|gb|GAXD01001148.1| | #N/A | #N/A | 157 | -33.01 | 2 | 19 | 469 | 489 | 18 | Query: 3' ccUCCCGCGCCG-CCGCCGCc 5' | Ref: 5' tgATGGCGGAGCGGGCGGCGg 3' |
| gga-miR-7475-5p | gi|619319522|gb|GAXD01001227.1| | #N/A | #N/A | 177 | -38.39 | 2 | 18 | 407 | 426 | 16 | Query: 3' ccuCCCGCGCCGCCGCCGCc 5' | Ref: 5' gacGGCCGCGGCGGCGGCGc 3' |
| gga-miR-7475-5p | gi|619319522|gb|GAXD01001227.1| | #N/A | #N/A | 161 | -35.54 | 2 | 19 | 211 | 231 | 18 | Query: 3' ccUCCCG-CGCCGCCGCCGCc 5' | Ref: 5' cgAGGCCAGCGTTGGCGGCGg 3' |
| gga-miR-7475-5p | gi|619319522|gb|GAXD01001227.1| | #N/A | #N/A | 157 | -35.06 | 2 | 15 | 376 | 396 | 14 | Query: 3' ccucccGC-GCCGCCGCCGCc 5' | Ref: 5' cggcaaCGTTGGCGGCGGCGg 3' |
| gga-miR-7475-5p | gi|619319522|gb|GAXD01001227.1| | #N/A | #N/A | 157 | -34.63 | 2 | 17 | 418 | 435 | 15 | Query: 3' ccucCCGCGCCGCCGCCGCc 5' | Ref: 5' cggcGGCGCG--GGCGGCGg 3' |
| gga-miR-7475-5p | gi|619319285|gb|GAXD01001464.1| | #N/A | #N/A | 164 | -32.97 | 2 | 19 | 325 | 343 | 17 | Query: 3' ccUCCCGCGCCGCCGCCGCc 5' | Ref: 5' ccAAGGC-CGGTGGCGGCGc 3' |
| gga-miR-7475-5p | gi|619319285|gb|GAXD01001464.1| | #N/A | #N/A | 159 | -31.5 | 2 | 17 | 65 | 85 | 16 | Query: 3' ccucCCGCGCC-GCCGCCGCc 5' | Ref: 5' aggtGGCGAGGTTGGCGGCGc 3' |
| gga-miR-7475-5p | gi|619319285|gb|GAXD01001464.1| | #N/A | #N/A | 157 | -32.94 | 2 | 14 | 186 | 205 | 12 | Query: 3' ccucccgCGCCGCCGCCGCc 5' | Ref: 5' gatgccgGCGCCGGCGGCGg 3' |
| gga-miR-7475-5p | gi|619319285|gb|GAXD01001464.1| | #N/A | #N/A | 151 | -31.61 | 2 | 12 | 12 | 31 | 10 | Query: 3' ccucccgcgCCGCCGCCGCc 5' | Ref: 5' cacgcccagGGTGGCGGCGg 3' |
| gga-miR-7475-5p | gi|619319144|gb|GAXD01001605.1| | #N/A | #N/A | 164 | -30.72 | 2 | 19 | 372 | 390 | 17 | Query: 3' ccUCCCGCGCCGCCGCCGCc 5' | Ref: 5' gaAGAGTGCGG-GGCGGCGc 3' |
| gga-miR-7475-5p | gi|619319064|gb|GAXD01001685.1| | #N/A | #N/A | 166 | -33.09 | 2 | 19 | 1441 | 1460 | 17 | Query: 3' ccUCCCGCGCCGCCGCCGCc 5' | Ref: 5' agGGGGCTCTGTGGCGGCGc 3' |
| gga-miR-7475-5p | gi|619319064|gb|GAXD01001685.1| | #N/A | #N/A | 161 | -31.65 | 2 | 18 | 3120 | 3139 | 16 | Query: 3' ccuCCCGCGCCGCCGCCGCc 5' | Ref: 5' gttGGGTGTAGAGGCGGCGg 3' |
| gga-miR-7475-5p | gi|619318980|gb|GAXD01001769.1| | #N/A | #N/A | 154 | -31.51 | 2 | 17 | 256 | 274 | 15 | Query: 3' ccucCCGCGCCGCCGCCGCc 5' | Ref: 5' gtccGGCGT-CCGGCGGCGg 3' |
| gga-miR-7475-5p | gi|619318573|gb|GAXD01002176.1| | #N/A | #N/A | 156 | -36.29 | 2 | 17 | 453 | 472 | 15 | Query: 3' ccucCCGCGCCGCCGCCGCc 5' | Ref: 5' ctccGACGGCGCGGCGGCGg 3' |
| gga-miR-7475-5p | gi|619318565|gb|GAXD01002184.1| | #N/A | #N/A | 166 | -30.14 | 2 | 19 | 611 | 630 | 17 | Query: 3' ccUCCCGCGCCGCCGCCGCc 5' | Ref: 5' gaAGGCAGCTGCGGCGGCGc 3' |
| gga-miR-7475-5p | gi|619318564|gb|GAXD01002185.1| | #N/A | #N/A | 159 | -35.79 | 2 | 16 | 178 | 197 | 14 | Query: 3' ccuccCGCGCCGCCGCCGCc 5' | Ref: 5' gagctGGGCGTCGGCGGCGg 3' |
| gga-miR-7475-5p | gi|619318564|gb|GAXD01002185.1| | #N/A | #N/A | 157 | -31.48 | 2 | 19 | 819 | 839 | 18 | Query: 3' ccUCCC-GCGCCGCCGCCGCc 5' | Ref: 5' tgAGGGCCGATGAGGCGGCGc 3' |
| gga-miR-7475-5p | gi|619318195|gb|GAXD01002554.1| | #N/A | #N/A | 165 | -32.84 | 2 | 18 | 742 | 761 | 16 | Query: 3' ccuCCCGCGCCGCCGCCGCc 5' | Ref: 5' gacGTGGGTGGCGGCGGCGc 3' |
| gga-miR-7475-5p | gi|619318119|gb|GAXD01002630.1| | #N/A | #N/A | 164 | -37.19 | 2 | 18 | 1735 | 1755 | 17 | Query: 3' ccuCCCG-CGCCGCCGCCGCc 5' | Ref: 5' tctGGGCGGTGGAGGCGGCGg 3' |
| gga-miR-7475-5p | gi|619318119|gb|GAXD01002630.1| | #N/A | #N/A | 163 | -34.77 | 2 | 17 | 1759 | 1779 | 16 | Query: 3' ccucCCGC-GCCGCCGCCGCc 5' | Ref: 5' tggcGGCGTTGGTGGCGGCGc 3' |
| gga-miR-7475-5p | gi|619318119|gb|GAXD01002630.1| | #N/A | #N/A | 159 | -30.36 | 2 | 17 | 1231 | 1251 | 16 | Query: 3' ccucCCGC-GCCGCCGCCGCc 5' | Ref: 5' cggtGGTGACGTCGGCGGCGc 3' |
| gga-miR-7475-5p | gi|619318100|gb|GAXD01002649.1| | #N/A | #N/A | 167 | -36.18 | 2 | 17 | 553 | 574 | 17 | Query: 3' ccucCCGCGCCG--CCGCCGCc 5' | Ref: 5' gcgtGGCGCGGCGTGGCGGCGc 3' |
| gga-miR-7475-5p | gi|619317723|gb|GAXD01003026.1| | #N/A | #N/A | 145 | -30.98 | 2 | 18 | 10 | 29 | 16 | Query: 3' ccuCCCGCGCCGCCGCCGCc 5' | Ref: 5' cgcGGCCCCCAGGGCGGCGg 3' |
| gga-miR-7475-5p | gi|619317621|gb|GAXD01003128.1| | #N/A | #N/A | 167 | -36.58 | 2 | 17 | 1874 | 1894 | 16 | Query: 3' ccucCCGC-GCCGCCGCCGCc 5' | Ref: 5' cggcGGCGTTGGCGGCGGCGc 3' |
| gga-miR-7475-5p | gi|619317604|gb|GAXD01003145.1| | #N/A | #N/A | 172 | -38.68 | 2 | 19 | 358 | 379 | 19 | Query: 3' ccUCC-CGC-GCCGCCGCCGCc 5' | Ref: 5' gcAGGTGCGACGGCGGCGGCGc 3' |
| gga-miR-7475-5p | gi|619316799|gb|GAXD01003950.1| | #N/A | #N/A | 155 | -30.11 | 2 | 18 | 121 | 139 | 16 | Query: 3' ccuCCCGCGCCGCCGCCGCc 5' | Ref: 5' gtcGGGGTCGG-GGCGGCGa 3' |
| gga-miR-7475-5p | gi|619316572|gb|GAXD01004177.1| | #N/A | #N/A | 159 | -33.52 | 2 | 18 | 167 | 190 | 20 | Query: 3' ccuCCCGCG---CCG-CCGCCGCc 5' | Ref: 5' cacGGGCGCAGAGGCTGGCGGCGt 3' |
| gga-miR-7475-5p | gi|619315613|gb|GAXD01005136.1| | #N/A | #N/A | 166 | -38.22 | 2 | 17 | 203 | 220 | 15 | Query: 3' ccucCCGCGCCGCCGCCGCc 5' | Ref: 5' gggcGGCGCGG-GGCGGCG- 3' |
| gga-miR-7475-5p | gi|619315613|gb|GAXD01005136.1| | #N/A | #N/A | 161 | -36.72 | 2 | 19 | 190 | 211 | 19 | Query: 3' ccUCCCGCGCCG--CCGCCGCc 5' | Ref: 5' acGGGGCGGGGTGGGGCGGCGc 3' |
| gga-miR-7475-5p | gi|619314960|gb|GAXD01005789.1| | #N/A | #N/A | 160 | -35.57 | 2 | 18 | 547 | 568 | 19 | Query: 3' ccuCCCGCGCCG---CCGCCGCc 5' | Ref: 5' ggcGGGCGGGGCCAGGGCGGCG- 3' |
| gga-miR-7475-5p | gi|619314770|gb|GAXD01005979.1| | #N/A | #N/A | 160 | -32.91 | 2 | 18 | 303 | 323 | 17 | Query: 3' ccuCCCG-CGCCGCCGCCGCc 5' | Ref: 5' gctGGACGGCCGCGGCGGCGc 3' |
| gga-miR-7475-5p | gi|619313509|gb|GAXD01007240.1| | #N/A | #N/A | 151 | -38.61 | 2 | 17 | 284 | 309 | 21 | Query: 3' ccucCCGCGCCG------CCGCCGCc 5' | Ref: 5' gtgaGGCGCGGCGGACAGGGCGGCGa 3' |
| gga-miR-7475-5p | gi|619313383|gb|GAXD01007366.1| | #N/A | #N/A | 149 | -31.53 | 2 | 16 | 6 | 27 | 16 | Query: 3' ccuccCGC-GCC-GCCGCCGCc 5' | Ref: 5' cgtacGCGACGGAGGGCGGCGg 3' |
| gga-miR-7475-5p | gi|619312513|gb|GAXD01008236.1| | #N/A | #N/A | 167 | -36.58 | 2 | 17 | 958 | 978 | 16 | Query: 3' ccucCCGC-GCCGCCGCCGCc 5' | Ref: 5' cggcGGCGTTGGCGGCGGCGc 3' |
| gga-miR-7475-5p | gi|619312512|gb|GAXD01008237.1| | #N/A | #N/A | 167 | -36.58 | 2 | 17 | 1872 | 1892 | 16 | Query: 3' ccucCCGC-GCCGCCGCCGCc 5' | Ref: 5' cggcGGCGTTGGCGGCGGCGc 3' |
| gga-miR-7475-5p | gi|619312447|gb|GAXD01008302.1| | #N/A | #N/A | 152 | -33.74 | 2 | 18 | 480 | 502 | 19 | Query: 3' ccuCCCG---CGCCGCCGCCGCc 5' | Ref: 5' ggtGGGCCTAGAGGAGGCGGCGg 3' |
| gga-miR-7475-5p | gi|619312446|gb|GAXD01008303.1| | #N/A | #N/A | 174 | -43.71 | 2 | 19 | 561 | 580 | 17 | Query: 3' ccUCCCGCGCCGCCGCCGCc 5' | Ref: 5' cgAGGCGGCGGCGGCGGCGg 3' |
| gga-miR-7475-5p | gi|619312446|gb|GAXD01008303.1| | #N/A | #N/A | 152 | -33.74 | 2 | 18 | 480 | 502 | 19 | Query: 3' ccuCCCG---CGCCGCCGCCGCc 5' | Ref: 5' ggtGGGCCTAGAGGAGGCGGCGg 3' |
| gga-miR-7475-5p | gi|619312301|gb|GAXD01008448.1| | #N/A | #N/A | 156 | -30.19 | 2 | 17 | 228 | 247 | 15 | Query: 3' ccucCCGCGCCGCCGCCGCc 5' | Ref: 5' gggcGGCTGGGAGGCGGCGc 3' |
| gga-miR-7475-5p | gi|619312089|gb|GAXD01008660.1| | #N/A | #N/A | 165 | -39.57 | 2 | 19 | 204 | 224 | 18 | Query: 3' ccUC-CCGCGCCGCCGCCGCc 5' | Ref: 5' agAGCGGTGTCGCGGCGGCGg 3' |
| gga-miR-7475-5p | gi|619310822|gb|GAXD01009927.1| | #N/A | #N/A | 158 | -38.09 | 2 | 17 | 54 | 76 | 18 | Query: 3' ccucCCG-CGCC--GCCGCCGCc 5' | Ref: 5' cggcGGCAGCGGCCCGGCGGCGg 3' |
| gga-miR-7475-5p | gi|619310817|gb|GAXD01009932.1| | #N/A | #N/A | 161 | -32.68 | 2 | 19 | 38 | 58 | 18 | Query: 3' ccUCCCGCG-CCGCCGCCGCc 5' | Ref: 5' ccAGGGCGTACTCGGCGGCGa 3' |
| gga-miR-7475-5p | gi|619310468|gb|GAXD01010243.1| | #N/A | #N/A | 160 | -31.1 | 2 | 18 | 158 | 178 | 17 | Query: 3' ccuCCCGCG-CCGCCGCCGCc 5' | Ref: 5' ggtGTGCTCTGGCGGCGGCGc 3' |
| gga-miR-7475-5p | gi|619310436|gb|GAXD01010274.1| | #N/A | #N/A | 160 | -31.76 | 2 | 17 | 260 | 279 | 15 | Query: 3' ccucCCGCGCCGCCGCCGCc 5' | Ref: 5' agctGGTCCGGAGGCGGCGg 3' |
| gga-miR-7475-5p | gi|619310407|gb|GAXD01010302.1| | #N/A | #N/A | 161 | -30.1 | 2 | 19 | 203 | 223 | 18 | Query: 3' ccUCC-CGCGCCGCCGCCGCc 5' | Ref: 5' ctAGGTACTCGGTGGCGGCGc 3' |
| gga-miR-7475-5p | gi|619309284|gb|GAXD01011338.1| | #N/A | #N/A | 158 | -30.29 | 2 | 16 | 67 | 87 | 15 | Query: 3' ccuccCGC-GCCGCCGCCGCc 5' | Ref: 5' cgatcGTGACGGTGGCGGCGa 3' |
| gga-miR-7475-5p | gi|619308706|gb|GAXD01011916.1| | #N/A | #N/A | 158 | -32.9 | 2 | 15 | 1 | 14 | 13 | Query: 3' ccucccGCGCCGCCGCCGCc 5' | Ref: 5' ------TGGGGCGGCGGCGg 3' |
| gga-miR-7475-5p | gi|619308539|gb|GAXD01012083.1| | #N/A | #N/A | 162 | -32.63 | 2 | 19 | 114 | 133 | 17 | Query: 3' ccUCCCGCGCCGCCGCCGCc 5' | Ref: 5' tgGGAGCGCGCAGGCGGCGc 3' |
| gga-miR-7475-5p | gi|619308067|gb|GAXD01012555.1| | #N/A | #N/A | 168 | -42.19 | 2 | 18 | 14 | 34 | 17 | Query: 3' ccuCCCGC-GCCGCCGCCGCc 5' | Ref: 5' cacGGGCGAGGGCGGCGGCGg 3' |
| gga-miR-7475-5p | gi|619308047|gb|GAXD01012575.1| | #N/A | #N/A | 160 | -34.56 | 2 | 13 | 191 | 210 | 11 | Query: 3' ccucccgcGCCGCCGCCGCc 5' | Ref: 5' cacattgaCGGCGGCGGCGg 3' |
| gga-miR-7475-5p | gi|619307736|gb|GAXD01012886.1| | #N/A | #N/A | 165 | -34.03 | 2 | 19 | 498 | 518 | 18 | Query: 3' ccUCCCGC-GCCGCCGCCGCc 5' | Ref: 5' acACGGAGCCGGCGGCGGCGa 3' |
| gga-miR-7475-5p | gi|619307736|gb|GAXD01012886.1| | #N/A | #N/A | 159 | -30.04 | 2 | 17 | 573 | 593 | 16 | Query: 3' ccucCCGC-GCCGCCGCCGCc 5' | Ref: 5' cggcGGTGTCGACGGCGGCGt 3' |
| gga-miR-7475-5p | gi|619307736|gb|GAXD01012886.1| | #N/A | #N/A | 156 | -32.36 | 2 | 18 | 558 | 578 | 17 | Query: 3' ccuCCCG-CGCCGCCGCCGCc 5' | Ref: 5' tgcGGCCGGCGTTGGCGGCGg 3' |
| gga-miR-7475-5p | gi|619307736|gb|GAXD01012886.1| | #N/A | #N/A | 155 | -31.79 | 2 | 17 | 765 | 785 | 16 | Query: 3' ccucCCG-CGCCGCCGCCGCc 5' | Ref: 5' tcttGGCGGCGAGGGCGGCGg 3' |
| gga-miR-7475-5p | gi|619307736|gb|GAXD01012886.1| | #N/A | #N/A | 152 | -30.58 | 2 | 18 | 1542 | 1562 | 17 | Query: 3' ccuCCCG-CGCCGCCGCCGCc 5' | Ref: 5' cctGGGCAACCTCGGCGGCGg 3' |
| gga-miR-7475-5p | gi|619307591|gb|GAXD01013031.1| | #N/A | #N/A | 171 | -35.98 | 2 | 16 | 94 | 113 | 14 | Query: 3' ccuccCGCGCCGCCGCCGCc 5' | Ref: 5' tcacaGTGCGGCGGCGGCGa 3' |
| gga-miR-7475-5p | gi|619307591|gb|GAXD01013031.1| | #N/A | #N/A | 157 | -32.31 | 2 | 14 | 11 | 30 | 12 | Query: 3' ccucccgCGCCGCCGCCGCc 5' | Ref: 5' gcgtccaGAGGCGGCGGCGg 3' |
| gga-miR-7475-5p | gi|619307475|gb|GAXD01013147.1| | #N/A | #N/A | 148 | -32.85 | 2 | 18 | 272 | 292 | 17 | Query: 3' ccuCCCGCGC-CGCCGCCGCc 5' | Ref: 5' accGGGATAGTGTGGCGGCGg 3' |
| gga-miR-7475-5p | gi|619307086|gb|GAXD01013536.1| | #N/A | #N/A | 177 | -41.9 | 2 | 18 | 228 | 247 | 16 | Query: 3' ccuCCCGCGCCGCCGCCGCc 5' | Ref: 5' atcGCGCGCGGCGGCGGCGg 3' |
| gga-miR-7475-5p | gi|619306347|gb|GAXD01014275.1| | #N/A | #N/A | 155 | -30.36 | 2 | 12 | 11 | 30 | 10 | Query: 3' ccucccgcgCCGCCGCCGCc 5' | Ref: 5' cccctccagGGCGGCGGCGg 3' |
| gga-miR-7475-5p | gi|619306254|gb|GAXD01014368.1| | #N/A | #N/A | 159 | -32.11 | 2 | 17 | 84 | 104 | 16 | Query: 3' ccucCCGC-GCCGCCGCCGCc 5' | Ref: 5' cgttGGAGTTGGCGGCGGCGt 3' |
| gga-miR-7475-5p | gi|619306206|gb|GAXD01014416.1| | #N/A | #N/A | 158 | -34.48 | 2 | 19 | 104 | 123 | 17 | Query: 3' ccUCCCGCGCCGCCGCCGCc 5' | Ref: 5' gcAGGCCGGACCGGCGGCGg 3' |
| gga-miR-7475-5p | gi|619305431|gb|GAXD01015191.1| | #N/A | #N/A | 153 | -32.78 | 2 | 19 | 140 | 160 | 18 | Query: 3' ccUCCCGCGCCG-CCGCCGCc 5' | Ref: 5' cgGGAGCTCCGCTGGCGGCGg 3' |
| gga-miR-7475-5p | gi|619305362|gb|GAXD01015260.1| | #N/A | #N/A | 166 | -31.16 | 2 | 19 | 202 | 221 | 17 | Query: 3' ccUCCCGCGCCGCCGCCGCc 5' | Ref: 5' gaAGGCGGCGTCGGCGGCGt 3' |
| gga-miR-7475-5p | gi|619304983|gb|GAXD01015639.1| | #N/A | #N/A | 156 | -32.42 | 2 | 19 | 175 | 196 | 19 | Query: 3' ccUCC-CGCGCC-GCCGCCGCc 5' | Ref: 5' cgAGGCCCGGGGTCGGCGGCGc 3' |
| gga-miR-7475-5p | gi|619304202|gb|GAXD01016420.1| | #N/A | #N/A | 154 | -30.11 | 2 | 15 | 16 | 35 | 13 | Query: 3' ccucccGCGCCGCCGCCGCc 5' | Ref: 5' agcgaaCCCGACGGCGGCGg 3' |
| gga-miR-7475-5p | gi|619304104|gb|GAXD01016518.1| | #N/A | #N/A | 165 | -37.01 | 2 | 19 | 49 | 71 | 20 | Query: 3' ccUCCCG---CGCCGCCGCCGCc 5' | Ref: 5' aaGGGGCAATGCGGTGGCGGCGc 3' |
| gga-miR-7475-5p | gi|619304020|gb|GAXD01016602.1| | #N/A | #N/A | 159 | -34.06 | 2 | 19 | 459 | 478 | 18 | Query: 3' ccUC-CCGCGCCGCCGCCGCc 5' | Ref: 5' gcAGCGGCCCGG-GGCGGCGg 3' |
| gga-miR-7475-5p | gi|619303946|gb|GAXD01016676.1| | #N/A | #N/A | 164 | -35.19 | 2 | 19 | 32 | 50 | 17 | Query: 3' ccUCCCGCGCCGCCGCCGCc 5' | Ref: 5' gcAGGGC-TGACGGCGGCGg 3' |
| gga-miR-7475-5p | gi|619303415|gb|GAXD01017207.1| | #N/A | #N/A | 155 | -30.98 | 2 | 12 | 107 | 126 | 10 | Query: 3' ccucccgcgCCGCCGCCGCc 5' | Ref: 5' cgccaaaaaGGCGGCGGCGg 3' |
| gga-miR-7475-5p | gi|619302199|gb|GAXD01018423.1| | #N/A | #N/A | 153 | -33.2 | 2 | 16 | 156 | 178 | 17 | Query: 3' ccuccCG-CGCCG--CCGCCGCc 5' | Ref: 5' ggactGCGGCGGCAGGGCGGCGc 3' |
| gga-miR-7475-5p | gi|619301828|gb|GAXD01018794.1| | #N/A | #N/A | 150 | -32.05 | 2 | 17 | 220 | 238 | 15 | Query: 3' ccucCCGCGCCGCCGCCGCc 5' | Ref: 5' ccgcGGCG-GAAGGCGGCGt 3' |
| gga-miR-7475-5p | gi|619300270|gb|GAXD01020352.1| | #N/A | #N/A | 165 | -34.95 | 2 | 19 | 724 | 744 | 18 | Query: 3' ccUCCCGCGCCG-CCGCCGCc 5' | Ref: 5' ggACGGCGAGGCTGGCGGCGa 3' |
| gga-miR-7475-5p | gi|619299787|gb|GAXD01020834.1| | #N/A | #N/A | 159 | -31.8 | 2 | 17 | 13 | 34 | 17 | Query: 3' ccucCCG--CGCCGCCGCCGCc 5' | Ref: 5' tcctGGCTGGGGGCGGCGGCGa 3' |
| gga-miR-7475-5p | gi|619299521|gb|GAXD01021100.1| | #N/A | #N/A | 169 | -36.1 | 2 | 19 | 445 | 465 | 18 | Query: 3' ccUCCCG-CGCCGCCGCCGCc 5' | Ref: 5' cgATGGCGGTGGCGGCGGCGc 3' |
| gga-miR-7475-5p | gi|619299521|gb|GAXD01021100.1| | #N/A | #N/A | 157 | -33.01 | 2 | 19 | 469 | 489 | 18 | Query: 3' ccUCCCGCGCCG-CCGCCGCc 5' | Ref: 5' tgATGGCGGAGCGGGCGGCGg 3' |
| gga-miR-7475-5p | gi|619299520|gb|GAXD01021101.1| | #N/A | #N/A | 169 | -36.1 | 2 | 19 | 445 | 465 | 18 | Query: 3' ccUCCCG-CGCCGCCGCCGCc 5' | Ref: 5' cgATGGCGGTGGCGGCGGCGc 3' |
| gga-miR-7475-5p | gi|619299520|gb|GAXD01021101.1| | #N/A | #N/A | 157 | -33.01 | 2 | 19 | 469 | 489 | 18 | Query: 3' ccUCCCGCGCCG-CCGCCGCc 5' | Ref: 5' tgATGGCGGAGCGGGCGGCGg 3' |
| gga-miR-7475-5p | gi|619299084|gb|GAXD01021537.1| | #N/A | #N/A | 164 | -30.17 | 2 | 17 | 352 | 371 | 15 | Query: 3' ccucCCGCGCCGCCGCCGCc 5' | Ref: 5' gcgcGGCCCGTCGGCGGCGc 3' |
| gga-miR-7475-5p | gi|619298713|gb|GAXD01021908.1| | #N/A | #N/A | 158 | -30.21 | 2 | 19 | 16 | 35 | 17 | Query: 3' ccUCCCGCGCCGCCGCCGCc 5' | Ref: 5' gcGGGCCGAGCTGGCGGCGc 3' |
| gga-miR-7475-5p | gi|619298690|gb|GAXD01021931.1| | #N/A | #N/A | 150 | -30.73 | 2 | 19 | 343 | 362 | 17 | Query: 3' ccUCCCGCGCCGCCGCCGCc 5' | Ref: 5' agAGGGGAAAGGGGCGGCGa 3' |
| gga-miR-7475-5p | gi|619298202|gb|GAXD01022419.1| | #N/A | #N/A | 155 | -32.96 | 2 | 17 | 792 | 812 | 16 | Query: 3' ccucCCG-CGCCGCCGCCGCc 5' | Ref: 5' ccgcGGCGGCGAGGGCGGCGa 3' |
| gga-miR-7475-5p | gi|619298153|gb|GAXD01022468.1| | #N/A | #N/A | 158 | -35.98 | 2 | 17 | 790 | 811 | 17 | Query: 3' ccucCCG-CGCCG-CCGCCGCc 5' | Ref: 5' cgccGGCGGCGGTGGGCGGCGg 3' |
| gga-miR-7475-5p | gi|619297704|gb|GAXD01022917.1| | #N/A | #N/A | 154 | -41.69 | 2 | 19 | 278 | 294 | 17 | Query: 3' ccUCCCGCGCCGCCGCCGCc 5' | Ref: 5' tgAGGGC---GTGGCGGCGg 3' |
| gga-miR-7475-5p | gi|619297663|gb|GAXD01022958.1| | #N/A | #N/A | 166 | -32.24 | 2 | 19 | 867 | 886 | 17 | Query: 3' ccUCCCGCGCCGCCGCCGCc 5' | Ref: 5' gcGGGCCGCGTTGGCGGCGc 3' |
| gga-miR-7475-5p | gi|619297660|gb|GAXD01022961.1| | #N/A | #N/A | 166 | -32.24 | 2 | 19 | 867 | 886 | 17 | Query: 3' ccUCCCGCGCCGCCGCCGCc 5' | Ref: 5' gcGGGCCGCGTTGGCGGCGc 3' |
| gga-miR-7475-5p | gi|619297658|gb|GAXD01022963.1| | #N/A | #N/A | 166 | -32.24 | 2 | 19 | 867 | 886 | 17 | Query: 3' ccUCCCGCGCCGCCGCCGCc 5' | Ref: 5' gcGGGCCGCGTTGGCGGCGc 3' |
| gga-miR-7475-5p | gi|619297621|gb|GAXD01023000.1| | #N/A | #N/A | 148 | -34.1 | 2 | 15 | 978 | 1000 | 16 | Query: 3' ccucccGCGCC--G-CCGCCGCc 5' | Ref: 5' ggcgacCGCGGACCAGGCGGCGg 3' |
| gga-miR-7475-5p | gi|619296295|gb|GAXD01023843.1| | #N/A | #N/A | 158 | -34.25 | 2 | 19 | 2570 | 2589 | 17 | Query: 3' ccUCCCGCGCCGCCGCCGCc 5' | Ref: 5' ggAGGGGATCGTGGCGGCGa 3' |
| gga-miR-7475-5p | gi|619296153|gb|GAXD01023985.1| | #N/A | #N/A | 149 | -35.56 | 2 | 18 | 175 | 194 | 16 | Query: 3' ccuCCCGCGCCGCCGCCGCc 5' | Ref: 5' accGGAAGGTGTGGCGGCGg 3' |
| gga-miR-7475-5p | gi|619295954|gb|GAXD01024184.1| | #N/A | #N/A | 159 | -34.12 | 2 | 16 | 225 | 244 | 14 | Query: 3' ccuccCGCGCCGCCGCCGCc 5' | Ref: 5' gagctGCGCATCGGCGGCGg 3' |
| gga-miR-7475-5p | gi|619295778|gb|GAXD01024360.1| | #N/A | #N/A | 157 | -32.25 | 2 | 14 | 233 | 252 | 12 | Query: 3' ccucccgCGCCGCCGCCGCc 5' | Ref: 5' ggccgagGCGGAGGCGGCGg 3' |
| gga-miR-7475-5p | gi|619295385|gb|GAXD01024753.1| | #N/A | #N/A | 172 | -39.28 | 2 | 17 | 12 | 31 | 15 | Query: 3' ccucCCGCGCCGCCGCCGCc 5' | Ref: 5' gtgcGGAGCGGCGGCGGCGg 3' |
| gga-miR-7475-5p | gi|619295254|gb|GAXD01024884.1| | #N/A | #N/A | 155 | -34.92 | 2 | 19 | 470 | 487 | 17 | Query: 3' ccUCCCGCGCCGCCGCCGCc 5' | Ref: 5' atAGGGGGTG--GGCGGCGg 3' |
| gga-miR-7475-5p | gi|619294866|gb|GAXD01025272.1| | #N/A | #N/A | 157 | -31.41 | 2 | 15 | 190 | 210 | 14 | Query: 3' ccucccGCGCCG-CCGCCGCc 5' | Ref: 5' aggcctCGTGGCTGGCGGCGc 3' |
| gga-miR-7475-5p | gi|619294503|gb|GAXD01025635.1| | #N/A | #N/A | 169 | -35.86 | 2 | 18 | 342 | 361 | 16 | Query: 3' ccuCCCGCGCCGCCGCCGCc 5' | Ref: 5' aatGCGCGCTGCGGCGGCGg 3' |
| gga-miR-7475-5p | gi|619294473|gb|GAXD01025665.1| | #N/A | #N/A | 155 | -33.35 | 2 | 17 | 2229 | 2249 | 16 | Query: 3' ccucCCGCGCCG-CCGCCGCc 5' | Ref: 5' gtgcGGCGGGCCGGGCGGCGg 3' |
| gga-miR-7475-5p | gi|619294085|gb|GAXD01026053.1| | #N/A | #N/A | 176 | -45.35 | 2 | 18 | 178 | 198 | 17 | Query: 3' ccuCCCG-CGCCGCCGCCGCc 5' | Ref: 5' cttGGGCGGCGGCGGCGGCGg 3' |
| gga-miR-7475-5p | gi|619294075|gb|GAXD01026063.1| | #N/A | #N/A | 165 | -35.18 | 2 | 14 | 1 | 13 | 12 | Query: 3' ccucccgCGCCGCCGCCGCc 5' | Ref: 5' -------GCGGCGGCGGCGg 3' |
| gga-miR-7475-5p | gi|619293861|gb|GAXD01026277.1| | #N/A | #N/A | 163 | -33.6 | 2 | 16 | 17 | 36 | 14 | Query: 3' ccuccCGCGCCGCCGCCGCc 5' | Ref: 5' gacctGTGCGACGGCGGCGg 3' |
| gga-miR-7475-5p | gi|619293676|gb|GAXD01026462.1| | #N/A | #N/A | 162 | -30.73 | 2 | 15 | 219 | 238 | 13 | Query: 3' ccucccGCGCCGCCGCCGCc 5' | Ref: 5' gagactCTCGGCGGCGGCGt 3' |
| gga-miR-7475-5p | gi|619293608|gb|GAXD01026530.1| | #N/A | #N/A | 160 | -36.22 | 2 | 17 | 176 | 195 | 15 | Query: 3' ccucCCGCGCCGCCGCCGCc 5' | Ref: 5' ggttGGCGCACTGGCGGCGg 3' |
| gga-miR-7475-5p | gi|619293443|gb|GAXD01026695.1| | #N/A | #N/A | 159 | -30.25 | 2 | 17 | 58 | 78 | 16 | Query: 3' ccucCCGC-GCCGCCGCCGCc 5' | Ref: 5' tggcGGCGATGTCGGCGGCGt 3' |
| gga-miR-7475-5p | gi|619293405|gb|GAXD01026733.1| | #N/A | #N/A | 163 | -37.15 | 2 | 17 | 125 | 146 | 17 | Query: 3' ccucCCGCGCCG--CCGCCGCc 5' | Ref: 5' cgccGGCGTGGCGTGGCGGCGg 3' |
| gga-miR-7475-5p | gi|619293130|gb|GAXD01027008.1| | #N/A | #N/A | 159 | -33.03 | 2 | 19 | 73 | 90 | 17 | Query: 3' ccUCCCGCGCCGCCGCCGCc 5' | Ref: 5' taAGGGGGCG--GGCGGCGc 3' |
| gga-miR-7475-5p | gi|619293025|gb|GAXD01027113.1| | #N/A | #N/A | 165 | -36.59 | 2 | 14 | 1 | 14 | 12 | Query: 3' ccucccgCGCCGCCGCCGCc 5' | Ref: 5' ------gGCGGCGGCGGCGg 3' |
| gga-miR-7475-5p | gi|619293011|gb|GAXD01027127.1| | #N/A | #N/A | 158 | -32.99 | 2 | 19 | 3 | 22 | 17 | Query: 3' ccUCCCGCGCCGCCGCCGCc 5' | Ref: 5' gcGGGATGCTGGGGCGGCGg 3' |
| gga-miR-7475-5p | gi|619292997|gb|GAXD01027141.1| | #N/A | #N/A | 150 | -33.85 | 2 | 19 | 151 | 169 | 18 | Query: 3' ccUCC-CGCGCCGCCGCCGCc 5' | Ref: 5' agAGGAGAGCG--GGCGGCGg 3' |
| gga-miR-7475-5p | gi|619292802|gb|GAXD01027336.1| | #N/A | #N/A | 164 | -30.72 | 2 | 19 | 193 | 211 | 17 | Query: 3' ccUCCCGCGCCGCCGCCGCc 5' | Ref: 5' gaAGAGTGCGG-GGCGGCGc 3' |
| gga-miR-7475-5p | gi|619292749|gb|GAXD01027389.1| | #N/A | #N/A | 160 | -34.94 | 2 | 19 | 244 | 262 | 17 | Query: 3' ccUCCCGCGCCGCCGCCGCc 5' | Ref: 5' tcAGCG-GTGGTGGCGGCGg 3' |
| gga-miR-7475-5p | gi|619292749|gb|GAXD01027389.1| | #N/A | #N/A | 152 | -30.65 | 2 | 13 | 78 | 97 | 11 | Query: 3' ccucccgcGCCGCCGCCGCc 5' | Ref: 5' gtccgttcTGGTGGCGGCGg 3' |
| gga-miR-7475-5p | gi|619292705|gb|GAXD01027433.1| | #N/A | #N/A | 161 | -35.23 | 2 | 19 | 261 | 281 | 18 | Query: 3' ccUCCCGCGC-CGCCGCCGCc 5' | Ref: 5' gcGGGACGCGCGGGGCGGCGa 3' |
| gga-miR-7475-5p | gi|619292651|gb|GAXD01027487.1| | #N/A | #N/A | 148 | -36.76 | 2 | 19 | 183 | 201 | 17 | Query: 3' ccUCCCGCGCCGCCGCCGCc 5' | Ref: 5' ggAGGGAAT-TCGGCGGCGg 3' |
| gga-miR-7475-5p | gi|619292650|gb|GAXD01027488.1| | #N/A | #N/A | 162 | -32.38 | 2 | 19 | 255 | 274 | 17 | Query: 3' ccUCCCGCGCCGCCGCCGCc 5' | Ref: 5' ttATGGGGTCGCGGCGGCGc 3' |
| gga-miR-7475-5p | gi|619292650|gb|GAXD01027488.1| | #N/A | #N/A | 161 | -33.41 | 2 | 19 | 126 | 149 | 21 | Query: 3' ccUCCCGCG----CCGCCGCCGCc 5' | Ref: 5' atACGGCGCTATGGGCGGCGGCGt 3' |
| gga-miR-7475-5p | gi|619292612|gb|GAXD01027526.1| | #N/A | #N/A | 166 | -39.49 | 2 | 16 | 150 | 170 | 15 | Query: 3' ccuccCGCG-CCGCCGCCGCc 5' | Ref: 5' cggccGCGCTGGCGGCGGCGg 3' |
| gga-miR-7475-5p | gi|619292522|gb|GAXD01027616.1| | #N/A | #N/A | 166 | -39 | 2 | 17 | 161 | 179 | 15 | Query: 3' ccucCCGCGCCGCCGCCGCc 5' | Ref: 5' gaccGGC-CGGCGGCGGCGg 3' |
| gga-miR-7475-5p | gi|619292307|gb|GAXD01027831.1| | #N/A | #N/A | 152 | -32.84 | 2 | 14 | 172 | 193 | 14 | Query: 3' ccucccgCGCC--GCCGCCGCc 5' | Ref: 5' cggagtaGCGGCTCGGCGGCGt 3' |
| gga-miR-7475-5p | gi|619291552|gb|GAXD01028586.1| | #N/A | #N/A | 149 | -34.35 | 2 | 19 | 11 | 31 | 18 | Query: 3' ccUC-CCGCGCCGCCGCCGCc 5' | Ref: 5' ggAGTGACGGGTGGGCGGCGg 3' |
| gga-miR-7475-5p | gi|619290809|gb|GAXD01029329.1| | #N/A | #N/A | 164 | -41.86 | 2 | 19 | 198 | 220 | 20 | Query: 3' ccUCC--CG-CGCCGCCGCCGCc 5' | Ref: 5' cgAGGCAGCAGCGGTGGCGGCGg 3' |
| gga-miR-7475-5p | gi|619289378|gb|GAXD01030760.1| | #N/A | #N/A | 166 | -38.26 | 2 | 17 | 255 | 273 | 15 | Query: 3' ccucCCGCGCCGCCGCCGCc 5' | Ref: 5' gggcGGCGCGG-GGCGGCGt 3' |
| gga-miR-7475-5p | gi|619289002|gb|GAXD01031136.1| | #N/A | #N/A | 158 | -33.73 | 2 | 19 | 6 | 23 | 17 | Query: 3' ccUCCCGCGCCGCCGCCGCc 5' | Ref: 5' cgAGGG-GC-GTGGCGGCGa 3' |
| ppc-miR-8316-3p | gi|619304162|gb|GAXD01016460.1| | #N/A | #N/A | 163 | -30.84 | 2 | 18 | 145 | 166 | 19 | Query: 3' cgCU-GCUGG--ACCUGUGGUa 5' | Ref: 5' gcGACCGACCATTGGACACCAc 3' |
| ppc-miR-8316-3p | gi|619293558|gb|GAXD01026580.1| | #N/A | #N/A | 169 | -33.01 | 2 | 18 | 54 | 72 | 16 | Query: 3' cgCUGCUGGACCUGUGGUa 5' | Ref: 5' acGACGACGCGGACACCAt 3' |
| hsa-miR-6787-5p | gi|619320545|gb|GAXD01000204.1| | #N/A | #N/A | 162 | -33.05 | 2 | 21 | 1303 | 1326 | 21 | Query: 3' cgUC-GGUCGAGAU-GGGGGCGGu 5' | Ref: 5' ggAGCCCCGCTTCATCCCCCGCCa 3' |
| hsa-miR-6787-5p | gi|619320301|gb|GAXD01000448.1| | #N/A | #N/A | 162 | -36.19 | 2 | 21 | 159 | 185 | 24 | Query: 3' cgUCGGUC---GA--GAUGGGGGCGGu 5' | Ref: 5' ccAGCCAGAACCTCGCTAACCCCGCCa 3' |
| hsa-miR-6787-5p | gi|619319878|gb|GAXD01000871.1| | #N/A | #N/A | 171 | -38.88 | 2 | 21 | 492 | 515 | 21 | Query: 3' cgUCGGUCGAGAUG--GGGGCGGu 5' | Ref: 5' acAGCCGGCGCTGCCGCCCCGCCt 3' |
| hsa-miR-6787-5p | gi|619319665|gb|GAXD01001084.1| | #N/A | #N/A | 153 | -30.06 | 2 | 18 | 802 | 823 | 16 | Query: 3' cgucgGUCGAGAUGGGGGCGGu 5' | Ref: 5' gctaaCGTCGCCGCCCCCGCCa 3' |
| hsa-miR-6787-5p | gi|619319665|gb|GAXD01001084.1| | #N/A | #N/A | 153 | -30.06 | 2 | 18 | 979 | 1000 | 16 | Query: 3' cgucgGUCGAGAUGGGGGCGGu 5' | Ref: 5' gctaaCGTCGCCGCCCCCGCCa 3' |
| hsa-miR-6787-5p | gi|619319628|gb|GAXD01001121.1| | #N/A | #N/A | 160 | -32.6 | 2 | 18 | 507 | 529 | 17 | Query: 3' cgucgGUCGAGAUG-GGGGCGGu 5' | Ref: 5' ggccgCGGCTCGGCGCCCCGCCg 3' |
| hsa-miR-6787-5p | gi|619319004|gb|GAXD01001745.1| | #N/A | #N/A | 159 | -30.55 | 2 | 20 | 389 | 408 | 18 | Query: 3' cguCGGUCGAGAUGGGGGCGGu 5' | Ref: 5' accGCC-GC-CGACCCCCGCCt 3' |
| hsa-miR-6787-5p | gi|619319003|gb|GAXD01001746.1| | #N/A | #N/A | 159 | -30.55 | 2 | 20 | 336 | 355 | 18 | Query: 3' cguCGGUCGAGAUGGGGGCGGu 5' | Ref: 5' accGCC-GC-CGACCCCCGCCt 3' |
| hsa-miR-6787-5p | gi|619318995|gb|GAXD01001754.1| | #N/A | #N/A | 163 | -33.33 | 2 | 20 | 42 | 63 | 18 | Query: 3' cguCGGUCGAGAUGGGGGCGGu 5' | Ref: 5' cctGCTGGCTGCCCCCCCGCCa 3' |
| hsa-miR-6787-5p | gi|619318728|gb|GAXD01002021.1| | #N/A | #N/A | 163 | -33.88 | 2 | 21 | 66 | 89 | 21 | Query: 3' cgUCGGUCGAGA--UGGGGGCGGu 5' | Ref: 5' ccACCAAGCTCTCCTCCCCCGCCt 3' |
| hsa-miR-6787-5p | gi|619317941|gb|GAXD01002808.1| | #N/A | #N/A | 151 | -32.42 | 2 | 21 | 981 | 1000 | 20 | Query: 3' cgUC-GGUCGAGAUGGGGGCGGu 5' | Ref: 5' gcAGACCGGC---ATCCCCGCCt 3' |
| hsa-miR-6787-5p | gi|619317057|gb|GAXD01003692.1| | #N/A | #N/A | 150 | -30.54 | 2 | 11 | 161 | 182 | 9 | Query: 3' cgucggucgagaUGGGGGCGGu 5' | Ref: 5' cagttcacaggcACCCCCGCCg 3' |
| hsa-miR-6787-5p | gi|619316828|gb|GAXD01003921.1| | #N/A | #N/A | 159 | -30.37 | 2 | 21 | 26 | 48 | 20 | Query: 3' cgUCGGUCGAGA-UGGGGGCGGu 5' | Ref: 5' cgAGCCCCCTTTCGACCCCGCCa 3' |
| hsa-miR-6787-5p | gi|619315720|gb|GAXD01005029.1| | #N/A | #N/A | 154 | -32.56 | 2 | 21 | 56 | 76 | 19 | Query: 3' cgUCGGUCGAGAUGGGGGCGGu 5' | Ref: 5' ccAGCCGGGCCCG-CCCCGCCa 3' |
| hsa-miR-6787-5p | gi|619315664|gb|GAXD01005085.1| | #N/A | #N/A | 156 | -30.91 | 2 | 21 | 87 | 105 | 19 | Query: 3' cgUCGGUCGAGAUGGGGGCGGu 5' | Ref: 5' caAACCAGC---ATCCCCGCCa 3' |
| hsa-miR-6787-5p | gi|619315586|gb|GAXD01005163.1| | #N/A | #N/A | 158 | -30.91 | 2 | 20 | 806 | 829 | 20 | Query: 3' cguCGGUCGA--GAUGGGGGCGGu 5' | Ref: 5' cccGTCAGTTTCCGGTCCCCGCCg 3' |
| hsa-miR-6787-5p | gi|619315566|gb|GAXD01005183.1| | #N/A | #N/A | 175 | -39.8 | 2 | 21 | 90 | 112 | 20 | Query: 3' cgUCGGU-CGAGAUGGGGGCGGu 5' | Ref: 5' tcAGCCGTGCTCTCTCCCCGCCt 3' |
| hsa-miR-6787-5p | gi|619315361|gb|GAXD01005388.1| | #N/A | #N/A | 161 | -35.38 | 2 | 19 | 207 | 230 | 19 | Query: 3' cgucGGUCGA--GAUGGGGGCGGu 5' | Ref: 5' tcgcCCAGCTAAACACCCCCGCCt 3' |
| hsa-miR-6787-5p | gi|619314122|gb|GAXD01006627.1| | #N/A | #N/A | 160 | -32.24 | 2 | 21 | 36 | 55 | 19 | Query: 3' cgUCGGUCGAGAUGGGGGCGGu 5' | Ref: 5' acACTCAGC-C-ACCCCCGCCc 3' |
| hsa-miR-6787-5p | gi|619313337|gb|GAXD01007412.1| | #N/A | #N/A | 150 | -31.88 | 2 | 21 | 217 | 242 | 23 | Query: 3' cgUCGGUCG-A---GAUGGGGGCGGu 5' | Ref: 5' taAGCAGGCGTAAGCTGACCCCGCCg 3' |
| hsa-miR-6787-5p | gi|619312378|gb|GAXD01008371.1| | #N/A | #N/A | 151 | -32.42 | 2 | 21 | 1067 | 1086 | 20 | Query: 3' cgUC-GGUCGAGAUGGGGGCGGu 5' | Ref: 5' gcAGACCGGC---ATCCCCGCCt 3' |
| hsa-miR-6787-5p | gi|619312377|gb|GAXD01008372.1| | #N/A | #N/A | 151 | -32.42 | 2 | 21 | 1073 | 1092 | 20 | Query: 3' cgUC-GGUCGAGAUGGGGGCGGu 5' | Ref: 5' gcAGACCGGC---ATCCCCGCCt 3' |
| hsa-miR-6787-5p | gi|619312121|gb|GAXD01008628.1| | #N/A | #N/A | 148 | -32.64 | 2 | 21 | 318 | 337 | 19 | Query: 3' cgUCGGUCGAGAUGGGGGCGGu 5' | Ref: 5' gcAGCC-GC-AGAACCCCGCCa 3' |
| hsa-miR-6787-5p | gi|619310876|gb|GAXD01009873.1| | #N/A | #N/A | 170 | -33.3 | 2 | 20 | 255 | 277 | 19 | Query: 3' cguCGGUCGAGAUG-GGGGCGGu 5' | Ref: 5' agcGCCCGCACTACTCCCCGCCt 3' |
| hsa-miR-6787-5p | gi|619307725|gb|GAXD01012897.1| | #N/A | #N/A | 156 | -31.3 | 2 | 13 | 252 | 273 | 11 | Query: 3' cgucggucgaGAUGGGGGCGGu 5' | Ref: 5' cgcactcaagCTGCCCCCGCCc 3' |
| hsa-miR-6787-5p | gi|619306883|gb|GAXD01013739.1| | #N/A | #N/A | 156 | -36.14 | 2 | 21 | 243 | 264 | 19 | Query: 3' cgUCGGUCGAGAUGGGGGCGGu 5' | Ref: 5' ccGGCCGGCCGGGGCCCCGCCg 3' |
| hsa-miR-6787-5p | gi|619306458|gb|GAXD01014164.1| | #N/A | #N/A | 161 | -32.49 | 2 | 15 | 42 | 64 | 14 | Query: 3' cgucggucGAGA-UGGGGGCGGu 5' | Ref: 5' cccctcctCTCTCACCCCCGCCa 3' |
| hsa-miR-6787-5p | gi|619305923|gb|GAXD01014699.1| | #N/A | #N/A | 163 | -37.66 | 2 | 21 | 6 | 28 | 20 | Query: 3' cgUCGGUCGAGAUG-GGGGCGGu 5' | Ref: 5' gcAGCCGGCGCCCCGCCCCGCCa 3' |
| hsa-miR-6787-5p | gi|619305760|gb|GAXD01014862.1| | #N/A | #N/A | 167 | -30.97 | 2 | 21 | 159 | 181 | 20 | Query: 3' cgUCGGUCGAGA-UGGGGGCGGu 5' | Ref: 5' agAGCAATCTCTCTCCCCCGCCc 3' |
| hsa-miR-6787-5p | gi|619305578|gb|GAXD01015044.1| | #N/A | #N/A | 158 | -33.16 | 2 | 20 | 235 | 257 | 19 | Query: 3' cguCGGUC-GAGAUGGGGGCGGu 5' | Ref: 5' accGCAAGAAGCTGCCCCCGCCg 3' |
| hsa-miR-6787-5p | gi|619305332|gb|GAXD01015290.1| | #N/A | #N/A | 156 | -30.63 | 2 | 20 | 169 | 193 | 21 | Query: 3' cguCGGU-CGAG-A-UGGGGGCGGu 5' | Ref: 5' actGCCATGCACATCGCCCCCGCCc 3' |
| hsa-miR-6787-5p | gi|619305059|gb|GAXD01015563.1| | #N/A | #N/A | 158 | -32.11 | 2 | 15 | 198 | 219 | 13 | Query: 3' cgucggucGAGAUGGGGGCGGu 5' | Ref: 5' gccctcctCTTTTCCCCCGCCg 3' |
| hsa-miR-6787-5p | gi|619304937|gb|GAXD01015685.1| | #N/A | #N/A | 144 | -32.15 | 2 | 21 | 244 | 267 | 22 | Query: 3' cgUC-GGUCGAG--AUGGGGGCGGu 5' | Ref: 5' acAGACCA-CTCGGAGGCCCCGCCg 3' |
| hsa-miR-6787-5p | gi|619304794|gb|GAXD01015828.1| | #N/A | #N/A | 152 | -31.19 | 2 | 14 | 44 | 66 | 13 | Query: 3' cgucggucgAGAUG-GGGGCGGu 5' | Ref: 5' cagtttttaTCTGCTCCCCGCCa 3' |
| hsa-miR-6787-5p | gi|619304623|gb|GAXD01015999.1| | #N/A | #N/A | 152 | -33.57 | 2 | 21 | 82 | 102 | 20 | Query: 3' cgUCG-GUCGAGAUGGGGGCGGu 5' | Ref: 5' gcGGCGCGGCCCT--CCCCGCCg 3' |
| hsa-miR-6787-5p | gi|619304332|gb|GAXD01016290.1| | #N/A | #N/A | 144 | -30.3 | 2 | 21 | 191 | 218 | 25 | Query: 3' cgUCG-GUCGA-GA---UG-GGGGCGGu 5' | Ref: 5' gcAGCGCTGCTCCTCGCGCGCCCCGCCc 3' |
| hsa-miR-6787-5p | gi|619304230|gb|GAXD01016392.1| | #N/A | #N/A | 160 | -37.02 | 2 | 21 | 84 | 105 | 19 | Query: 3' cgUCGGUCGAGAUGGGGGCGGu 5' | Ref: 5' gcAGCCTACTCGCGCCCCGCCg 3' |
| hsa-miR-6787-5p | gi|619303934|gb|GAXD01016688.1| | #N/A | #N/A | 153 | -30.45 | 2 | 19 | 106 | 131 | 21 | Query: 3' cgucGGUCGAGAUG----GGGGCGGu 5' | Ref: 5' cattCGAGTTCTGCCGCACCCCGCCg 3' |
| hsa-miR-6787-5p | gi|619302098|gb|GAXD01018524.1| | #N/A | #N/A | 154 | -31.48 | 2 | 19 | 242 | 263 | 17 | Query: 3' cgucGGUCGAGAUGGGGGCGGu 5' | Ref: 5' gcgaCGACCGCCGCCCCCGCCa 3' |
| hsa-miR-6787-5p | gi|619300560|gb|GAXD01020062.1| | #N/A | #N/A | 151 | -30.83 | 2 | 20 | 275 | 294 | 18 | Query: 3' cguCGGUCGAGAUGGGGGCGGu 5' | Ref: 5' cgtGCCCG-GCTA-CCCCGCCa 3' |
| hsa-miR-6787-5p | gi|619299253|gb|GAXD01021368.1| | #N/A | #N/A | 163 | -31.34 | 2 | 20 | 44 | 65 | 18 | Query: 3' cguCGGUCGAGAUGGGGGCGGu 5' | Ref: 5' cgcGCGGCCTCGGCCCCCGCCc 3' |
| hsa-miR-6787-5p | gi|619298877|gb|GAXD01021744.1| | #N/A | #N/A | 159 | -33.84 | 2 | 19 | 299 | 320 | 18 | Query: 3' cgucGGUCGAGAUG-GGGGCGGu 5' | Ref: 5' acgtCCAGC-CTCCGCCCCGCCg 3' |
| hsa-miR-6787-5p | gi|619298446|gb|GAXD01022175.1| | #N/A | #N/A | 166 | -30.78 | 2 | 20 | 79 | 101 | 19 | Query: 3' cguCGGUCGA-GAUGGGGGCGGu 5' | Ref: 5' cccGCCTTCTCCTATCCCCGCCc 3' |
| hsa-miR-6787-5p | gi|619298383|gb|GAXD01022238.1| | #N/A | #N/A | 150 | -30.85 | 2 | 21 | 158 | 182 | 22 | Query: 3' cgUCGGU--CGAGAUG-GGGGCGGu 5' | Ref: 5' tcAGTCGTCGCGTTGTGCCCCGCCg 3' |
| hsa-miR-6787-5p | gi|619298091|gb|GAXD01022530.1| | #N/A | #N/A | 154 | -35.14 | 2 | 21 | 10 | 30 | 19 | Query: 3' cgUCGGUCGAGAUGGGGGCGGu 5' | Ref: 5' tcGGCCGAATC-GTCCCCGCCg 3' |
| hsa-miR-6787-5p | gi|619297940|gb|GAXD01022681.1| | #N/A | #N/A | 162 | -31.28 | 2 | 21 | 18 | 38 | 19 | Query: 3' cgUCGGUCGAGAUGGGGGCGGu 5' | Ref: 5' tcAATTAGCTTTG-CCCCGCCt 3' |
| hsa-miR-6787-5p | gi|619297925|gb|GAXD01022696.1| | #N/A | #N/A | 161 | -31.48 | 2 | 19 | 199 | 221 | 18 | Query: 3' cgucGGUCGAG-AUGGGGGCGGu 5' | Ref: 5' gtttCCGGCGCACACCCCCGCCt 3' |
| hsa-miR-6787-5p | gi|619297255|gb|GAXD01023366.1| | #N/A | #N/A | 150 | -31.46 | 2 | 21 | 1 | 19 | 19 | Query: 3' cgUCGGUCGAGAUGGGGGCGGu 5' | Ref: 5' --GGCGTGC-CAGTCCCCGCCg 3' |
| hsa-miR-6787-5p | gi|619297078|gb|GAXD01023543.1| | #N/A | #N/A | 158 | -31.47 | 2 | 20 | 502 | 524 | 19 | Query: 3' cguCGGUCGAGA-UGGGGGCGGu 5' | Ref: 5' cgtGGCGGCGCTGGTCCCCGCCa 3' |
| hsa-miR-6787-5p | gi|619295973|gb|GAXD01024165.1| | #N/A | #N/A | 155 | -30.11 | 2 | 21 | 264 | 286 | 20 | Query: 3' cgUCGGUCGAGAUG-GGGGCGGu 5' | Ref: 5' cgGGTCGGATTCGCGCCCCGCCg 3' |
| hsa-miR-6787-5p | gi|619295927|gb|GAXD01024211.1| | #N/A | #N/A | 158 | -31.33 | 2 | 20 | 455 | 479 | 21 | Query: 3' cguCGGUCGAGAUG---GGGGCGGu 5' | Ref: 5' aacGCCAGCGCCGCCTACCCCGCCt 3' |
| hsa-miR-6787-5p | gi|619295662|gb|GAXD01024476.1| | #N/A | #N/A | 166 | -31.42 | 2 | 19 | 198 | 219 | 17 | Query: 3' cgucGGUCGAGAUGGGGGCGGu 5' | Ref: 5' tgcaCCTGCCCTCCCCCCGCCt 3' |
| hsa-miR-6787-5p | gi|619295403|gb|GAXD01024735.1| | #N/A | #N/A | 171 | -38.88 | 2 | 21 | 171 | 194 | 21 | Query: 3' cgUCGGUCGAGAUG--GGGGCGGu 5' | Ref: 5' acAGCCGGCGCTGCCGCCCCGCCt 3' |
| hsa-miR-6787-5p | gi|619295402|gb|GAXD01024736.1| | #N/A | #N/A | 171 | -38.88 | 2 | 21 | 547 | 570 | 21 | Query: 3' cgUCGGUCGAGAUG--GGGGCGGu 5' | Ref: 5' acAGCCGGCGCTGCCGCCCCGCCt 3' |
| hsa-miR-6787-5p | gi|619295296|gb|GAXD01024842.1| | #N/A | #N/A | 158 | -31.49 | 2 | 21 | 27 | 47 | 19 | Query: 3' cgUCGGUCGAGAUGGGGGCGGu 5' | Ref: 5' tgGGTCAGCGC-GGCCCCGCCg 3' |
| hsa-miR-6787-5p | gi|619294958|gb|GAXD01025180.1| | #N/A | #N/A | 155 | -31.35 | 2 | 20 | 53 | 72 | 18 | Query: 3' cguCGGUCGAGAUGGGGGCGGu 5' | Ref: 5' cgcGCC-GCGC-GCCCCCGCCt 3' |
| hsa-miR-6787-5p | gi|619294372|gb|GAXD01025766.1| | #N/A | #N/A | 153 | -31.17 | 2 | 19 | 19 | 42 | 19 | Query: 3' cgucGGUCGAGAUG--GGGGCGGu 5' | Ref: 5' cggtCCGGTTCGCCCACCCCGCCc 3' |
| hsa-miR-6787-5p | gi|619294079|gb|GAXD01026059.1| | #N/A | #N/A | 163 | -32.38 | 2 | 20 | 206 | 227 | 18 | Query: 3' cguCGGUCGAGAUGGGGGCGGu 5' | Ref: 5' gccGTCGGGCCCACCCCCGCCa 3' |
| hsa-miR-6787-5p | gi|619293702|gb|GAXD01026436.1| | #N/A | #N/A | 161 | -32.78 | 2 | 19 | 240 | 262 | 18 | Query: 3' cgucGGUCGAGAUG-GGGGCGGu 5' | Ref: 5' aacaCTAGCTCGCCTCCCCGCCt 3' |
| hsa-miR-6787-5p | gi|619293368|gb|GAXD01026770.1| | #N/A | #N/A | 163 | -31 | 2 | 21 | 175 | 198 | 21 | Query: 3' cgUCGGUCG--AGAUGGGGGCGGu 5' | Ref: 5' caAGCAGGCCTTCCGCCCCCGCCc 3' |
| hsa-miR-6787-5p | gi|619293216|gb|GAXD01026922.1| | #N/A | #N/A | 155 | -33.71 | 2 | 21 | 183 | 203 | 20 | Query: 3' cgUCGGUCGAGAUG-GGGGCGGu 5' | Ref: 5' agGGCCGGC-C-ACGCCCCGCCa 3' |
| hsa-miR-6787-5p | gi|619292379|gb|GAXD01027759.1| | #N/A | #N/A | 168 | -33.63 | 2 | 21 | 119 | 140 | 19 | Query: 3' cgUCGGUCGAGAUGGGGGCGGu 5' | Ref: 5' gtGGCCTGGACTGCCCCCGCCc 3' |
| hsa-miR-6787-5p | gi|619292109|gb|GAXD01028029.1| | #N/A | #N/A | 163 | -32.7 | 2 | 21 | 173 | 195 | 20 | Query: 3' cgUCGGUCGAGAUG-GGGGCGGu 5' | Ref: 5' tgGGCCAGTATGACTCCCCGCCa 3' |
| hsa-miR-6787-5p | gi|619291430|gb|GAXD01028708.1| | #N/A | #N/A | 156 | -30.13 | 2 | 21 | 116 | 137 | 19 | Query: 3' cgUCGGUCGAGAUGGGGGCGGu 5' | Ref: 5' gcAGGCCCCTGCGCCCCCGCCc 3' |
| hsa-miR-6787-5p | gi|619291404|gb|GAXD01028734.1| | #N/A | #N/A | 163 | -31.58 | 2 | 21 | 144 | 166 | 20 | Query: 3' cgUC-GGUCGAGAUGGGGGCGGu 5' | Ref: 5' agAGCCCCCCGCTATCCCCGCCc 3' |
| hsa-miR-6787-5p | gi|619291258|gb|GAXD01028880.1| | #N/A | #N/A | 164 | -31.59 | 2 | 20 | 18 | 37 | 18 | Query: 3' cguCGGUCGAGAUGGGGGCGGu 5' | Ref: 5' tctGCCTGCTCT--CCCCGCCt 3' |
| hsa-miR-6787-5p | gi|619289905|gb|GAXD01030233.1| | #N/A | #N/A | 157 | -31.15 | 2 | 17 | 143 | 164 | 16 | Query: 3' cgucggUC-GAGAUGGGGGCGGu 5' | Ref: 5' agtgaaAGCCTCT-CCCCCGCCg 3' |
| hsa-miR-6787-5p | gi|619289466|gb|GAXD01030672.1| | #N/A | #N/A | 161 | -31.33 | 2 | 21 | 248 | 272 | 22 | Query: 3' cgUCGGU-CGA-GAU-GGGGGCGGu 5' | Ref: 5' aaGGCTAGGCTACTGTCCCCCGCCc 3' |
| gga-miR-1723 | gi|619318835|gb|GAXD01001914.1| | #N/A | #N/A | 155 | -30.85 | 2 | 17 | 43 | 66 | 16 | Query: 3' acuccgaCGUGUAAG-GCGAGGGu 5' | Ref: 5' gcccgggGCACACGCACGCTCCCg 3' |
| gga-miR-1723 | gi|619317880|gb|GAXD01002869.1| | #N/A | #N/A | 172 | -33.84 | 2 | 21 | 273 | 295 | 19 | Query: 3' acuCCGACGUGUAAGGCGAGGGu 5' | Ref: 5' cgtGGTTGCGTGTGTCGCTCCCg 3' |
| gga-miR-1723 | gi|619315556|gb|GAXD01005193.1| | #N/A | #N/A | 168 | -37.37 | 2 | 22 | 209 | 232 | 21 | Query: 3' acUCCG-ACGUGUAAGGCGAGGGu 5' | Ref: 5' ggAGGCTTGCTGGGTCCGCTCCCa 3' |
| gga-miR-1723 | gi|619311542|gb|GAXD01009207.1| | #N/A | #N/A | 158 | -30.28 | 2 | 20 | 160 | 183 | 19 | Query: 3' acucCGACG-UGUAAGGCGAGGGu 5' | Ref: 5' cctcGCTGCTTTAGGCCGCTCCCa 3' |
| gga-miR-1723 | gi|619299597|gb|GAXD01021024.1| | #N/A | #N/A | 163 | -30.31 | 2 | 21 | 188 | 211 | 20 | Query: 3' acuCCGACG-UGUAAGGCGAGGGu 5' | Ref: 5' gtcGACTGCTGCCGTCCGCTCCCt 3' |
| gga-miR-1723 | gi|619299078|gb|GAXD01021543.1| | #N/A | #N/A | 161 | -30.3 | 2 | 20 | 1 | 21 | 18 | Query: 3' acucCGACGUGUAAGGCGAGGGu 5' | Ref: 5' -tgaGCCG-ACGCTCCGCTCCCg 3' |
| gga-miR-1723 | gi|619298687|gb|GAXD01021934.1| | #N/A | #N/A | 176 | -30.11 | 2 | 21 | 297 | 319 | 19 | Query: 3' acuCCGACGUGUAAGGCGAGGGu 5' | Ref: 5' ggtGGCTACAATTTCCGCTCCCt 3' |
| gga-miR-1723 | gi|619297443|gb|GAXD01023178.1| | #N/A | #N/A | 151 | -31.04 | 2 | 22 | 427 | 454 | 25 | Query: 3' acUCCGACGUGU---A--AGGCGAGGGu 5' | Ref: 5' atAGGGAGCGCGAGCTCCTCCGCTCCCa 3' |
| gga-miR-1723 | gi|619297437|gb|GAXD01023184.1| | #N/A | #N/A | 151 | -31.04 | 2 | 22 | 1109 | 1136 | 25 | Query: 3' acUCCGACGUGU---A--AGGCGAGGGu 5' | Ref: 5' atAGGGAGCGCGAGCTCCTCCGCTCCCa 3' |
| gga-miR-1723 | gi|619294549|gb|GAXD01025589.1| | #N/A | #N/A | 165 | -34.34 | 2 | 22 | 49 | 71 | 20 | Query: 3' acUCCGACGUGUAAGGCGAGGGu 5' | Ref: 5' ggAGGCCGCCGCTCCCGCTCCCg 3' |
| gga-miR-1723 | gi|619288874|gb|GAXD01031264.1| | #N/A | #N/A | 169 | -31.25 | 2 | 22 | 102 | 121 | 20 | Query: 3' acUCCGACGUGUAAGGCGAGGGu 5' | Ref: 5' tcAGGCT---CATTTCGCTCCCa 3' |
| bmo-miR-3344 | gi|619298503|gb|GAXD01022118.1| | #N/A | #N/A | 174 | -31.43 | 2 | 21 | 3789 | 3812 | 19 | Query: 3' gagcgACCGACUCAGGAAGAACGUu 5' | Ref: 5' agttgTGGCTGAGT-TGTCTTGCAa 3' |
| bmo-miR-3344 | gi|619298500|gb|GAXD01022121.1| | #N/A | #N/A | 174 | -31.43 | 2 | 21 | 3857 | 3880 | 19 | Query: 3' gagcgACCGACUCAGGAAGAACGUu 5' | Ref: 5' agttgTGGCTGAGT-TGTCTTGCAa 3' |
| bmo-miR-3344 | gi|619294008|gb|GAXD01026130.1| | #N/A | #N/A | 171 | -30.39 | 2 | 22 | 43 | 70 | 23 | Query: 3' gagcGACC--GACUCAGG-AAGAACGUu 5' | Ref: 5' acttCTGGGCTTTAGTCCTTTCTTGCAa 3' |
| hsa-miR-4459 | gi|619320428|gb|GAXD01000321.1| | #N/A | #N/A | 151 | -30.31 | 2 | 21 | 11 | 33 | 20 | Query: 3' gaGGU-GGAGGAGGCGGAGGACc 5' | Ref: 5' ctCCAGCAGTGTCTTCCTCCTGg 3' |
| hsa-miR-4459 | gi|619320070|gb|GAXD01000679.1| | #N/A | #N/A | 172 | -30.55 | 2 | 21 | 197 | 218 | 19 | Query: 3' gaGGUGGAGGAGGCGGAGGACc 5' | Ref: 5' atCCACCATATCCCCCTCCTGa 3' |
| hsa-miR-4459 | gi|619320040|gb|GAXD01000709.1| | #N/A | #N/A | 173 | -42.64 | 2 | 21 | 145 | 166 | 20 | Query: 3' gaGGUGGAGGAGG-CGGAGGACc 5' | Ref: 5' atCCACC-CCTCTGGCCTCCTGg 3' |
| hsa-miR-4459 | gi|619319059|gb|GAXD01001690.1| | #N/A | #N/A | 166 | -33.65 | 2 | 20 | 61 | 83 | 19 | Query: 3' gagGUGGAGGAGGC-GGAGGACc 5' | Ref: 5' ccgCATCTTCGTCGTCCTCCTGg 3' |
| hsa-miR-4459 | gi|619319058|gb|GAXD01001691.1| | #N/A | #N/A | 166 | -33.65 | 2 | 20 | 10 | 32 | 19 | Query: 3' gagGUGGAGGAGGC-GGAGGACc 5' | Ref: 5' ccgCATCTTCGTCGTCCTCCTGg 3' |
| hsa-miR-4459 | gi|619319055|gb|GAXD01001694.1| | #N/A | #N/A | 160 | -31.27 | 2 | 21 | 226 | 247 | 19 | Query: 3' gaGGUGGAGGAGGCGGAGGACc 5' | Ref: 5' ctCCAGTGTGCCCGCCTCCTGc 3' |
| hsa-miR-4459 | gi|619318685|gb|GAXD01002064.1| | #N/A | #N/A | 160 | -30.08 | 2 | 21 | 406 | 427 | 19 | Query: 3' gaGGUGGAGGAGGCGGAGGACc 5' | Ref: 5' ccCCGTTACCATGGCCTCCTGg 3' |
| hsa-miR-4459 | gi|619318505|gb|GAXD01002244.1| | #N/A | #N/A | 166 | -33.13 | 2 | 21 | 223 | 242 | 19 | Query: 3' gaGGUGGAGGAGGCGGAGGACc 5' | Ref: 5' tgCCGGC-CTTCTGCCTCCTG- 3' |
| hsa-miR-4459 | gi|619318041|gb|GAXD01002708.1| | #N/A | #N/A | 162 | -32.13 | 2 | 20 | 5 | 27 | 19 | Query: 3' gagGUGGAGGA-GGCGGAGGACc 5' | Ref: 5' aggCTCCTCGTGCCCCCTCCTGg 3' |
| hsa-miR-4459 | gi|619317941|gb|GAXD01002808.1| | #N/A | #N/A | 154 | -30.05 | 2 | 20 | 300 | 322 | 19 | Query: 3' gagGUGGAGGAGGC-GGAGGACc 5' | Ref: 5' gcgCGGCATCGCCGACCTCCTGg 3' |
| hsa-miR-4459 | gi|619317681|gb|GAXD01003068.1| | #N/A | #N/A | 158 | -34.17 | 2 | 21 | 261 | 281 | 19 | Query: 3' gaGGUGGAGGAGGCGGAGGACc 5' | Ref: 5' ctCTGTCTGC-CCCCCTCCTGg 3' |
| hsa-miR-4459 | gi|619317545|gb|GAXD01003204.1| | #N/A | #N/A | 171 | -33.45 | 2 | 21 | 137 | 161 | 22 | Query: 3' gaGGUGGAG---GAGGCGGAGGACc 5' | Ref: 5' caTCACTTCAATCTCTGCCTCCTGt 3' |
| hsa-miR-4459 | gi|619317516|gb|GAXD01003233.1| | #N/A | #N/A | 175 | -34 | 2 | 21 | 95 | 117 | 20 | Query: 3' gaGGU-GGAGGAGGCGGAGGACc 5' | Ref: 5' acCCAGTCACCTCTGCCTCCTGc 3' |
| hsa-miR-4459 | gi|619315701|gb|GAXD01005048.1| | #N/A | #N/A | 174 | -31.37 | 2 | 19 | 295 | 316 | 17 | Query: 3' gaggUGGAGGAGGCGGAGGACc 5' | Ref: 5' gagaAGCTCCACCGCCTCCTGt 3' |
| hsa-miR-4459 | gi|619315530|gb|GAXD01005219.1| | #N/A | #N/A | 162 | -30.65 | 2 | 19 | 812 | 833 | 17 | Query: 3' gaggUGGAGGAGGCGGAGGACc 5' | Ref: 5' agagAGCGCCTCTTCCTCCTGg 3' |
| hsa-miR-4459 | gi|619314627|gb|GAXD01006122.1| | #N/A | #N/A | 167 | -36.7 | 2 | 21 | 222 | 247 | 23 | Query: 3' gaGGUGGAGGAGGC----GGAGGACc 5' | Ref: 5' gcCCGCCTGCTCCGCGCCCCTCCTGt 3' |
| hsa-miR-4459 | gi|619314550|gb|GAXD01006199.1| | #N/A | #N/A | 161 | -30.12 | 2 | 20 | 347 | 367 | 18 | Query: 3' gagGUGGAGGAGGCGGAGGACc 5' | Ref: 5' cagTACCT-TTTGGCCTCCTGg 3' |
| hsa-miR-4459 | gi|619314462|gb|GAXD01006287.1| | #N/A | #N/A | 171 | -35.58 | 2 | 21 | 611 | 633 | 20 | Query: 3' gaGGUGG-AGGAGGCGGAGGACc 5' | Ref: 5' cgCAGCCGTCCTCCCCCTCCTGg 3' |
| hsa-miR-4459 | gi|619314372|gb|GAXD01006377.1| | #N/A | #N/A | 166 | -31.24 | 2 | 20 | 251 | 273 | 19 | Query: 3' gagGUGGAGGA-GGCGGAGGACc 5' | Ref: 5' aggTACTTCCTGATGCCTCCTGt 3' |
| hsa-miR-4459 | gi|619312378|gb|GAXD01008371.1| | #N/A | #N/A | 154 | -30.05 | 2 | 20 | 386 | 408 | 19 | Query: 3' gagGUGGAGGAGGC-GGAGGACc 5' | Ref: 5' gcgCGGCATCGCCGACCTCCTGg 3' |
| hsa-miR-4459 | gi|619312377|gb|GAXD01008372.1| | #N/A | #N/A | 154 | -30.05 | 2 | 20 | 300 | 322 | 19 | Query: 3' gagGUGGAGGAGGC-GGAGGACc 5' | Ref: 5' gcgCGGCATCGCCGACCTCCTGg 3' |
| hsa-miR-4459 | gi|619312376|gb|GAXD01008373.1| | #N/A | #N/A | 154 | -30.05 | 2 | 20 | 300 | 322 | 19 | Query: 3' gagGUGGAGGAGGC-GGAGGACc 5' | Ref: 5' gcgCGGCATCGCCGACCTCCTGg 3' |
| hsa-miR-4459 | gi|619311305|gb|GAXD01009444.1| | #N/A | #N/A | 167 | -40.34 | 2 | 21 | 17 | 41 | 22 | Query: 3' gaGGUGGAGG---AGGCGGAGGACc 5' | Ref: 5' gtCCACCTCCCAGCCCTCCTCCTGg 3' |
| hsa-miR-4459 | gi|619311050|gb|GAXD01009699.1| | #N/A | #N/A | 153 | -32.15 | 2 | 21 | 362 | 383 | 20 | Query: 3' gaGGUGG-AGGAGGCGGAGGACc 5' | Ref: 5' taCCACCAGACTTT-CCTCCTGg 3' |
| hsa-miR-4459 | gi|619310192|gb|GAXD01010458.1| | #N/A | #N/A | 164 | -30.57 | 2 | 21 | 433 | 454 | 19 | Query: 3' gaGGUGGAGGAGGCGGAGGACc 5' | Ref: 5' ggCGAGCTCCTTGTCCTCCTGg 3' |
| hsa-miR-4459 | gi|619310113|gb|GAXD01010533.1| | #N/A | #N/A | 174 | -35.58 | 2 | 21 | 32 | 52 | 19 | Query: 3' gaGGUGGAGGAGGCGGAGGACc 5' | Ref: 5' gcCCGCCGCCT-CGCCTCCTGt 3' |
| hsa-miR-4459 | gi|619309444|gb|GAXD01011178.1| | #N/A | #N/A | 180 | -34.6 | 2 | 21 | 73 | 94 | 19 | Query: 3' gaGGUGGAGGAGGCGGAGGACc 5' | Ref: 5' caCCACTTCCCCCACCTCCTGc 3' |
| hsa-miR-4459 | gi|619309288|gb|GAXD01011334.1| | #N/A | #N/A | 169 | -32.88 | 2 | 18 | 256 | 277 | 16 | Query: 3' gagguGGAGGAGGCGGAGGACc 5' | Ref: 5' aaatcCCCCCTCCCCCTCCTGa 3' |
| hsa-miR-4459 | gi|619308935|gb|GAXD01011687.1| | #N/A | #N/A | 164 | -32.66 | 2 | 21 | 208 | 229 | 19 | Query: 3' gaGGUGGAGGAGGCGGAGGACc 5' | Ref: 5' ccTCTTGTCTTCCTCCTCCTGg 3' |
| hsa-miR-4459 | gi|619308620|gb|GAXD01012002.1| | #N/A | #N/A | 168 | -33.36 | 2 | 21 | 348 | 369 | 19 | Query: 3' gaGGUGGAGGAGGCGGAGGACc 5' | Ref: 5' acCCGTCCGCTCAGCCTCCTGg 3' |
| hsa-miR-4459 | gi|619308508|gb|GAXD01012114.1| | #N/A | #N/A | 164 | -32.96 | 2 | 21 | 303 | 324 | 19 | Query: 3' gaGGUGGAGGAGGCGGAGGACc 5' | Ref: 5' ttCCTTTATCTCCACCTCCTGg 3' |
| hsa-miR-4459 | gi|619308358|gb|GAXD01012264.1| | #N/A | #N/A | 156 | -30.97 | 2 | 21 | 225 | 246 | 19 | Query: 3' gaGGUGGAGGAGGCGGAGGACc 5' | Ref: 5' gcCCATCGAGCCCTCCTCCTGg 3' |
| hsa-miR-4459 | gi|619306464|gb|GAXD01014158.1| | #N/A | #N/A | 174 | -35.37 | 2 | 21 | 168 | 188 | 19 | Query: 3' gaGGUGGAGGAGGCGGAGGACc 5' | Ref: 5' ttTCACCCCCTCC-CCTCCTGc 3' |
| hsa-miR-4459 | gi|619306393|gb|GAXD01014229.1| | #N/A | #N/A | 151 | -30.27 | 2 | 21 | 186 | 208 | 20 | Query: 3' gaGGUGGAGGAGG-CGGAGGACc 5' | Ref: 5' ccCCTCCCCGACCTTCCTCCTGc 3' |
| hsa-miR-4459 | gi|619305779|gb|GAXD01014843.1| | #N/A | #N/A | 164 | -36.23 | 2 | 21 | 73 | 94 | 19 | Query: 3' gaGGUGGAGGAGGCGGAGGACc 5' | Ref: 5' ctCTTTCTCAACTGCCTCCTGg 3' |
| hsa-miR-4459 | gi|619305080|gb|GAXD01015542.1| | #N/A | #N/A | 176 | -33.38 | 2 | 21 | 305 | 326 | 19 | Query: 3' gaGGUGGAGGAGGCGGAGGACc 5' | Ref: 5' tcCCTCCTCCTCATCCTCCTGt 3' |
| hsa-miR-4459 | gi|619305039|gb|GAXD01015583.1| | #N/A | #N/A | 167 | -30.98 | 2 | 20 | 313 | 334 | 18 | Query: 3' gagGUGGAGGAGGCGGAGGACc 5' | Ref: 5' tggCCCCTCCTGTTCCTCCTGc 3' |
| hsa-miR-4459 | gi|619303960|gb|GAXD01016662.1| | #N/A | #N/A | 152 | -31.94 | 2 | 21 | 289 | 308 | 19 | Query: 3' gaGGUGGAGGAGGCGGAGGACc 5' | Ref: 5' ctCCA-CTCCAGT-CCTCCTGg 3' |
| hsa-miR-4459 | gi|619303940|gb|GAXD01016682.1| | #N/A | #N/A | 184 | -37.44 | 2 | 21 | 149 | 170 | 19 | Query: 3' gaGGUGGAGGAGGCGGAGGACc 5' | Ref: 5' caCCACCACCTCCACCTCCTGc 3' |
| hsa-miR-4459 | gi|619303782|gb|GAXD01016840.1| | #N/A | #N/A | 171 | -32.54 | 2 | 21 | 3 | 25 | 20 | Query: 3' gaGGU-GGAGGAGGCGGAGGACc 5' | Ref: 5' ccCCATCCTCCGTCTCCTCCTGa 3' |
| hsa-miR-4459 | gi|619303565|gb|GAXD01017057.1| | #N/A | #N/A | 167 | -30.63 | 2 | 18 | 22 | 42 | 16 | Query: 3' gagguGGAGGAGGCGGAGGACc 5' | Ref: 5' tcagcCCTCTTCC-CCTCCTGt 3' |
| hsa-miR-4459 | gi|619303164|gb|GAXD01017458.1| | #N/A | #N/A | 184 | -40.76 | 2 | 21 | 195 | 216 | 19 | Query: 3' gaGGUGGAGGAGGCGGAGGACc 5' | Ref: 5' ctCCTCCTCCTCCTCCTCCTGc 3' |
| hsa-miR-4459 | gi|619303164|gb|GAXD01017458.1| | #N/A | #N/A | 171 | -31.1 | 2 | 20 | 225 | 246 | 18 | Query: 3' gagGUGGAGGAGGCGGAGGACc 5' | Ref: 5' ctgCTCCTGCTCCTCCTCCTGc 3' |
| hsa-miR-4459 | gi|619303027|gb|GAXD01017595.1| | #N/A | #N/A | 180 | -35.27 | 2 | 21 | 26 | 47 | 19 | Query: 3' gaGGUGGAGGAGGCGGAGGACc 5' | Ref: 5' gaTCTCCTCCTCCTCCTCCTGc 3' |
| hsa-miR-4459 | gi|619302731|gb|GAXD01017891.1| | #N/A | #N/A | 156 | -33.44 | 2 | 21 | 14 | 35 | 19 | Query: 3' gaGGUGGAGGAGGCGGAGGACc 5' | Ref: 5' ctCTATCACTCAAGCCTCCTGg 3' |
| hsa-miR-4459 | gi|619302729|gb|GAXD01017893.1| | #N/A | #N/A | 156 | -33.44 | 2 | 21 | 148 | 169 | 19 | Query: 3' gaGGUGGAGGAGGCGGAGGACc 5' | Ref: 5' ctCTATCACTCAAGCCTCCTGg 3' |
| hsa-miR-4459 | gi|619301268|gb|GAXD01019354.1| | #N/A | #N/A | 171 | -31.3 | 2 | 21 | 127 | 149 | 20 | Query: 3' gaGGUGGAG-GAGGCGGAGGACc 5' | Ref: 5' caCCTCCTCGTTCCTCCTCCTGc 3' |
| hsa-miR-4459 | gi|619301000|gb|GAXD01019622.1| | #N/A | #N/A | 162 | -34.79 | 2 | 21 | 295 | 318 | 21 | Query: 3' gaGGUG-GAGGAGGC-GGAGGACc 5' | Ref: 5' ctCCACGCTCCGGTGCCCTCCTGt 3' |
| hsa-miR-4459 | gi|619300279|gb|GAXD01020343.1| | #N/A | #N/A | 155 | -30.64 | 2 | 21 | 752 | 774 | 20 | Query: 3' gaGGUGGAGGAGGC-GGAGGACc 5' | Ref: 5' cgTCGTCGTTGCCGCCCTCCTGg 3' |
| hsa-miR-4459 | gi|619298461|gb|GAXD01022160.1| | #N/A | #N/A | 166 | -33.65 | 2 | 20 | 61 | 83 | 19 | Query: 3' gagGUGGAGGAGGC-GGAGGACc 5' | Ref: 5' ccgCATCTTCGTCGTCCTCCTGg 3' |
| hsa-miR-4459 | gi|619298460|gb|GAXD01022161.1| | #N/A | #N/A | 166 | -33.65 | 2 | 20 | 61 | 83 | 19 | Query: 3' gagGUGGAGGAGGC-GGAGGACc 5' | Ref: 5' ccgCATCTTCGTCGTCCTCCTGg 3' |
| hsa-miR-4459 | gi|619298305|gb|GAXD01022316.1| | #N/A | #N/A | 171 | -31.71 | 2 | 20 | 194 | 215 | 18 | Query: 3' gagGUGGAGGAGGCGGAGGACc 5' | Ref: 5' ctgCACCAACTCCCCCTCCTGt 3' |
| hsa-miR-4459 | gi|619297879|gb|GAXD01022742.1| | #N/A | #N/A | 165 | -32.2 | 2 | 20 | 139 | 162 | 20 | Query: 3' gagGUG-GAGG-AGGCGGAGGACc 5' | Ref: 5' tcgCGCTCTCCGTGCGCCTCCTGc 3' |
| hsa-miR-4459 | gi|619297320|gb|GAXD01023301.1| | #N/A | #N/A | 145 | -30.6 | 2 | 21 | 38 | 64 | 24 | Query: 3' gaGGUGGAG--G--AG-GCGGAGGACc 5' | Ref: 5' taCCACTTCGACAATCAAACCTCCTGg 3' |
| hsa-miR-4459 | gi|619297320|gb|GAXD01023301.1| | #N/A | #N/A | 145 | -30.6 | 2 | 21 | 146 | 172 | 24 | Query: 3' gaGGUGGAG--G--AG-GCGGAGGACc 5' | Ref: 5' taCCACTTCGACAATCAAACCTCCTGg 3' |
| hsa-miR-4459 | gi|619297319|gb|GAXD01023302.1| | #N/A | #N/A | 145 | -30.6 | 2 | 21 | 81 | 107 | 24 | Query: 3' gaGGUGGAG--G--AG-GCGGAGGACc 5' | Ref: 5' taCCACTTCGACAATCAAACCTCCTGg 3' |
| hsa-miR-4459 | gi|619297319|gb|GAXD01023302.1| | #N/A | #N/A | 145 | -30.6 | 2 | 21 | 189 | 215 | 24 | Query: 3' gaGGUGGAG--G--AG-GCGGAGGACc 5' | Ref: 5' taCCACTTCGACAATCAAACCTCCTGg 3' |
| hsa-miR-4459 | gi|619297091|gb|GAXD01023530.1| | #N/A | #N/A | 163 | -31.87 | 2 | 18 | 11 | 31 | 16 | Query: 3' gagguGGAGGAGGCGGAGGACc 5' | Ref: 5' gaattCCT-CTCCTCCTCCTGg 3' |
| hsa-miR-4459 | gi|619295085|gb|GAXD01025053.1| | #N/A | #N/A | 158 | -30.28 | 2 | 21 | 199 | 219 | 19 | Query: 3' gaGGUGGAGGAGGCGGAGGACc 5' | Ref: 5' caCAGTGTTCTCC-CCTCCTGg 3' |
| hsa-miR-4459 | gi|619294732|gb|GAXD01025406.1| | #N/A | #N/A | 176 | -39.5 | 2 | 21 | 29 | 50 | 19 | Query: 3' gaGGUGGAGGAGGCGGAGGACc 5' | Ref: 5' caCTCCCTCCGCTGCCTCCTGg 3' |
| hsa-miR-4459 | gi|619294372|gb|GAXD01025766.1| | #N/A | #N/A | 164 | -31.82 | 2 | 21 | 30 | 49 | 19 | Query: 3' gaGGUGGAGGAGGCGGAGGACc 5' | Ref: 5' gcCCACC-CCGCC-CCTCCTGc 3' |
| hsa-miR-4459 | gi|619293669|gb|GAXD01026469.1| | #N/A | #N/A | 162 | -32.29 | 2 | 15 | 5 | 26 | 13 | Query: 3' gagguggaGGAGGCGGAGGACc 5' | Ref: 5' cctctggcCCTGCGCCTCCTGc 3' |
| hsa-miR-4459 | gi|619293231|gb|GAXD01026907.1| | #N/A | #N/A | 154 | -30.78 | 2 | 20 | 219 | 243 | 21 | Query: 3' gagGUGGAGGAGG---CGGAGGACc 5' | Ref: 5' gaaCCTTTCCACCAAGGCCTCCTGg 3' |
| hsa-miR-4459 | gi|619292598|gb|GAXD01027540.1| | #N/A | #N/A | 172 | -32.33 | 2 | 21 | 87 | 108 | 19 | Query: 3' gaGGUGGAGGAGGCGGAGGACc 5' | Ref: 5' caCCACCTTCAACCCCTCCTGc 3' |
| hsa-miR-4459 | gi|619292399|gb|GAXD01027739.1| | #N/A | #N/A | 153 | -33.09 | 2 | 20 | 96 | 119 | 20 | Query: 3' gagGUGGAG-GAGGC-GGAGGACc 5' | Ref: 5' ctaCTCCGCTCGCCGCCCTCCTGa 3' |
| hsa-miR-4459 | gi|619292322|gb|GAXD01027816.1| | #N/A | #N/A | 161 | -31.35 | 2 | 19 | 208 | 230 | 18 | Query: 3' gaggUGGAGGAG-GCGGAGGACc 5' | Ref: 5' tttgGTCTTCTCTCCCCTCCTGg 3' |
| hsa-miR-4459 | gi|619292035|gb|GAXD01028103.1| | #N/A | #N/A | 177 | -33.82 | 2 | 18 | 117 | 138 | 16 | Query: 3' gagguGGAGGAGGCGGAGGACc 5' | Ref: 5' ctgacCCTCCTCCTCCTCCTGc 3' |
| hsa-miR-4459 | gi|619292035|gb|GAXD01028103.1| | #N/A | #N/A | 168 | -31.96 | 2 | 21 | 33 | 54 | 19 | Query: 3' gaGGUGGAGGAGGCGGAGGACc 5' | Ref: 5' ctCCTCGCCCTCCTCCTCCTGc 3' |
| hsa-miR-4459 | gi|619292035|gb|GAXD01028103.1| | #N/A | #N/A | 167 | -33.48 | 2 | 21 | 96 | 120 | 22 | Query: 3' gaGGUGGA---GGAGGCGGAGGACc 5' | Ref: 5' ctCCACTTGCCCCTCTTCCTCCTGa 3' |
| hsa-miR-4459 | gi|619291584|gb|GAXD01028554.1| | #N/A | #N/A | 160 | -31.27 | 2 | 21 | 436 | 457 | 19 | Query: 3' gaGGUGGAGGAGGCGGAGGACc 5' | Ref: 5' ctCCAGTGTGCCCGCCTCCTGc 3' |
| hsa-miR-4459 | gi|619291165|gb|GAXD01028973.1| | #N/A | #N/A | 163 | -30.7 | 2 | 20 | 32 | 53 | 18 | Query: 3' gagGUGGAGGAGGCGGAGGACc 5' | Ref: 5' gtgTGTCTACTGTGCCTCCTGg 3' |
| hsa-miR-4459 | gi|619290936|gb|GAXD01029202.1| | #N/A | #N/A | 172 | -32.27 | 2 | 21 | 156 | 177 | 19 | Query: 3' gaGGUGGAGGAGGCGGAGGACc 5' | Ref: 5' ctTTGCCTCCCCCTCCTCCTGc 3' |
| hsa-miR-4459 | gi|619290347|gb|GAXD01029791.1| | #N/A | #N/A | 179 | -31.59 | 2 | 20 | 117 | 138 | 18 | Query: 3' gagGUGGAGGAGGCGGAGGACc 5' | Ref: 5' gaaCACATCCTCCTCCTCCTGa 3' |
| hsa-miR-4459 | gi|619290289|gb|GAXD01029849.1| | #N/A | #N/A | 165 | -32.81 | 2 | 20 | 20 | 40 | 18 | Query: 3' gagGUGGAGGAGGCGGAGGACc 5' | Ref: 5' aaaCACC-CACCCGCCTCCTGc 3' |
| hsa-miR-4459 | gi|619290013|gb|GAXD01030125.1| | #N/A | #N/A | 163 | -33.49 | 2 | 20 | 179 | 200 | 18 | Query: 3' gagGUGGAGGAGGCGGAGGACc 5' | Ref: 5' ctgCCCCAGCTCCACCTCCTGg 3' |
| hsa-miR-4459 | gi|619289556|gb|GAXD01030582.1| | #N/A | #N/A | 162 | -30.15 | 2 | 21 | 113 | 136 | 21 | Query: 3' gaGGUGGA-GGA-GGCGGAGGACc 5' | Ref: 5' caCCGCCTGCCTGTTTCCTCCTGa 3' |
| hsa-miR-4459 | gi|619289529|gb|GAXD01030609.1| | #N/A | #N/A | 158 | -30.2 | 2 | 20 | 197 | 220 | 20 | Query: 3' gagGUGGAGGA--GGCGGAGGACc 5' | Ref: 5' cgaCATTGCCTGATTGCCTCCTGg 3' |
| hsa-miR-4638-3p | gi|619320161|gb|GAXD01000588.1| | #N/A | #N/A | 165 | -30.98 | 2 | 22 | 818 | 838 | 20 | Query: 3' gcCGGCCGACUCGCCACAGGUCc 5' | Ref: 5' ttGCTGG-TGAG-GCTGTCCAGg 3' |
| hsa-miR-4638-3p | gi|619309222|gb|GAXD01011400.1| | #N/A | #N/A | 162 | -32.59 | 2 | 21 | 13 | 34 | 19 | Query: 3' gccGGCCGACUCGCCACAGGUCc 5' | Ref: 5' cgtCAGGCGCAG-GGTGTCCAGg 3' |
| hsa-miR-4638-3p | gi|619306645|gb|GAXD01013977.1| | #N/A | #N/A | 158 | -30.95 | 2 | 17 | 186 | 210 | 17 | Query: 3' gccggccGAC-UCG-CCACAGGUCc 5' | Ref: 5' gaagcacTTGAAGCAGGTGTCCAGg 3' |
| hsa-miR-4638-3p | gi|619306307|gb|GAXD01014315.1| | #N/A | #N/A | 155 | -31.41 | 2 | 22 | 29 | 48 | 21 | Query: 3' gcCG-GCCGACUCGCCACAGGUCc 5' | Ref: 5' caGCACGGCTGA----TGTCCAGg 3' |
| hsa-miR-4638-3p | gi|619300022|gb|GAXD01020600.1| | #N/A | #N/A | 166 | -31.72 | 2 | 22 | 785 | 808 | 22 | Query: 3' gcCGGCCG--ACUCGCCACAGGUCc 5' | Ref: 5' acGTCAGCGATGAGC-GTGTCCAGg 3' |
| hsa-miR-4638-3p | gi|619299083|gb|GAXD01021538.1| | #N/A | #N/A | 165 | -36.79 | 2 | 19 | 185 | 208 | 18 | Query: 3' gccggCCGAC-UCGCCACAGGUCc 5' | Ref: 5' acagaGGCGGTGGTGGTGTCCAGg 3' |
| hsa-miR-4638-3p | gi|619295228|gb|GAXD01024910.1| | #N/A | #N/A | 165 | -30.98 | 2 | 22 | 721 | 741 | 20 | Query: 3' gcCGGCCGACUCGCCACAGGUCc 5' | Ref: 5' ttGCTGG-TGAG-GCTGTCCAGg 3' |
| hsa-miR-4638-3p | gi|619290791|gb|GAXD01029347.1| | #N/A | #N/A | 163 | -30.64 | 2 | 20 | 118 | 138 | 18 | Query: 3' gccgGCCGACUCGCCACAGGUCc 5' | Ref: 5' gtagCGGC-GGGC-GTGTCCAGc 3' |
| mmu-miR-5625-5p | gi|619304441|gb|GAXD01016181.1| | #N/A | #N/A | 165 | -30.79 | 2 | 19 | 190 | 211 | 18 | Query: 3' aggAUG-AGUUCUUGAAGGCCc 5' | Ref: 5' cagTACAGCGAGGACTTCCGGg 3' |
| mja-miR-6493-5p | gi|619316799|gb|GAXD01003950.1| | #N/A | #N/A | 151 | -31.06 | 2 | 21 | 227 | 251 | 22 | Query: 3' ucCCCAU---UUUGGACGGCCUGCa 5' | Ref: 5' ggGGGTACTTGTGCGGGCCGGACGa 3' |
| mja-miR-6493-5p | gi|619306805|gb|GAXD01013817.1| | #N/A | #N/A | 163 | -30.18 | 2 | 21 | 17 | 40 | 21 | Query: 3' ucCCCAUUUUGG--ACGGCCUGCa 5' | Ref: 5' tcGGGGAGGACCCTTTCCGGACGg 3' |
| mja-miR-6493-5p | gi|619299360|gb|GAXD01021261.1| | #N/A | #N/A | 141 | -30.57 | 2 | 21 | 2656 | 2680 | 23 | Query: 3' ucCCCAUUUUGGAC----GGCCUGCa 5' | Ref: 5' ggGGGT-CTCCCTGGCACCCGGACGg 3' |
| mja-miR-6493-5p | gi|619299359|gb|GAXD01021262.1| | #N/A | #N/A | 141 | -30.57 | 2 | 21 | 2553 | 2577 | 23 | Query: 3' ucCCCAUUUUGGAC----GGCCUGCa 5' | Ref: 5' ggGGGT-CTCCCTGGCACCCGGACGg 3' |
| mja-miR-6493-5p | gi|619293879|gb|GAXD01026259.1| | #N/A | #N/A | 164 | -30.96 | 2 | 21 | 243 | 264 | 19 | Query: 3' ucCCCAUUUUGGACGGCCUGCa 5' | Ref: 5' caGGGAGGGGGTTGCCGGACGg 3' |
| mja-miR-6493-5p | gi|619290270|gb|GAXD01029868.1| | #N/A | #N/A | 160 | -32.69 | 2 | 17 | 5 | 26 | 15 | Query: 3' uccccaUUUUGGACGGCCUGCa 5' | Ref: 5' actgagGGGACCTTCCGGACGt 3' |
| ssa-miR-15a-5p | gi|619290818|gb|GAXD01029320.1| | #N/A | #N/A | 166 | -31.89 | 2 | 20 | 163 | 186 | 19 | Query: 3' uguuUGGU-ACUGCACGACGAUGc 5' | Ref: 5' ccctGCCACACACGTGCTGCTACg 3' |
| ssa-miR-15a-5p | gi|619290587|gb|GAXD01029551.1| | #N/A | #N/A | 175 | -32.65 | 2 | 22 | 292 | 313 | 20 | Query: 3' ugUUUGGUACUGCACGACGAUGc 5' | Ref: 5' tgAGCCCGT-ACGTGCTGCTACg 3' |
|  |  |  |  |  |  |  |  |  |  |  |  |  |
| **The first report of miRNAome from a thysanopteran insect, Thrips palmi Karny using high-throughput sequencing.**  **Authors : K. B. Rebijith, R. Asokan, H. Ranjitha Hande and N. K. Krishna Kumar** | | | | | | | | | | | | |
