# Supplementary material for: The First Report of miRNAs from a Thysanopteran Insect, Thrips palmi Karny Using High-Throughput Sequencing
Source: PLoS One. 2016 Sep 29;11(9):e0163635. doi: 10.1371/journal.pone.0163635 (PMC5042526; doi:10.1371/journal.pone.0163635)
Supplement: S4 Table — (DOC) [file pone.0163635.s004.doc]

| **Supplementary Table S4 Complete Functional categories of gene ontology classification of the putative target genes for the T. palmi miRNAs against ESTs of *F. occidentalis.*** | | |
| --- | --- | --- |
| **Target Id** | **Functional Categories** | **Functional Class** |
| gi|297502781|gb|GT298823.1|GT298823 | integral component of membrane | C |
| gi|297503334|gb|GT311546.1|GT311546 | mitochondrion | C |
| gi|297503678|gb|GT311702.1|GT311702 | ribosome | C |
| gi|297505989|gb|GT301792.1|GT301792 | membrane | C |
| gi|297508542|gb|GT299907.1|GT299907 | mitochondrial inner membrane | C |
| gi|297508542|gb|GT299907.1|GT299907 | respiratory chain | C |
| gi|297508542|gb|GT299907.1|GT299907 | integral component of membrane | C |
| gi|297510320|gb|GT308705.1|GT308705 | membrane coat | C |
| gi|297511947|gb|GT299620.1|GT299620 | ribosome | C |
| gi|297514625|gb|GT300247.1|GT300247 | ribosome | C |
| gi|297514916|gb|GT308368.1|GT308368 | mitochondrial intermembrane space | C |
| gi|297502781|gb|GT298823.1|GT298823 | octopamine receptor activity | F |
| gi|297503218|gb|GT303535.1|GT303535 | oxidoreductase activity, acting on CH-OH group of donors | F |
| gi|297503334|gb|GT311546.1|GT311546 | methylmalonate-semialdehyde dehydrogenase (acylating) activity | F |
| gi|297503348|gb|GT311517.1|GT311517 | catalytic activity | F |
| gi|297503678|gb|GT311702.1|GT311702 | structural constituent of ribosome | F |
| gi|297503678|gb|GT311702.1|GT311702 | rRNA binding | F |
| gi|297503792|gb|GT302478.1|GT302478 | spermine synthase activity | F |
| gi|297504051|gb|GT302651.1|GT302651 | ligase activity | F |
| gi|297505076|gb|GT309965.1|GT309965 | catalytic activity | F |
| gi|297506043|gb|GT309490.1|GT309490 | nucleotide binding | F |
| gi|297507013|gb|GT307884.1|GT307884 | hydrolase activity | F |
| gi|297508542|gb|GT299907.1|GT299907 | copper ion binding | F |
| gi|297508542|gb|GT299907.1|GT299907 | cytochrome-c oxidase activity | F |
| gi|297508675|gb|GT302497.1|GT302497 | binding | F |
| gi|297510320|gb|GT308705.1|GT308705 | protein transporter activity | F |
| gi|297510415|gb|GT298587.1|GT298587 | translation initiation factor activity | F |
| gi|297510447|gb|GT308789.1|GT308789 | RNA binding | F |
| gi|297510657|gb|GT303130.1|GT303130 | transferase activity, transferring acyl groups other than amino-acyl groups | F |
| gi|297511947|gb|GT299620.1|GT299620 | structural constituent of ribosome | F |
| gi|297511947|gb|GT299620.1|GT299620 | ubiquitin thiolesterase activity | F |
| gi|297512905|gb|GT311911.1|GT311911 | homogentisate 1,2-dioxygenase activity | F |
| gi|297513287|gb|GT298882.1|GT298882 | ion binding | F |
| gi|297513287|gb|GT298882.1|GT298882 | oxidoreductase activity, acting on paired donors, with incorporation or reduction of molecular oxygen, reduced pteridine as one donor, and incorporation of one atom of oxygen | F |
| gi|297513834|gb|GT303969.1|GT303969 | catalytic activity | F |
| gi|297514625|gb|GT300247.1|GT300247 | structural constituent of ribosome | F |
| gi|297514916|gb|GT308368.1|GT308368 | adenylate kinase activity | F |
| gi|297514916|gb|GT308368.1|GT308368 | ATP binding | F |
| gi|297502781|gb|GT298823.1|GT298823 | G-protein coupled receptor signaling pathway | P |
| gi|297503334|gb|GT311546.1|GT311546 | valine metabolic process | P |
| gi|297503334|gb|GT311546.1|GT311546 | pyrimidine nucleobase metabolic process | P |
| gi|297503678|gb|GT311702.1|GT311702 | translation | P |
| gi|297503792|gb|GT302478.1|GT302478 | spermine biosynthetic process | P |
| gi|297504051|gb|GT302651.1|GT302651 | metabolic process | P |
| gi|297505076|gb|GT309965.1|GT309965 | metabolic process | P |
| gi|297508542|gb|GT299907.1|GT299907 | transport | P |
| gi|297508542|gb|GT299907.1|GT299907 | electron transport chain | P |
| gi|297508675|gb|GT302497.1|GT302497 | organic substance metabolic process | P |
| gi|297508675|gb|GT302497.1|GT302497 | cellular metabolic process | P |
| gi|297508675|gb|GT302497.1|GT302497 | single-organism metabolic process | P |
| gi|297508675|gb|GT302497.1|GT302497 | primary metabolic process | P |
| gi|297510320|gb|GT308705.1|GT308705 | vesicle-mediated transport | P |
| gi|297510320|gb|GT308705.1|GT308705 | intracellular protein transport | P |
| gi|297510415|gb|GT298587.1|GT298587 | RNA metabolic process | P |
| gi|297510415|gb|GT298587.1|GT298587 | translational initiation | P |
| gi|297510447|gb|GT308789.1|GT308789 | translation | P |
| gi|297510657|gb|GT303130.1|GT303130 | metabolic process | P |
| gi|297511947|gb|GT299620.1|GT299620 | ubiquitin-dependent protein catabolic process | P |
| gi|297511947|gb|GT299620.1|GT299620 | translation | P |
| gi|297512905|gb|GT311911.1|GT311911 | L-phenylalanine catabolic process | P |
| gi|297512905|gb|GT311911.1|GT311911 | oxidation-reduction process | P |
| gi|297512905|gb|GT311911.1|GT311911 | tyrosine metabolic process | P |
| gi|297513287|gb|GT298882.1|GT298882 | aromatic amino acid family metabolic process | P |
| gi|297513834|gb|GT303969.1|GT303969 | metabolic process | P |
| gi|297514625|gb|GT300247.1|GT300247 | translation | P |
| gi|297514916|gb|GT308368.1|GT308368 | nucleotide phosphorylation | P |
|  |  |  |
| **F** | **Molecular Function** |  |
| **P** | **Biological Process** |  |
| **C** | **Cellular Component** |  |
| **The first report of miRNAome from a thysanopteran insect, Thrips palmi Karny using high-throughput sequencing.**  **Authors : K. B. Rebijith, R. Asokan, H. Ranjitha Hande and N. K. Krishna Kumar** | | |
