# Supplementary material for: The First Report of miRNAs from a Thysanopteran Insect, Thrips palmi Karny Using High-Throughput Sequencing
Source: PLoS One. 2016 Sep 29;11(9):e0163635. doi: 10.1371/journal.pone.0163635 (PMC5042526; doi:10.1371/journal.pone.0163635)
Supplement: S5 Table — (DOC) [file pone.0163635.s005.doc]

| **Supplementary Table S5.**  **Complete Functional categories of gene ontology classification of the putative target genes for the known miRNAs against transcriptome sequences of *F. occidentalis*.** | | |
| --- | --- | --- |
| **Target Id** | **Functional Categories** | **Functional Class** |
| gi|619289128|gb|GAXD01031010.1| | biosynthetic process | P |
| gi|619289128|gb|GAXD01031010.1| | nucleotidyltransferase activity | F |
| gi|619291737|gb|GAXD01028401.1| | protein dephosphorylation | P |
| gi|619291737|gb|GAXD01028401.1| | protein tyrosine phosphatase activity | F |
| gi|619291737|gb|GAXD01028401.1| | rhodopsin kinase activity | F |
| gi|619292226|gb|GAXD01027912.1| | microtubule-based movement | P |
| gi|619292226|gb|GAXD01027912.1| | ATP binding | F |
| gi|619292226|gb|GAXD01027912.1| | microtubule motor activity | F |
| gi|619292226|gb|GAXD01027912.1| | microtubule | C |
| gi|619293840|gb|GAXD01026298.1| | transferase activity | F |
| gi|619293993|gb|GAXD01026145.1| | carboxylic ester hydrolase activity | F |
| gi|619294508|gb|GAXD01025630.1| | actin binding | F |
| gi|619294914|gb|GAXD01025224.1| | aromatase activity | F |
| gi|619294914|gb|GAXD01025224.1| | oxidation-reduction process | P |
| gi|619295064|gb|GAXD01025074.1| | cation transmembrane transporter activity | F |
| gi|619295064|gb|GAXD01025074.1| | cation transport | P |
| gi|619295095|gb|GAXD01025043.1| | transport | P |
| gi|619295106|gb|GAXD01025032.1| | integral component of membrane | C |
| gi|619295106|gb|GAXD01025032.1| | organic phosphonate transmembrane-transporting ATPase activity | F |
| gi|619295106|gb|GAXD01025032.1| | ATP catabolic process | P |
| gi|619295106|gb|GAXD01025032.1| | ATP binding | F |
| gi|619295142|gb|GAXD01024996.1| | metal ion binding | F |
| gi|619295167|gb|GAXD01024971.1| | carbohydrate binding | F |
| gi|619295167|gb|GAXD01024971.1| | carbohydrate metabolic process | P |
| gi|619295167|gb|GAXD01024971.1| | isomerase activity | F |
| gi|619295318|gb|GAXD01024820.1| | binding | F |
| gi|619295318|gb|GAXD01024820.1| | hydrolase activity | F |
| gi|619295715|gb|GAXD01024423.1| | intracellular signal transduction | P |
| gi|619295715|gb|GAXD01024423.1| | signal transducer activity | F |
| gi|619295715|gb|GAXD01024423.1| | phosphatidylinositol phospholipase C activity | F |
| gi|619295715|gb|GAXD01024423.1| | lipid catabolic process | P |
| gi|619295715|gb|GAXD01024423.1| | calcium ion binding | F |
| gi|619295785|gb|GAXD01024353.1| | fatty-acyl-CoA binding | F |
| gi|619296120|gb|GAXD01024018.1| | cellular carbohydrate metabolic process | P |
| gi|619296120|gb|GAXD01024018.1| | mitochondrial matrix | C |
| gi|619296120|gb|GAXD01024018.1| | citrate (Si)-synthase activity | F |
| gi|619296120|gb|GAXD01024018.1| | tricarboxylic acid cycle | P |
| gi|619296185|gb|GAXD01023953.1| | zinc ion binding | F |
| gi|619296185|gb|GAXD01023953.1| | oxidoreductase activity | F |
| gi|619296185|gb|GAXD01023953.1| | oxidation-reduction process | P |
| gi|619296927|gb|GAXD01023694.1| | ATP binding | F |
| gi|619296927|gb|GAXD01023694.1| | response to stress | P |
| gi|619297000|gb|GAXD01023621.1| | transport | P |
| gi|619297263|gb|GAXD01023358.1| | zinc ion binding | F |
| gi|619297263|gb|GAXD01023358.1| | ligase activity | F |
| gi|619297567|gb|GAXD01023054.1| | oxidoreductase activity, acting on paired donors, with incorporation or reduction of molecular oxygen, reduced ascorbate as one donor, and incorporation of one atom of oxygen | F |
| gi|619297567|gb|GAXD01023054.1| | metabolic process | P |
| gi|619297567|gb|GAXD01023054.1| | cellular process | P |
| gi|619297568|gb|GAXD01023053.1| | copper ion binding | F |
| gi|619297568|gb|GAXD01023053.1| | membrane | C |
| gi|619297568|gb|GAXD01023053.1| | peptide metabolic process | P |
| gi|619297568|gb|GAXD01023053.1| | peptidylglycine monooxygenase activity | F |
| gi|619297568|gb|GAXD01023053.1| | oxidation-reduction process | P |
| gi|619297593|gb|GAXD01023028.1| | intracellular | C |
| gi|619297593|gb|GAXD01023028.1| | cellular protein modification process | P |
| gi|619297593|gb|GAXD01023028.1| | acid-amino acid ligase activity | F |
| gi|619297953|gb|GAXD01022668.1| | Golgi membrane | C |
| gi|619297953|gb|GAXD01022668.1| | beta-1,3-galactosyl-O-glycosyl-glycoprotein beta-1,3-N-acetylglucosaminyltransferase activity | F |
| gi|619297953|gb|GAXD01022668.1| | alpha-1,3-mannosylglycoprotein 2-beta-N-acetylglucosaminyltransferase activity | F |
| gi|619297953|gb|GAXD01022668.1| | protein O-linked glycosylation | P |
| gi|619297953|gb|GAXD01022668.1| | integral component of membrane | C |
| gi|619297953|gb|GAXD01022668.1| | protein N-linked glycosylation | P |
| gi|619297967|gb|GAXD01022654.1| | kinase activity | F |
| gi|619297991|gb|GAXD01022630.1| | ribosome | C |
| gi|619297991|gb|GAXD01022630.1| | structural constituent of ribosome | F |
| gi|619297991|gb|GAXD01022630.1| | translation | P |
| gi|619298015|gb|GAXD01022606.1| | steroid hormone mediated signaling pathway | P |
| gi|619298015|gb|GAXD01022606.1| | zinc ion binding | F |
| gi|619298015|gb|GAXD01022606.1| | steroid binding | F |
| gi|619298015|gb|GAXD01022606.1| | regulation of transcription, DNA-templated | P |
| gi|619298015|gb|GAXD01022606.1| | sequence-specific DNA binding | F |
| gi|619298015|gb|GAXD01022606.1| | steroid hormone receptor activity | F |
| gi|619298015|gb|GAXD01022606.1| | ligand-activated sequence-specific DNA binding RNA polymerase II transcription factor activity | F |
| gi|619298015|gb|GAXD01022606.1| | nucleus | C |
| gi|619298015|gb|GAXD01022606.1| | transcription, DNA-templated | P |
| gi|619298016|gb|GAXD01022605.1| | steroid hormone mediated signaling pathway | P |
| gi|619298016|gb|GAXD01022605.1| | zinc ion binding | F |
| gi|619298016|gb|GAXD01022605.1| | steroid binding | F |
| gi|619298016|gb|GAXD01022605.1| | regulation of transcription, DNA-templated | P |
| gi|619298016|gb|GAXD01022605.1| | sequence-specific DNA binding | F |
| gi|619298016|gb|GAXD01022605.1| | steroid hormone receptor activity | F |
| gi|619298016|gb|GAXD01022605.1| | ligand-activated sequence-specific DNA binding RNA polymerase II transcription factor activity | F |
| gi|619298016|gb|GAXD01022605.1| | nucleus | C |
| gi|619298016|gb|GAXD01022605.1| | transcription, DNA-templated | P |
| gi|619298186|gb|GAXD01022435.1| | signal transduction | P |
| gi|619298258|gb|GAXD01022363.1| | single-organism developmental process | P |
| gi|619298287|gb|GAXD01022334.1| | nucleic acid binding | F |
| gi|619298287|gb|GAXD01022334.1| | nucleotide binding | F |
| gi|619298357|gb|GAXD01022264.1| | purine-nucleoside phosphorylase activity | F |
| gi|619298357|gb|GAXD01022264.1| | nucleoside metabolic process | P |
| gi|619298518|gb|GAXD01022103.1| | glutathione transferase activity | F |
| gi|619298518|gb|GAXD01022103.1| | translation elongation factor activity | F |
| gi|619298518|gb|GAXD01022103.1| | eukaryotic translation elongation factor 1 complex | C |
| gi|619298518|gb|GAXD01022103.1| | translational elongation | P |
| gi|619298520|gb|GAXD01022101.1| | translation elongation factor activity | F |
| gi|619298520|gb|GAXD01022101.1| | eukaryotic translation elongation factor 1 complex | C |
| gi|619298520|gb|GAXD01022101.1| | translational elongation | P |
| gi|619298543|gb|GAXD01022078.1| | hydrolase activity | F |
| gi|619299000|gb|GAXD01021621.1| | plasma membrane part | C |
| gi|619299000|gb|GAXD01021621.1| | transmembrane transport | P |
| gi|619299000|gb|GAXD01021621.1| | trehalose transport | P |
| gi|619299000|gb|GAXD01021621.1| | trehalose transmembrane transporter activity | F |
| gi|619299000|gb|GAXD01021621.1| | integral component of membrane | C |
| gi|619299001|gb|GAXD01021620.1| | nucleic acid binding | F |
| gi|619299001|gb|GAXD01021620.1| | nucleotide binding | F |
| gi|619299095|gb|GAXD01021526.1| | transport | P |
| gi|619299128|gb|GAXD01021493.1| | cytoskeleton | C |
| gi|619299128|gb|GAXD01021493.1| | calcium ion binding | F |
| gi|619299128|gb|GAXD01021493.1| | cell cycle arrest | P |
| gi|619299248|gb|GAXD01021373.1| | binding | F |
| gi|619299249|gb|GAXD01021372.1| | binding | F |
| gi|619299265|gb|GAXD01021356.1| | nucleic acid binding | F |
| gi|619299266|gb|GAXD01021355.1| | nucleic acid binding | F |
| gi|619299267|gb|GAXD01021354.1| | nucleic acid binding | F |
| gi|619299293|gb|GAXD01021328.1| | metabolic process | P |
| gi|619299293|gb|GAXD01021328.1| | catalytic activity | F |
| gi|619299574|gb|GAXD01021047.1| | protein domain specific binding | F |
| gi|619299712|gb|GAXD01020909.1| | protein ubiquitination | P |
| gi|619299712|gb|GAXD01020909.1| | zinc ion binding | F |
| gi|619299712|gb|GAXD01020909.1| | ligase activity | F |
| gi|619299712|gb|GAXD01020909.1| | ubiquitin-protein transferase activity | F |
| gi|619299765|gb|GAXD01020856.1| | cellular metabolic process | P |
| gi|619299765|gb|GAXD01020856.1| | coenzyme binding | F |
| gi|619299765|gb|GAXD01020856.1| | catalytic activity | F |
| gi|619299999|gb|GAXD01020623.1| | SRP-dependent cotranslational protein targeting to membrane | P |
| gi|619299999|gb|GAXD01020623.1| | signal recognition particle | C |
| gi|619299999|gb|GAXD01020623.1| | 7S RNA binding | F |
| gi|619300227|gb|GAXD01020395.1| | nucleus | C |
| gi|619300227|gb|GAXD01020395.1| | ribosome biogenesis | P |
| gi|619300248|gb|GAXD01020374.1| | transferase activity, transferring acyl groups | F |
| gi|619300248|gb|GAXD01020374.1| | metabolic process | P |
| gi|619300250|gb|GAXD01020372.1| | transferase activity, transferring acyl groups | F |
| gi|619300250|gb|GAXD01020372.1| | metabolic process | P |
| gi|619300310|gb|GAXD01020312.1| | zinc ion binding | F |
| gi|619300310|gb|GAXD01020312.1| | nucleic acid binding | F |
| gi|619300310|gb|GAXD01020312.1| | nucleotide binding | F |
| gi|619300311|gb|GAXD01020311.1| | zinc ion binding | F |
| gi|619300311|gb|GAXD01020311.1| | nucleic acid binding | F |
| gi|619300311|gb|GAXD01020311.1| | nucleotide binding | F |
| gi|619300330|gb|GAXD01020292.1| | protein phosphatase type 2A complex | C |
| gi|619300330|gb|GAXD01020292.1| | signal transduction | P |
| gi|619300330|gb|GAXD01020292.1| | binding | F |
| gi|619300330|gb|GAXD01020292.1| | protein phosphatase type 2A regulator activity | F |
| gi|619300331|gb|GAXD01020291.1| | protein phosphatase type 2A complex | C |
| gi|619300331|gb|GAXD01020291.1| | signal transduction | P |
| gi|619300331|gb|GAXD01020291.1| | binding | F |
| gi|619300331|gb|GAXD01020291.1| | protein phosphatase type 2A regulator activity | F |
| gi|619300385|gb|GAXD01020237.1| | glutathione transferase activity | F |
| gi|619300545|gb|GAXD01020077.1| | phospholipid binding | F |
| gi|619301231|gb|GAXD01019391.1| | DNA binding | F |
| gi|619301448|gb|GAXD01019174.1| | kinase activity | F |
| gi|619301479|gb|GAXD01019143.1| | heme binding | F |
| gi|619301479|gb|GAXD01019143.1| | monooxygenase activity | F |
| gi|619301479|gb|GAXD01019143.1| | iron ion binding | F |
| gi|619301479|gb|GAXD01019143.1| | electron carrier activity | F |
| gi|619301479|gb|GAXD01019143.1| | oxidation-reduction process | P |
| gi|619301712|gb|GAXD01018910.1| | transport | P |
| gi|619302625|gb|GAXD01017997.1| | ribosome | C |
| gi|619302625|gb|GAXD01017997.1| | structural constituent of ribosome | F |
| gi|619302625|gb|GAXD01017997.1| | translation | P |
| gi|619303816|gb|GAXD01016806.1| | binding | F |
| gi|619303891|gb|GAXD01016731.1| | nucleic acid binding | F |
| gi|619303891|gb|GAXD01016731.1| | ATP binding | F |
| gi|619303891|gb|GAXD01016731.1| | ATP-dependent helicase activity | F |
| gi|619304058|gb|GAXD01016564.1| | carbohydrate metabolic process | P |
| gi|619304058|gb|GAXD01016564.1| | phosphotransferase activity, alcohol group as acceptor | F |
| gi|619304058|gb|GAXD01016564.1| | kinase activity | F |
| gi|619304058|gb|GAXD01016564.1| | cellular metabolic process | P |
| gi|619304058|gb|GAXD01016564.1| | nucleotide binding | F |
| gi|619304279|gb|GAXD01016343.1| | small GTPase mediated signal transduction | P |
| gi|619304279|gb|GAXD01016343.1| | intracellular | C |
| gi|619304279|gb|GAXD01016343.1| | GTP catabolic process | P |
| gi|619304279|gb|GAXD01016343.1| | GTPase activity | F |
| gi|619304279|gb|GAXD01016343.1| | membrane | C |
| gi|619304279|gb|GAXD01016343.1| | GTP binding | F |
| gi|619304731|gb|GAXD01015891.1| | single-organism process | P |
| gi|619304731|gb|GAXD01015891.1| | transport | P |
| gi|619305564|gb|GAXD01015058.1| | oxygen transport | P |
| gi|619305564|gb|GAXD01015058.1| | oxygen transporter activity | F |
| gi|619305660|gb|GAXD01014962.1| | metalloendopeptidase activity | F |
| gi|619305660|gb|GAXD01014962.1| | proteolysis | P |
| gi|619306138|gb|GAXD01014484.1| | clathrin adaptor complex | C |
| gi|619306138|gb|GAXD01014484.1| | vesicle-mediated transport | P |
| gi|619306138|gb|GAXD01014484.1| | intracellular protein transport | P |
| gi|619306139|gb|GAXD01014483.1| | clathrin adaptor complex | C |
| gi|619306139|gb|GAXD01014483.1| | vesicle-mediated transport | P |
| gi|619306139|gb|GAXD01014483.1| | intracellular protein transport | P |
| gi|619306310|gb|GAXD01014312.1| | proteinaceous extracellular matrix | C |
| gi|619306335|gb|GAXD01014287.1| | DNA binding | F |
| gi|619306825|gb|GAXD01013797.1| | 5-aminolevulinate synthase activity | F |
| gi|619306825|gb|GAXD01013797.1| | pyridoxal phosphate binding | F |
| gi|619306825|gb|GAXD01013797.1| | tetrapyrrole biosynthetic process | P |
| gi|619306825|gb|GAXD01013797.1| | transferase activity, transferring nitrogenous groups | F |
| gi|619306829|gb|GAXD01013793.1| | biosynthetic process | P |
| gi|619306829|gb|GAXD01013793.1| | transferase activity | F |
| gi|619307186|gb|GAXD01013436.1| | microtubule | C |
| gi|619307186|gb|GAXD01013436.1| | protein polymerization | P |
| gi|619307186|gb|GAXD01013436.1| | GTP catabolic process | P |
| gi|619307186|gb|GAXD01013436.1| | microtubule-based movement | P |
| gi|619307186|gb|GAXD01013436.1| | GTPase activity | F |
| gi|619307186|gb|GAXD01013436.1| | structural molecule activity | F |
| gi|619307186|gb|GAXD01013436.1| | cytoplasm | C |
| gi|619307186|gb|GAXD01013436.1| | GTP binding | F |
| gi|619307188|gb|GAXD01013434.1| | microtubule | C |
| gi|619307188|gb|GAXD01013434.1| | protein polymerization | P |
| gi|619307188|gb|GAXD01013434.1| | GTP catabolic process | P |
| gi|619307188|gb|GAXD01013434.1| | microtubule-based movement | P |
| gi|619307188|gb|GAXD01013434.1| | GTPase activity | F |
| gi|619307188|gb|GAXD01013434.1| | structural molecule activity | F |
| gi|619307188|gb|GAXD01013434.1| | cytoplasm | C |
| gi|619307188|gb|GAXD01013434.1| | GTP binding | F |
| gi|619307189|gb|GAXD01013433.1| | microtubule | C |
| gi|619307189|gb|GAXD01013433.1| | protein polymerization | P |
| gi|619307189|gb|GAXD01013433.1| | GTP catabolic process | P |
| gi|619307189|gb|GAXD01013433.1| | microtubule-based movement | P |
| gi|619307189|gb|GAXD01013433.1| | GTPase activity | F |
| gi|619307189|gb|GAXD01013433.1| | structural molecule activity | F |
| gi|619307189|gb|GAXD01013433.1| | cytoplasm | C |
| gi|619307189|gb|GAXD01013433.1| | GTP binding | F |
| gi|619307707|gb|GAXD01012915.1| | nucleosome | C |
| gi|619307707|gb|GAXD01012915.1| | DNA repair | P |
| gi|619307707|gb|GAXD01012915.1| | DNA binding | F |
| gi|619307707|gb|GAXD01012915.1| | nucleosome assembly | P |
| gi|619307707|gb|GAXD01012915.1| | nucleus | C |
| gi|619307738|gb|GAXD01012884.1| | sequence-specific DNA binding transcription factor activity | F |
| gi|619307738|gb|GAXD01012884.1| | transcription, DNA-templated | P |
| gi|619307745|gb|GAXD01012877.1| | extracellular matrix | C |
| gi|619307745|gb|GAXD01012877.1| | metalloendopeptidase activity | F |
| gi|619307759|gb|GAXD01012863.1| | non-membrane spanning protein tyrosine kinase activity | F |
| gi|619307759|gb|GAXD01012863.1| | protein phosphorylation | P |
| gi|619307759|gb|GAXD01012863.1| | ATP binding | F |
| gi|619308265|gb|GAXD01012357.1| | cytoplasm | C |
| gi|619308265|gb|GAXD01012357.1| | glycine N-methyltransferase activity | F |
| gi|619308265|gb|GAXD01012357.1| | folic acid binding | F |
| gi|619308265|gb|GAXD01012357.1| | methylation | P |
| gi|619308265|gb|GAXD01012357.1| | methionine metabolic process | P |
| gi|619308919|gb|GAXD01011703.1| | DNA metabolic process | P |
| gi|619308919|gb|GAXD01011703.1| | nucleic acid binding | F |
| gi|619308919|gb|GAXD01011703.1| | catalytic activity | F |
| gi|619309239|gb|GAXD01011383.1| | membrane | C |
| gi|619310929|gb|GAXD01009820.1| | oxidoreductase activity | F |
| gi|619310929|gb|GAXD01009820.1| | binding | F |
| gi|619311405|gb|GAXD01009344.1| | small GTPase regulator activity | F |
| gi|619311405|gb|GAXD01009344.1| | metal ion binding | F |
| gi|619311405|gb|GAXD01009344.1| | intracellular signal transduction | P |
| gi|619311405|gb|GAXD01009344.1| | protein phosphorylation | P |
| gi|619311405|gb|GAXD01009344.1| | ATP binding | F |
| gi|619311405|gb|GAXD01009344.1| | protein serine/threonine kinase activity | F |
| gi|619311890|gb|GAXD01008859.1| | structural constituent of cytoskeleton | F |
| gi|619311890|gb|GAXD01008859.1| | cytoskeletal anchoring at plasma membrane | P |
| gi|619311890|gb|GAXD01008859.1| | actin binding | F |
| gi|619311890|gb|GAXD01008859.1| | focal adhesion | C |
| gi|619311890|gb|GAXD01008859.1| | cell adhesion | P |
| gi|619311890|gb|GAXD01008859.1| | ruffle | C |
| gi|619311890|gb|GAXD01008859.1| | insulin receptor binding | F |
| gi|619311890|gb|GAXD01008859.1| | actin cytoskeleton | C |
| gi|619311959|gb|GAXD01008790.1| | catalytic activity | F |
| gi|619312043|gb|GAXD01008706.1| | sulfotransferase activity | F |
| gi|619312460|gb|GAXD01008289.1| | structural constituent of ribosome | F |
| gi|619312460|gb|GAXD01008289.1| | small ribosomal subunit | C |
| gi|619312460|gb|GAXD01008289.1| | translation | P |
| gi|619312460|gb|GAXD01008289.1| | rRNA binding | F |
| gi|619312554|gb|GAXD01008195.1| | nucleic acid binding | F |
| gi|619312554|gb|GAXD01008195.1| | ATP binding | F |
| gi|619312554|gb|GAXD01008195.1| | ATP-dependent helicase activity | F |
| gi|619312815|gb|GAXD01007934.1| | NADPH:quinone reductase activity | F |
| gi|619312815|gb|GAXD01007934.1| | palmitoyl-[acyl-carrier-protein] hydrolase activity | F |
| gi|619312815|gb|GAXD01007934.1| | ACP phosphopantetheine attachment site binding involved in fatty acid biosynthetic process | F |
| gi|619312815|gb|GAXD01007934.1| | [acyl-carrier-protein] S-malonyltransferase activity | F |
| gi|619312815|gb|GAXD01007934.1| | enoyl-[acyl-carrier-protein] reductase (NADPH, B-specific) activity | F |
| gi|619312815|gb|GAXD01007934.1| | [acyl-carrier-protein] S-acetyltransferase activity | F |
| gi|619312815|gb|GAXD01007934.1| | oleoyl-[acyl-carrier-protein] hydrolase activity | F |
| gi|619312815|gb|GAXD01007934.1| | 3-oxoacyl-[acyl-carrier-protein] reductase (NADPH) activity | F |
| gi|619312815|gb|GAXD01007934.1| | 3-oxoacyl-[acyl-carrier-protein] synthase activity | F |
| gi|619312815|gb|GAXD01007934.1| | myristoyl-[acyl-carrier-protein] hydrolase activity | F |
| gi|619312815|gb|GAXD01007934.1| | biosynthetic process | P |
| gi|619312815|gb|GAXD01007934.1| | zinc ion binding | F |
| gi|619312815|gb|GAXD01007934.1| | 3-hydroxypalmitoyl-[acyl-carrier-protein] dehydratase activity | F |
| gi|619312815|gb|GAXD01007934.1| | oxidation-reduction process | P |
| gi|619313173|gb|GAXD01007576.1| | zinc ion binding | F |
| gi|619313313|gb|GAXD01007436.1| | receptor activity | F |
| gi|619313313|gb|GAXD01007436.1| | protein kinase activity | F |
| gi|619313313|gb|GAXD01007436.1| | protein phosphorylation | P |
| gi|619313313|gb|GAXD01007436.1| | ATP binding | F |
| gi|619313324|gb|GAXD01007425.1| | cytoplasm | C |
| gi|619313324|gb|GAXD01007425.1| | flavin adenine dinucleotide binding | F |
| gi|619313324|gb|GAXD01007425.1| | thioredoxin-disulfide reductase activity | F |
| gi|619313324|gb|GAXD01007425.1| | NADP binding | F |
| gi|619313324|gb|GAXD01007425.1| | cell redox homeostasis | P |
| gi|619313324|gb|GAXD01007425.1| | oxidation-reduction process | P |
| gi|619313345|gb|GAXD01007404.1| | receptor activity | F |
| gi|619313345|gb|GAXD01007404.1| | integral component of membrane | C |
| gi|619313345|gb|GAXD01007404.1| | calcium ion binding | F |
| gi|619313838|gb|GAXD01006911.1| | metal ion binding | F |
| gi|619313838|gb|GAXD01006911.1| | intracellular | C |
| gi|619313838|gb|GAXD01006911.1| | acid-amino acid ligase activity | F |
| gi|619313838|gb|GAXD01006911.1| | protein ubiquitination | P |
| gi|619313838|gb|GAXD01006911.1| | ubiquitin-protein transferase activity | F |
| gi|619313951|gb|GAXD01006798.1| | cell redox homeostasis | P |
| gi|619313951|gb|GAXD01006798.1| | isomerase activity | F |
| gi|619314326|gb|GAXD01006423.1| | co-SMAD binding | F |
| gi|619314326|gb|GAXD01006423.1| | ubiquitin-dependent protein catabolic process | P |
| gi|619314326|gb|GAXD01006423.1| | ubiquitin thiolesterase activity | F |
| gi|619314326|gb|GAXD01006423.1| | mitotic nuclear division | P |
| gi|619314326|gb|GAXD01006423.1| | chromosome segregation | P |
| gi|619314326|gb|GAXD01006423.1| | BMP signaling pathway | P |
| gi|619314326|gb|GAXD01006423.1| | female gamete generation | P |
| gi|619314326|gb|GAXD01006423.1| | cysteine-type endopeptidase activity | F |
| gi|619314326|gb|GAXD01006423.1| | transforming growth factor beta receptor signaling pathway | P |
| gi|619314326|gb|GAXD01006423.1| | protein deubiquitination | P |
| gi|619314326|gb|GAXD01006423.1| | cytoplasm | C |
| gi|619314631|gb|GAXD01006118.1| | ribosome | C |
| gi|619314631|gb|GAXD01006118.1| | structural constituent of ribosome | F |
| gi|619314631|gb|GAXD01006118.1| | translation | P |
| gi|619314650|gb|GAXD01006099.1| | cytoskeleton organization | P |
| gi|619314650|gb|GAXD01006099.1| | actin binding | F |
| gi|619314650|gb|GAXD01006099.1| | zinc ion binding | F |
| gi|619314650|gb|GAXD01006099.1| | transferase activity | F |
| gi|619314677|gb|GAXD01006072.1| | lipid transport | P |
| gi|619314730|gb|GAXD01006019.1| | transport | P |
| gi|619314730|gb|GAXD01006019.1| | transferase activity | F |
| gi|619315339|gb|GAXD01005410.1| | intracellular | C |
| gi|619315339|gb|GAXD01005410.1| | ubiquitin-protein transferase activity | F |
| gi|619315339|gb|GAXD01005410.1| | acid-amino acid ligase activity | F |
| gi|619315339|gb|GAXD01005410.1| | RNA binding | F |
| gi|619315339|gb|GAXD01005410.1| | protein ubiquitination | P |
| gi|619315339|gb|GAXD01005410.1| | zinc ion binding | F |
| gi|619315603|gb|GAXD01005146.1| | myosin filament | C |
| gi|619315603|gb|GAXD01005146.1| | actin binding | F |
| gi|619315603|gb|GAXD01005146.1| | ATP binding | F |
| gi|619315603|gb|GAXD01005146.1| | motor activity | F |
| gi|619315604|gb|GAXD01005145.1| | myosin filament | C |
| gi|619315604|gb|GAXD01005145.1| | ATP binding | F |
| gi|619315604|gb|GAXD01005145.1| | motor activity | F |
| gi|619315605|gb|GAXD01005144.1| | myosin filament | C |
| gi|619315605|gb|GAXD01005144.1| | actin binding | F |
| gi|619315605|gb|GAXD01005144.1| | ATP binding | F |
| gi|619315605|gb|GAXD01005144.1| | motor activity | F |
| gi|619315825|gb|GAXD01004924.1| | palmitoyl-[acyl-carrier-protein] hydrolase activity | F |
| gi|619315825|gb|GAXD01004924.1| | ACP phosphopantetheine attachment site binding involved in fatty acid biosynthetic process | F |
| gi|619315825|gb|GAXD01004924.1| | [acyl-carrier-protein] S-malonyltransferase activity | F |
| gi|619315825|gb|GAXD01004924.1| | enoyl-[acyl-carrier-protein] reductase (NADPH, B-specific) activity | F |
| gi|619315825|gb|GAXD01004924.1| | [acyl-carrier-protein] S-acetyltransferase activity | F |
| gi|619315825|gb|GAXD01004924.1| | oleoyl-[acyl-carrier-protein] hydrolase activity | F |
| gi|619315825|gb|GAXD01004924.1| | 3-oxoacyl-[acyl-carrier-protein] reductase (NADPH) activity | F |
| gi|619315825|gb|GAXD01004924.1| | 3-oxoacyl-[acyl-carrier-protein] synthase activity | F |
| gi|619315825|gb|GAXD01004924.1| | myristoyl-[acyl-carrier-protein] hydrolase activity | F |
| gi|619315825|gb|GAXD01004924.1| | biosynthetic process | P |
| gi|619315825|gb|GAXD01004924.1| | zinc ion binding | F |
| gi|619315825|gb|GAXD01004924.1| | 3-hydroxypalmitoyl-[acyl-carrier-protein] dehydratase activity | F |
| gi|619315825|gb|GAXD01004924.1| | oxidation-reduction process | P |
| gi|619315943|gb|GAXD01004806.1| | nucleic acid binding | F |
| gi|619315943|gb|GAXD01004806.1| | nucleotide binding | F |
| gi|619316177|gb|GAXD01004572.1| | RNA binding | F |
| gi|619316327|gb|GAXD01004422.1| | protein dephosphorylation | P |
| gi|619316327|gb|GAXD01004422.1| | protein tyrosine/serine/threonine phosphatase activity | F |
| gi|619316444|gb|GAXD01004305.1| | hydrolase activity | F |
| gi|619316518|gb|GAXD01004231.1| | integral component of membrane | C |
| gi|619316518|gb|GAXD01004231.1| | phospho-N-acetylmuramoyl-pentapeptide-transferase activity | F |
| gi|619316661|gb|GAXD01004088.1| | regulation of transcription, DNA-templated | P |
| gi|619316661|gb|GAXD01004088.1| | DNA binding | F |
| gi|619316919|gb|GAXD01003830.1| | guanyl-nucleotide exchange factor activity | F |
| gi|619316919|gb|GAXD01003830.1| | GTPase binding | F |
| gi|619316919|gb|GAXD01003830.1| | GTP binding | F |
| gi|619317124|gb|GAXD01003625.1| | GTPase activator activity | F |
| gi|619317124|gb|GAXD01003625.1| | intracellular | C |
| gi|619317124|gb|GAXD01003625.1| | small GTPase regulator activity | F |
| gi|619317124|gb|GAXD01003625.1| | regulation of small GTPase mediated signal transduction | P |
| gi|619317267|gb|GAXD01003482.1| | hydrolase activity | F |
| gi|619317377|gb|GAXD01003372.1| | intracellular | C |
| gi|619317920|gb|GAXD01002829.1| | S-acyltransferase activity | F |
| gi|619317920|gb|GAXD01002829.1| | oxidoreductase activity | F |
| gi|619317920|gb|GAXD01002829.1| | acyl-[acyl-carrier-protein] hydrolase activity | F |
| gi|619317920|gb|GAXD01002829.1| | cofactor binding | F |
| gi|619317920|gb|GAXD01002829.1| | metabolic process | P |
| gi|619317972|gb|GAXD01002777.1| | phospholipid binding | F |
| gi|619317973|gb|GAXD01002776.1| | 1-phosphatidylinositol binding | F |
| gi|619317973|gb|GAXD01002776.1| | clathrin coat | C |
| gi|619317973|gb|GAXD01002776.1| | clathrin binding | F |
| gi|619317973|gb|GAXD01002776.1| | clathrin coat assembly | P |
| gi|619318144|gb|GAXD01002605.1| | sulfotransferase activity | F |
| gi|619318417|gb|GAXD01002332.1| | binding | F |
| gi|619318520|gb|GAXD01002229.1| | zinc ion binding | F |
| gi|619318520|gb|GAXD01002229.1| | oxidoreductase activity | F |
| gi|619318520|gb|GAXD01002229.1| | oxidation-reduction process | P |
| gi|619318602|gb|GAXD01002147.1| | cell redox homeostasis | P |
| gi|619318602|gb|GAXD01002147.1| | peroxiredoxin activity | F |
| gi|619318602|gb|GAXD01002147.1| | antioxidant activity | F |
| gi|619318695|gb|GAXD01002054.1| | microtubule motor activity | F |
| gi|619318695|gb|GAXD01002054.1| | dynein complex | C |
| gi|619318695|gb|GAXD01002054.1| | ATP catabolic process | P |
| gi|619318695|gb|GAXD01002054.1| | ATP binding | F |
| gi|619318695|gb|GAXD01002054.1| | microtubule-based movement | P |
| gi|619318695|gb|GAXD01002054.1| | ATPase activity | F |
| gi|619318948|gb|GAXD01001801.1| | binding | F |
| gi|619319041|gb|GAXD01001708.1| | regulation of transcription, DNA-templated | P |
| gi|619319041|gb|GAXD01001708.1| | sequence-specific DNA binding transcription factor activity | F |
| gi|619319041|gb|GAXD01001708.1| | transcription, DNA-templated | P |
| gi|619319041|gb|GAXD01001708.1| | nucleus | C |
| gi|619319054|gb|GAXD01001695.1| | metalloendopeptidase activity | F |
| gi|619319054|gb|GAXD01001695.1| | proteolysis | P |
| gi|619319054|gb|GAXD01001695.1| | calcium ion binding | F |
| gi|619319054|gb|GAXD01001695.1| | extracellular matrix | C |
| gi|619319054|gb|GAXD01001695.1| | zinc ion binding | F |
| gi|619319202|gb|GAXD01001547.1| | mitochondrion | C |
| gi|619319202|gb|GAXD01001547.1| | transferase activity | F |
| gi|619319227|gb|GAXD01001522.1| | metal ion binding | F |
| gi|619319227|gb|GAXD01001522.1| | intracellular | C |
| gi|619319227|gb|GAXD01001522.1| | acid-amino acid ligase activity | F |
| gi|619319227|gb|GAXD01001522.1| | protein ubiquitination | P |
| gi|619319227|gb|GAXD01001522.1| | ubiquitin-protein transferase activity | F |
| gi|619319236|gb|GAXD01001513.1| | regulation of transcription, DNA-templated | P |
| gi|619319236|gb|GAXD01001513.1| | DNA binding | F |
| gi|619319303|gb|GAXD01001446.1| | translation elongation factor activity | F |
| gi|619319303|gb|GAXD01001446.1| | eukaryotic translation elongation factor 1 complex | C |
| gi|619319303|gb|GAXD01001446.1| | translational elongation | P |
| gi|619319541|gb|GAXD01001208.1| | cation transmembrane transporter activity | F |
| gi|619319541|gb|GAXD01001208.1| | cation transport | P |
| gi|619319722|gb|GAXD01001027.1| | plasma membrane part | C |
| gi|619319722|gb|GAXD01001027.1| | transmembrane transport | P |
| gi|619319722|gb|GAXD01001027.1| | lactose transport | P |
| gi|619319722|gb|GAXD01001027.1| | trehalose transport | P |
| gi|619319722|gb|GAXD01001027.1| | trehalose transmembrane transporter activity | F |
| gi|619319722|gb|GAXD01001027.1| | integral component of membrane | C |
| gi|619319722|gb|GAXD01001027.1| | maltose transport | P |
| gi|619319722|gb|GAXD01001027.1| | sucrose transport | P |
| gi|619319820|gb|GAXD01000929.1| | transport | P |
| gi|619319820|gb|GAXD01000929.1| | transferase activity | F |
| gi|619320029|gb|GAXD01000720.1| | protein dephosphorylation | P |
| gi|619320029|gb|GAXD01000720.1| | protein tyrosine/serine/threonine phosphatase activity | F |
| gi|619320037|gb|GAXD01000712.1| | nucleic acid binding | F |
| gi|619320037|gb|GAXD01000712.1| | nucleotide binding | F |
| gi|619320076|gb|GAXD01000673.1| | regulation of ARF protein signal transduction | P |
| gi|619320076|gb|GAXD01000673.1| | intracellular | C |
| gi|619320076|gb|GAXD01000673.1| | ARF guanyl-nucleotide exchange factor activity | F |
| gi|619320078|gb|GAXD01000671.1| | zinc ion binding | F |
| gi|619320078|gb|GAXD01000671.1| | oxidoreductase activity | F |
| gi|619320078|gb|GAXD01000671.1| | oxidation-reduction process | P |
| gi|619320106|gb|GAXD01000643.1| | biosynthetic process | P |
| gi|619320106|gb|GAXD01000643.1| | nucleotidyltransferase activity | F |
| gi|619320202|gb|GAXD01000547.1| | hydrolase activity, acting on glycosyl bonds | F |
| gi|619320203|gb|GAXD01000546.1| | hydrolase activity, acting on glycosyl bonds | F |
| gi|619320352|gb|GAXD01000397.1| | membrane | C |
| gi|619320352|gb|GAXD01000397.1| | single-organism transport | P |
| gi|619320352|gb|GAXD01000397.1| | transporter activity | F |
| gi|619320416|gb|GAXD01000333.1| | zinc ion binding | F |
| gi|619320416|gb|GAXD01000333.1| | nucleic acid binding | F |
| gi|619320416|gb|GAXD01000333.1| | intracellular | C |
| gi|619320416|gb|GAXD01000333.1| | nucleotide binding | F |
| gi|619320703|gb|GAXD01000046.1| | hydrolase activity | F |
|  |  |  |
|  |  |  |
|  |  |  |
|  |  |  |
|  |  |  |
| **F** | **Molecular Function** |  |
| **P** | **Biological Process** |  |
| **C** | **Cellular Component** |  |
| **The first report of miRNAome from a thysanopteran insect, Thrips palmi Karny using high-throughput sequencing.**  **Authors : K. B. Rebijith, R. Asokan, H. Ranjitha Hande and N. K. Krishna Kumar** | | |
