# Supplementary material for: The First Report of miRNAs from a Thysanopteran Insect, Thrips palmi Karny Using High-Throughput Sequencing
Source: PLoS One. 2016 Sep 29;11(9):e0163635. doi: 10.1371/journal.pone.0163635 (PMC5042526; doi:10.1371/journal.pone.0163635)
Supplement: S6 Table — (DOC) [file pone.0163635.s006.doc]

| **Supplementary Table S6. Potential targets for the identified novel miRNAs with Transcriptome sequences of *F. occidentalis*.** | | | | | | | | | | | |
| --- | --- | --- | --- | --- | --- | --- | --- | --- | --- | --- | --- |
| **Novel miRNA** | **Target** | **Protein Name** | **score** | **energy** | **miRNA.start** | **miRNA.end** | **Target.start** | **Target.end** | **Alignment Length** | **Alignment Percentage** | |
| tpa-miR-N1 | gi|619313681|gb|GAXD01007068.1| | protein scai | 186 | -29.6 | 2 | 19 | 301 | 324 | 17 | 94.12% | 100.00% |
| tpa-miR-N1 | gi|619298151|gb|GAXD01022470.1| | 60s ribosomal protein l27a | 161 | -23.98 | 2 | 14 | 277 | 300 | 12 | 91.67% | 100.00% |
| tpa-miR-N1 | gi|619305719|gb|GAXD01014903.1| | rho gtpase-activating protein 44-like isoform x1 | 161 | -19.26 | 2 | 14 | 453 | 476 | 12 | 91.67% | 100.00% |
| tpa-miR-N1 | gi|619298732|gb|GAXD01021889.1| | transforming growth factor-beta-induced protein ig-h3 | 175 | -30.46 | 2 | 16 | 488 | 511 | 14 | 100.00% | 100.00% |
| tpa-miR-N2 | gi|619298659|gb|GAXD01021962.1| | alpha- sarcomeric isoform x2 | 161 | -27.77 | 2 | 14 | 3904 | 3924 | 12 | 91.67% | 100.00% |
| tpa-miR-N2 | gi|619298660|gb|GAXD01021961.1| | alpha- sarcomeric isoform x2 | 161 | -27.77 | 2 | 14 | 3919 | 3939 | 12 | 91.67% | 100.00% |
| tpa-miR-N3 | gi|619290623|gb|GAXD01029515.1| | cg1785- partial | 166 | -26.36 | 2 | 15 | 165 | 188 | 13 | 92.31% | 100.00% |
| tpa-miR-N4 | gi|619307432|gb|GAXD01013190.1| | 97 kda heat shock protein isoform x2 | 161 | -15.51 | 2 | 14 | 2968 | 2991 | 12 | 91.67% | 100.00% |
| tpa-miR-N4 | gi|619314482|gb|GAXD01006267.1| | ancient ubiquitous protein 1-like | 161 | -19.27 | 2 | 14 | 517 | 540 | 12 | 91.67% | 100.00% |
| tpa-miR-N4 | gi|619297500|gb|GAXD01023121.1| | uncharacterized membrane protein ddb_g0293934-like | 165 | -20 | 2 | 14 | 477 | 500 | 12 | 100.00% | 100.00% |
| tpa-miR-N4 | gi|619297501|gb|GAXD01023120.1| | uncharacterized membrane protein ddb_g0293934-like | 165 | -20 | 2 | 14 | 477 | 500 | 12 | 100.00% | 100.00% |
| tpa-miR-N4 | gi|619292746|gb|GAXD01027392.1| | PREDICTED: uncharacterized protein LOC102808229 | 160 | -17.5 | 2 | 13 | 222 | 245 | 11 | 100.00% | 100.00% |
| tpa-miR-N4 | gi|619315090|gb|GAXD01005659.1| | elongator complex protein 1 | 160 | -18.86 | 2 | 13 | 366 | 389 | 11 | 100.00% | 100.00% |
| tpa-miR-N5 | gi|619314440|gb|GAXD01006309.1| | probable trafficking protein particle complex subunit 2 | 162 | -20.98 | 2 | 15 | 137 | 157 | 13 | 84.62% | 100.00% |
| tpa-miR-N5 | gi|619305218|gb|GAXD01015404.1| | low-density lipoprotein receptor-related protein partial | 163 | -19.13 | 2 | 16 | 2821 | 2841 | 14 | 78.57% | 100.00% |
| tpa-miR-N5 | gi|619300562|gb|GAXD01020060.1| | #N/A | 160 | -20.69 | 2 | 13 | 183 | 203 | 11 | 100.00% | 100.00% |
| tpa-miR-N6 | gi|619317313|gb|GAXD01003436.1| | acyl carrier mitochondrial-like isoform x1 | 160 | -19.61 | 2 | 13 | 243 | 266 | 11 | 100.00% | 100.00% |
| tpa-miR-N8 | gi|619306775|gb|GAXD01013847.1| | #N/A | 161 | -19.04 | 2 | 14 | 58 | 80 | 12 | 91.67% | 100.00% |
| tpa-miR-N8 | gi|619317484|gb|GAXD01003265.1| | #N/A | 161 | -16.17 | 2 | 14 | 92 | 114 | 12 | 91.67% | 100.00% |
| tpa-miR-N8 | gi|619304098|gb|GAXD01016524.1| | metastasis-associated protein mta1 | 160 | -21.77 | 2 | 13 | 165 | 187 | 11 | 100.00% | 100.00% |
| tpa-miR-N9 | gi|619299699|gb|GAXD01020922.1| | hypothetical protein L798_02279 | 160 | -34.76 | 2 | 13 | 32 | 53 | 11 | 100.00% | 100.00% |
| tpa-miR-N10 | gi|619320345|gb|GAXD01000404.1| | rab-protein 10 cg17060-pa | 177 | -24.23 | 2 | 22 | 2889 | 2911 | 20 | 100.00% | 100.00% |
| tpa-miR-N10 | gi|619292462|gb|GAXD01027676.1| | #N/A | 177 | -30.01 | 2 | 22 | 74 | 96 | 20 | 100.00% | 100.00% |
| tpa-miR-N10 | gi|619289677|gb|GAXD01030461.1| | #N/A | 177 | -30.01 | 2 | 22 | 65 | 87 | 20 | 91.67% | 100.00% |
| tpa-miR-N10 | gi|619304165|gb|GAXD01016457.1| | #N/A | 176 | -24.78 | 2 | 22 | 26 | 49 | 21 | 91.67% | 100.00% |
| tpa-miR-N10 | gi|619318457|gb|GAXD01002292.1| | odd oz protein | 175 | -25.38 | 2 | 22 | 2351 | 2372 | 20 | 100.00% | 100.00% |
| tpa-miR-N10 | gi|619318201|gb|GAXD01002548.1| | PREDICTED: hypothetical protein LOC411065 isoform 1 | 173 | -26.82 | 2 | 18 | 597 | 619 | 16 | 84.62% | 100.00% |
| tpa-miR-N10 | gi|619300045|gb|GAXD01020577.1| | PREDICTED: hypothetical protein LOC411065 isoform 1 | 173 | -26.82 | 2 | 18 | 218 | 240 | 16 | 84.62% | 100.00% |
| tpa-miR-N10 | gi|619302164|gb|GAXD01018458.1| | glutamyl-trna amidotransferase subunit mitochondrial-like | 172 | -22.48 | 2 | 21 | 79 | 101 | 19 | 100.00% | 100.00% |
| tpa-miR-N10 | gi|619318708|gb|GAXD01002041.1| | neuropathy target esterase sws-like isoform 1 | 172 | -22.89 | 2 | 21 | 847 | 869 | 19 | 100.00% | 100.00% |
| tpa-miR-N10 | gi|619297800|gb|GAXD01022821.1| | down syndrome critical region protein 3 | 172 | -22.17 | 2 | 22 | 40 | 63 | 21 | 100.00% | 100.00% |
| tpa-miR-N10 | gi|619295618|gb|GAXD01024520.1| | #N/A | 172 | -22.17 | 2 | 21 | 681 | 703 | 19 | 100.00% | 100.00% |
| tpa-miR-N10 | gi|619294885|gb|GAXD01025253.1| | protein flightless-1 | 180 | -31.13 | 2 | 22 | 178 | 201 | 21 | 100.00% | 100.00% |
|  |  |  |  |  |  |  |  |  |  |  |  |
|  |  |  |  |  |  |  |  |  |  |  |  |
|  |  |  |  |  |  |  |  |  |  |  |  |
| **The first report of miRNAome from a thysanopteran insect, Thrips palmi Karny using high-throughput sequencing.**  **Authors : K. B. Rebijith, R. Asokan, H. Ranjitha Hande and N. K. Krishna Kumar** | | | | | | | | | | | |
