# Supplementary material for: The First Report of miRNAs from a Thysanopteran Insect, Thrips palmi Karny Using High-Throughput Sequencing
Source: PLoS One. 2016 Sep 29;11(9):e0163635. doi: 10.1371/journal.pone.0163635 (PMC5042526; doi:10.1371/journal.pone.0163635)
Supplement: S7 Table — (DOC) [file pone.0163635.s007.doc]

| **Supplementary Table S7.**  **Complete Functional categories of gene ontology classification of the putative target genes for the novel miRNAs against transcriptome sequences of *F. occidentalis*.** | | |
| --- | --- | --- |
| **Seq Name** | **Functional categories** | **Functional class** |
| gi|619298660|gb|GAXD01021961.1| | actin filament bundle assembly; actin crosslink formation; | P |
| gi|619298659|gb|GAXD01021962.1| | actin filament bundle assembly; actin crosslink formation; | P |
| gi|619314440|gb|GAXD01006309.1| | ER to Golgi vesicle-mediated transport; | P |
| gi|619317313|gb|GAXD01003436.1| | fatty acid biosynthetic process; | P |
| gi|619304098|gb|GAXD01016524.1| | regulation of transcription, DNA-dependent; | P |
| gi|619313681|gb|GAXD01007068.1| | transcription, DNA-dependent; | P |
| gi|619320345|gb|GAXD01000404.1| | small GTPase mediated signal transduction | P |
| gi|619320345|gb|GAXD01000404.1| | protein transport | P |
| gi|619314440|gb|GAXD01006309.1| | intracellular; | C |
| gi|619304098|gb|GAXD01016524.1| | nucleus; | C |
| gi|619298151|gb|GAXD01022470.1| | ribonucleoprotein complex; | C |
| gi|619298660|gb|GAXD01021961.1| | calcium ion binding; actin filament binding; | F |
| gi|619298659|gb|GAXD01021962.1| | calcium ion binding; actin filament binding; | F |
| gi|619304098|gb|GAXD01016524.1| | chromatin binding; sequence-specific DNA binding transcription factor activity; zinc ion binding; sequence-specific DNA binding; | F |
| gi|619307432|gb|GAXD01013190.1| | nucleotide binding; | F |
| gi|619313681|gb|GAXD01007068.1| | transcription corepressor activity; | F |
| gi|619320345|gb|GAXD01000404.1| | GTP binding | F |
| gi|619294885|gb|GAXD01025253.1| | actin binding | F |
| **The first report of miRNAome from a thysanopteran insect, Thrips palmi Karny using high-throughput sequencing.**  **Authors : K. B. Rebijith, R. Asokan, H. Ranjitha Hande and N. K. Krishna Kumar** | | |
